# An Epigenetics‐Inspired DNA‐Based Data Storage System

**DOI:** 10.1002/anie.201605531

**Published:** 2016-07-21

**Authors:** Clemens Mayer, Gordon R. McInroy, Pierre Murat, Pieter Van Delft, Shankar Balasubramanian

**Affiliations:** ^1^ Department of ChemistryUniversity of CambridgeLensfield RoadCambridgeCB2 1EWUK; ^2^ Cancer Research, UK, Cambridge Institute, Li Ka Shing CentreUniversity of CambridgeRobinson WayCambridgeCB2 0REUK; ^3^ School of Clinical MedicineUniversity of CambridgeCambridgeCB2 0SPUK

**Keywords:** DNA, epigenetics, information storage, sequencing, supramolecular chemistry

## Abstract

Biopolymers are an attractive alternative to store and circulate information. DNA, for example, combines remarkable longevity with high data storage densities and has been demonstrated as a means for preserving digital information. Inspired by the dynamic, biological regulation of (epi)genetic information, we herein present how binary data can undergo controlled changes when encoded in synthetic DNA strands. By exploiting differential kinetics of hydrolytic deamination reactions of cytosine and its naturally occurring derivatives, we demonstrate how multiple layers of information can be stored in a single DNA template. Moreover, we show that controlled redox reactions allow for interconversion of these DNA‐encoded layers of information. Overall, such interlacing of multiple messages on synthetic DNA libraries showcases the potential of chemical reactions to manipulate digital information on (bio)polymers.

Means to access, circulate, and preserve information have shaped human society by increasing knowledge, stimulating the economy and enriching culture. In this respect, the development of optical and magnetic storage devices has facilitated an unprecedented increase of accessible information, but their limited shelf lives and storage densities have prompted a search for alternative data carriers.^1^ Current lines of research focus on further increasing storage densities by compacting information into single atoms,^2^ supramolecular systems,^3^ or biopolymers.^4^ Nucleic acids, for example, are remarkably compact and long‐lived, and have been proposed for storing digital information. The advent of high‐throughput oligonucleotide synthesis^5^ and DNA sequencing^6^ has allowed DNA‐based data storage to rapidly progress from proof‐of‐concept studies toward systems that can rival established storage media.^7^ While such systems have enabled writing and reading of non‐trivial amounts of information with synthetic DNA templates, the “one template, one information layer” coding scheme employed (Figure 1 A) is in stark contrast to nature's dynamic control over the primary information encoded in genomes. In order to produce a complex organism from a single genetic makeup, cells regulate access to different layers of information by modifying histone proteins and DNA nucleobases (Figure 1 B).^8^ This epigenetic regulation orchestrates processes such as gene expression and ultimately drives cell differentiation. Herein, we apply principles from biological regulation toward DNA data storage, through the controlled chemical transformations of nucleobases^9^ and their associated binary value. As a result, we were able to (reversibly) recover multiple layers of binary data from a single DNA template (Figure 1 C).

**Figure 1:**
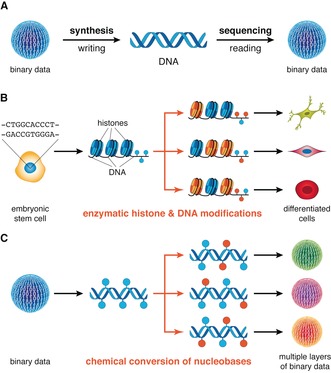
Emulating nature's control over biopolymers for information storage. A) In DNA‐based data storage binary data is written onto oligonucleotides by synthesis and read by sequencing on demand. B) The epigenome (histone and DNA modifications) of an embryonic stem cell undergoes controlled changes upon cell differentiation leading to the development of different phenotypes from the same genetic information. C) The use of selective chemical reactions facilitates alteration of the primary sequence of a synthetic DNA template, and thus the retrieval of multiple layers of information from it.

Moving away from the conventional concatenation approach to encode information on DNA,^7^ we examined the possibility of merging several strings of information within the same DNA molecule. In a first step, we investigated an irreversible chemical transformation to recover two, separate layers of information from a single DNA template (Figure 2 A). For this, we exploited the bisulfite ion (HSO_3_^−^)‐catalyzed hydrolytic deamination of cytosine (C) to uracil (U, Figure 2 B).^10^ The distinct Watson–Crick base pairing properties of C and U mean that after PCR amplification, bisulfite converted positions yield 1:1 mixtures of C:thymine (T) for the forward strand and 1:1 mixtures of guanine (G):adenine (A) for the reverse strand (Figure 2 C). These splits arise, because cytosine deamination reactions on one strand are accompanied by retention of the cognate G base on the complementary one. We surmised that bisulfite‐mediated C‐to‐U conversions could be used to alter bits encoded by C nucleobases and thus allow for merging of two information layers in the same DNA template. Specifically, while C positions encode for 0 when sequenced directly, the 1:1 mixture of C and T that results from chemical conversion is registered as 1. Conversely, G positions that encode for 1 before bisulfite conversion are transformed to 0 following the chemical treatment (Figure 2 C). When combined with A′s and T′s (encoding for 0 and 1, respectively) that are unaffected by bisulfite treatment, appropriately designed sequences (see Supporting Information) have two sense meanings before and after bisulfite conversion. For example, Figure 2 D reports our sequencing results for a stretch of 40 nucleotides that encodes for ASCII representations of two words. While we obtained “BLACK” in the absence of chemical treatment, bisulfite‐induced C‐to‐U conversion generates “WHITE”.

**Figure 2:**
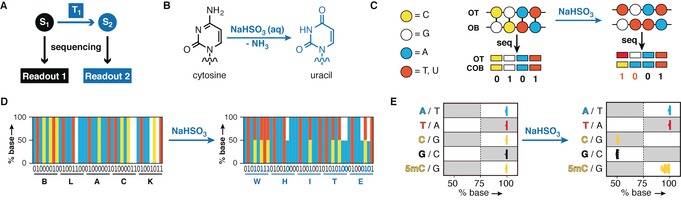
Bisulfite sequencing enables two‐layer encoding in a single nucleic acid template. A) A chemical transformation, T_1_, can transform an initial state, S_1_, into a distinct information state, S_2_. Upon sequencing of both information states, different readouts are obtained. B) Bisulfite mediated cytosine to uracil conversion. C) Deamination of C′s in both the original top (OT) and original bottom (OB) strand with retention of the respective G gives rise to 1:1 mixtures of C:T or G:A reads at these positions after PCR amplification and sequencing (COB=complementary original bottom). Reassigning the binary digits at these positions unravels a second layer of information from a single molecule of DNA. D) Proof‐of‐concept two‐layer encoding by bisulfite‐induced bit conversion. Stacked bar charts display the measured percentage base at a given position across all sequencing reads (same color code applies as for panel C). Shown is a 40 base pair region of an oligonucleotide, which encodes for binary representations of the ASCII text “BLACK” before, and “WHITE” after bisulfite treatment. E) upon bisulfite conversion, a library of oligonucleotides, originally encoding for the first stanza of Edgar A. Poe's The Raven, is read as the second stanza. Sequencing results are presented as percent base calls of 2668 positions. Bisulfite treatment affects 440 C and 420 G positions while 971 A, 789 T, and 68 5mC positions remain unchanged. Gray and white areas denote ranges in which bases are assigned as either 1 or 0 in the reading process. The threshold for calling a bit switch was set at 75 %.

To assess the robustness of this two‐layer encoding strategy, we designed and synthesized a library of oligonucleotides encoding a binary representation of the first stanza of The Raven by Edgar A. Poe (for details, see Figure S1 and the Supporting Information).^11^ When we subjected the same library to bisulfite‐catalyzed cytosine conversion before sequencing, we recovered the second stanza of the poem as the readout. Overall, our strategy proved to be efficient and selective (Figure 2 E). Without assuming any prior knowledge of the information, we recovered both stanzas error‐free. In the process, 860 positions were selectively deaminated upon chemical treatment (99.35±0.35 % conversion, Figure S1). In the design we introduced 5‐methyl cytosine (5mC), which is largely resistant to bisulfite‐conversion, at 68 positions to balance the GC content (37 % in the library prior conversion) and avoid extended homopolymer runs, which can be problematic for high throughput sequencing techniques.^12^ Since 5mC deaminates 100 times slower than C upon bisulfite treatment,^13^ these positions register as 0 in both readouts. We confirmed that at 5mC positions 97.0±2.1 % of sequencing reads indicated non‐conversion (i.e. still read as C during sequencing), and therefore retention of the initial binary value of 0 (Figure 2 E and Figure S1). Our two‐layer encoding Scheme is reminiscent of the C deamination process catalyzed by enzymes, such as the Activation‐Induced Cytidine Deaminase, to produce antibody diversity in B cells.^14^

A three‐layer encoding strategy can be achieved when incorporating an additional chemical transformation that alters a nucleobase, and, as a result, its associated binary information. As depicted in Figure 3 A, this would give rise to another information state, which encodes for a third, distinct message within a synthetic DNA template. Toward this end, we took advantage of the selective, potassium perruthenate (KRuO_4_) oxidation of 5‐hydroxymethylcytosine (5hmC) into 5‐formylcytosine (5fC) and 5‐carboxycytosine (5caC, Figure 3 B).^15^ 5fC and 5caC are both converted to uracil upon reaction with bisulfite, while 5hmC forms cytosine‐5‐methylenesulfonate which is read as a C upon DNA sequencing. As such, the resultant primary sequence readout of DNA that initially comprises 5hmC is different depending on whether or not chemical oxidation is carried out prior to a bisulfite‐reaction. When a DNA library also comprises 5mC, three‐layer encoding can be achieved. Assigning bit switches at positions that undergo changes following the use of the described chemical transformations, we recovered three strings of information from the same template (Figure 3 C). First, in the absence of chemical treatment, sequencing of the DNA library revealed a first message. Next, by combining KRuO_4_ oxidation with bisuflite‐catalyzed hydrolytic deamination, a second information layer is revealed. This process also identifies all 5mC positions present in the DNA library, as they are the only cytosine species read as C. Finally, by inverting the binary values at these positions in the third information state, which is obtained by omitting the oxidation step before bisulfite treatment, we recover a third information layer. Following this scheme, we designed an oligonucleotide that encodes simultaneously for ASCII representations of the words “BLACK”, “WHITE”, and “COLOR” (Figure 3 D).

**Figure 3:**
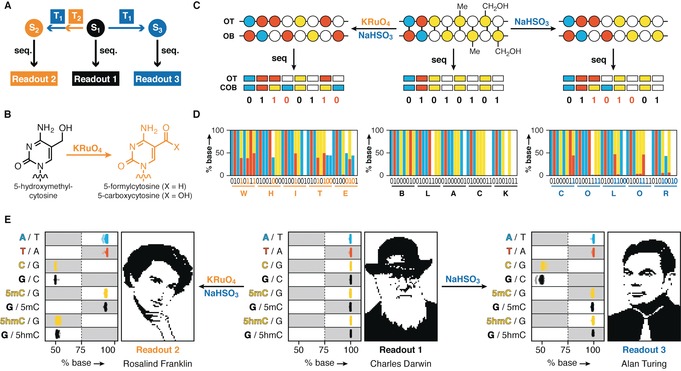
Chemical alteration of modified nucleobases enables three‐layer encoding in DNA. A) Chemical transformations T_1_ and T_2_ can be combined to access a new information state, S_2_, while the use of transformation T_1_ generates a third information state, S_3_, from the initial state S_1_. Upon sequencing, this strategy results in three distinct readouts. B) KRuO_4_‐mediated conversion of 5hmC (T_2_) to 5fC or 5caC. C) Use of KRuO_4_ and bisulfite‐mediated transformation for the decoding of three layers of information encoded within the same DNA template. Interconversion of bits rely on identifying 1:1 mixtures of C:T and G:A positions after chemical conversion (same color code as Figure 2 C is used). Sequencing before chemical conversion uncovers the first message, while oxidation and subsequent bisulfite treatment (left side) reveals a second layer of information. 5mC positions are also identified by this procedure, as they are the only cytosine species to be read as C when sequenced. By assigning the opposite binary values at the 5mC positions to the information state obtained by the bisulfite reaction of the original template (S_3_, right side), a third message is revealed. D) Three‐layer encoding proof‐of‐concept. Shown is a 40 base pair region of an oligonucleotide, which encodes for binary representation of the ASCII text “BLACK”, “WHITE” and “COLOR” before and after chemical transformations. E) Run length and Elias gamma encoded binary portraits (see the Supporting Information) of Charles Darwin, Rosalind Franklin and Alan Turing were stored in a single DNA template. Without prior knowledge of the information, reading of the synthesized oligonucleotide library by direct sequencing (middle), oxidation and bisulfite treatment followed by sequencing (left), and bisulfite treatment then sequencing (right), each retrieves one portrait. Recovery of the three layers of information relies on selectively switching a total of 1380 binary values (479 C, 488 5mC, and 413 5hmC). Sequencing results are presented as percent base calls of 2016 positions. Gray and white boxes denote the areas in which bases are called as 1 and 0 during bit assignment.

To exemplify the robustness and generality of our three‐layer encoding strategy, we designed and synthesized a library of oligonucleotides comprising A, C, G, T, 5mC and 5hmC that simultaneously encodes for three images (Figure 3 E and the Supporting Information).^16^ Sequencing data from the original DNA template can be decoded to give a picture of Charles Darwin. Consecutive treatment with KRuO_4_ and NaHSO_3_ oxidized 413 5hmCs and deaminated a total of 892 positions (i.e. all 5hmCs and Cs are converted to T in the final sequence readout) in the library and revealed a portrait of Rosalind Franklin. As described above, this process also identified 488 5mC positions. When assigning the opposite binary values to these positions in the third information state obtained by bisulfite treatment, we recovered a picture of Alan Turing (Figure 3 E). Both the oxidation and bisulfite reactions proved to be robust and selective: while A, T, and 5mC positions remained unchanged by the oxidation and/or bisulfite treatment in all experiments (>98 % retention of bases), 5hmCs were efficiently converted (96.2±2.1 %) when oxidized but were retained (99.4±0.3 %) in the absence of KRuO_4_. Bisulfite conversion of unmodified C′s to T′s (>98.0 %) was independent of the oxidation step (Table S1). The efficiency and selectivity of all employed transformations supports the scalability of the overall approach (see the Supporting Information for further discussion).

The reversible addition, removal and interconversion of DNA modifications, through the demethylation of DNA mediated by the Ten‐eleven translocation (TET) enzymes for example, are vital to control the expression of information encoded in genomes.^17^ To mimic this type of control in synthetic DNA templates we envisioned incorporating the oxidation reaction of 5hmC into a redox cycle (Figure 4 A). Oxidation conditions were optimized to enable the selective transformation of 5hmC to 5fC (Figure S2), and we employed NaBH_4_ as a reducing agent to transform the oxidation‐derived 5fC back to 5hmC (Figure 4 B).^18^ These alternating reactions enabled the interconversion of informational states (5hmC→5fC→5hmC) as exemplified in Figure 4 C. To assess the proficiency of the employed chemical transformations we performed five consecutive redox cycles on the portraits‐encoding library. When following the conversion efficiency of 5hmC positions over the course of these 10 transformations we observed the desired cycling behavior, while C and 5mC positions remain largely unaffected (Figure 4 D). Oxidation and reduction steps displayed mean conversion efficiencies over the 5 full cycles of 83.28±4.43 % and 83.55±10.77 %, respectively for 5hmC and 5fC (see Table S2). The apparent decrease in 5hmC reactivity over five cycles may reflect a degree of over oxidation to 5caC, which cannot be reduced by NaBH_4_. Overall, our redox chemistry for this reversible recovery of multiple information layers was efficient and selective, and in its current state enabled the correct bit recovery for 5hmC positions over 4 full cycles, and >95 % after the fifth reduction (Table S2).

**Figure 4:**
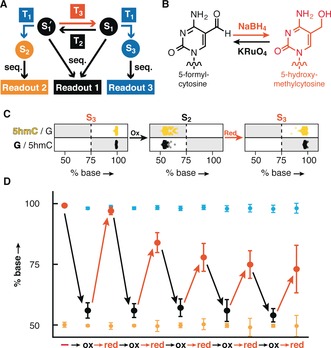
Interconversion of three information layers encoded in DNA. A) Combining reversible chemical transformations (T_2_ and T_3_) allows for cycling between the information states S_1_ and S_1_′, and enables the reversible encoding of three layers of information in DNA. B) Redox interconversion between 5hmC and 5fC by KRuO_4_ (T_2_) and NaBH_4_ (T_3_). C) Sequencing results of 413 5hmC positions within the portraits‐encoding DNA library (Figure 3 E) over one full redox cycle. Starting from a fully deaminated reduced state (S_1_), mild oxidation prior bisulfite treatment converts all 5hmC positions into S_1_′, while subsequent reduction restores the initial state of the oligonucleotide library. Conversion efficiencies are measured by subsequent bisulfite treatment to recover S_2_ and S_3_. D) Sequencing results for 5hmC positions (in % cytosine called, data represent mean values±standard deviation, *n*=413) over five full cycles (red and black points represent the oxidized and reduced states of the 5hmC′s, respectively). C′s (yellow, n=479) and 5mC′s (blue, n=488) are not affected by the chemical transformations. 5hmC positions, on average, remain distinct from other cytosine species after five cycles.

In this manuscript we demonstrate the potential of chemical reactions to manipulate digital information encoded within DNA. While our work focused on storing multiple data sets in one library—a strategy reminiscent of steganography—it is noteworthy that multilayer encoding represents an enticing approach to maximize storage capabilities of DNA templates. The information content of additional layers could be repurposed for different tasks, such as error‐correcting algorithms or encoding barcodes that are usually installed into synthetic libraries at the expense of storage space.^7^ The use of additional modified nucleobases (see Figure S3 for an example)^19^ together with reversible chemical reactions and direct sequencing readouts (e.g. nanopore sequencing) should enable the development of more complex systems.^20^ One significant challenge lies in the engineering of polymerases and other enzymes that will allow for amplification and sequencing of templates containing modified nucleobase.^21^ Future efforts are likely to expand on our approach by also employing non‐natural, sequence‐specific oligomers^22^ that will enable greater control over the encoded digital information. Ultimately, such developments may permit the design of multistable DNA systems that could facilitate the development of operative, molecular computers, such as Turing machines.^23^

## Supporting information

As a service to our authors and readers, this journal provides supporting information supplied by the authors. Such materials are peer reviewed and may be re‐organized for online delivery, but are not copy‐edited or typeset. Technical support issues arising from supporting information (other than missing files) should be addressed to the authors.

Supplementary
