# Supplementary material for: An Epigenetics‐Inspired DNA‐Based Data Storage System
Source: Angew Chem Int Ed Engl. 2016 Jul 21;55(37):11144–8. doi: 10.1002/anie.201605531 (PMC5113786; doi:10.1002/anie.201605531)
Supplement: Supplementary file 1 — Supplementary [file ANIE-55-11144-s001.pdf]

Angewandte Chemie
Eine Zeitschrift der Gesellschaft Deutscher Chemiker

Supporting Information

An Epigenetics-Inspired DNA-Based Data Storage System

*Clemens Mayer+, Gordon R. McInroy+, Pierre Murat+, Pieter Van Delft, and Shankar Balasubramanian**

anie_201605531_sm_miscellaneous_information.pdf

Supporting Information

MATERIALS AND METHODS 2
PROTOCOL FOR MULTILAYER ENCODING OF DATA IN DNA 7
SUPPORTING DISCUSSION 12
SUPPLEMENTARY FIGURES 15
OLIGONUCLEOTIDE LIST 21
SUPPORTING REFERENCES 24
SUPPORTING DATA 25

MATERIALS AND METHODS

Oligonucleotides

A list of all oligonucleotide sequences used in this work can be found in **Table S3**. Biomers GmBH performed oligonucleotide synthesis and phosphoramidites for 5hmC and 5fC were prepared according to previously published procedures[1] or purchased from Glen Research (10-1062-02 and 10-1564-02).

Encoding and Decoding

A detailed step-by-step protocol for encoding and recovering of multiple layers of information in DNA can be found on page 7. Sequencing data generated during this study (BioProject PRJNA292092) are available online at ftp://ftp-trace.ncbi.nlm.nih.gov/sra/review/SRP062361_20150820_143448_4a42799b212019a2db0b77644e33790c. All custom scripts employed for encoding and decoding purposes can be found online at github.com/GRMcInroy/steg_scripts.

Text encoding

ASCII text was converted into binary and encoded into DNA using the lemonjuice script. In brief, binary messages are entered into rows of a matrix, and base pairs assigned according to the values in a given column. Positions that have identical, stacked binary values are assigned A/T (000) or T/A (111). Conversely, sites that require simultaneous encoding of different bits are assigned as C/G (011), G/C (100), 5mC/G (001), G/5mC (110), 5hmC/G (010), or G/5hmC (101). Following this matrix, canonical DNA bases suffice for two-layer encoding, while three-layer encoding requires modified cytosine analogs. As pointed out in the main text, it can be advantageous to avoid long runs of homopolymers by substituting A/T and T/A with the equivalent 5mC/G and G/5mC base pairs.

The Raven library preparation

Oligonucleotides for *The Raven* library (RAVEN-1 to -24) were combined at equimolar concentrations to create a pool at 1 µg/µL. To generate a dsDNA input for library preparation, a single primer extension (2 µg input) was performed in presence of 1 µM STEG-4, 200 µM dNTPs, 10 µL VeraSeq Buffer II and 1 U VeraSeq Ultra (both Enzymatics). Following incubation at 95 °C for 3 min and annealing at 58 °C for

45 s, extension was carried out at 72 °C for 15 min. The dsDNA was subsequently isolated and sequencing libraries were prepared with the NEBNext end repair (E6050S), dA-tailing (E6053S), and quick ligation (E6056S) modules. In order to recover the different stanzas of *The Raven*, one library was amplified directly with HiFi Uracil+ (KAPA Biosystems), while another one was bisulfite treated (CEGX TrueMethyl) before PCR amplification. Following size selection and purification by gel electrophoresis, sequencing was performed on an Illumina MiSeq with single-end 150 cycle reads.

The Raven **analysis**

Raw reads were trimmed to remove adapter sequences and low quality reads using `fastx_clipper` and `fastx_quality_filter` from the `fastx_toolkit` (http://hannonlab.cshl.edu/fastx_toolkit/index.html). Trimmed reads were split by inline barcodes (forward sequences at the 5' end and reverse complement sequences at the 3' end) employing `fastx_barcode_splitter`. To recover the information without presuming prior knowledge, the resulting reads were aligned against their corresponding barcode and a common primer binding site, using a short reference global aligner (https://github.com/dariober/bioinformatics-cafe/tree/master/eclipse/ShortRefGlobalAligner, written by Dr. Dario Beraldi). A draft reference sequence for each barcode was then generated using the script `steg_draft` and reads realigned to this reference. ASCII text was recovered from the generated alignment by running `steg_decode_text`, which assigns binary values dependent on the base composition at each position and the method from which the data was generated. The binary data is then converted to ASCII text. This script also generates a file that summarizes the reads of each library member (see **Supplementary Data**). The same analysis was performed on the model oligonucleotides encoding *BLACK-WHITE*, *BLACK-SHADE-WHITE*, and *BLACK-WHITE-COLOR*.

Image encoding

Portable bitmap format image files were converted into DNA sequences (DFT-1 to -56) using the `bakingsoda` script. In brief, the images were first compressed with run length encoding, where runs of data holding the same binary value are stored as

a count. Scanning across the horizontal axis gave the best compression factor. The obtained counts were further encoded with the universal Elias gamma code.[2] In this code the value 1 remains as such, but for a number *x*, where $x \geq 1$, define N as the highest power of 2 it contains. This value N is encoded in unary (N zeroes followed by a 1), and the remaining N binary digits of *x* are appended to the unary representation of N. To signal the end of the image, a unique stop code was appended to the Elias gamma encoded binary string. This string was then split into fragments of the desired length – 72 in the present study. If the number of fragments for each image was not integer or equal, 0s and 1s were randomly inserted to pad the strings. A list equal in length to the number of fragments was generated, with every element containing a matrix where each row contained the code from one image. Bases were then assigned depending on the bit pattern in a column, using the same assignment matrix as for text encoding. The generated 72mers were then barcoded (7 nucleotides in length) and an additional A added to the 3' end of each resulting oligonucleotide.

Portraits library preparation

Complementary pairs of DFT oligonucleotides were combined at equal concentration and 5' phosphorylated with T4 polynucleotide kinase (NEB). Subsequent incubation to 98 °C for 10 min allowed heat denaturation of the enzyme and annealing of the complementary strands in one step. Following purification by Bio-Spin 6-column (Bio-Rad), the resulting 5' phosphate, 3'-A overhang dsDNA sequences were combined at equimolar concentrations to create a pool at 1 µg/µL. Direct ligation of sequencing adapters with the NEBNext quick ligation module (E6056S) completed library preparation. To recover the portraits encoded in the template, the libraries were subjected to three different conditions. One sample was PCR amplified directly by HiFi Uracil+ (KAPA Biosystems). A second was denatured and oxidized with $KRuO_4$ before bisulfite treatment (CEGX TrueMethyl). The third sample was bisulfite treated and amplified with HiFi Uracil+ (KAPA Biosystems). Sequencing was performed on an Illumina MiSeq with single-end 150 cycle reads.

Portraits analysis

Raw reads were trimmed, had low quality reads removed, and were split by inline barcodes as described in *The Raven* analysis. As before, reads split by barcodes were aligned twice using a short reference global aligner; first against the corresponding barcode to generate a draft sequence, which was then used as the input for the re-alignment. The DNA encoded images were recovered by running the script `steg_decode_images`. In brief, binary data is recovered as described in *The Raven* analysis. This binary string is then decoded from Elias gamma to yield a sequence of decimal numbers describing the run length encoded images. Reverting the run length encoded enables plotting of the recovered image files. To successfully recover the portrait from the bisulfite treated sample, the script requires additional, positional information of any 5mC present in the sequences. This mC list is generated when analyzing the oxidation plus bisulfite data, as 5mC nucleobases are unambiguously identified during this procedure.

Reversible data encoding via a controlled redox cycle

Portraits-encoding libraries were prepared as described previously. Oxidation involved denaturation (0.2 M NaOH for 30 min at 37 °C) and treatment with $KRuO_4$ (10-fold dilution of CEGX TrueMethyl oxidant, at 40 °C for 30 min). Reduction was achieved by treatment with $NaBH_4$ (3 additions of 50 mM at room temperature over 60 min). Samples were subjected to multiple cycles of oxidation and reduction and purified between treatments with pre-washed (four times molecular biology grade H_2O) Bio-Spin P6 columns (BioRad). Following the desired number of oxidations and reductions, samples were treated with bisulfite (CEGX TrueMethyl) and PCR amplified before sequencing. Analysis was performed as described previously and sequencing summaries for the redox cycles can be found at the end of the Supporting Information. To obtain optimal coverage for each oligonucleotide reads of multiple experiments carried out under comparable conditions were combined

Four-layer information storage

Oligonucleotides were designed analogously to the three-layer encoding, except employing the alternative assignment matrix given in the Supplementary Text. Oligonucleotides DARWIN-ENCODE-DECODE-TURING-1 and -2 were annealed

(incubation at 98 °C for 10 minutes, then slowly cooled to room temperature) before library preparation was performed (NEBNext modules). In total, four libraries were generated bearing different indexes. The first sample was untreated, the second library was denatured and oxidized with $KRuO_4$ before bisulfite treatment (Cambridge Epigenetix, UK, TrueMethyl), and a third sample bisulfite treated directly. The fourth library was denatured and reduced with $NaBH_4$ (50 mM aqueous or with a reduction cocktail provided by Cambridge Epigenetix, UK) before bisulfite treatment. All samples were then amplified with HiFi Uracil+ (KAPA Biosystems), size selected and quantified by qPCR before sequencing on an Illumina MiSeq with 150 cycle single-end reads. Raw reads were trimmed to remove adapter sequences and low quality reads as described in the main methods section. Within each indexed library, reads were aligned using a short reference global aligner, and a draft sequence generated. Reads were realigned against this draft sequence and the base calls at each position quantified. Depending on the base composition at each position, a binary value was assigned and thus a binary string generated. The binary data was then converted to its ASCII representation. To recover the fourth message *TURING*, a 0 to 1 *in silico* transposition cipher was applied at 5mC positions determined from the oxidative bisulfite sequencing data.

Liquid chromatography–mass spectrometry (LC-MS)

Analyses were performed on a Bruker amaZon system, with an XTerra MS C18 column (2.5 µm, 2.1 x 50 mm). Solvents were A (10 mM TEA, 100 mM HFIP) and B (MeOH), with a gradient from 5% B to 30% B over 25 minutes.

PROTOCOL FOR MULTILAYER ENCODING OF DATA IN DNA

In this section we provide a description of the protocol used for the multilayer encoding of data in DNA. As an example we give a step-by-step protocol for the simultaneous encoding and reading of the ASCII representations of the words *BLACK*, *WHITE* and *COLOR* in a single oligonucleotide (results and sequencing data are reported in Fig. 3D). All experiments described in the manuscript use a similar approach.

1. In this example, three layers of information are encoded. The words *BLACK*, *WHITE* and *COLOR* are used as input.

```
BLACK
WHITE
COLOR
```

2. The three layers of information are represented as binary strings using the ASCII character-encoding scheme (eight bits per characters) and aligned at their start position.

```
BLACK: 0100001001001100010000010100001101001011
WHITE: 0101011101001000010010010101010001000101
COLOR: 0100001101001111010011000100111101010010
```

   The information may be compressed via any standard algorithm prior to this step. If the binary strings are not of equal length, binary digits can be added individually in order to pad the strings. These digits may be inserted randomly, or with constraints in place to minimize the number of modified bases.

3. At each position in the bit string, the binary pattern vertically across all three messages is read. Here, the first four readings are ‘000’, ‘111’, ‘000’, and ‘010’.

4. The cross-layer readings are converted to a DNA base pair following an assignment matrix (given below). In this example, the first four positions become ‘AT’, ‘TA’, ‘AT’, and ‘6G’. The non-canonical bases 5mC and 5hmC are indicated as 5 and 6.

AT	TA	CG	GC	5G	G5	6G	G6
0	1	0	1	0	1	0	1
0	1	1	0	0	1	1	0
0	1	1	0	1	0	0	1

5. According to the assignment matrix, the text BLACK, WHITE and COLOR are encoded in the following DNA sequence string:

```
5′ ATA6A6TCATAATG55ATAAC5AGATA65CGGATA5G6GG 3′
3′ TATGTGAGTATTA6GGTATTGGT5TATGGG66TATGCG65 5′
```

6. At this stage, long oligonucleotide sequences are split into manageable pieces of equal length (e.g. 72 nucleotides for the portraits). For purely illustrative purposes, the DNA sequence obtained from step 4 is split into two segments of length 20.

```
Oligo 1:    5′ ATA6A6TCATAATG55ATAA 3′
Oligo 2:    3′ TATGTGAGTATTA6GGTATT 5′
Oligo 3:    5′ C5AGATA65CGGATA5G6GG 3′
Oligo 4:    3′ GGT5TATGGG66TATGCG65 5′
```

7. A unique barcode sequence (containing A, T, G and 5mC) is appended to each oligonucleotide, at the 5' terminus if numbered odd, or the 3' end if numbered even. The minimum barcode length is determined by the library size, with larger libraries requiring longer barcodes.

```
Oligo 1:    5′ A5TT5GGATA6A6TCATAATG55ATAA 3′
Oligo 2:    3′ TGAAG55TATGTGAGTATTA6GGTATT 5′

Oligo 3:    5′ GA5AG5AC5AGATA65CGGATA5G6GG 3′
Oligo 4:    3′ 5TGT5GTGGT5TATGGG66TATGCG65 5′
```

8. A 3'-A can be appended to every oligonucleotide to simplify library preparation. Similarly, a 5' phosphate can be introduced. The resulting list of oligonucleotides is then synthesized.

```
Oligo 1:    5′ A5TT5GGATA6A6TCATAATG55ATAAA 3′
Oligo 2:    5′ TTATGG6ATTATGAGTGTAT55GAAGTA 3′
Oligo 3:    5′ GA5AG5AC5AGATA65CGGATA5G6GGA 3′
Oligo 4:    5′ 56GCGTAT66GGGTAT5TGGTG5TGT5A 3′
```

9. Once synthesized, oligonucleotides may be stored for long periods of time. Grass et al. (ref 10 in the manuscript) have previously described suitable conditions for long-term storage of DNA.

10. The stored information can be read by a number of different sequencing methods. In the present study, we use Illumina sequencing, which requires library preparation before reading the information; other sequencing methodologies may have different or no such requirements. Retrieval of each information layer is possible by employing standard sequencing (BLACK = first layer), oxidative bisuflite sequencing (WHITE = second layer) and bisulfite sequencing (COLOR = third layer). The expected sequence of Oligo 1 for each readout method is depicted below.

```
Oligo 1 seq:     5′ ACTTCGGATACACTCATAATGCCATAAA 3′
Oligo 1 oxBS:    5′ ACTTCGGATATATTTATAATGCCATAAA 3′
Oligo 1 BS:      5′ ACTTCGGATACACTTATAATGCCATAAA 3′
```

11. Raw sequencing data are filtered to remove reads shorter than the oligonucleotides comprising the library (20 in this example) and adapter dimers. If the sequencing instrument assigns data quality scores, for example a Q score, low quality reads may also be discarded.

12. Reads are separated by barcode, scanning for a 5’-3’ barcode at the 5’ terminus (beginning of line) and the reverse complement at the 3’ terminus (end of line).

13. Members within each barcode are analyzed with a short read global alignment program, to generate a reference sequence containing quantitative information of each base at every position.

14. Depending on the fraction of each nucleobase at a given position, binary values are assigned. As example, data generated from oxidative bisulfite sequencing is shown below (A: blue; C: yellow; G: white; T: red). In position one the data is

almost exclusively A, and so is assigned a 0. In position eight the data is 0% A, 50% C, 0% G, and 50% T, therefore 1 is assigned.

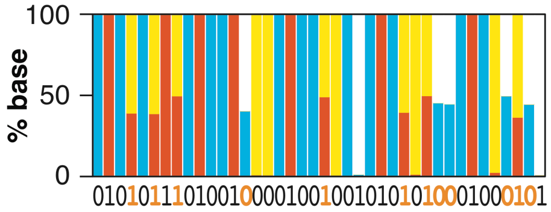

15. A binary string is generated that can then be transformed into the original data. In this example, oxidative bisulfite sequencing will produce the following string:

0101011101001000010010010101010001000101

16. A simple binary to ASCII conversion generates the text WHITE, recovering the second layer of information.

WHITE

Note

(i) For the three-layer encoding of BLACK-WHITE-SHADE using only 5mC as an extra nucleobases (**Supplementary Figure 2**), we have used the same assignment matrix as described in step 4. Because 5hmC is absent in the designed DNA template, this approach does not allow the encoding of all bit permutations and hence, can be used to encode only a limited combination of characters.

(ii) For the four-layer encoding of DARWIN-ENCODE-DECODE-TURING (**Supplementary Figure 4**), we have used 5fC as an independently variable nucleobase and the following assignment matrix:

	**AT**	**TA**	**CG**	**GC**	**5G**	**G5**	**6G**	**G6**	**7G**	**G7**
**seq**	0	1	0	1	0	1	0	1	0	1
**oxBS**	0	1	1	0	0	1	1	0	1	0
**BS**	0	1	1	0	0	1	0	1	1	0
**redBS**	0	1	1	0	*1*	*0*	0	1	0	1

The non-canonical bases 5mC, 5hmC and 5fC are indicated as 5, 6 and 7. Positions highlighted in blue indicate an *in silico* transposition cipher at unambiguously identified 5mC sites.

The following binary combinations cannot be encoded with the current approach:

**seq**	0	1	1	0	0	1
**oxBS**	0	1	0	1	0	1
**BS**	1	0	1	0	1	0
**redBS**	0	0	0	1	1	1

SUPPORTING DISCUSSION

Scalability and robustness of multilayer DNA data storage

Herein, we assess the scalability of our approaches discussed in the main text in greater detail. The following sections independently address library quality, robustness in sequence context, and general scalability aspects for the three reading methods. For the analyses, we used data generated from *The Raven* and portraits-encoding libraries of a total of 5 experiments. These are the first stanza of the poem and the picture of Charles Darwin for standard sequencing, the second stanza and the portrait of Alan Turing for bisulfite sequencing, and the picture of Rosalind Franklin for the oxidation and bisulfite (oxBS) workflow.

Library quality: Before decoding a message with the desired readout method, we processed raw sequencing data to discard unsuitable reads. Such filtering reduces the effective data storage capacity of DNA libraries and must be considered when assessing scalability of our approach.

For the first readout, standard sequencing, we retained 41.02% and 81.67% of raw reads for *The Raven* and portraits-encoding libraries (**Fig. S4A**, dark and light blue pie charts). In both experiments, reads that are shorter than the expected coding sequences account for the majority of reads lost during processing. The two-fold increase of unsuitable reads for *The Raven* library can be rationalized when considering the length of oligonucleotides in both libraries (139 for the Raven compared to 80 for the portraits). During synthesis the prevalence of (n-1), (n-2), … species increases with length, and thus purification becomes more challenging. Such length dependency has previously been observed, with read retention of 63% for 117mers and 47.62% for 159mers.[3]

As expected, results for the second readout, bisulfite sequencing, followed the same trend (**Fig. S4A**, dark and light green pie charts). Again, significantly fewer reads were retained for *The Raven* library (30.66%) than for the portraits-encoding oligonucleotides (81.44%). Moreover, for the former we observe a further increase in reads deemed too short, when compared to the analogous standard sequencing data set. This increase could be a result of bisulfite-induced strand scissions, a

known phenomenon whose probability increases with oligonucleotides length. As a result, full-length fragments may be depleted from the library pool relative to shorter by-products from synthesis or library preparation (*e.g.* adapter dimers). Accordingly, for the shorter oligonucleotides in the portraits-encoding library, we retained the same portion of initial reads as in the standard sequencing experiment.

The third reading method, the oxBS workflow, relies on an additional chemical step. This additional transformation appears to have a minor impact on library quality, as the percentage retained reads decreased by 25% compared to the other two readouts (**Fig. S4A**, red pie chart). We suggest that the observed loss might either reflect an adverse effect of residual oxidant on the polymerase during amplification or an increased probability for strand scission during the bisulfite treatment.

Overall, our results demonstrate that neither oxidation nor bisulfite treatment drastically decrease library quality, which supports the scalability of our approach.

Sequence context: Although **Table S1** demonstrates robustness of all readout methods, we will discuss the potential influence of sequence context on the reading properties of cytosine species in this section. For this purpose, **Fig. S4B-D** depicts base calls (**Fig. S4B** for standard sequencing) and conversion efficiencies (**Fig. S4C-D** for bisulfite sequencing and the oxBS workflow respectively) of C, 5mC and 5hmC in the context of their adjacent nucleotides. From these plots it becomes evident that a potential effect of neighboring positions is negligible. All cytosine species are read >99% as C in standard sequencing. Canonical cytosine positions convert consistently and with good efficiencies in the bisulfite sequencing readout, while the modified bases remain unaltered. Lastly, in the oxBS workflow, C and 5hmC species behave as expected regardless of their sequence context while 5mC does not convert. In total, we monitored the behavior of 5681 positions and did not observe outliers. Consequently, our results unambiguously demonstrate the robustness of all three readout methods.

Scalability: The number of reads required to reliably retrieve the information encoded in a given template is crucial for assessing the scalability of our readout

methods. Having initially obtained high coverage across all our experiments, we randomly subsampled sequencing reads to mimic a data set that would be obtained for larger, more diverse oligonucleotide libraries. Subsampling was performed one hundred times with replacement, thus corresponding to the retrieval of 24.5 – 33.6 kB of information in our five experiments. **Fig. S4E** reports on the percentage of correctly decoded information for a given sample size and Fig. S4 depicts the corresponding error rate over all hundred iterations.

When randomly selecting only a single read per strand, message recovery was possible at >96% for all reading methods (**Fig. S4E**). For standard sequencing, the information was recovered error-free, when four strand pairs were sampled for the portrait of Charles Darwin and six for the first stanza of *The Raven*. Bisulfite sequencing selectively switched encoded bits at cytosine positions and eight read pairs sufficed to recover the picture of Alan Turing without error over one hundred iterations. For the second stanza of the poem and the information decoded by the oxBS workflow, 10-fold coverage was sufficient to retrieve the information at an error rate < 1 in 10^5 nucleotides or one bit over 100 iterations, while 15-fold coverage of read pairs suffices to recover the information error-free in all our experiments (**Fig. S4F**). Therefore, the three images totaling 5,955 bits can be reliably retrieved from 2520 reads (15x56x3). An Illumina Nextseq500 run produces 400 million reads at a cost of £1,700, enabling the reading of up to 118 MB of data for £14.4 per megabyte.

SUPPLEMENTARY FIGURES

A

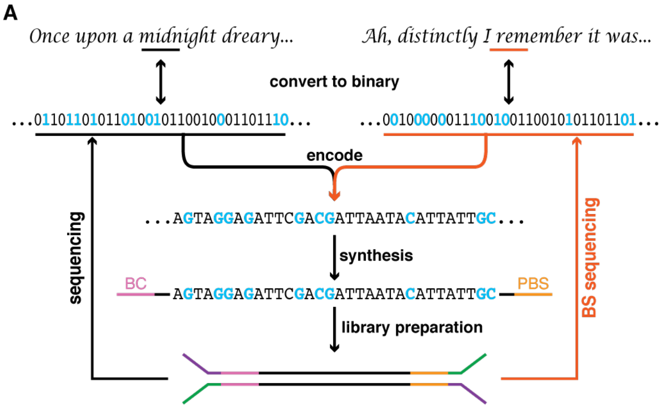

B

base pair	N	mean [%]	σ [%]
A/T	971	99.90	0.16
T/A	789	99.95	0.09
C/G	440	99.86	0.10
G/C	420	99.82	0.16
5mC/G	68	99.50	0.24

C

base pair	N	mean [%]	σ [%]
A/T	971	99.79	0.18
T/A	789	99.84	0.12
C/G	440	50.37	0.16
G/C	420	49.72	0.19
5mC/G	68	96.97	2.09

Figure S1: Two-layer encoding and reading of the first two stanzas of Edgar A. Poe's *The Raven*. A) Workflow for encoding and reading of the two first stanzas of Edgar A. Poe's *The Raven*. ASCII Text is converted into binary and translated into a nucleic acid sequence following the matrix depicted in the "Protocol for multilayer encoding of data in DNA" section. Oligonucleotides are synthesized harboring a barcode (BC) and a common primer binding site (PBS). Then, libraries are prepared for next generation sequencing (see Methods for details), and stanzas 1 and 2 are recovered from traditional sequencing and bisulfite sequencing. Results for sequencing **B)** and BS-sequencing (**C**) of the poem-encoding oligonucleotide library. Results are presented as means and standard deviations of correct reads in percent over all positions (N) in the library for a given base pair. Note: In panel **C,** 50% correct reads for C/G and G/C base pairs indicates full conversion in the bisulfite treatment.

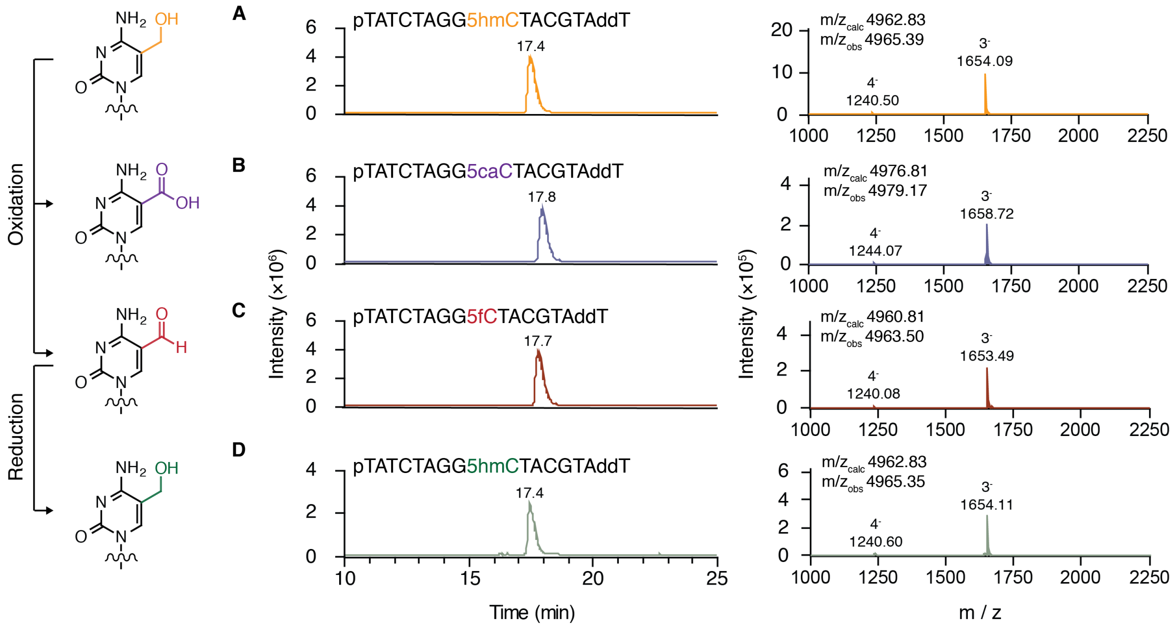

Figure S2: LC/MS analysis for the selective oxidation of 5hmC to 5fC in a model oligonucleotide. A) Base peak chromatogram and corresponding mass data obtained for a 5hmC containing oligonucleotide (STEG5). The oligonucleotide is further modified with a 5' phosphate and a 3' dideoxy-T to prevent oxidation of the 5'- and 3'-hydroxyl groups. **B)** $KRuO_4$ (Cambridge Epigenetix, UK, TrueMethyl solution) oxidation for 30 minutes at 40 °C leads to complete oxidation to 5caC. **C)** A 10-fold dilution of the $KRuO_4$ oxidation solution and a decrease of the incubation temperature to 0 °C, enables a selective oxidation to 5fC. **D)** The oligonucleotide oxidized with the conditions from **C** was subsequently reduced with $NaBH_4$ (50 mM) for 60 min in the dark, regenerating the original 5hmC.

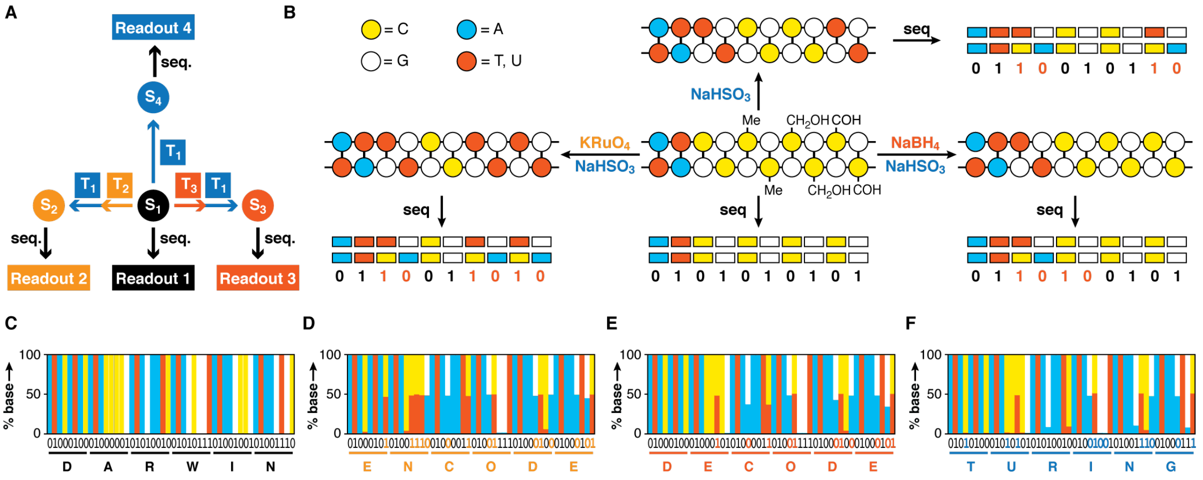

Figure S3: Proof-of-concept of four-layer encoding using 5fC as an independently variable nucleotide. A) Addition of a further modified base, 5fC, enables encoding of four messages in a single DNA template due to the ability to access a fourth state from the original input. **B)** The principle of four-layer encoding with the inclusion of 5fC. Employing the same bit switching strategy as in previous example, oxidative or reductive conditions prior to bisulfite treatment affect the cytosine analogs' susceptibility to conversion, and thus the encoded message. (**C–F**) Four-layer encoding proof-of-concept. Shown is a 48 base pair region of an oligonucleotide, which encodes binary for the ASCII text *DARWIN* (**C**), *ENCODE* (**D**), *DECODE* (**E**), and *TURING* (**F**) following standard, oxBS, traditional bisulfite, and reductive bisulfite sequencing respectively.

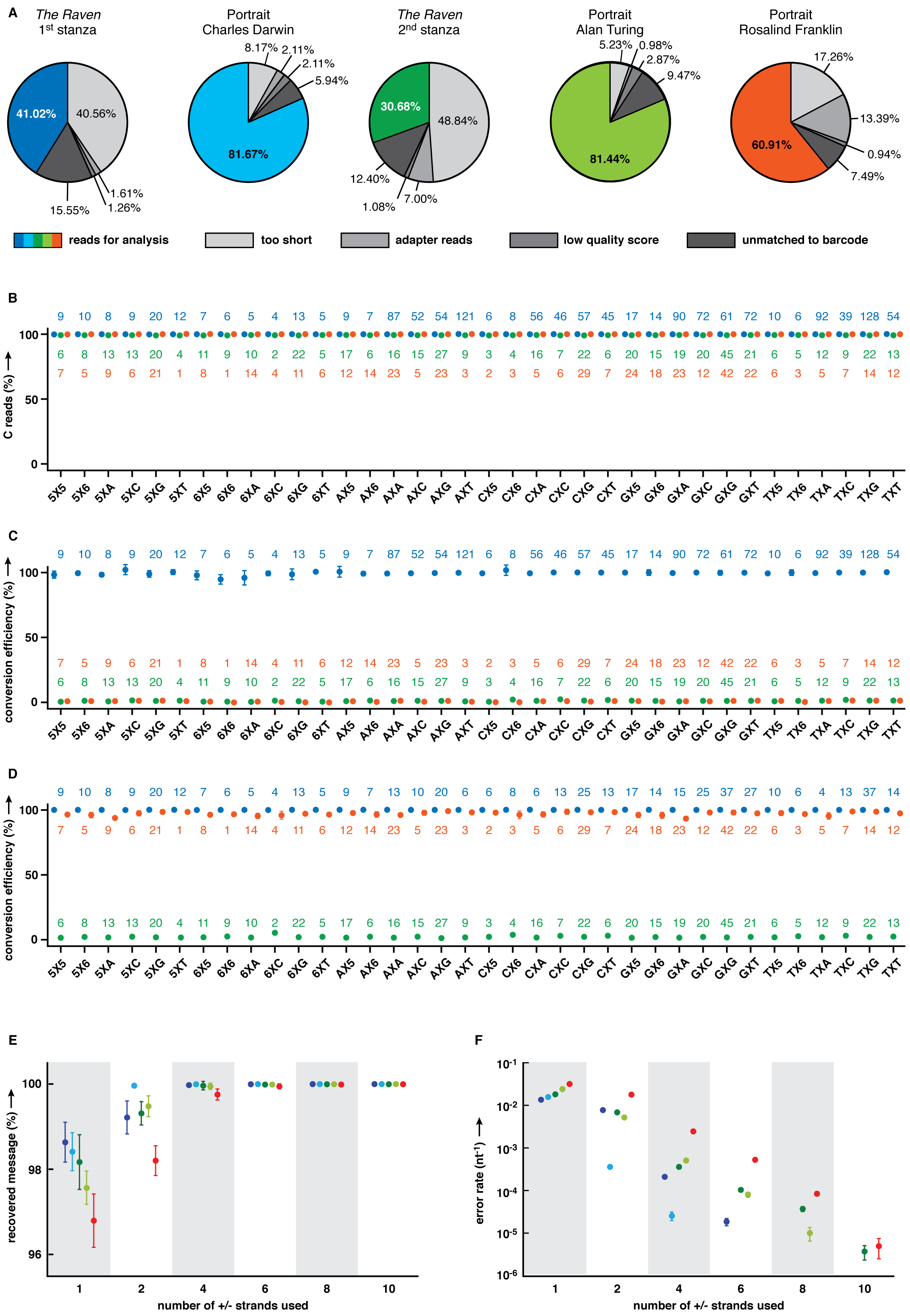

Figure S4: A) Library quality obtained for the different sequencing experiments in the manuscript. Influence of neighboring nucleotides on the behavior of C (blue), 5mC (green) and 5hmC (red) in standard sequencing **B)**, bisulfite sequencing **C)** and the oxBS workflow (**d**). Numbers associated with data points denote occurrence of the sequence in the original DNA templates. Percentage of correctly decoded information **E)** and error rate **F)** with respect to the number of reads used in the analysis. Error rates < 1 in 10^6 not shown. Refer to the **Supporting Discussion** for details.

base pair	N	Readout 1		Readout 2		Readout 3	
		mean [%]	σ [%]	mean [%]	σ [%]	mean [%]	σ [%]
**A**/T	534	99.90	0.10	99.61	0.61	99.84	0.17
**T**/A	102	99.87	0.23	99.65	0.55	99.78	0.28
**C**/G	163	99.81	0.07	49.95	0.21	50.67	1.17
**G**/C	316	99.81	0.09	49.92	0.23	49.85	0.99
**5mC**/G	323	99.47	0.17	98.57	0.41	98.95	0.39
**G**/5mC	165	99.43	0.19	98.26	0.43	98.69	0.38
**5hmC**/G	271	99.83	0.06	52.04	1.14	99.39	0.39
**G**/5hmC	142	99.82	0.06	51.64	0.80	99.27	0.29

Table S1: Sequencing results for the picture-encoding DNA library (Fig. 3). Results are presented as means and standard deviations of correct reads in percent over all positions (N) in the library for a given base pair. Note, 50% correct reads for C/G and G/C base pairs indicates full conversion for Readouts 2 and 3, and 50% correct reads for hmC/G and G/hmC base pairs indicates full conversion for Readout 2.

step	C/G & G/C			5mC/G & G/5mC			5hmC/G & G/5hmC			A/T & T/A		
	errors / N	mean [%]	σ [%]	errors / N	mean [%]	σ [%]	errors / N	mean [%]	σ [%]	errors / N	mean [%]	σ [%]
**Start**	0 / 479	50.13	1.04	0 / 488	98.77	0.39	0 / 413	99.27	0.35	0 / 636	99.81	0.19
**Ox1**	0 / 479	49.75	0.41	0 / 488	98.08	0.47	0 / 413	56.00	3.16	0 / 636	99.70	0.30
**Red1**	0 / 479	49.61	1.36	0 / 488	98.78	0.50	0 / 413	97.00	1.51	0 / 636	99.78	0.28
**Ox2**	0 / 479	4978	0.41	0 / 488	98.13	0.54	0 / 413	55.97	2.85	0 / 636	99.69	0.32
**Red2**	0 / 479	49.57	2.30	0 / 488	98.37	0.82	0 / 413	83.95	4.11	0 / 636	99.71	0.48
**Ox3**	0 / 479	49.87	0.97	0 / 488	98.07	0.90	0 / 413	57.15	3.61	0 / 636	99.55	0.75
**Red3**	0 / 479	50.34	2.24	0 / 488	98.48	0.89	0 / 413	77.88	5.74	0 / 636	99.71	0.57
**Ox3**	0 / 479	49.83	0.80	0 / 488	97.92	1.20	0 / 413	55.98	4.50	0 / 636	99.38	1.04
**Red3**	8 / 479	49.08	3.68	0 / 488	98.13	1.54	0 / 413	74.92	5.69	0 / 636	99.60	0.76
**Ox3**	0 / 479	49.69	0.62	0 / 488	97.85	1.53	0 / 413	54.04	2.74	0 / 636	99.42	0.91
**Red3**	12 / 479	49.63	4.33	0 / 488	98.13	2.05	20 / 413	73.04	9.77	0 / 636	99.67	0.87

Table S2 I Sequencing results and error rates for all bases throughout redox cycles mediated by $KRuO_4$ and $NaBH_4$ (Fig. 4D). Cytosines convert efficiently, and A's, T's, as well as 5mC's are retained, in all treatments. The 5hmC positions are either converted (following oxidation) or retained (when reduced) during the cycles. Efficiency of 5hmC manipulation decreases over time, likely due to the over-oxidation of 5hmC to 5caC, which cannot be reduced with $NaBH_4$. Bits incorrectly assigned during the analyses of the experiments are presented as errors / total number of bases (N). For the reduction the treshhold for assigning bit switches was set to: %base C - %base T > 20% and %base G - %base A > 20%, respectively.

OLIGONUCLEOTIDE LIST

ODN	Sequence (5' to 3'), 5 = 5mC, 6 = 5hmC, 7 = 5fC	length
STEG-1	CTA ATA CCA CTC ACT ATA CCC	21
STEG-2	CTA CCT ACT TAT TCC TCA CCC C	22
STEG-3	5TA ATA 55A 5T5 A5T ATA 555	18
STEG-4	55T A5T TAT T55 T5A 555 5	19
STEG-5	pTAT CTA GG6 TAC GTA ddT	16
BLACK-WHITE-1	CTA ATA CCA CTC ACT ATA CCC AGC TGA CTC AGT TAG CAG CTG ACT CAG TTA<br>GCA TAC ACT C	61
BLACK-WHITE-2	CTA CCT ACT TAT TCC TCA CCC CAC GCT TAT CCG TGT ATA TTG TTA TTT CAT<br>TAT GAG TGT ATG CTA ACT GAG TCA GC	77
BLACK-WHITE-SHADE	5TA ATA 55A 5T5 A5T ATA 555 AGC TGA CTC AGT TAG CAG CTG ACT CAG TTA<br>GCA TAC A5T CAT AAT GAA ATA A5A ATA TA5 ACG GAT AAG CGT GGG GTG AGG<br>AAT AAG TAG GTA G	115
BLACK-WHITE-COLOR-1	5TA ATA 55A 5T5 A5T ATA 555 AGC TGA CTC AGT TAG CAG CTG ACT CAG TTA<br>GCA TA6 A6T CAT AAT G55 ATA AC5 AGA TA6 5CG GAT A5G 6GG GGG GTG AGG<br>AAT AAG TAG G	112
BLACK-WHITE-COLOR-2	55T A5T TAT T55 T5A 555 556 GCG TAT 66G GGT AT5 TGG TTA TGG 6AT TAT<br>GAG TGT ATG CTA ACT GAG TCA GCT GCT AAC TGA GTC AGC TGG GTA TAG TGA<br>GTG GTA TTA G	112
DARWIN-ENCODE-DECODE-TURING-1	55T A5T TAT T55 T5A 555 5G7 ACT TAT CGG 7TT ATA 55G CTA TGA TT7 TAT<br>6GG GGT ATG TAT GTA TAG TCA GCT GCT AAC TGA GTC AGC TGG GTA TAG TGA<br>GTG GTA TTA G	112
DARWIN-ENCODE-DECODE-TURING-2	5TA ATA 55A 5T5 A5T ATA 555 AGC TGA CTC AGT TAG CAG CTG ACT ATA 5AT<br>A6A TA5 6C6 GAT AGA AT7 ATA GCG GTA TAA GC5 GAT AAG TGC GGG GTG AGG<br>AAT AAG TAG G	112
RAVEN-1	A5A 5A5 ATA TA5 GGG TAT TAT GG5 AGT ACC GGA GTA AG5 GAC TAA CAA ATT<br>GCG ATA TTT AAC CAT TCG TGG ATT ATG GCA CTA CCC AAT TAA ACT ACT CAC<br>AAA TTA TTA GAT TCT AAT GGG GTG AGG AAT AAG TAG G	139
RAVEN-2	A5A 5A5 T5A GTA AG5 AAT GAT GGC AGT AG5 AGA TTC AGT GAT TAG CAC ATT<br>GCT ACA CTA ACA CAT TAC TAC ATT GAA TAA TTA ATA TAT TCA ACG AGT GA5<br>GAA TTG TAA TAC TCG TAA GGG GTG AGG AAT AAG TAG G	139
RAVEN-3	A5A 5A5 TTA ATA A5A AAT TTA TTT ATT AGA ACA TTC GAC TAG TAG G5A ATT<br>ACG ATA CTA CCC AAG CAG 5AG ACT CAC AAA TTG CAA AAT TAG TGT AGT AGG<br>G5A TTA AGC AAT TAC TAG GGG GTG AGG AAT AAG TAG G	139
RAVEN-4	A5A 5AT A5A TTG ACG CAT TAA GAT ATT ACG CCA ATA GG5 AAC GAA CAA ATT<br>GAT GTA TTA AGC TAT TAA CAT ATT ATC GTA CTA AAC AAT TAA CAT ATT CGG<br>TAA GTC CGC CAA GAC ACA GGG GTG AGG AAT AAG TAG G	139
RAVEN-5	A5A 5AT ATA TGG 5GG TAT TAC TCG ATT AAC AGA GTG A5G AAT TGG CAT ACT<br>AGG ACA CCA GAT CAT CAT GGG 5GT GAG G5A TTC AGC TAT TGA CGC ACT CAA<br>5AA TTA GGA TAT TCA ACG GGG GTG AGG AAT AAG TAG G	139
RAVEN-6	A5A 5AT T5A TTA GGG CAT TTG CAG ACT AAC ACA GTA A5A GAC TAA CAA ATT<br>TCA ATA TTG CGA TAT TAC CCG ATT AGC CTA GTA GGG 5AT TGA TAC ACT ACC<br>ACA TTA AAC GAT TAG TGC GGG GTG AGG AAT AAG TAG G	139
RAVEN-7	A5A 5AT TTA TTC AGC AAA TAA 5AA ATT CAC TTA TTT AGC GAT TGC CTC ATT<br>CGC ATA TTA GTT TAT TGC GAG ATT TAC GGA ATA A5A AAT TGC GGC ATT CGT<br>GGA TTC GGC CAG TG5 GAG GGG GTG AGG AAT AAG TAG G	139
RAVEN-8	A5A 5T5 A5A TTA GTC TAT TAC GAG ACT ACC CCA TTC GGT TAT TCA TGA AAT<br>AA5 AAA TTC ATG CAT TCG GGG 5TT GCC TCA TTA CTT GAG T5G GGG 5TT TAT<br>AAA TTG CGA AAT TAA TAT GGG GTG AGG AAT AAG TAG G	139
RAVEN-9	A5A 5T5 ATA GTA GGG 5AC TAA CCA ATT ATT AAA TTA TTT TAT TGC CTC ATT<br>CAG CGA ATA CCC AA5 GAT GCG ACA AGC GCA TCG AGG TAT TAG CCC ATT AGC<br>ATA TTC GGC AAT TAC TAG GGG GTG AGG AAT AAG TAG G	139
RAVEN-10	A5A 5T5 T5A CTC CAA CAG CAG A5G ACG ACA ACA GTA GGG 5AT TCG TTT ATT<br>ACG ACA TTC AGC CAT TAC GAG ATT AAT ACA CTA GTA AAA TAA A5A ATT CGT<br>GAA TTA CGA GAT TAA CAT GGG GTG AGG AAT AAG TAG G	139
RAVEN-11	A5A 5TT A5A GTG A5G AAT TAT TAC ATT GTC CTA CTC AAC AAT TCG GTA ATT<br>ACC CTA TTT ACC CAG TTC ACC AGT AGA 5GA GTA TTG CAG TA5 GGG ACT CGT<br>CAA CTA AAA CAT TGC AGT GGG GTG AGG AAT AAG TAG G	139
RAVEN-12	A5A 5T5 TTA TTG CTC GAT TAC TAA ATT CCG ACA GTA AG5 GAT GAT GGC AGT<br>AGG 5AA TTG TAA GAC TAA AAC ATT GAT AAA GTA GA5 AAT TCA GCT ATT GCC<br>TCA TTC ATA TAC TAA CCC GGG GTG AGG AAT AAG TAG G	139
RAVEN-13	A5A 5TT ATA TTA CAG GAT TCA CAG AGT AGG 5GA TTC ATA GAC TAC CCC AGT<br>AA5 AGA CTA AAC AAT TGC TCC ATT CAA CGA TTT AAC AAT TGC CCC ATT CGC<br>CTA GGA TGT AAT GAA TTG GGG GTG AGG AAT AAG TAG G	139

RAVEN-14	A5A 5TT T5A CTC GGC AAC CAT CTC ATC ACC ATA GTG A5G GAC TAC CAC ATT<br>CTG GTA GTA 5GG AAC TAA ACA ATT GCC TTA TTA TTT TAT TAT GCT ATT CAG<br>CTA ATA AA5 AAT TCG GTT GGG GTG AGG AAT AAG TAG G	139
RAVEN-15	A5A 5TT TTA TTC GTG CAT TCA GCG ACT AAA CCA TTA ATG TAT TAA GAT ATT<br>CGG TCA TTG ATA CAG TAG G5A ATT GTC CTA CTA ACC AAG TGA 5GA ATT CAA<br>CTA TTG CCC CAT TTA ACA GGG GTG AGG AAT AAG TAG G	139
RAVEN-16	A5A TA5 A5A TTC GAC GAT TAT TTC ATT CAT TTA ATA GG5 AAA TAC CAC AGT<br>GA5 GAA TTC AAC TAT TGC CCC ATT TAA CAA TTC GAC GAT TAT TTC ATT CAT<br>TTA ATA A5A AAT TAA CCG GGG GTG AGG AAT AAG TAG G	139
RAVEN-17	A5A TA5 ATA TTG CTC CAC TCA ACA AGT AGG 5GA TTT GCA GAC TAC AAA ATT<br>AAC GTA GTA G5A AAT TAC CAG ATT ATT CTA TTC AAT CAT TCA TAG AGT GA5<br>GAA CGA CCA AAT TAA TAC GGG GTG AGG AAT AAG TAG G	139
RAVEN-18	A5A TA5 T5A TTA TTT GAT TAT TTT ATT TAA TAA CTA GTG CAA CAG AG5 AAT<br>ACC GCA AGA C5C AAC GAA TTG ATC GCT CCA TTC GAC GAG TGA 5GG ACT CAC<br>AAA TTG CAG GAT TAG TGT GGG GTG AGG AAT AAG TAG G	139
RAVEN-19	A5A TAT A5A GTA GG5 GAT TCA GCG ACT AAA ACA TTT AGT AAT TAG CAT AGT<br>GA5 GGA TTA GAA TAT TGC TCA ATT AAT AGA GTG A5G AAC TCG GTA ACT AAA<br>GCA CTA ACA AAT CAT AAT GGG GTG AGG AAT AAG TAG G	139
RAVEN-20	A5A TT5 A5A CTA AAA CAT TAT TCG ATT TAT AGA GTG AG5 AAT TGC TAC ATT<br>AAG ATA TTG CAG CAT TAA TAG ATT AAT ACA CTA TTC AAA TAA A5A ACT CAC<br>TCA TTG CGA AAT TAC CCT GGG GTG AGG AAT AAG TAG G	139
RAVEN-21	A5A TA5 TTA TTG CCA CAG TGA A5A ATT CGC AGA TTA TGG AAT TAA TGT AAT<br>AA5 AAA TTA AAA TAT TGC TCA ACT AAC CCA TTA GTA TAT TGT CAG ACT CAA<br>CCA GTA A5G GAT TAT CCA GGG GTG AGG AAT AAG TAG G	139
RAVEN-22	A5A TAT ATA TTA AAA TAT TAT TAT ATT AAC GCA GTA AG5 GAT GGC CGA ACT<br>AAC ACA TTA CTC AAT TAT TTT ATT CGG TGA TTG ACG CAA TAA A5A AAT ATT<br>ATA AAA T5T AAT AAT TTG GGG GTG AGG AAT AAG TAG G	139
RAVEN-23	A5A TT5 ATA TTA GGG CAT TAT TAC ATT GGC ATA CTA CCA AAT TGA TAC ATT<br>CGA CCA TTC GAC TAG TGA 5GG ACT ACA AAA TTA ACA TAT TCG GTA ATT AAT<br>ACA ATA A5A AAT TAG TTA GGG GTG AGG AAT AAG TAG G	139
RAVEN-24	A5A TAT T5A TTA TTT TAT TTA GCA AGT AGA 5AA TTA GCA TAT TCG TTA ATT<br>AAT GTA CTC AAC AAT TAT TAT ATT ATT TTA TTT AAT AAT TAA TAT AAT ATT<br>TAA ATA A5G AAA TAA 5AA GGG GTG AGG AAT AAG TAG G	139
DFT-1	AAA 5AA 5AA AA5 CA5 AG6 65G GGG AG6 G55 AA5 CGG GCA GCG GA6 GGG GA5<br>5AA GTA A6A 6AG GC5 G5G GAG A56 6AA GA	80
DFT-2	CTT GGG TCT C5G CGG CCT GTG TTA 5TT GGT 566 CGT CCG 5TG 5C5 GGT TGG<br>CGC TC5 6CG GG5 TGT GGT TTT GTT GTT TA	80
DFT-3	AAA G5T 56G G5G G5G A6A GGG G66 GAG AC5 ACG CGA GG5 GGG AAC TG5 GC6<br>6C5 GAA GGG G5G G65 AAT 55G 6AA A55 GA	80
DFT-4	CGG TTT GCG GAT TGG 65G 655 CTT 6GG GGG CG5 AGT T55 CG6 6T5 G5G TGG<br>T5T CGG CC6 CTG TCG 56G C5G GAG 5TT TA	80
DFT-5	AA5 AAG TC6 GG6 GCT AAA 6AG 56G GGG ACC AAG 66A G56 55C GG5 A5G 6GA<br>A55 65G 56A 56C AAA GGT CA5 5C6 AGA CA	80
DFT-6	GT6 TGG GGT GAC CTT TGG GTG G6G GGG TT5 GCG TGC 5GG GGG 6TG GCT TGG<br>TC6 C6G G6T GTT TAG 6G5 6GG A5T TGT TA	80
DFT-7	AAG T5A TAA 66A 55G GT5 66G 6G6 6AA GT5 TG6 5A5 GGG GGG T55 A6G G5T<br>GA6 6A5 G5A 65G C5A AGA GGG TAG T65 CA	80
DFT-8	GGG ACT A66 5TC TTG G5G GTG 6GT GGT 5AG C6G TGG AC5 5CC 6GT GG5 AGA<br>CTT GGC G6G GGA 6CG GTG GTT ATG A5T TA	80
DFT-9	A5A TAT GAA GAA 65T 565 AG6 GTG GAG AC5 CAA GAA CGC A66 G5C G5G A5C<br>6GG AC5 G5G A56 ACG A5A G66 A5G 66G 6A	80
DFT-10	G5G G6G TGG CTG T6G TGG TCG CGG T5C GGG TCG CGG CGG TGC GTT CTT GGG<br>T5T CCA 6G6 TGG GAG GTT CTT 5AT ATG TA	80
DFT-11	A5G GGT AAG 656 6AG 5G5 C5A C6G A5G CTA AAG C6G 56G AG5 AAT A6G A5A<br>A5G 5AG AGA CAG A6G A5G G65 6AG 5AC CA	80
DFT-12	GGT G5T GGG 6CG TCG T6T GT6 TCT G5G TTG TCG TAT TGC TCG G6G GCT TTA<br>G6G T5G GTG GGC GCT GGG G6T TA5 55G TA	80
DFT-13	A5T T5G GAA ACT 5AG CGA AAG 56G 65G GGA ATG GC5 AAA GC5 G6G 65A GC6<br>G5A 6GC 5CA AGG A6A 65A G5A GA6 A5A GA	80

DFT-14	CTG TGT 5TG 5TG GTG TC6 TTG GGC GTG 6GG 5TG GCG 5GG 6TT TGG 55A TTC C5G GCG G6T TTC GCT GAG TTT 55G AAG TA	80
DFT-15	AGA GTT GAG 6AC 6GA 5TA 6GA GAA 6CA T66 GA6 5AA G5G A66 5GC GAA GGA 5A5 CG6 5GA GA5 5G5 GA6 ACG CGG 6GG AA	80
DFT-16	TC5 GCC GCG TGT CG6 GGT 5T6 GGC GGT GTC 5TT 5G5 GGG TCG CTT GGT CGG ATG GTT 6TC GTA GT6 GGT G5T 5AA 5T5 TA	80
DFT-17	AGG AAG G5C A6G ACG AA6 55T AA6 AAC GGG 5AG AAA CA5 G6A GAG 65G A55 6CA AGG ATG G6G 55C 5AA GGG 65G G6A 5A	80
DFT-18	GTG CCG G55 CTT GGG G5G CCA T6C TTG GGG TCG GCT CTG 5GT GTT T5T G6C CGT TGT TAG GGT TCG TCG TGG 55T T55 TA	80
DFT-19	AGT A55 AAG 5GG GGA 66T GC6 GGG 66A 5AT 6GG AAG 656 GTA G6G A55 6G6 5GG AA5 6AG G66 G5C CCG AGA AC6 C5T 5A	80
DFT-20	GAG GGG TTC T5G GGG CGG 66T GGT T5C GGC GGG T5G CTA CGG GCT T6C GAT GTG GCC 6GG CAG GTC 56C G6T TGG TA5 TA	80
DFT-21	AT5 55T A66 AGG A6A AAG AC5 G6G AAA ACG GGA GGG 555 5GA GTT G6A ACG 5GA GG5 6AC 6GA AT5 GG6 AAA G66 G6G GA	80
DFT-22	55G 6GG 6TT TG6 6GA TTC GGT GG5 CTC G6G TTG CAA 5TC GGG G5C CT5 C6G TTT TCG 5GG T5T TTG T66 TGG TAG GGA TA	80
DFT-23	ATG GTG 55G G5A 655 GAA GA5 G5A 6TG G6T 5G6 A5G GG6 C5A AG6 5AG 6GA 65G TGG G5A GGG 6TG TGA AA5 TGG GGT AA	80
DFT-24	TAC CC6 AGT TT6 ACA GC5 6TG CCC A6G GTC G6T GGC TTG GGC C6G TG5 GAG C5A GTG 6GT 5TT CGG GTG C6G G5A 55A TA	80
DFT-25	5A5 55T TA6 6GA A5A GCC GGT 5AG A6G AGA 5GG G66 A5C TAG 5TA G6A GG5 5TG AGA AG6 AC5 A5T A5G GGG 66C GG5 TA	80
DFT-26	AG6 5GG GCC CCG TAG TGG TGC TT6 T5A GG6 CTG CTA G6T AGG TGG CC6 GTC T6G TCT GA5 6GG CTG TTC GGT AAG GGT GA	80
DFT-27	5AG AA5 AGC AG5 5T6 A6A GTG 55T 656 GGG TGA C5G T5G GGG 5C6 6GT AGG GC5 CAG GA6 AG5 G55 G56 GGG 656 AAG AA	80
DFT-28	TCT TGG G66 5GG 5GG CGC TGT 66T GGG C5C TAC GGG GCC CCG A6G GT5 AC6 CGG GAG GCA 5TG TGA GGC TG6 TGT T5T GA	80
DFT-29	55A TT5 5TT 6TG AAG AGG AA6 G5C GT5 56A 6GG 55C ACT C6G 5GC A6A GGG T5A GCA CA5 G6C AGA G6A 5GA 5CG ACG AA	80
DFT-30	T5G TCG GT6 GTG 5TC TGG 6GT GTG 6TG A55 CTG TGC G6G GAG TGG GCC GTG GGA 5GG 6GT TC5 T6T TCA GAA GGA ATG GA	80
DFT-31	55T 5AA A6A G5T G6T 5AA 665 G5A ATG 6AG ATT A5G 6AA G5C 5G5 GTA AAT C5A 55A CGA G5G GC6 AGG GA6 565 GAC 6A	80
DFT-32	GGT 6GG GGT 6C5 TGG C6G 6TC GTG GTG GAT TTA 5GC GGG CTT GCG TAA T6T GCA TTG 5GG GTT GAG CAG 5TG TTT GAG GA	80
DFT-34	GTG TT5 G55 C6T CC5 CTG 5TG G5G GGC GGT GGG GT6 TGG GAC CCG T65 C6G TGT ATG G5G 6TT GTT 6CG GC6 T5G AT5 GA	80
DFT-35	5TA GGT 5GG ACG C6A GGT GCC 5AC G6G GGA 556 GG5 T6A AAA TAG GGG 6C5 GG5 A65 5C6 GG5 65T AA5 T5G GAC ACG GA	80
DFT-36	5CG TGT CCG AGT TAG GGC CGG GGG TG6 CGG G6C 66T ATT TTG AGC CGG GT5 C6G CGT GGG CAC 6TG G5G TCC GA5 5TA GA	80
DFT-37	5TG G5T AGG ATG G5G G6G AGG 6A5 G5G GA6 CGA AA6 ACA GG6 GA5 G56 G6G A5A AT6 GGC AAG GG5 556 GAG 6GG 6G5 GA	80
DFT-38	CGC G5C G6T 5GG GG5 5CT TG5 5GA TTG TCG 5GG CGT CGC CTG TGT TTC GGT 56G CGT GC6 T6G CCG 55A TCC TAG 55A GA	80

DFT-39	GA5 AG5 A6G A6G 5A5 C6G G65 GAC GG6 A66 55G T6G GG5 5GG 66A 6AG ACC 5TG G65 5A5 GAA AGG AA5 CGA 665 55C GA	80
DFT-40	5GG GGG GT5 GGT TC6 TTT 5GT GGG 5CA GGG T6T GTG G5C GGC 55G A6G GGG TG6 5GT CGG C5G GGT GCG T6G TG5 TGT 5A	80
DFT-41	GAG GGA TAA G5C GAA AAA CAG 6C5 AAA 65T TGG AA6 TC6 G66 AGG AGA CA5 GAG GG5 C5G GGG AC5 G56 A6A 5GA GGA CA	80
DFT-42	GT5 CTC GTG TGG CGG TCC C5G GG5 CCT CGT GT5 T6C TGG 6GG AGT T65 AAG GTT TGG GCT GTT TTT CGG CTT AT5 55T 5A	80
DFT-43	G5A AT5 556 GGA GC5 ACG GGG 56G GGA ACG GAA AG5 GA6 AGG 5TA 5CG AA5 5G5 AA6 GGG GAA AG5 65G 6GA 55A T5A GA	80
DFT-44	CTG ATG GTC G5G GGC TTT C56 CGT TG5 GGT TCG GTA GCC TGT 5G5 TTT C5G TT5 CCG G6C C5G TGG 6TC CGG GGA TTG 5A	80
DFT-45	G5G 5TT TA5 5GG GAA ACA GGC GA5 AAG AG6 55A GC6 G65 AGT GGA 656 GGA G65 A5G GAA TG6 ATA GGT CAA GGA GCA AA	80
DFT-46	TTG CTC CTT GAC CTA TG6 ATT CCG TGG CT6 5GG GT5 5A6 TGG CGG 6TG GG5 T5T TGT CG5 CTG TTT 5CC GGT AAA G5G 5A	80
DFT-47	GGA 55T TCG GAG CAA 6AA GAC 5CG AA5 5GG 6GG A5A CAG AAA 5G6 A6A GG5 GTA CA6 6GA 556 G6A 5AG G6G GA5 A55 GA	80
DFT-48	CGG TGT 5CG C6T GTG 5GG GT6 GGT GTA CGC CTG TGC GTT T5T GTG TCC GC5 GGT TCG GGT 6TT GTT G6T C5G AAG GT5 5A	80
DFT-49	GGG ATT G6A 6CG A6A 6GG 5GG A66 AAG A56 5GA AA6 GGG C5A AAA AT6 555 AAA ACG TGC CAA 5CC GAA A5A 5C5 AGG GA	80
DFT-50	5C5 TGG GTG TTT 5GG GTT GG5 A6G TTT TGG GGA TTT TTG GCC 6GT TT5 GGG T6T TGG T56 GCC GTG T5G GTG 5AA T55 5A	80
DFT-51	GGT G5T A6G A6C 6GG GG6 CCA AGG GGG 5C5 5GG G6G 6AA AAG 556 CAA AAA GG6 GG5 AAA AG5 CAT 5AA AAT G5C T6A AA	80
DFT-52	TTG AGG 6AT TTT GAT GG5 TTT TGC CG6 5TT TTT GGG G5T TTT G6G C65 GGG GC5 CCC TTG GG5 CC6 GGG T6G TAG 5A5 5A	80
DFT-53	GT5 GTA TA5 T5C GAA 5AA 5GA GGT 5AA 5AG 6AG GAA 5A6 G6G ATA 5C5 AGA GAT 6AC CGG GG6 C6C 6GG AA6 6C6 5GA 5A	80
DFT-54	GTC GGG GGT TC6 GGG GGC CCC GGT GAT CTC TGG GTA T5G 5GT GTT 6CT G5T GTT GA5 CT5 GTT GTT 5GG AGT ATA 5GA 5A	80
DFT-55	GTT G55 AG6 665 6AA 5GA 6AA G5G GGG GGT 5AG AAG AAG ATA 5T5 55G G5T 5AA AGG AGG TAG TTT GAA CCT TAG CTA GA	80
DFT-56	CTA GCT AAG GTT CAA ACT ACC TCC TTT GAG 55G GGA GTA T5T TCT T5T GA5 C6C CCG 6TT GTC GTT GGG GGC TGG 5AA 5A	80

SUPPORTING REFERENCES

[1] A. S. Schroder, et al., *Angew. Chem. Int. Ed.* **2014**, *53*, 315-318.
[2] P. Elias, *IEEE Trans. Inf. Theory* **1975**, *21*, 194-203.
[3] a) G. M. Church, Y. Gao, S. Kosuri, *Science* **2012**, *337*, 1628; b) N. Goldman, et al., *Nature* **2013**, *494*, 77-80.

SUPPORTING DATA

Herein we provide the sequencing results together with the analysis of the following experiments:

The Raven (1st stanza, Readout 1)	p27
The Raven (2nd stanza, Readout 2)	p53
Portrait of DARWIN (Readout 1)	p79
Portrait of FRANKLIN (Readout 2)	p109
Portrait of TURING (Readout 3	p139
Redox cycle (S1, one oxidation / no reduction steps, FRANKLIN)	p169
Redox cycle (S0, one oxidation / one reduction steps, TURING)	p195
Summary of all redox cycles	p229

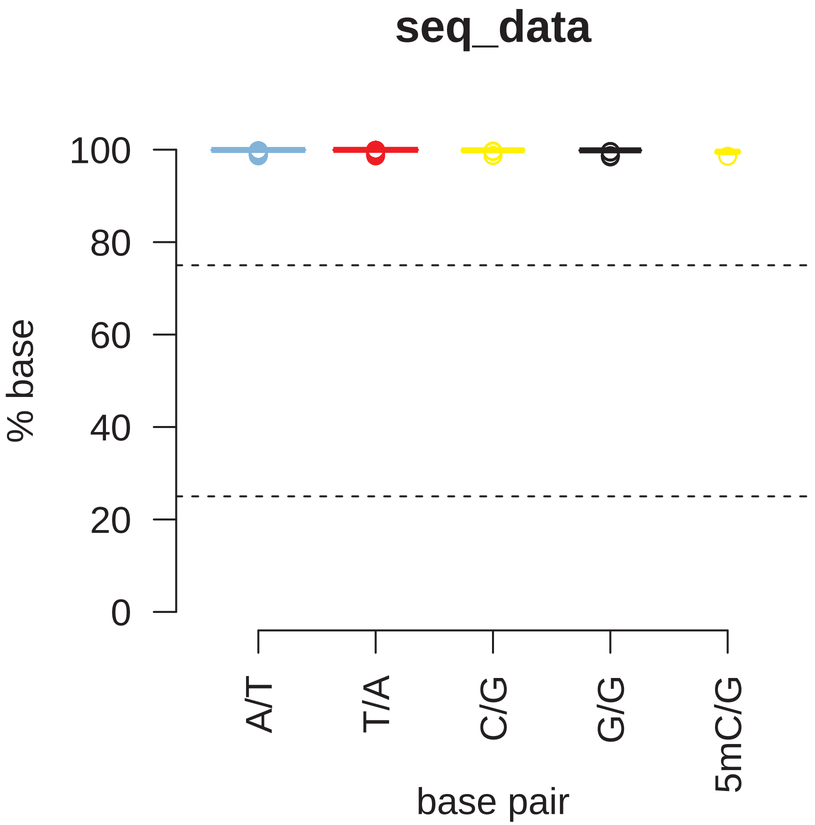
seq_data
100
80
60
40
20
0
% base
A/T
T/A
C/G
G/G
5mC/G
base pair

Once upon a midnight dreary, while I pondered, weak and weary,
Over many a quaint and curious volume of forgotten lore —
While I nodded, nearly napping, suddenly there came a tapping,
As of some one gently rapping, rapping at my chamber door.
"'Tis some visiter," I muttered, "tapping at my chamber door —
Only this and nothing more."

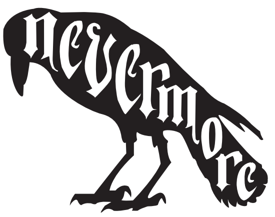

THE RAVEN

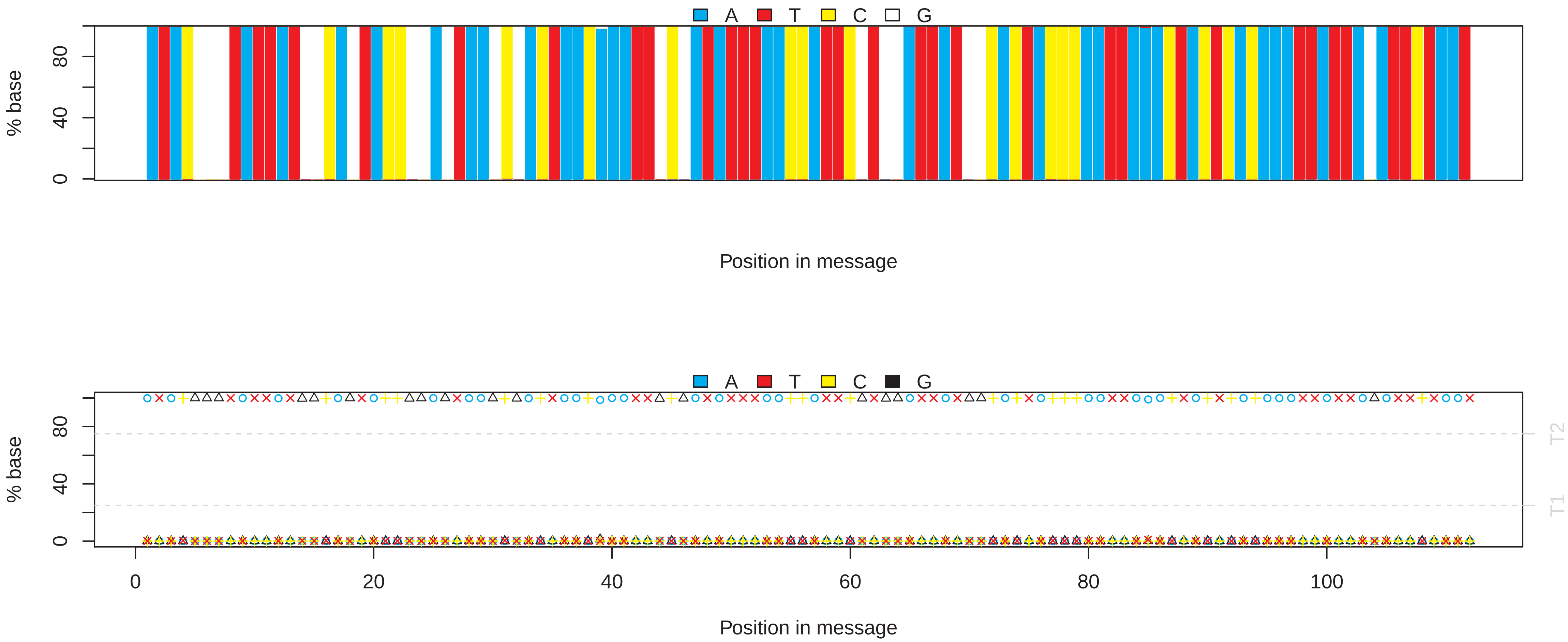

0100111101101110011000110110010100100000011101010111000001101111011011100010000001100001001000000110110101101001

Once upon a mi

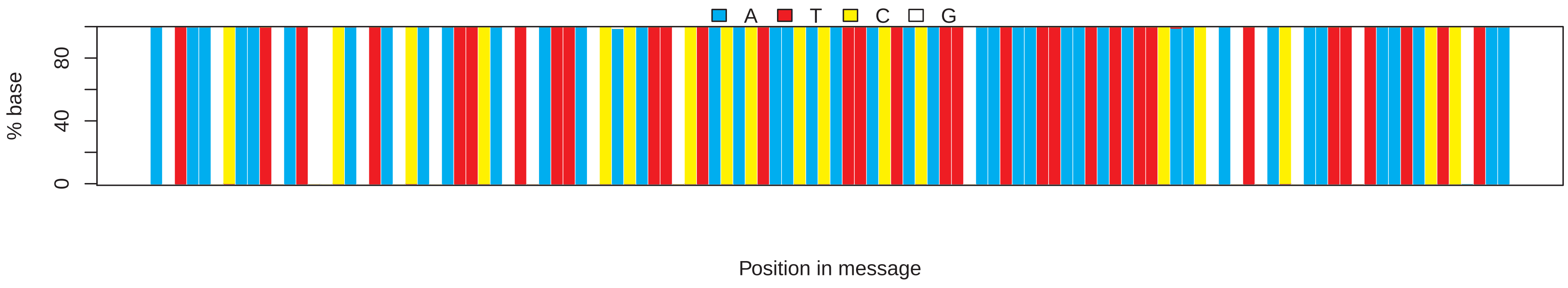

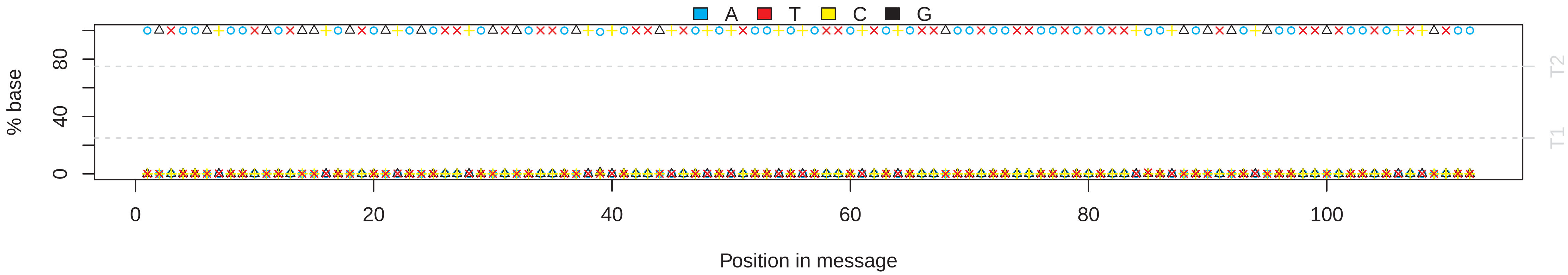

0110010001101110011010010110011101101000011101000010000001100100011100100110010101100001011110010010110‌0

dnight dreary,

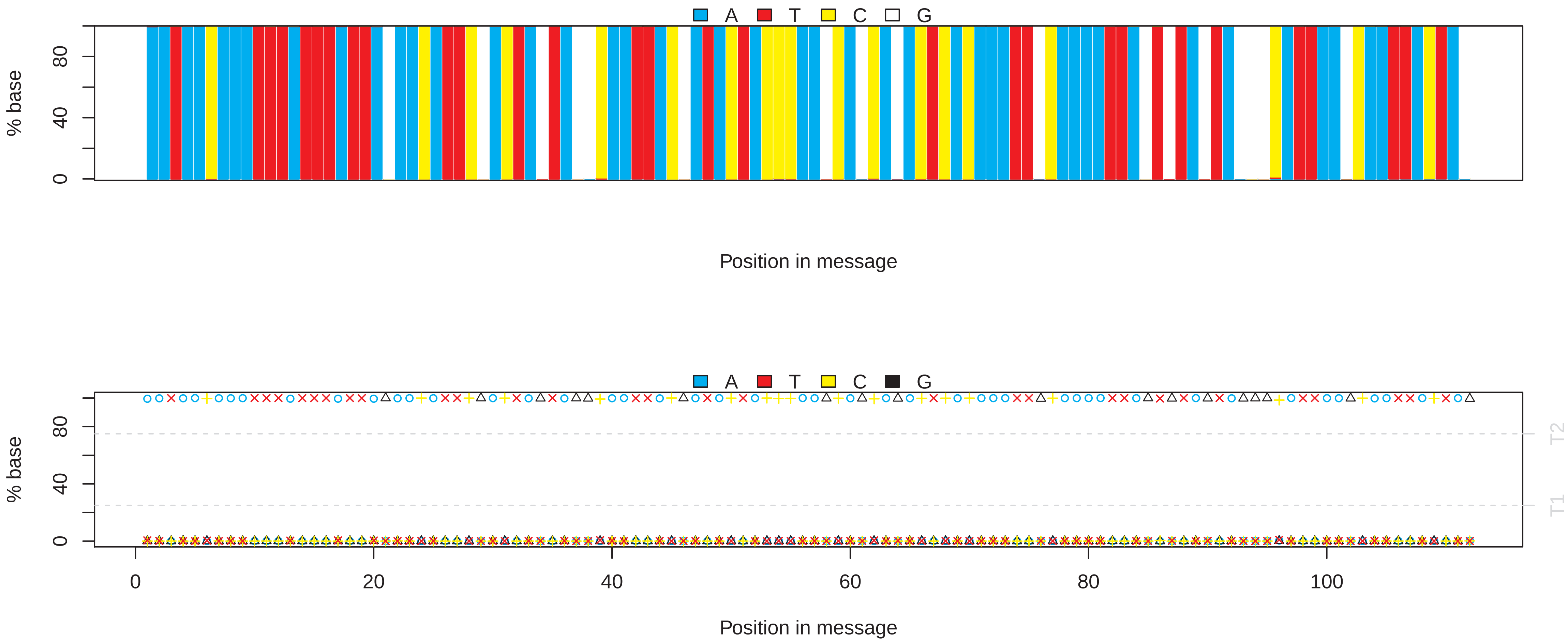

0010000001110111011010000110100101101100011001010010000001001001001000000111000001101111011011100110010001100101

while I ponde

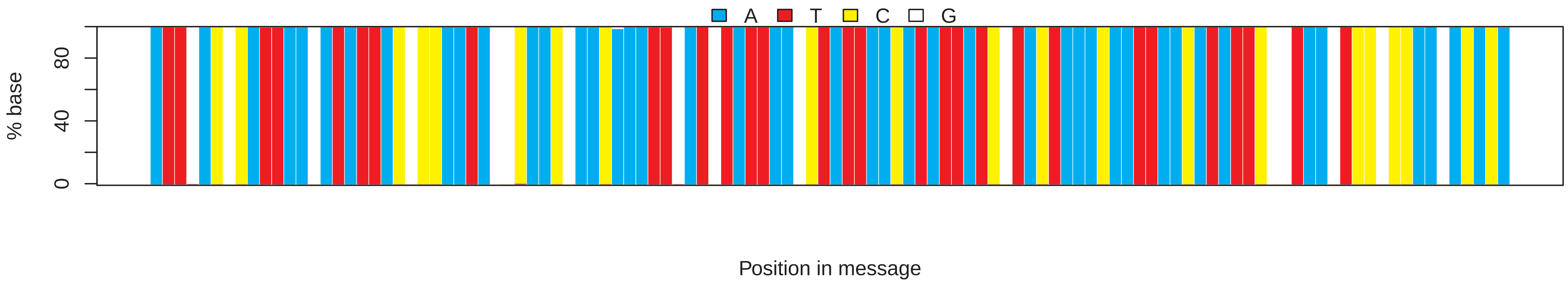

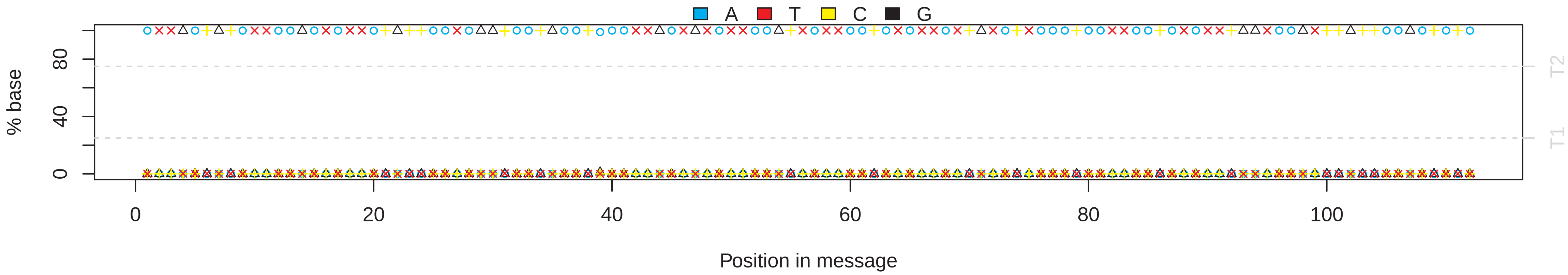

0111001001100101011001000010110000100000011101110110010101100001011010110010000001100001011011100110010000100000

red, weak and

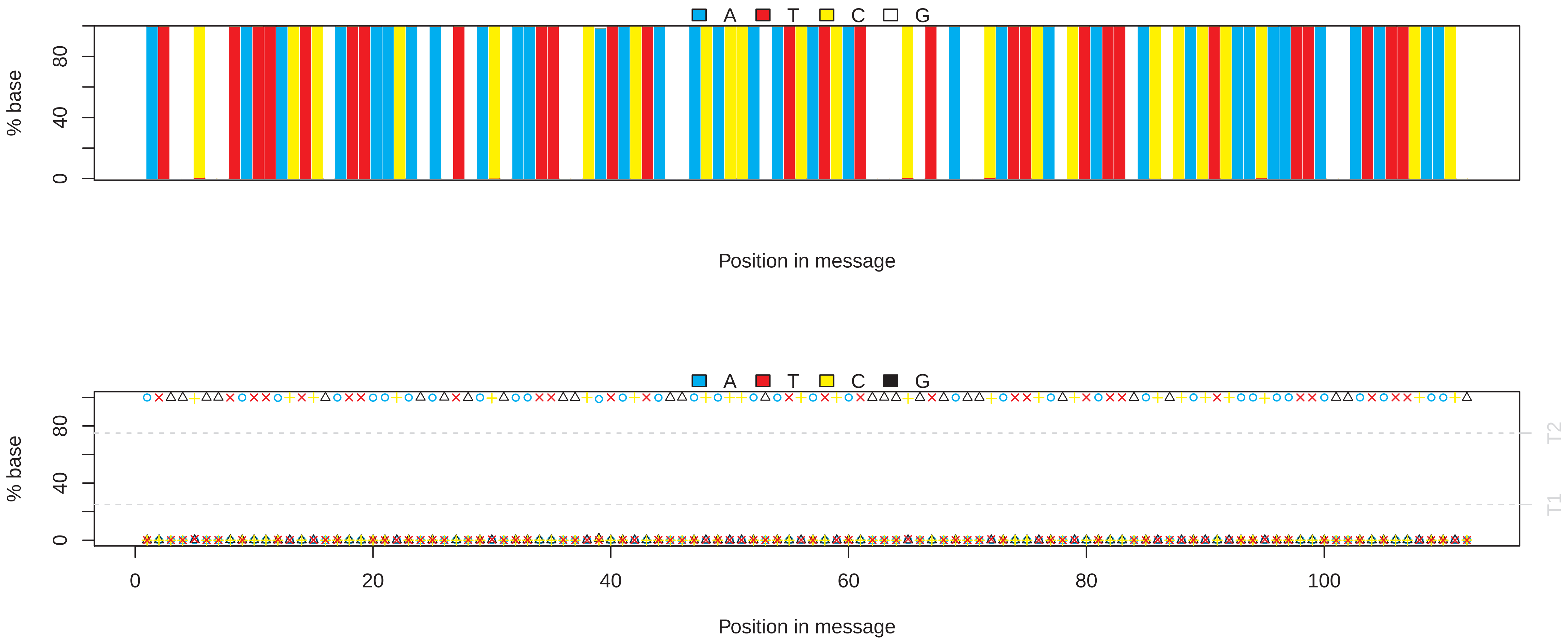

0111011101100101011000010111001001111001001011000000101001001111011101100110010101110010001000000110110101100001

weary,
Over ma

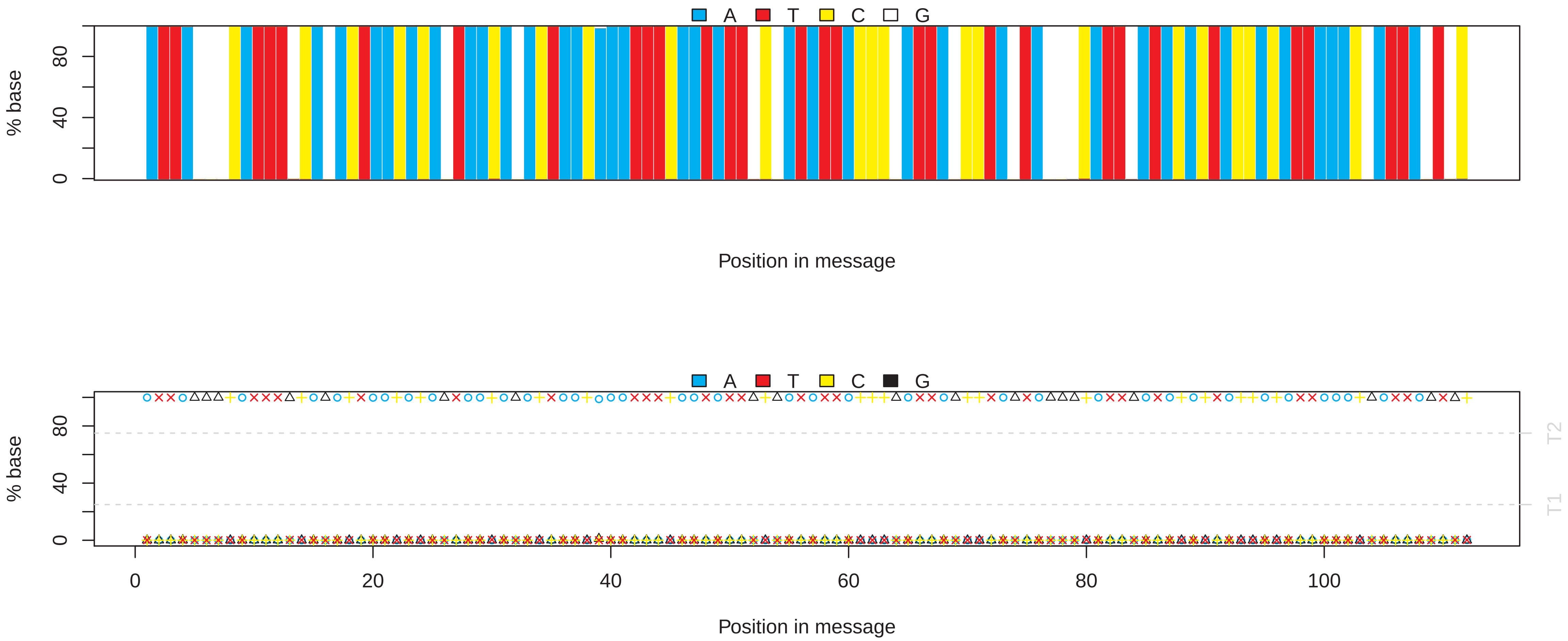

0110111001111001001000000110000100100000011100010111010101100001011010010110111001110100001000000110000101101110

ny a quaint an

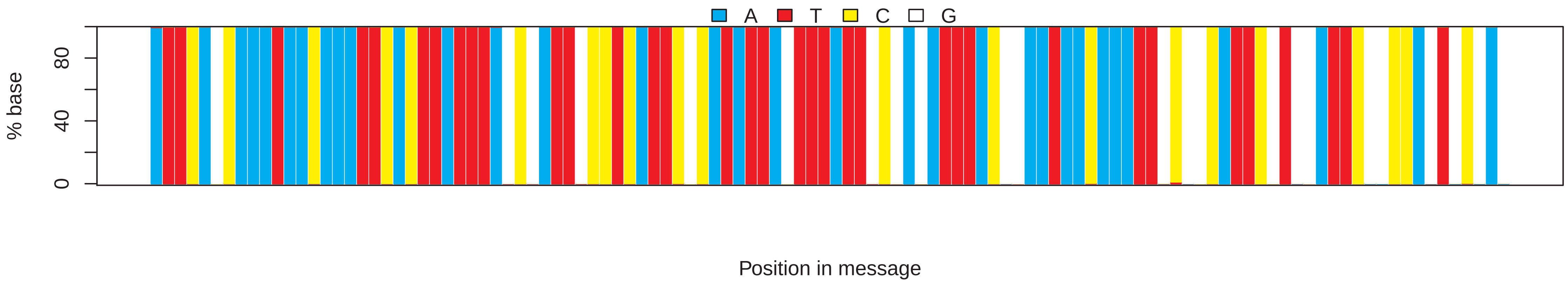

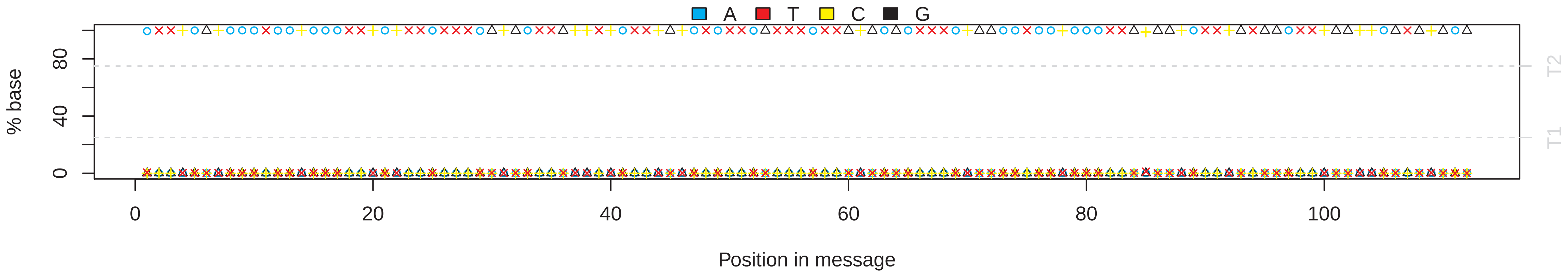

0110010000100000011000110111010101110010011010010110111101110101011100110010000001110110011011110110110001110101

d curious volu

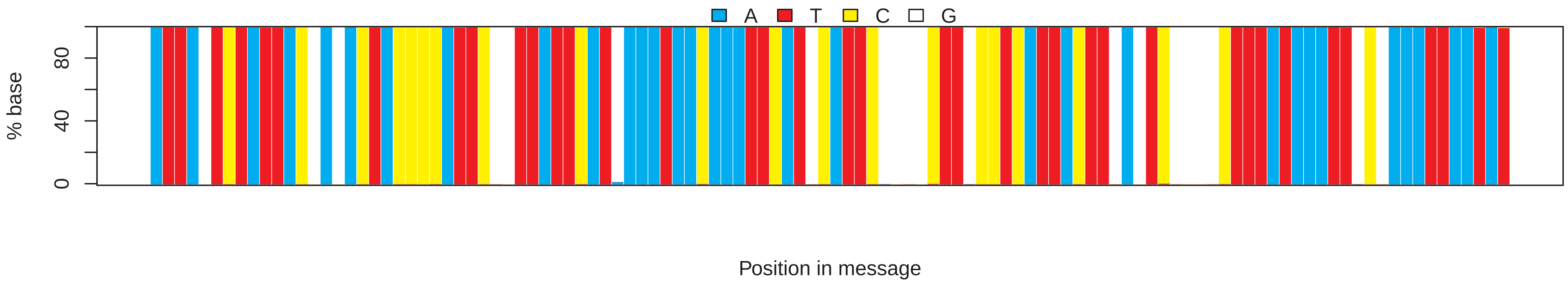

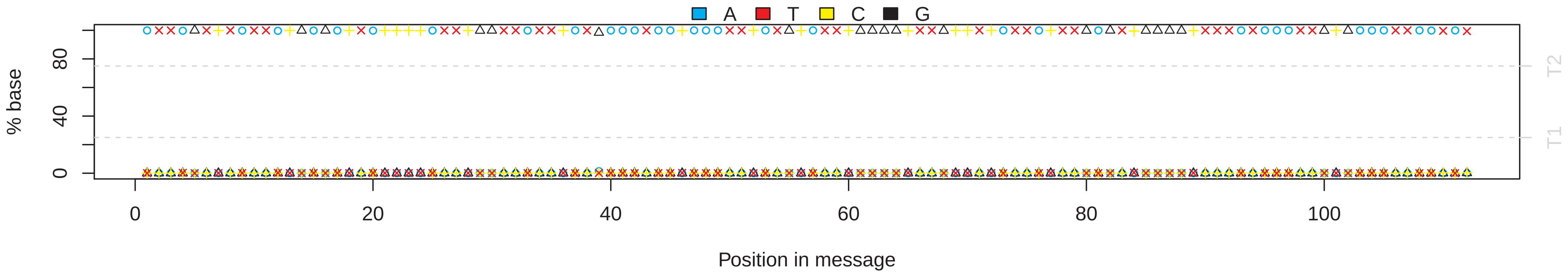

0110110101100101001000000110111101100110001000000110011001101111011100100110011101101111011101000111010001100101

me of forgotte

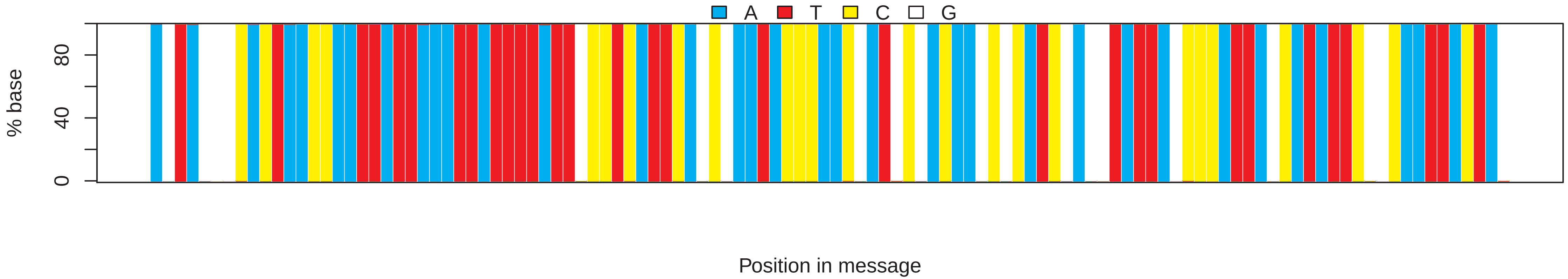

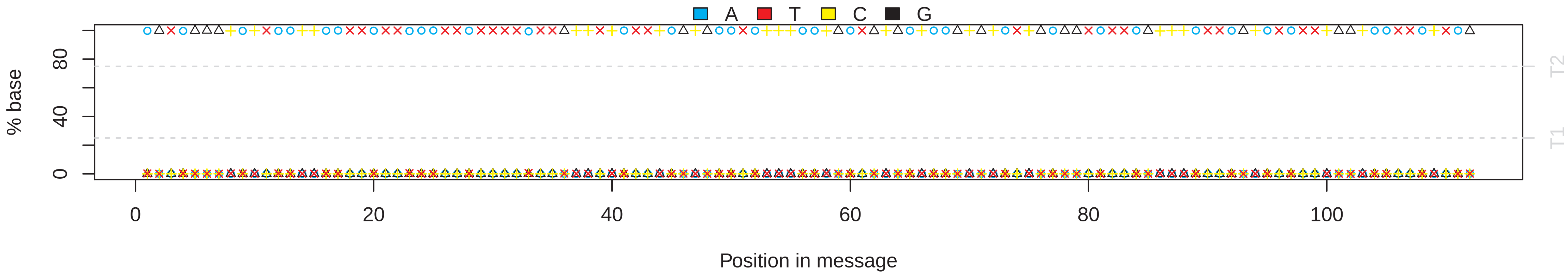

011011100010000001101100011011110111001001100101001000000101101000010100101011101101000011010010110110001100101

n lore −
While

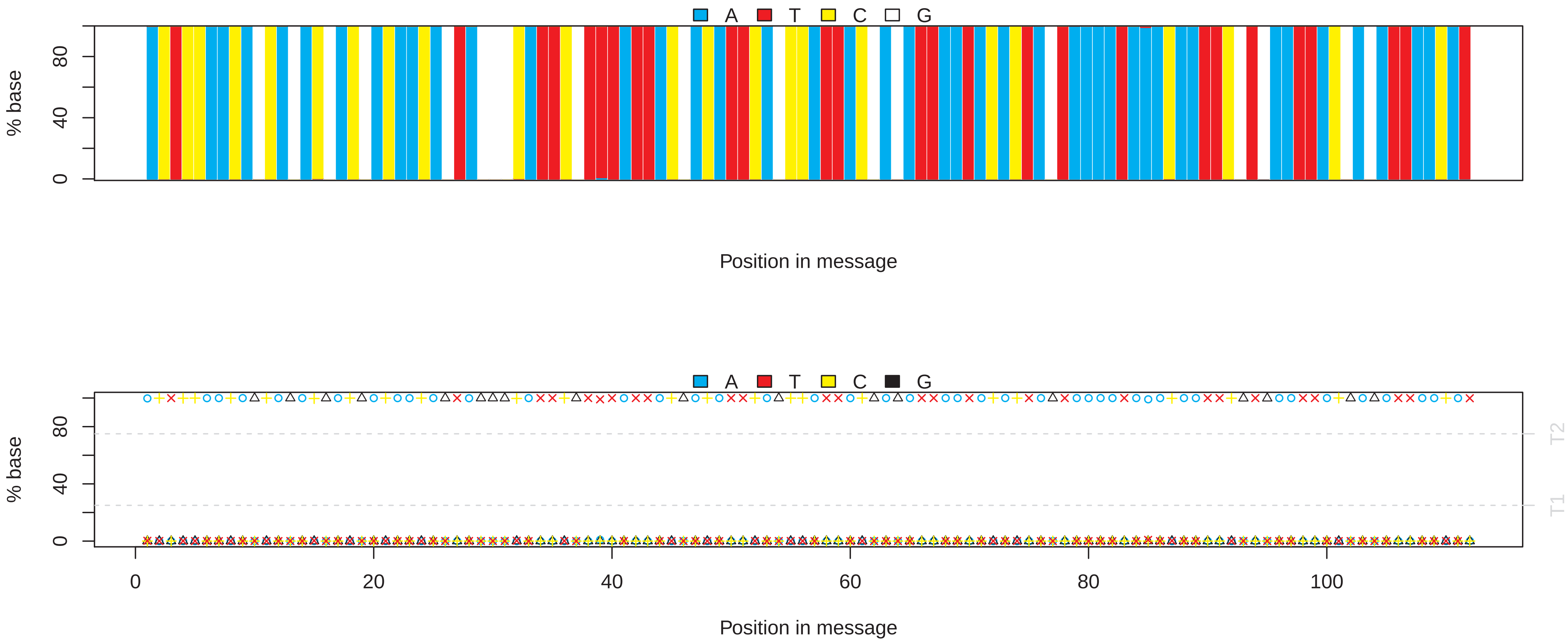

0010000001001001001000000110111001101111011001000110010001100101011001000010110000100000011011100110010101100001

I nodded, nea

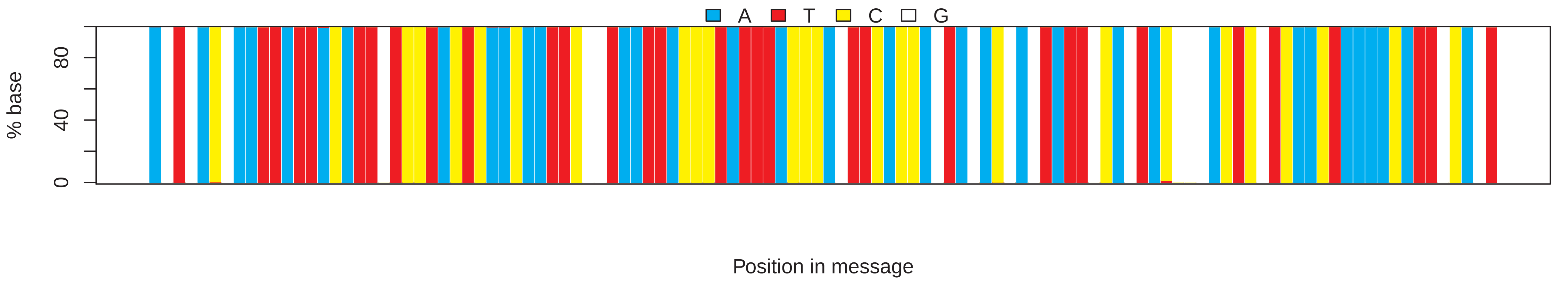

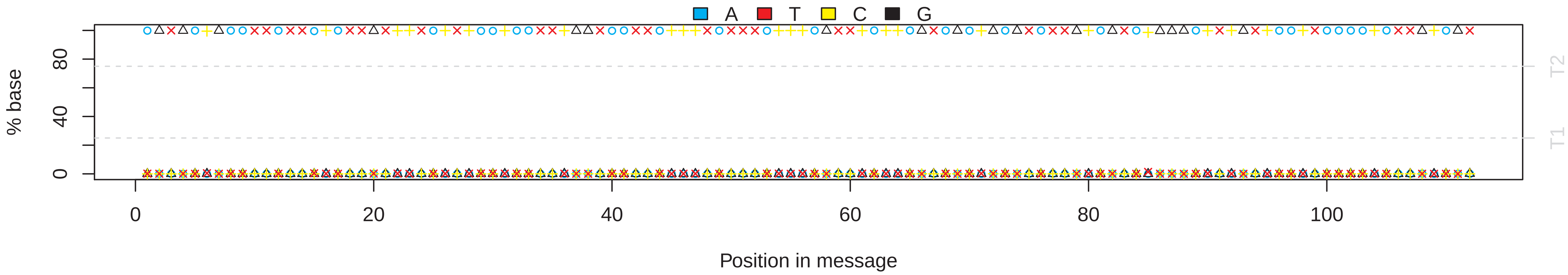

0111001001101100011110010010000001101110011000010111000001110000011010010110111001100111001011000010000001110011

rly napping, s

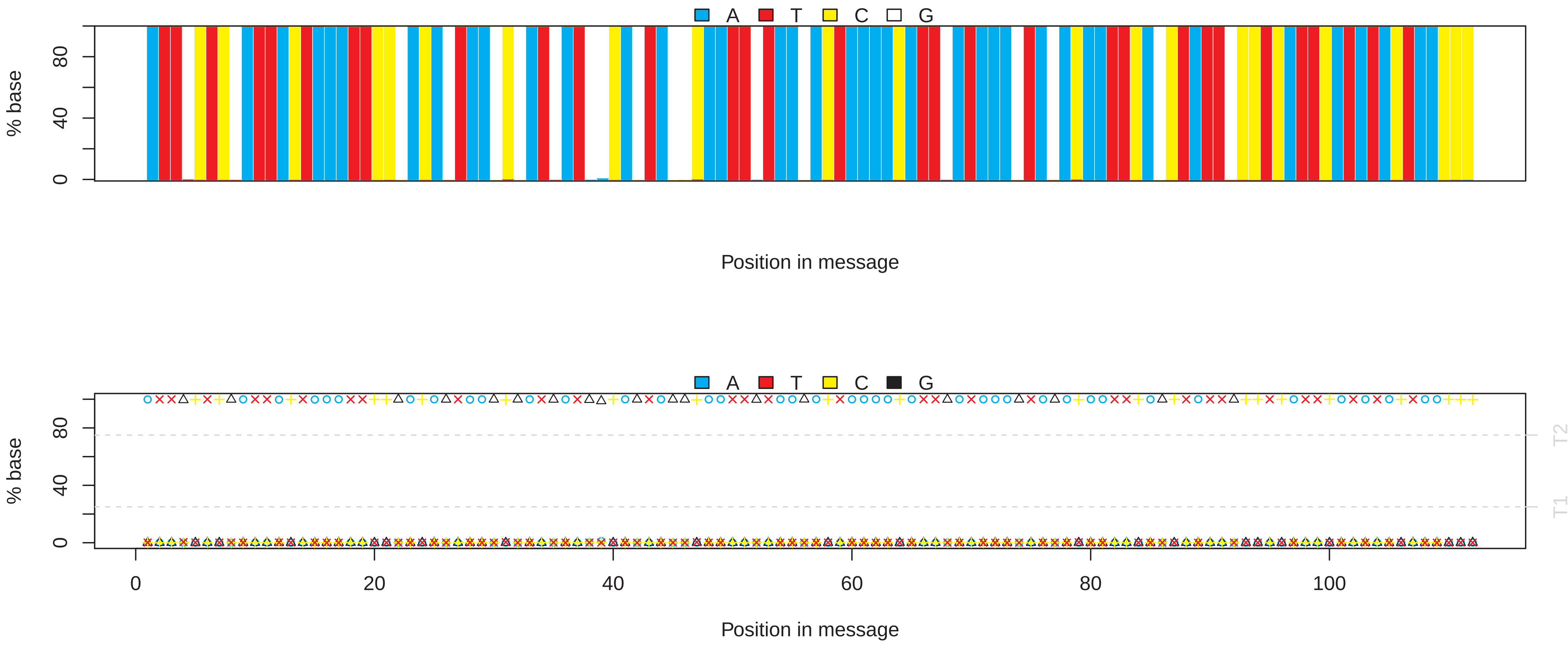

0111010101100100011001000110010101101110011011000111100100100000011101000110100001100101011100100110010100100000

uddenly there

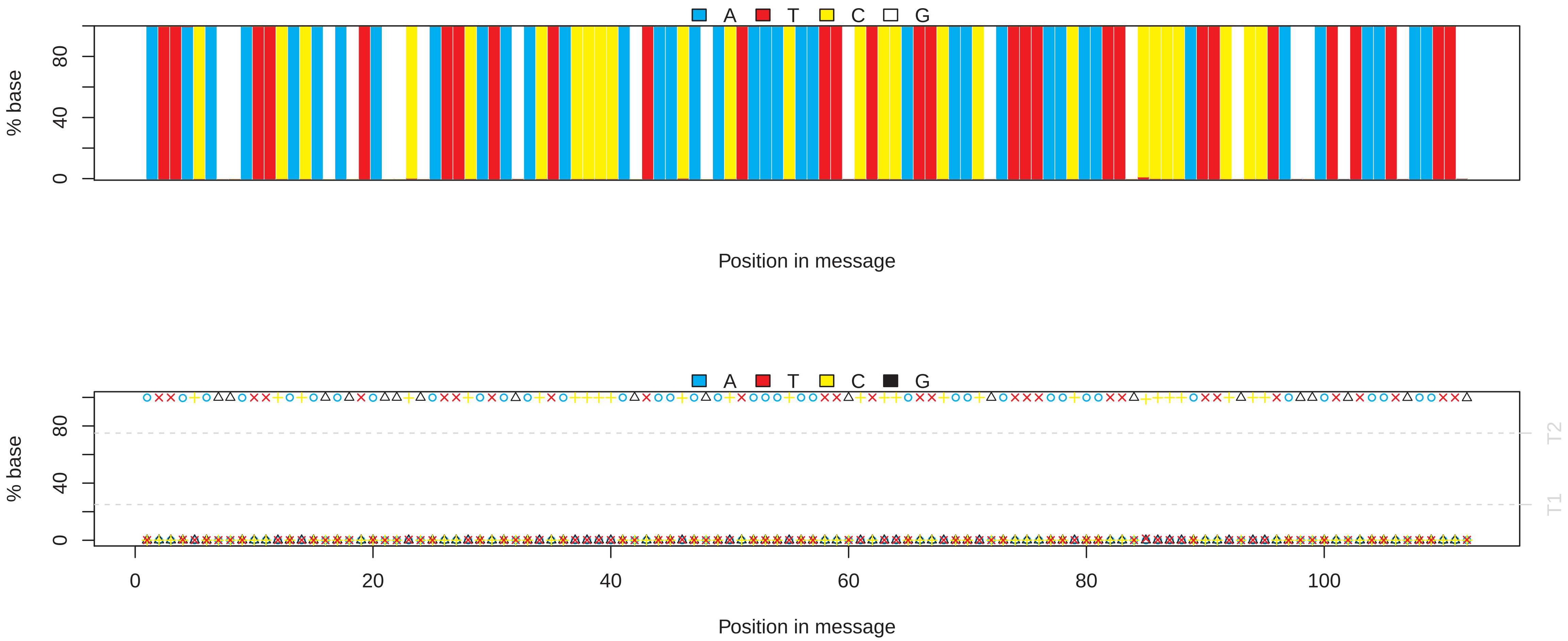

0110001101100001011011010110010100100000011000010010000001110100011000010111000001110000011010010110111001100111

came a tapping

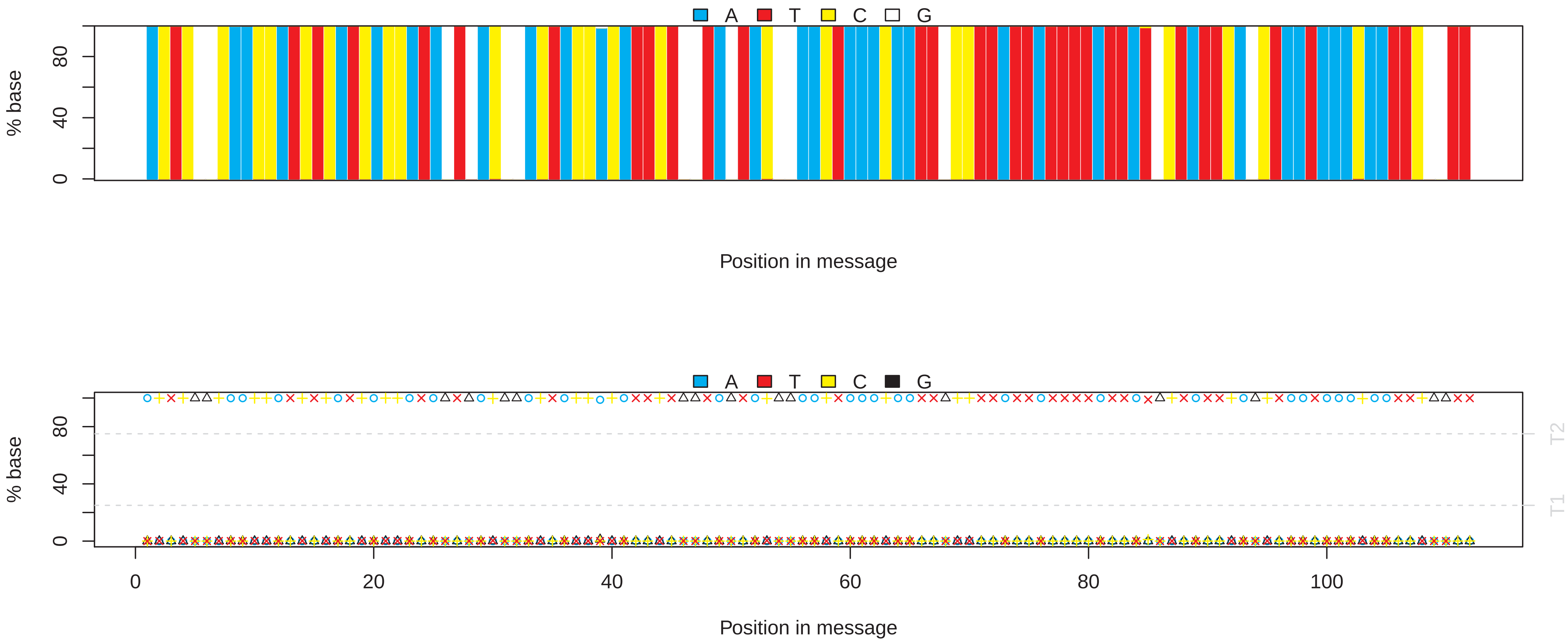

0010110000001010010000010111001100100000011011110110011000100000011100110110111101101101011001010010000001101111

,
As of some o

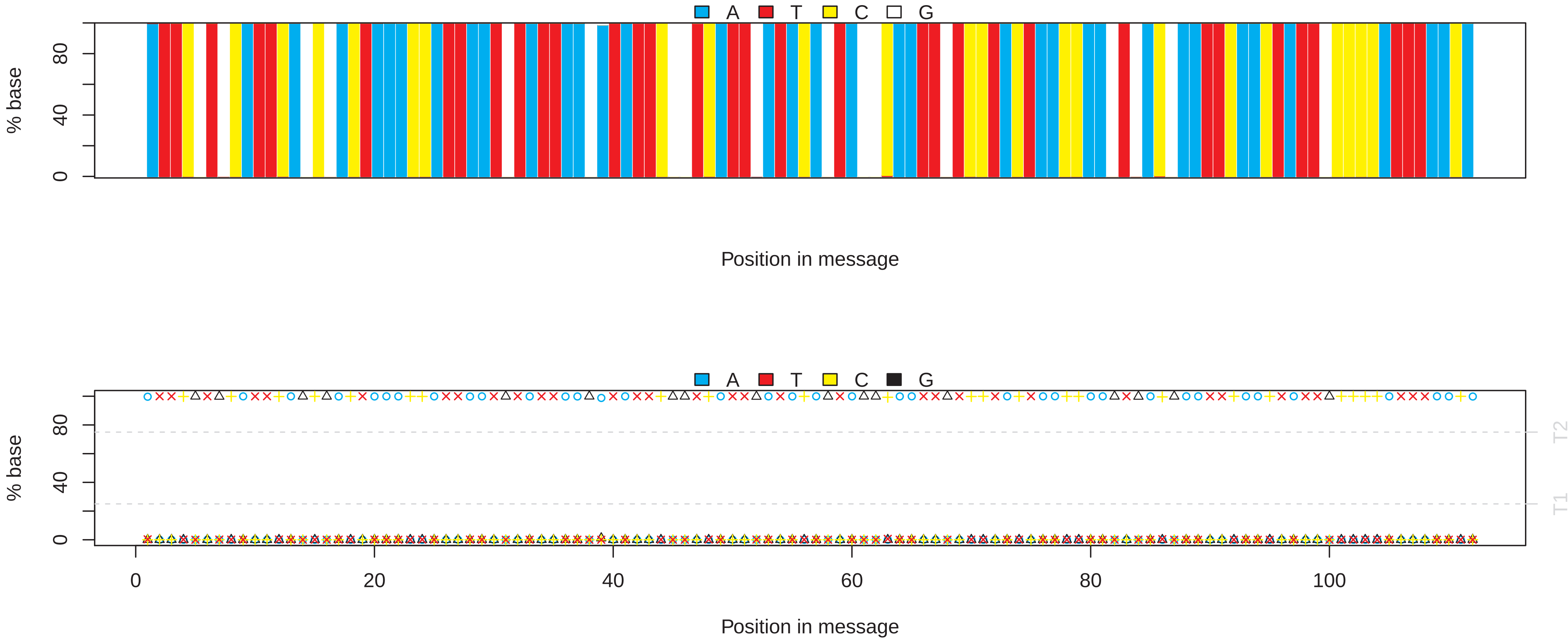

0110111001100101001000000110011101100101011011100111010001101100011110010010000001110010011000010111000001110000

ne gently rapp

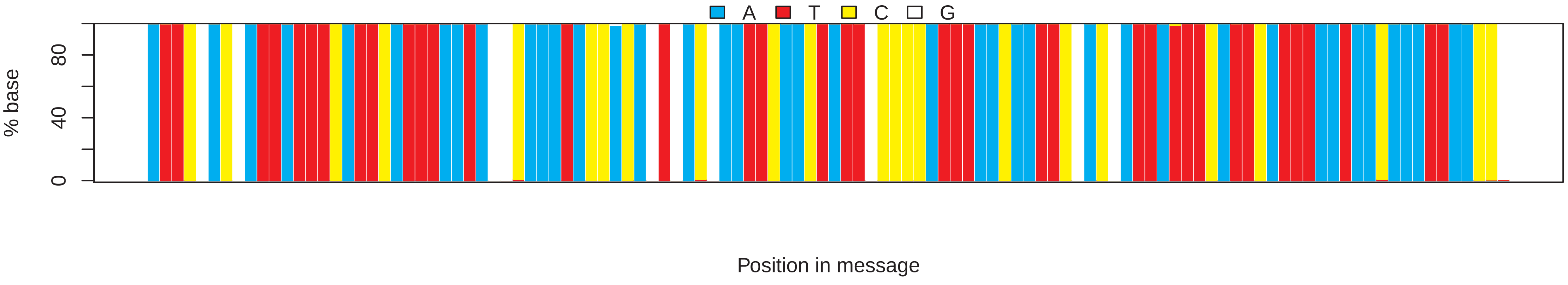

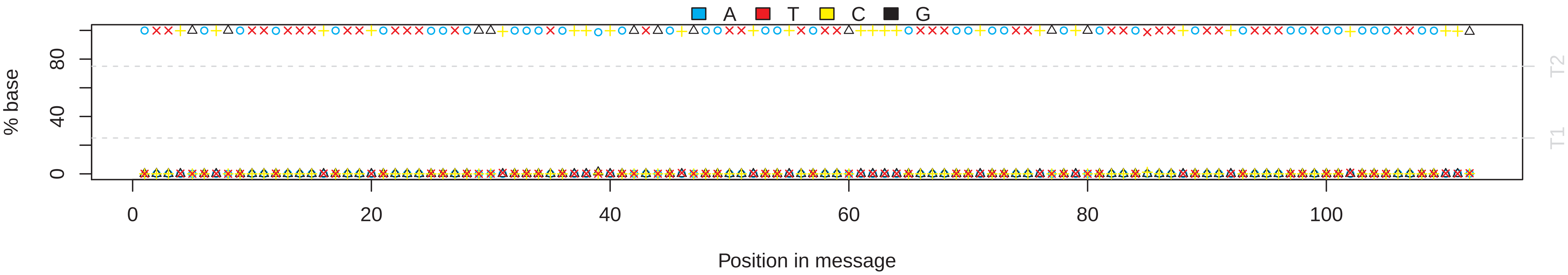

0110100101101110011001110010110000100000011100100110000101110000011100000110100101101110011001110010000001100001

ing, rapping a

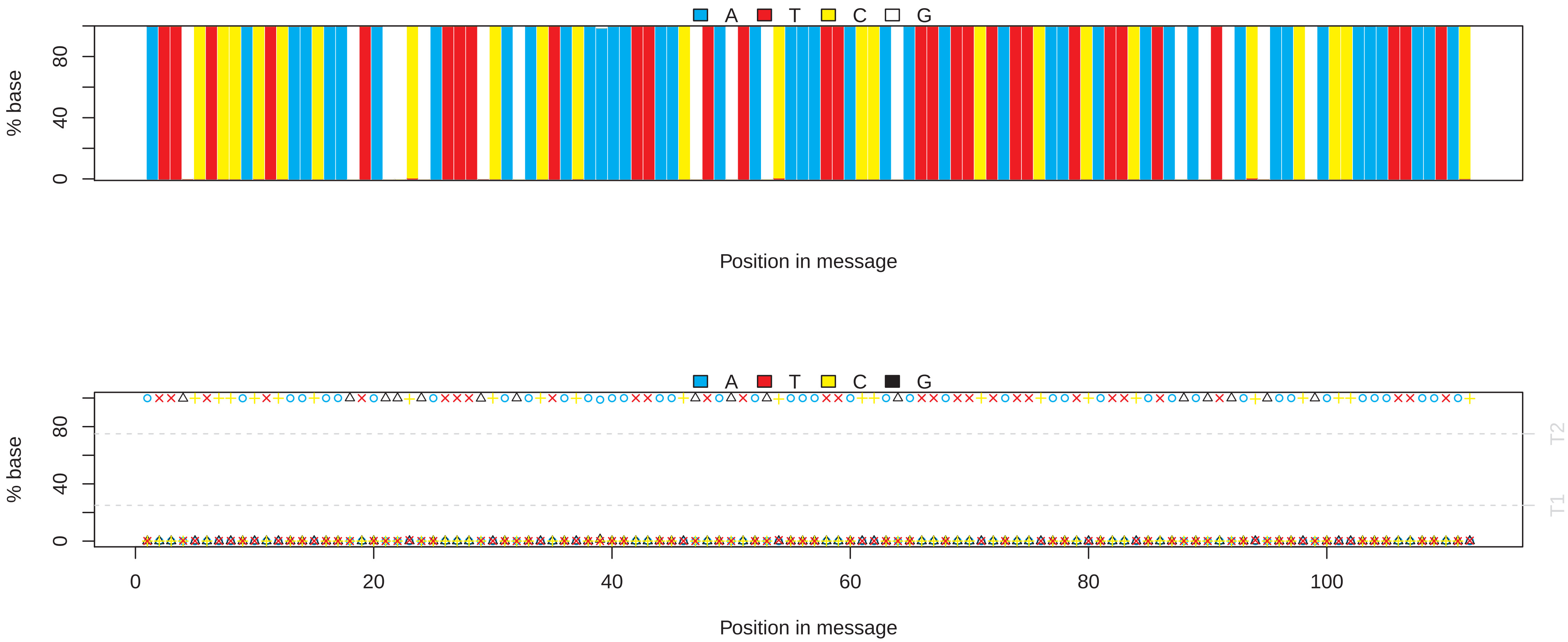

0111010000100000011011010111100100100000011000110110100001100001011011010110001001100101011100100010000001100100

t my chamber d

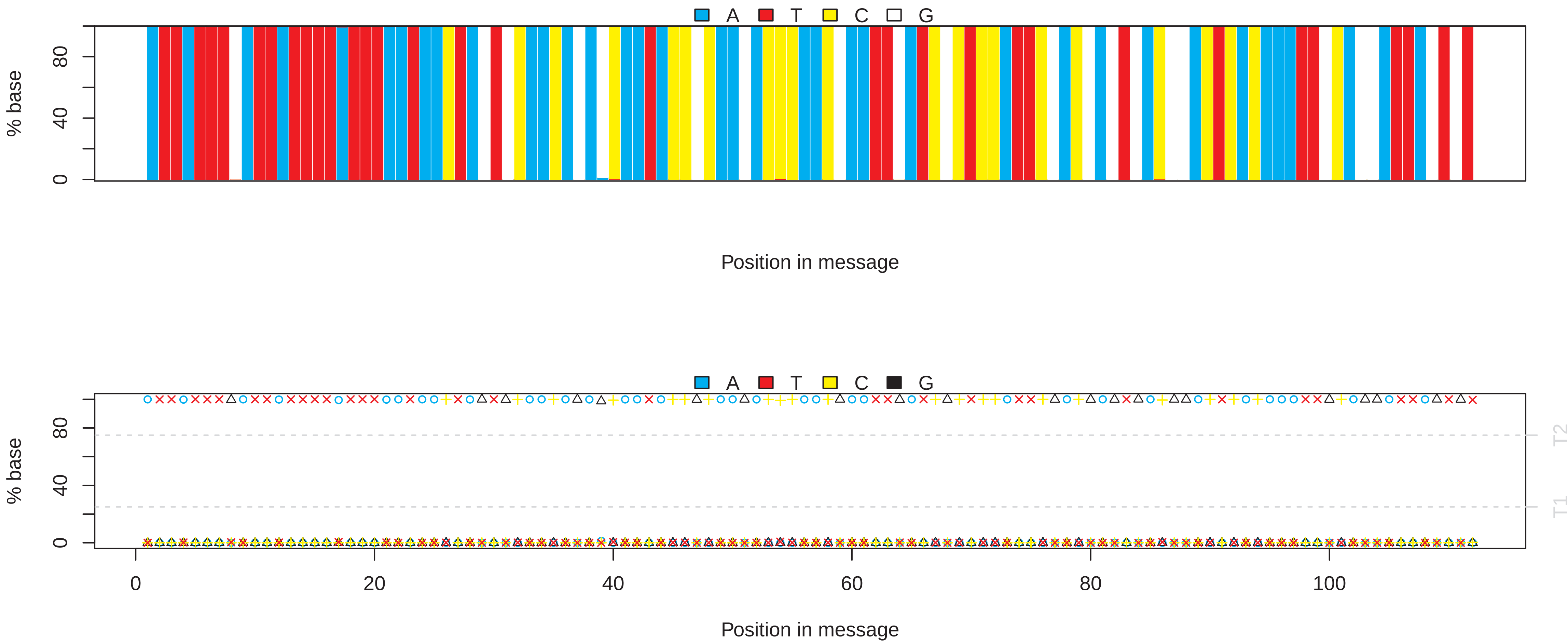

0110111101101111011100100010111000000101000100010001000000001001110101010001101001011100110010000000111001101101111

oor.

" 'Tis so

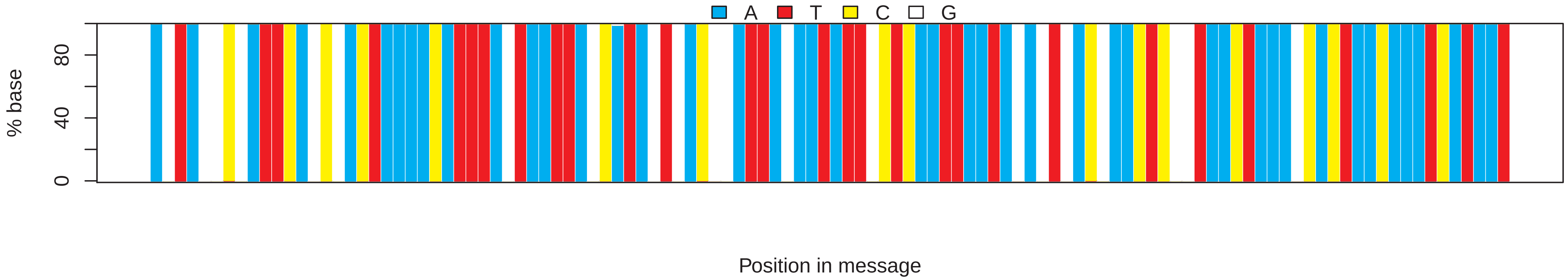

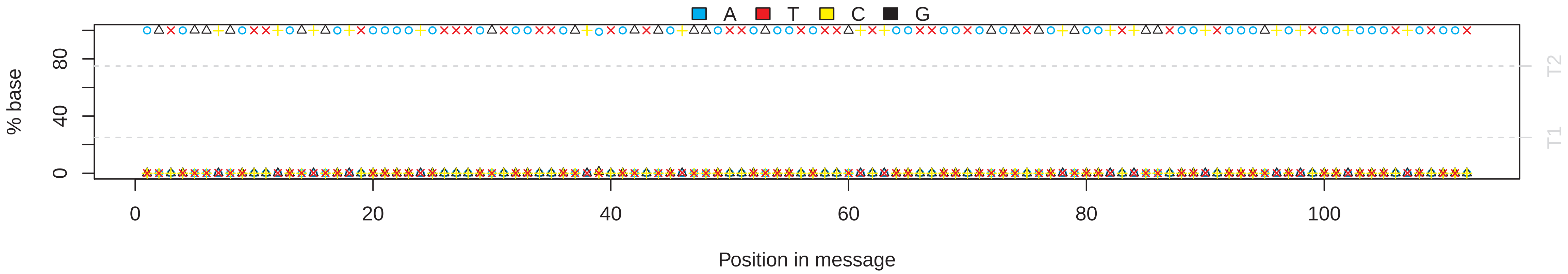

0110110101100101001000000111011001101001011100110110100101110100011001010111001000101110001000100010000001001001

me visiter." I

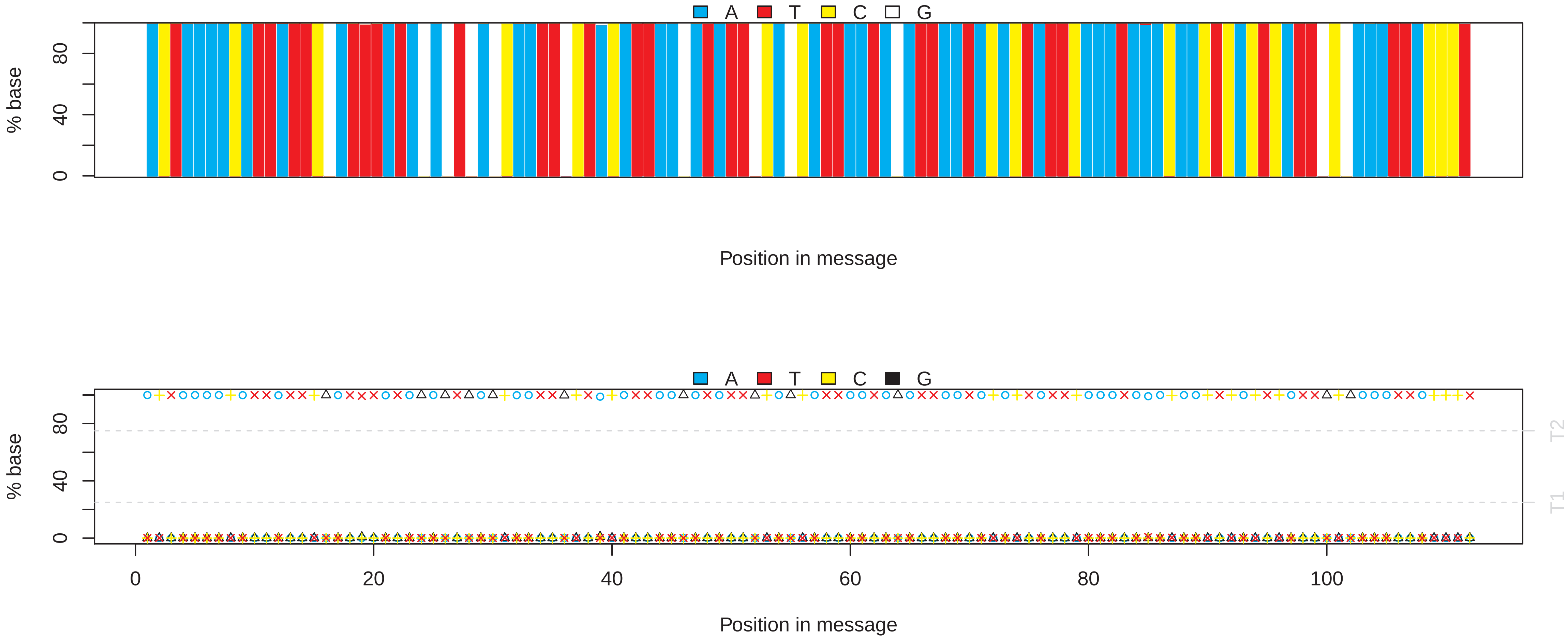

0010000001101101011101010111010001110100011001010111001001100101011001000010110000100000001000100111010001100001

muttered, "ta

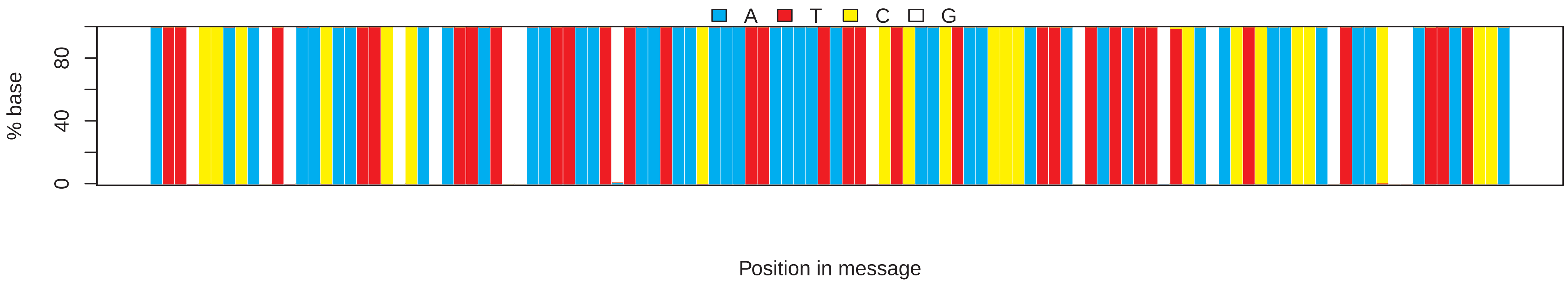

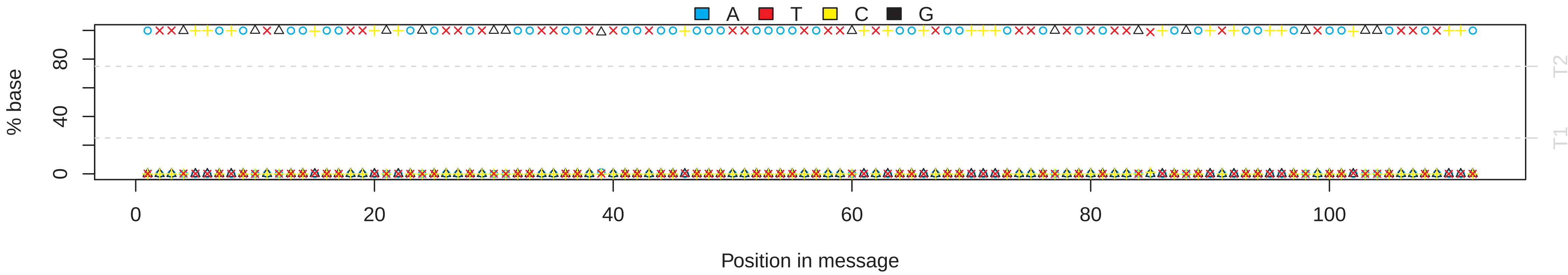

0111000001110000011010010110111001100111001000000110000101110100001000000110110101111001001000000110001101101000

pping at my ch

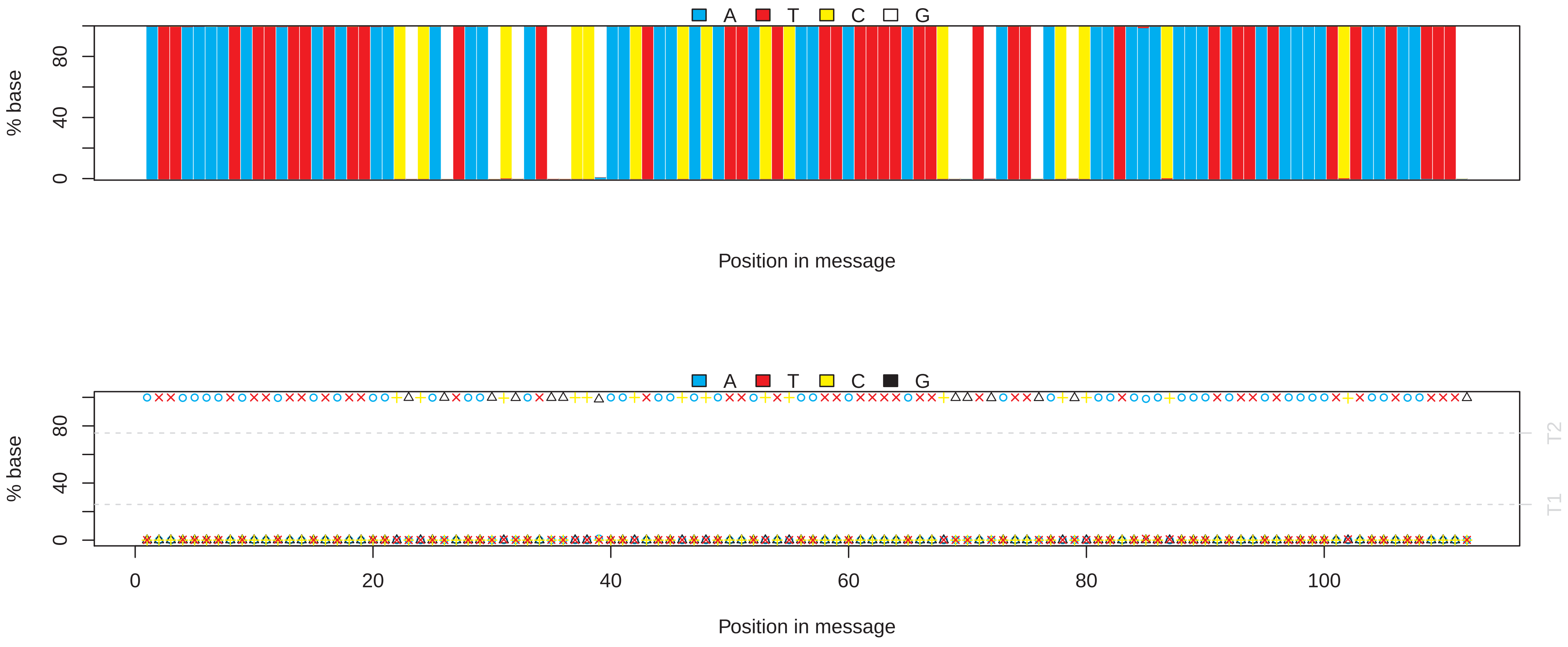

0110000101101101011000100110010101110010001000000110010001101111011011110111001000100000001011010000101001001111

amber door −
O

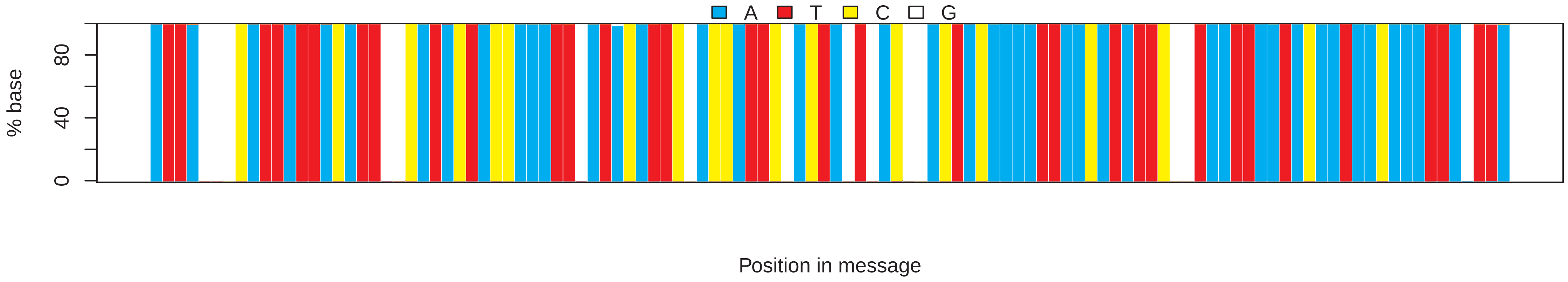

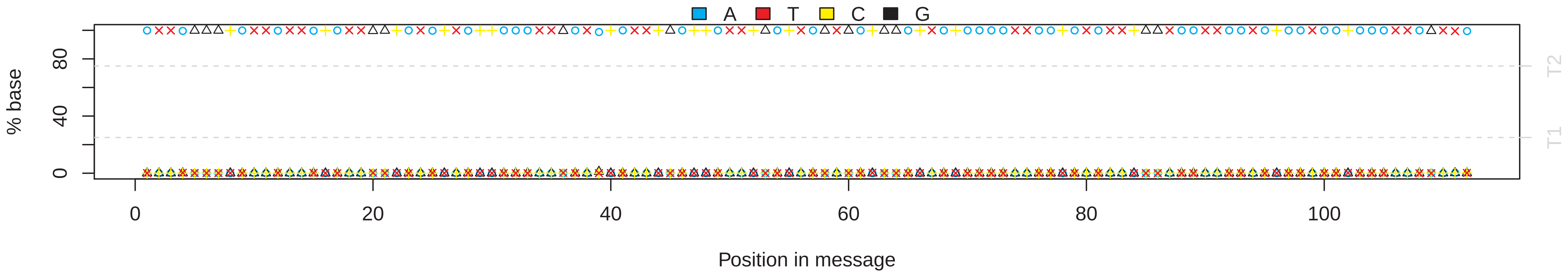

0110111001101100011110010010000001110100011010000110100101110011001000000110000101101110011001000010000001101110

nly this and n

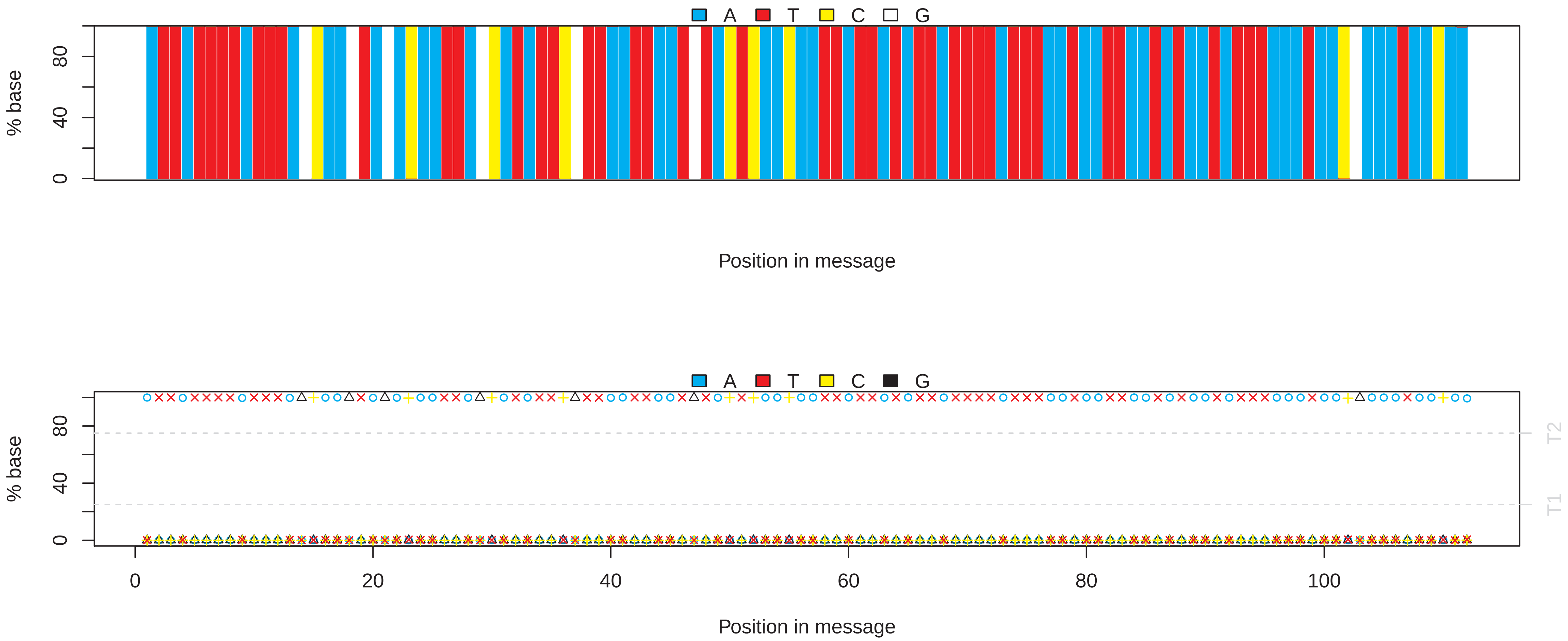

0110111101110100011010000110100101101110011001110010000001101101011011110111001001100101001011100010001000100000

othing more."

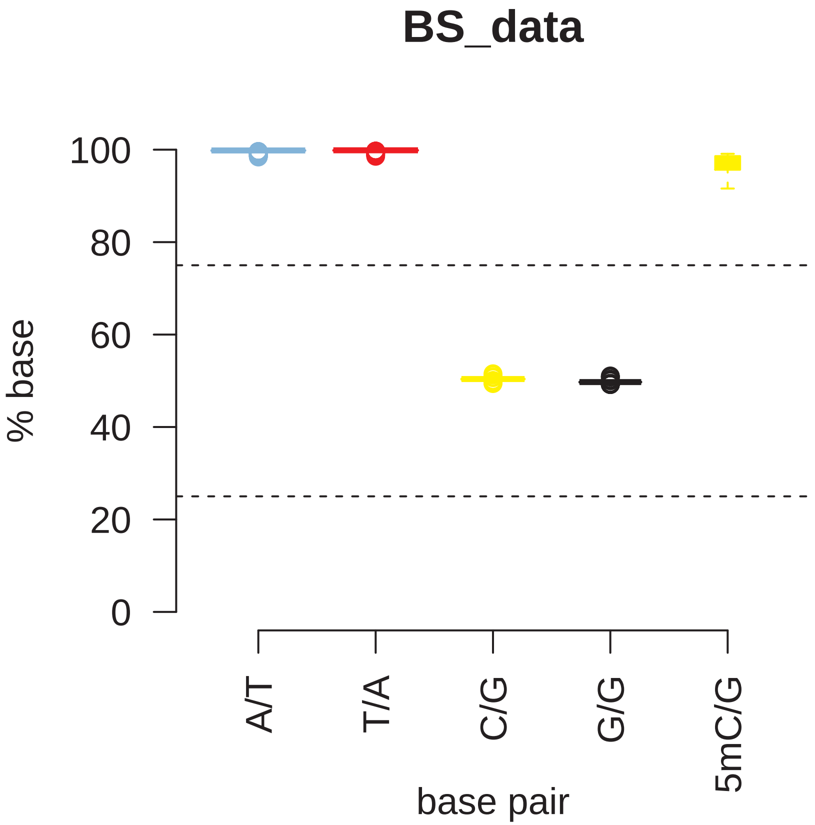

BS_data
100
80
60
40
20
0
% base
A/T
T/A
C/G
G/G
5mC/G
base pair

Ah, distinctly I remember it was in the bleak December;
And each separate dying ember wrought its ghost upon the floor.
Eagerly I wished the morrow; — vainly I had sought to borrow
From my books surcease of sorrow — sorrow for the lost Lenore —
For the rare and radiant maiden whom the angels name Lenore —
Nameless here for evermore.

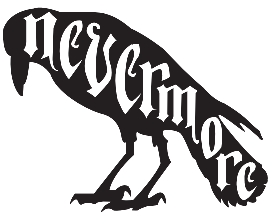

THE RAVEN

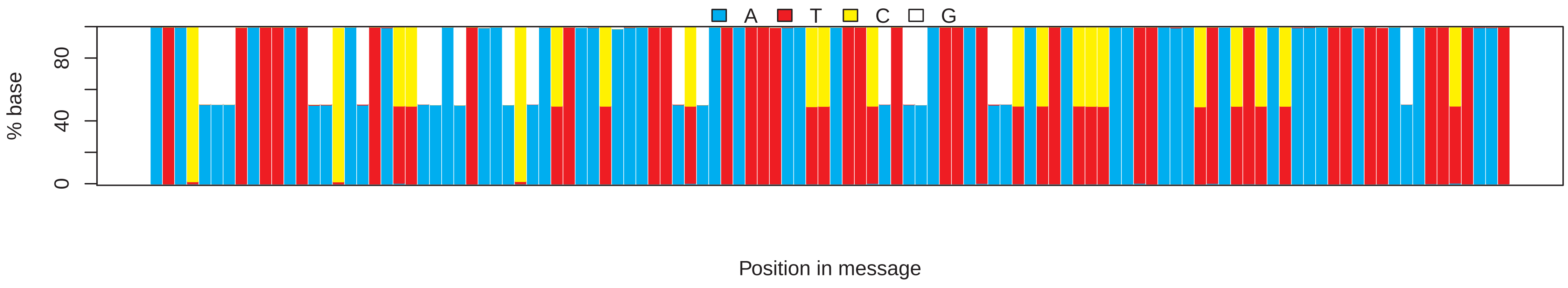

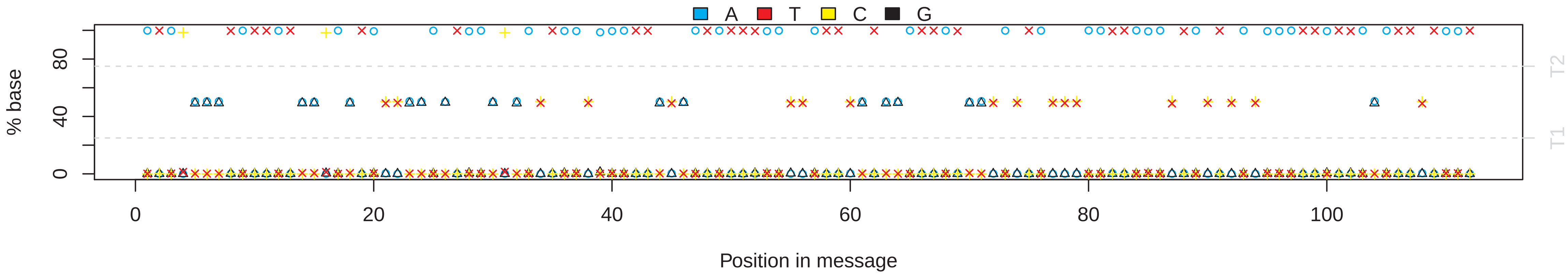

0100000101101000001011000010000001100100011010010111001101110100011010010110111001100011011101000110110001111001

Ah, distinctly

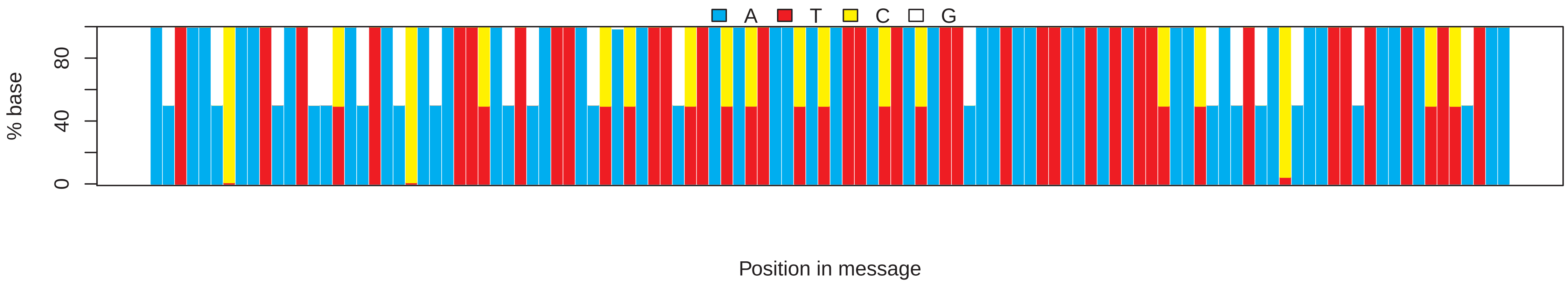

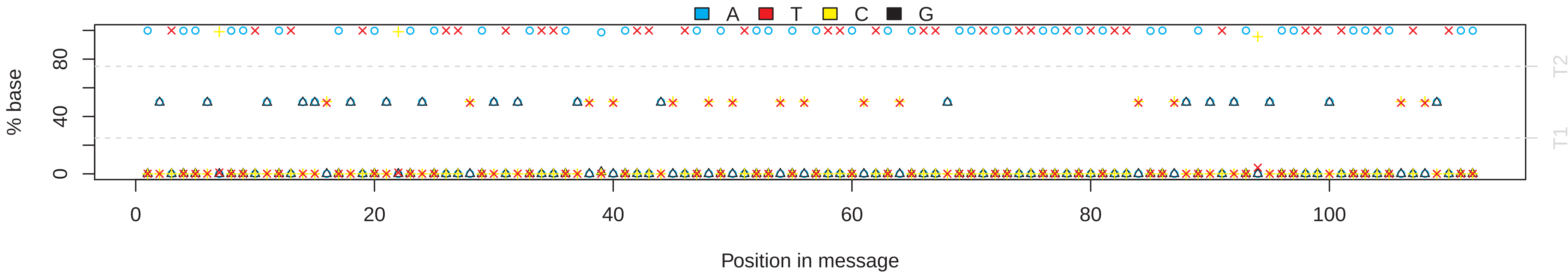

0010000000100100100100000011100100111001010110110101100101011011010110001001100101011100100010000001101001011110100

I remember it

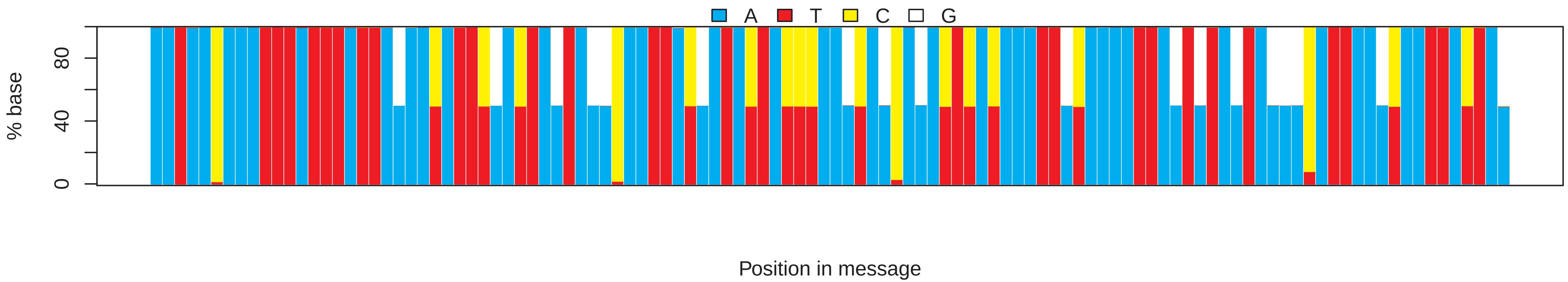

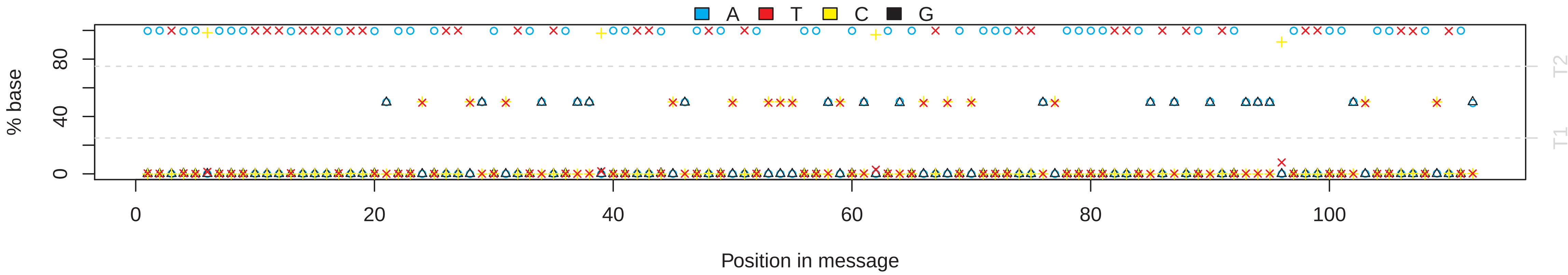

0010000001110111011000010111001100100000011010010110111000100000011101000110100001100101001000000110001001101100

was in the bl

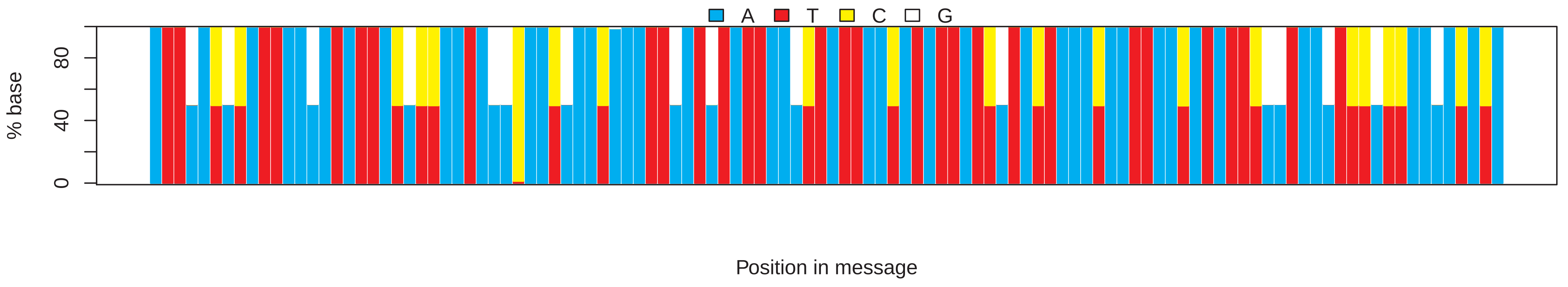

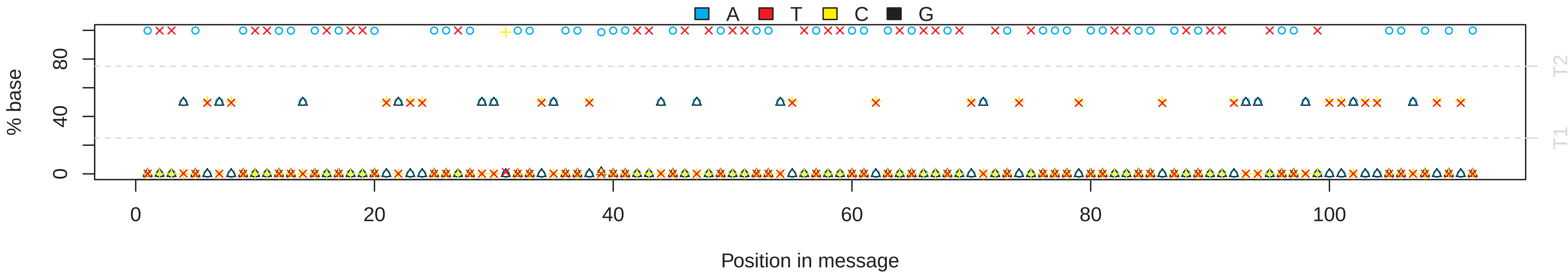

0110010101100001011010110010000001000100011001010110001101100101011011010110001001100101011100100011101100001010

eak December;

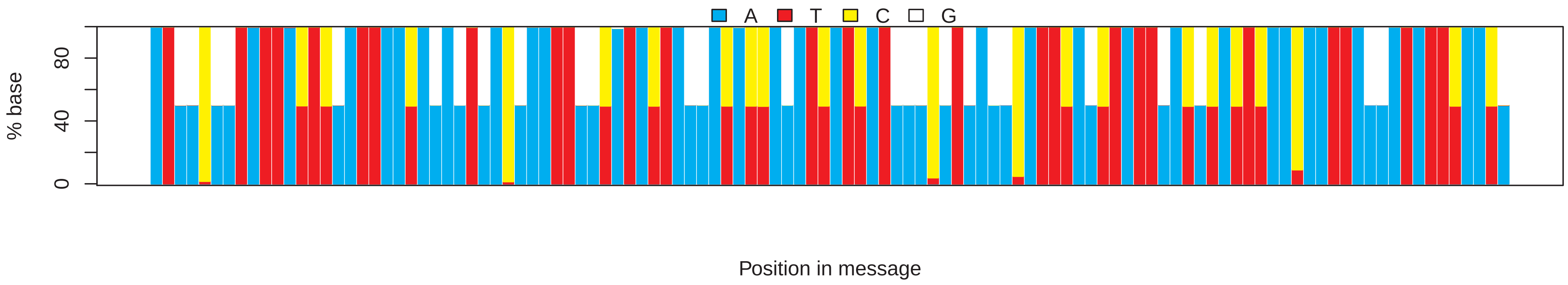

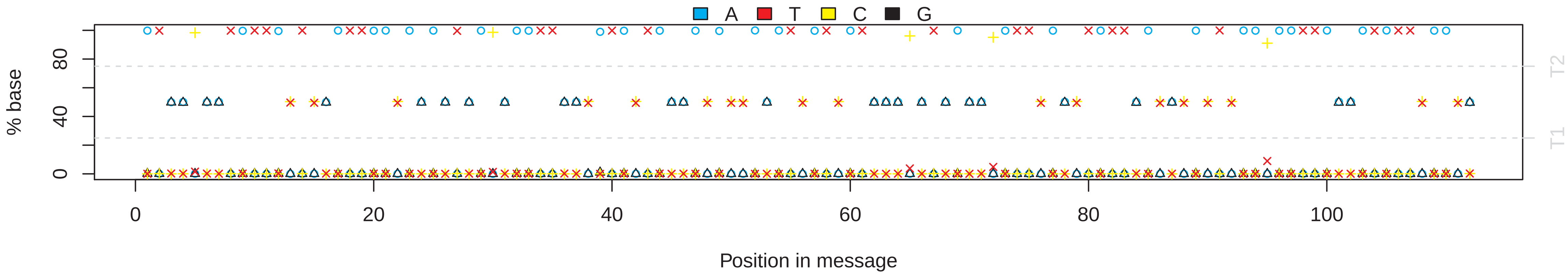

0100000101101110011001000010000001100101011000010110001101101000001000000111001101100101011100000110000101110010

And each separ

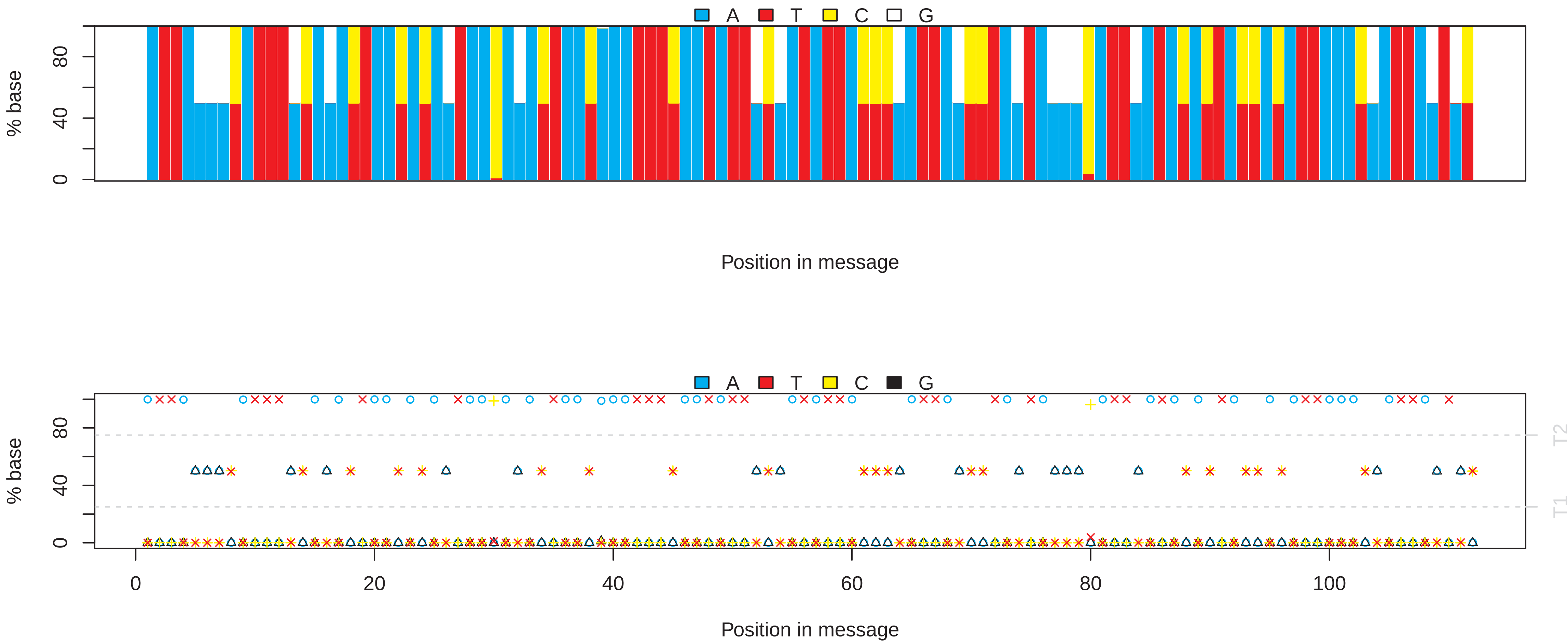

0110000101110100011001010010000001100100011110010110100101101110011001110010000001100101011011010110001001100101

ate dying embe

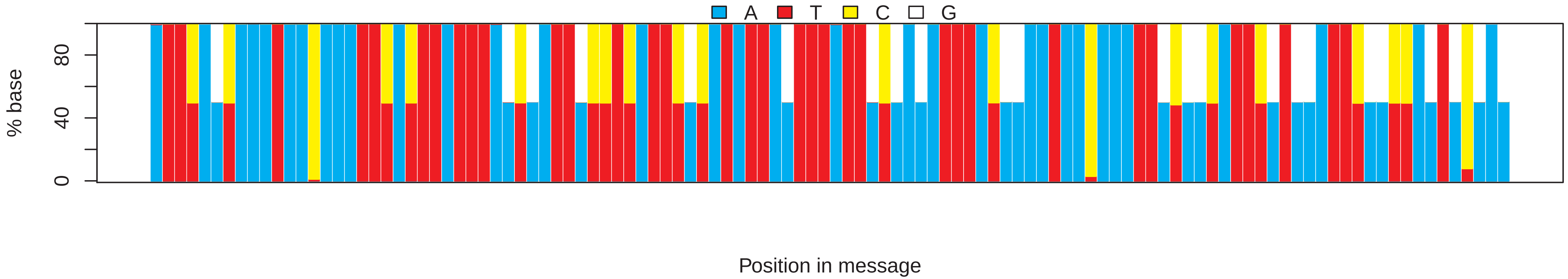

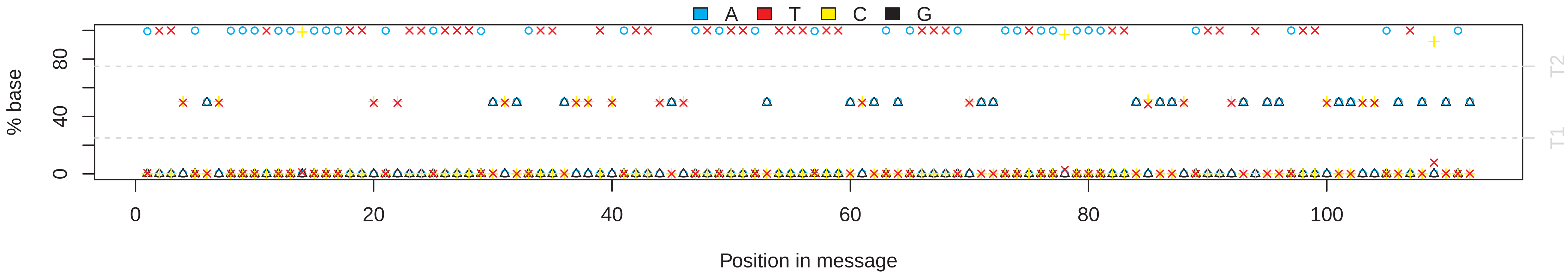

0111001000100000011101110111001001101111011101010110011101101000011101000010000001101001011101000111001100100000

r wrought its

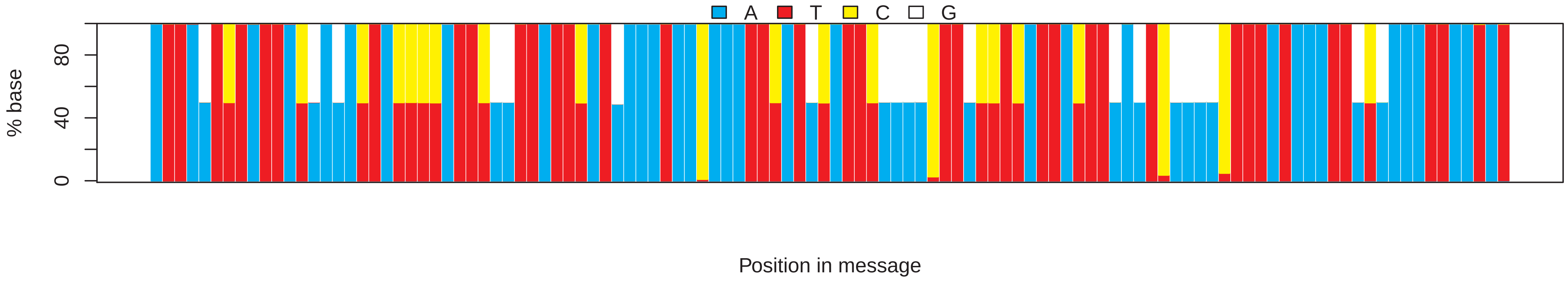

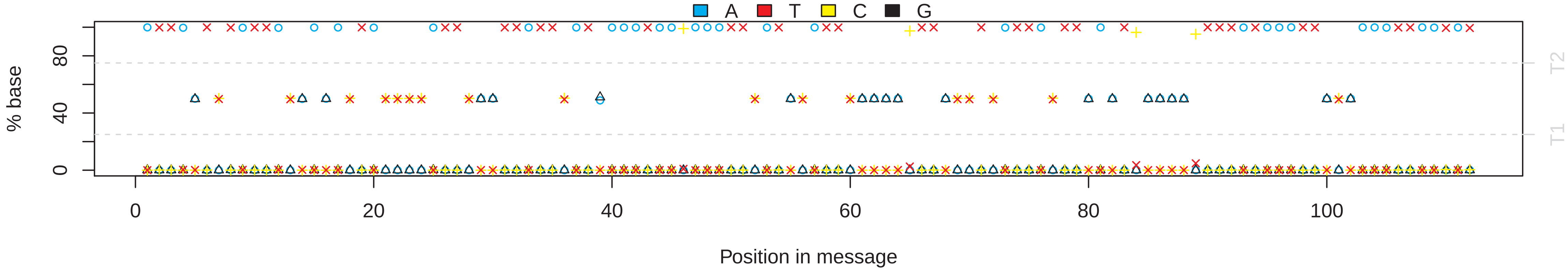

0110011101101000011011110111001101110100001000000111010101110000011011110110111000100000011101000110100001100101

ghost upon the

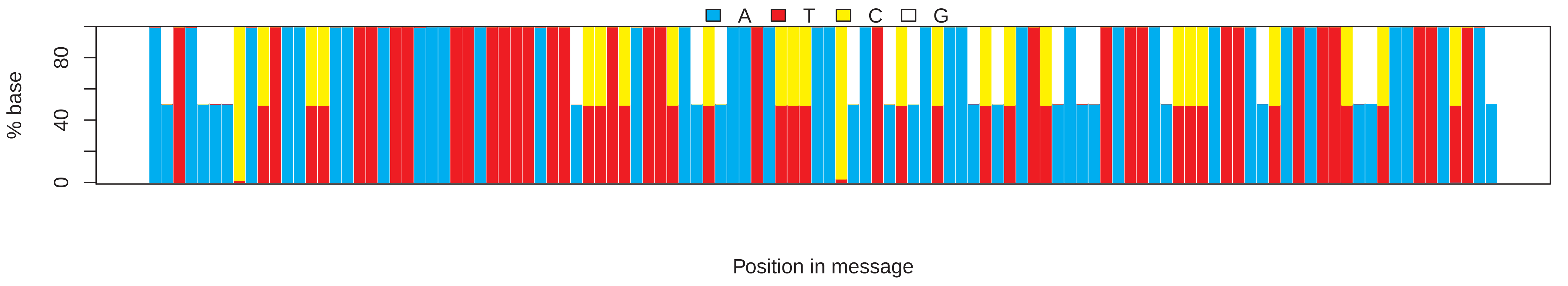

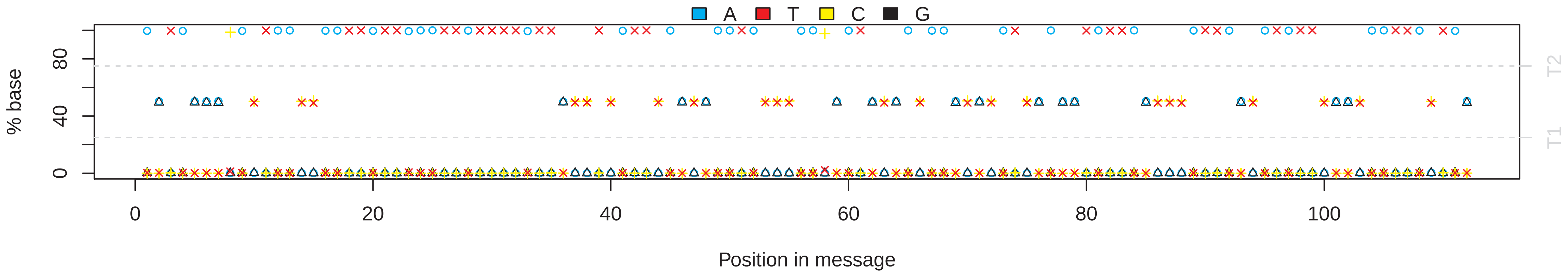

0010000001100110011011000110111101101111011100100010111000001010010001010110000101100111011001010111001001101100

floor.
Eagerl

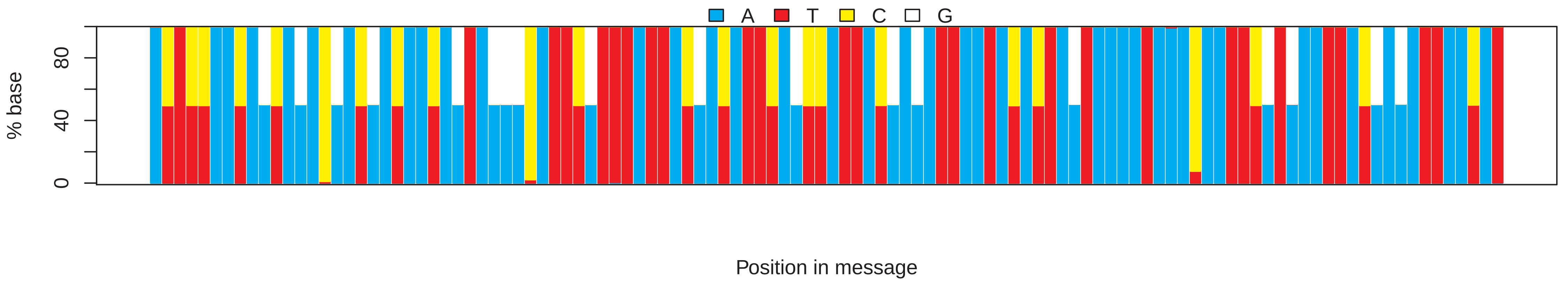

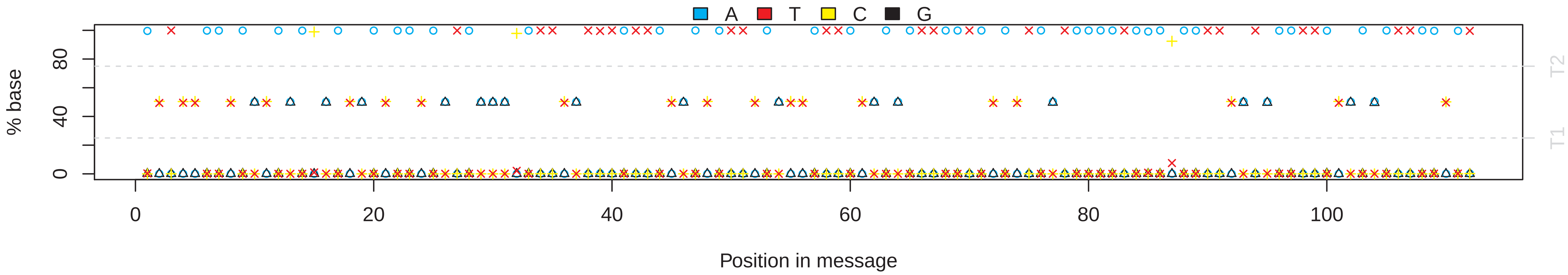

0111100100100000010010010010000001110111011010010111001101101000011001010110010000100000011101000110100001100101

y I wished the

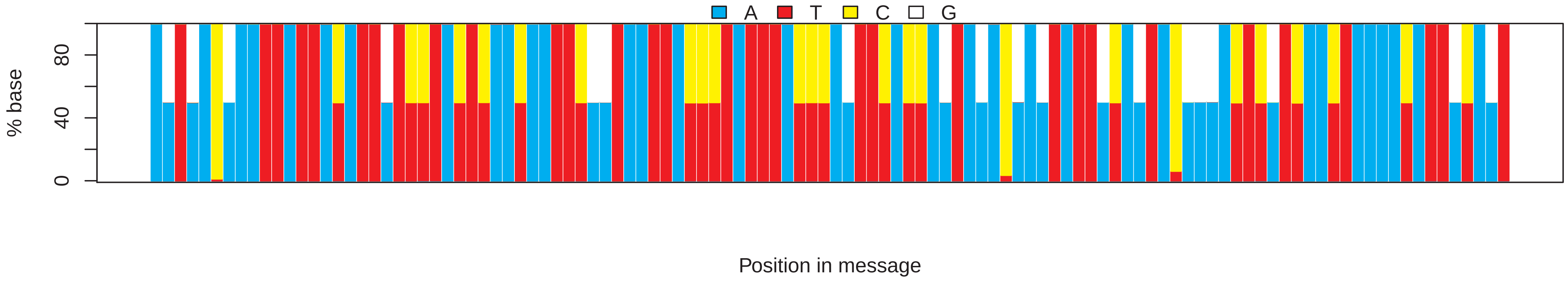

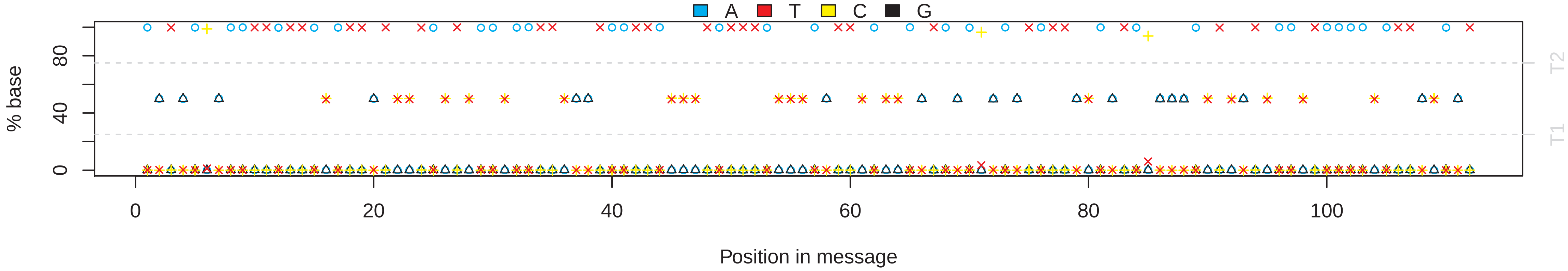

0010000001101101011011110111001001110010011011110111011100111011001000000010110100100000011101100110000101101001

morrow; − vai

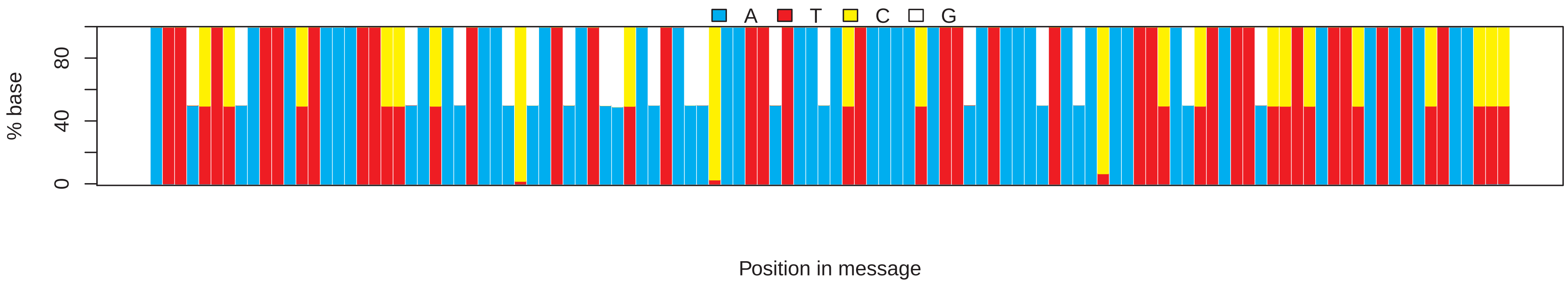

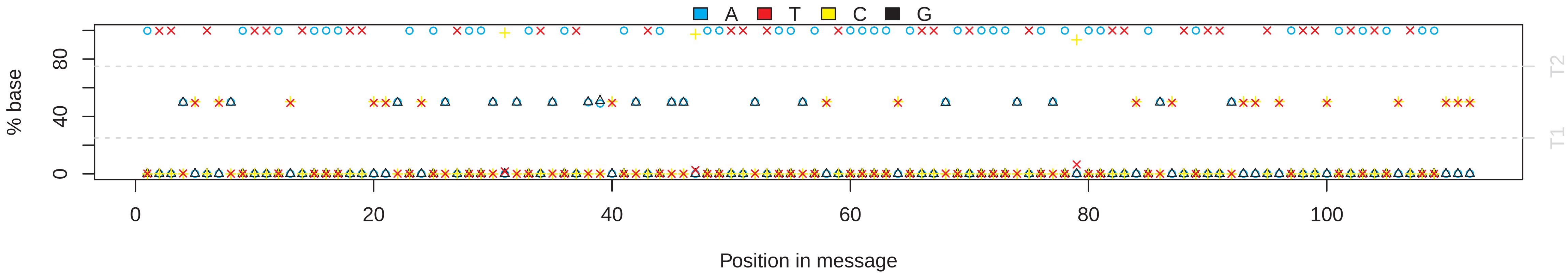

0110111001101100011110010010000001001001001000000110100001100001011001000010000001110011011011110111010101100111

nly I had soug

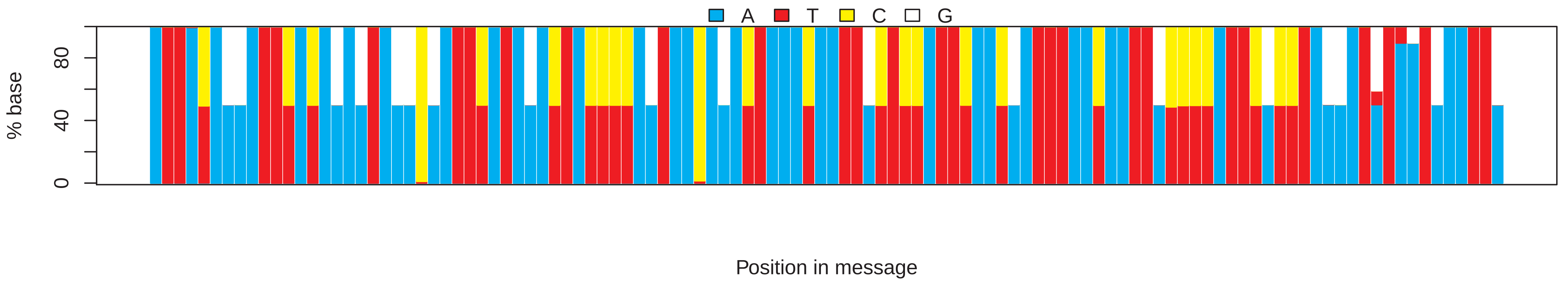

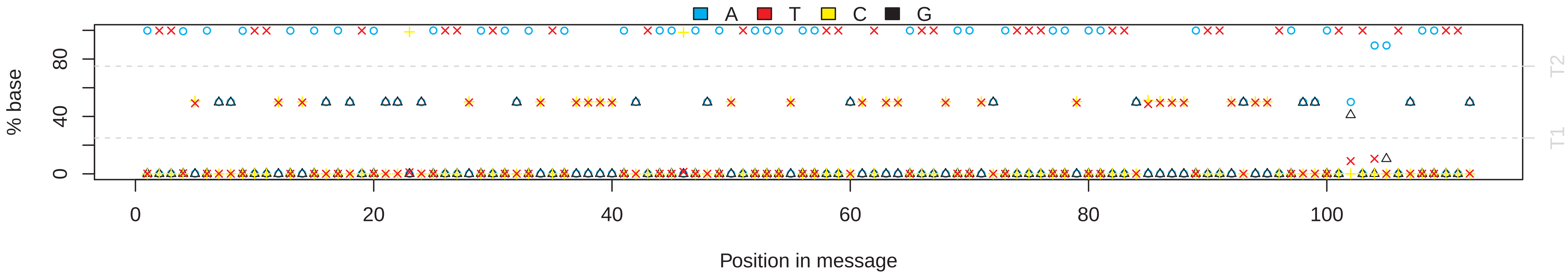

0110100001110100001000000111010001101111001000000110001001101111011100100111001001101111011101110000101001000110

ht to borrow
F

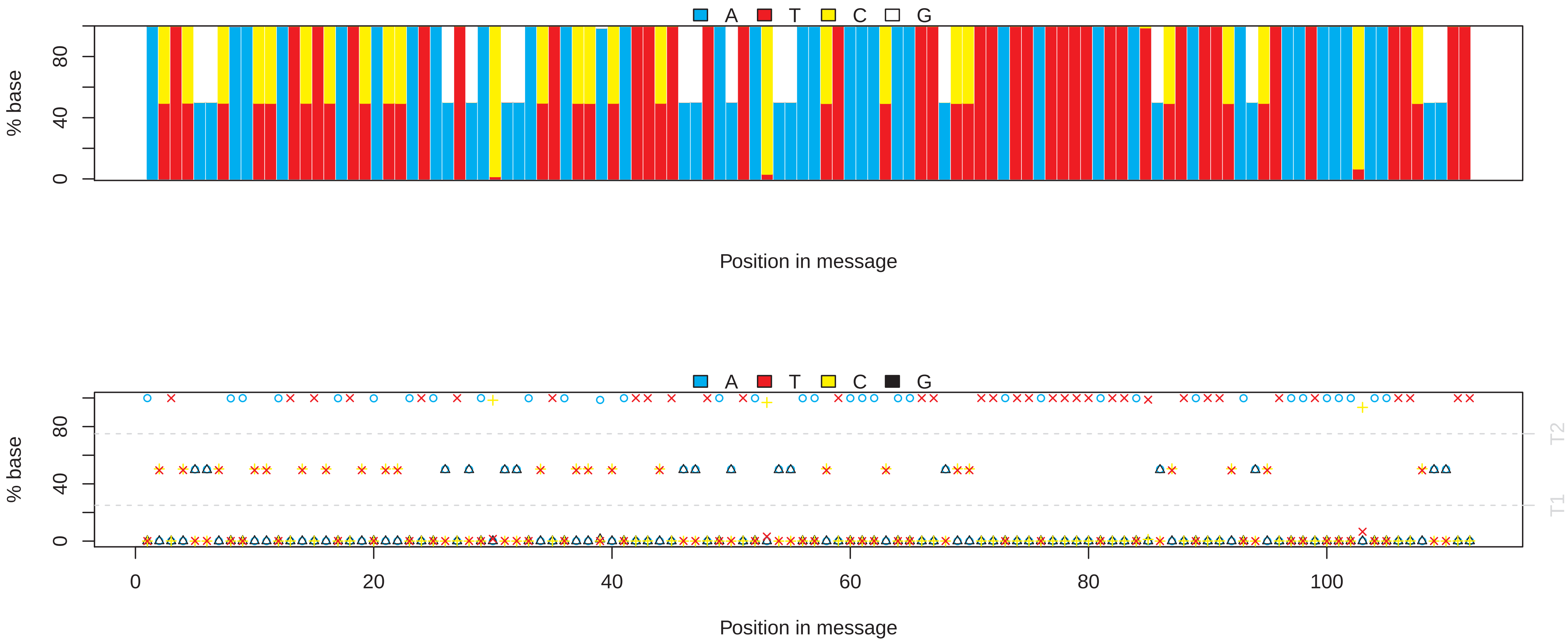

0111001001101111011011010010000001101101011110010010000001100010011011110110111101101011011100110010000001110011

rom my books s

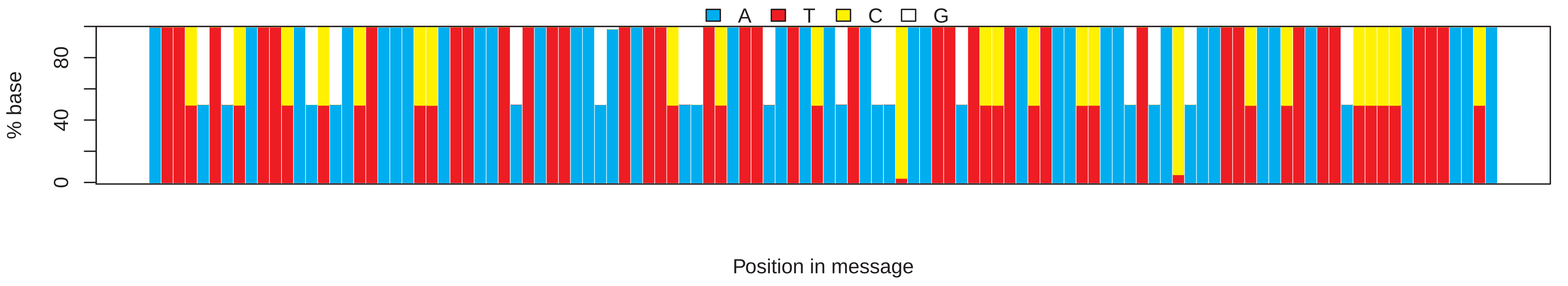

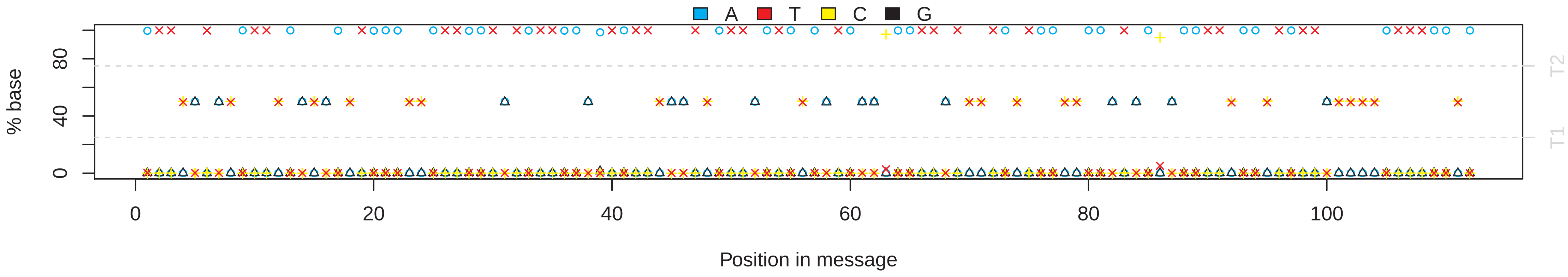

0111010101110010011000110110010101100001011100110110010100100000011011110110011000100000011100110110111101110010

urcease of sor

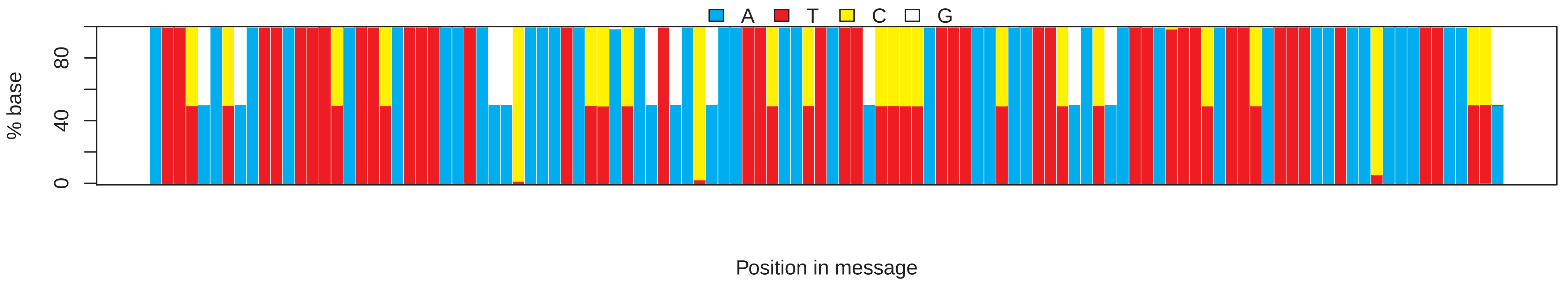

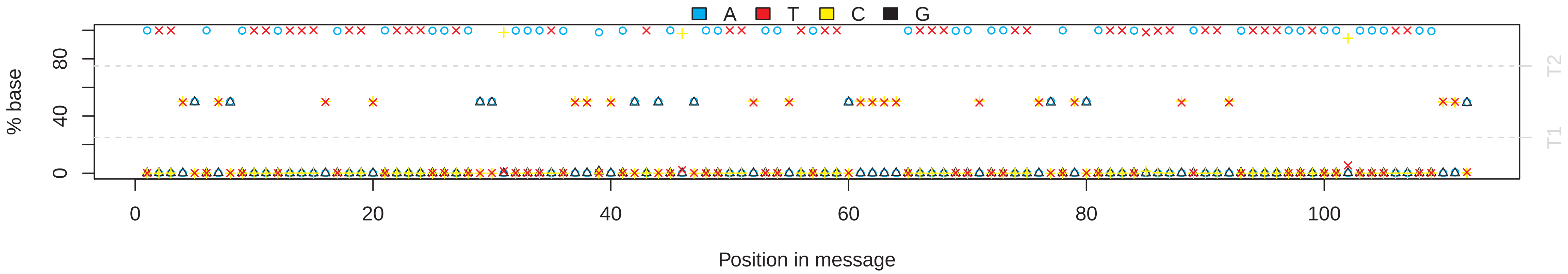

0111001001101111011101110010000000101101001000000111001101101111011100100111001001101111011101110010000001100110

row − sorrow f

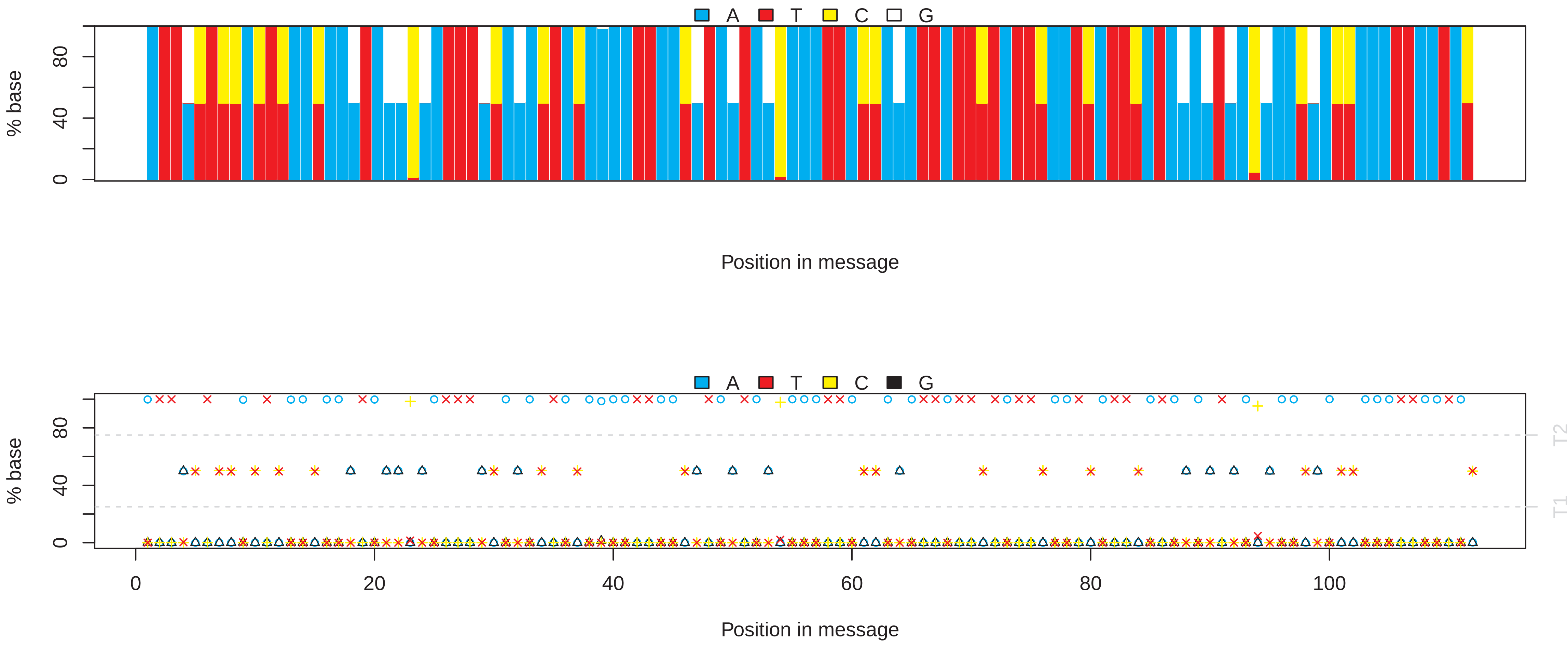

0110111101110010001000000111010001101000011001010010000001101100011011110111001101110100001000000100110001100101

or the lost Le

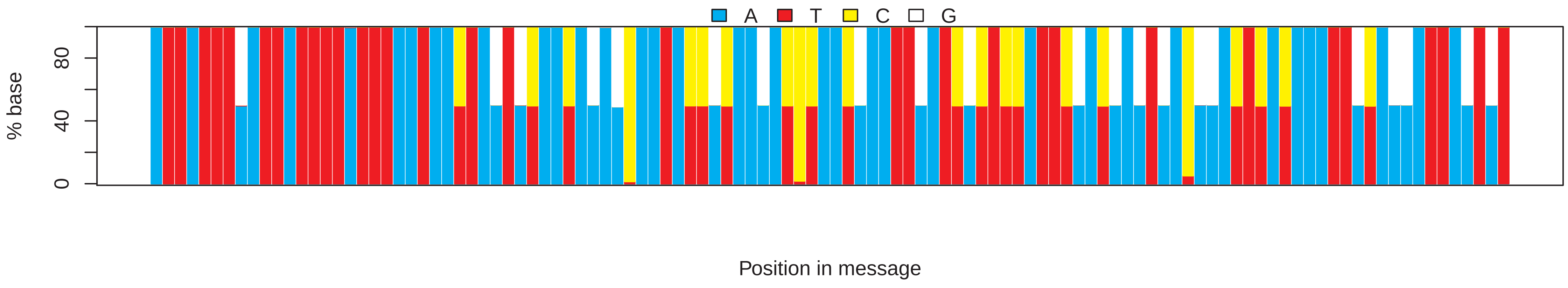

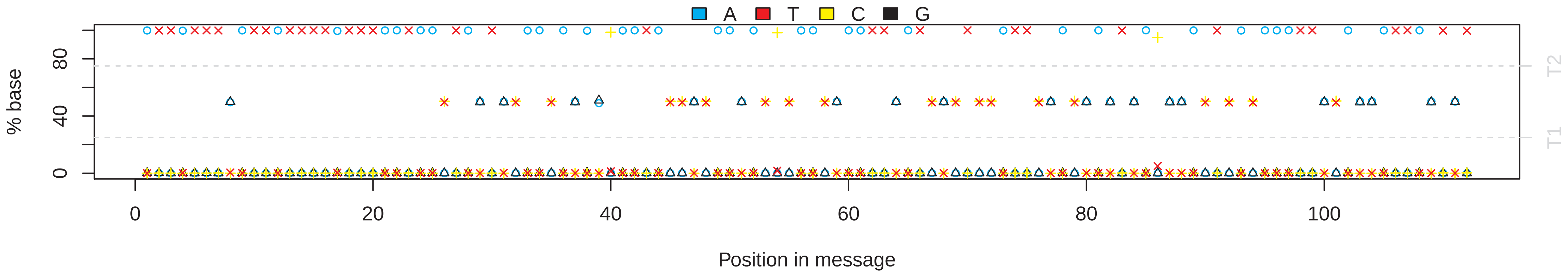

0110111001101111011100100110010100100000001011010000101001000110011011110111001000100000011101000110100001100101

nore −
For the

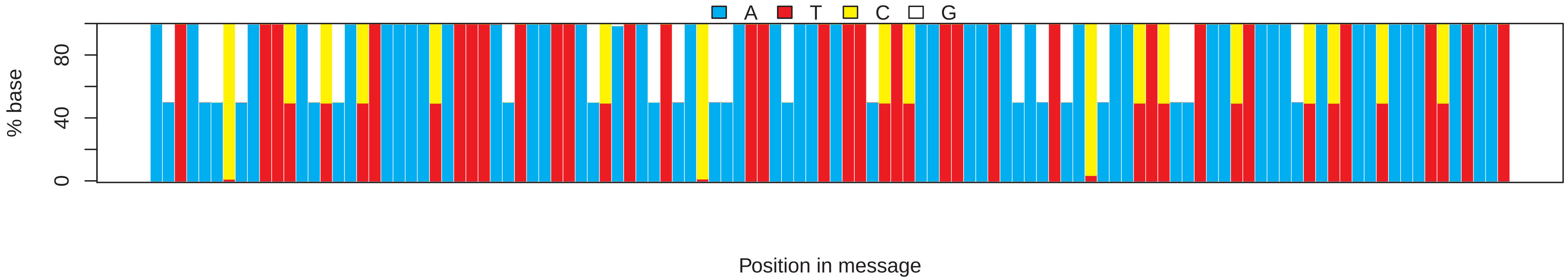

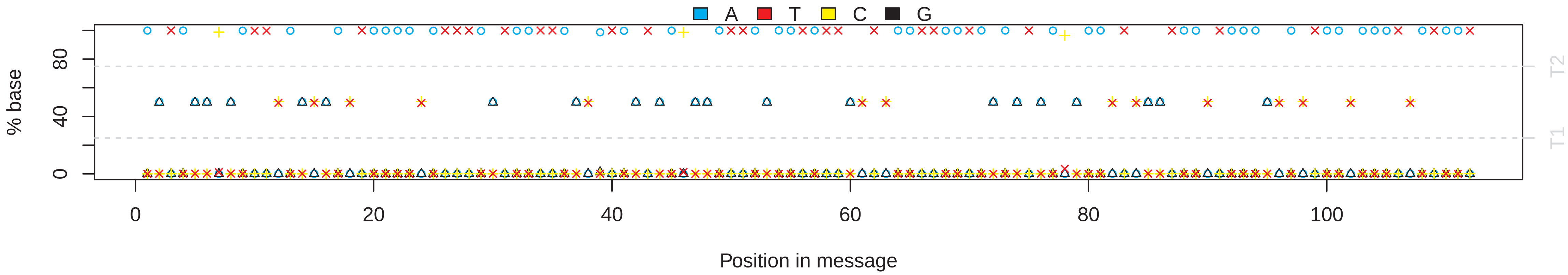

0010000001110010011000010111001001100101001000000110000101101110011001000010000001110010011000010110010001101001

rare and radi

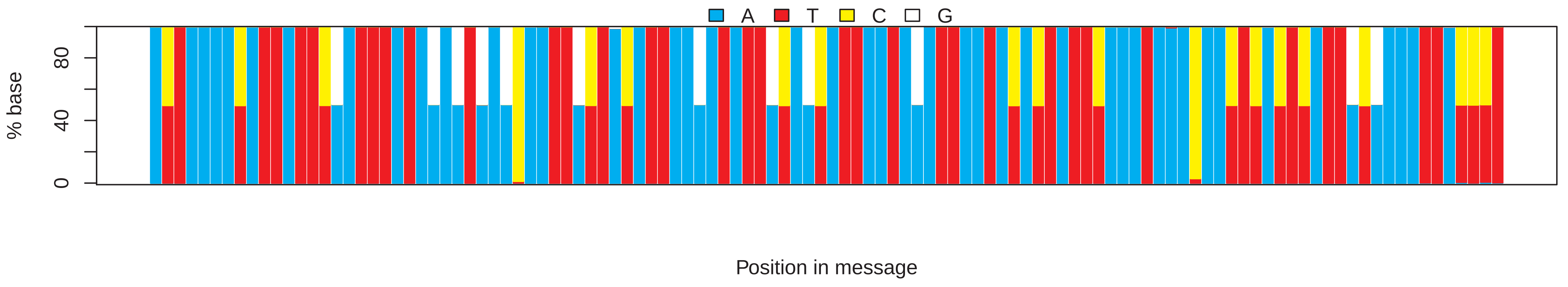

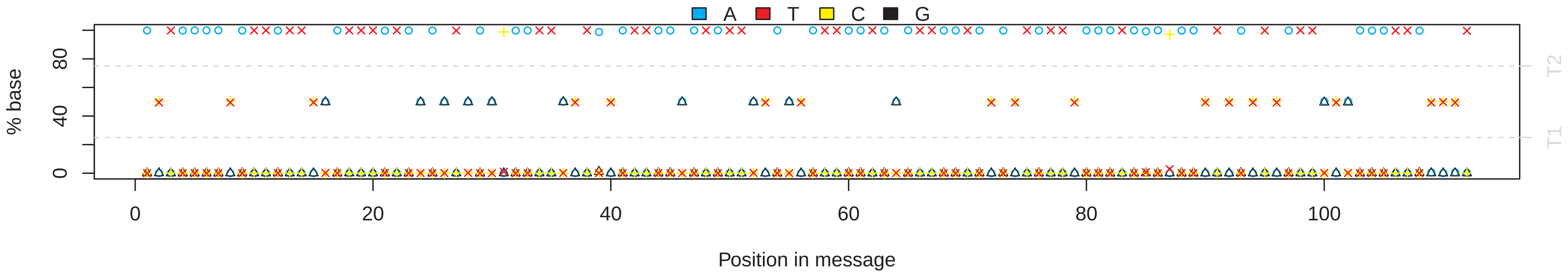

0110000101101110011101000010000001101101011000010110100101100100011001010110111000100000011101110110100001101111

ant maiden who

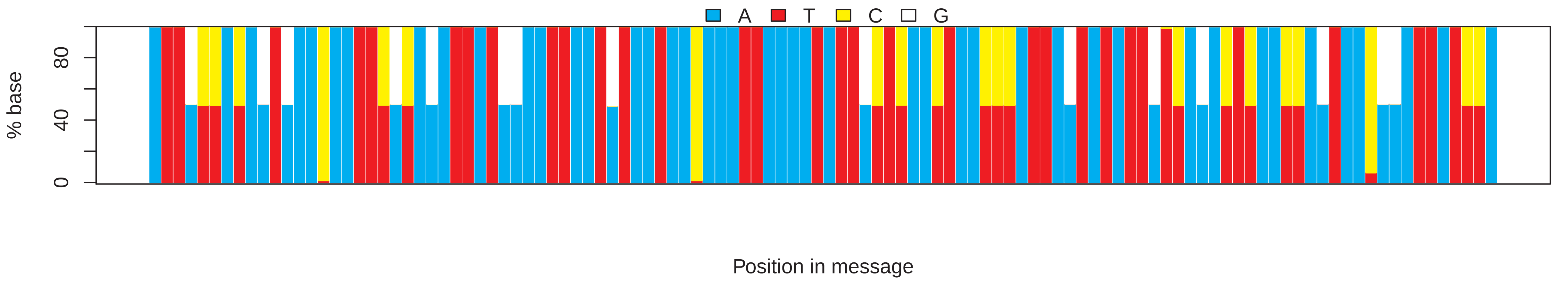

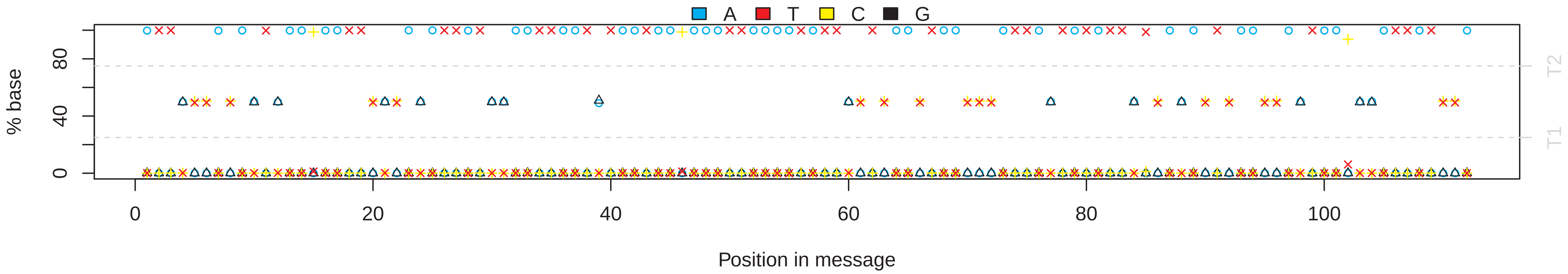

0110110100100000011101000110100001100101001000000110000101101110011001110110010101101100011100110010000001101110

m the angels n

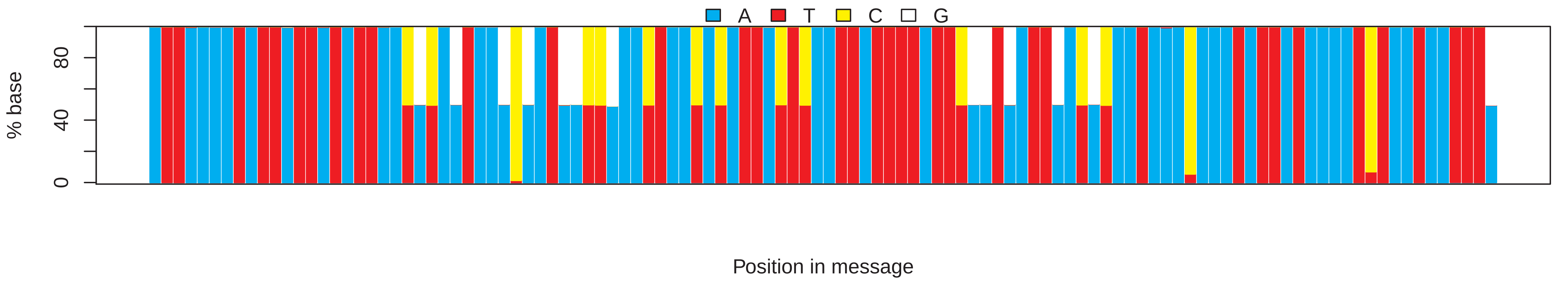

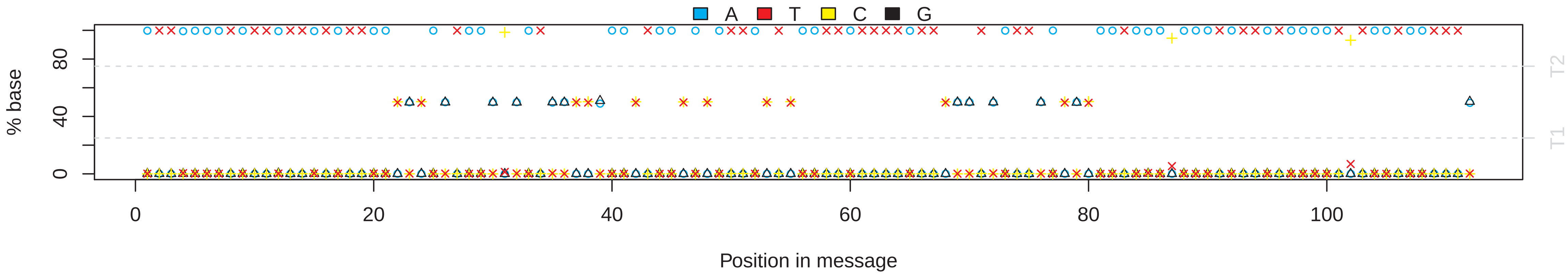

0110000101101101011001010010000001001100011001010110111001101111011100100110010100100000001011010000010100100111 0

ame Lenore −
N

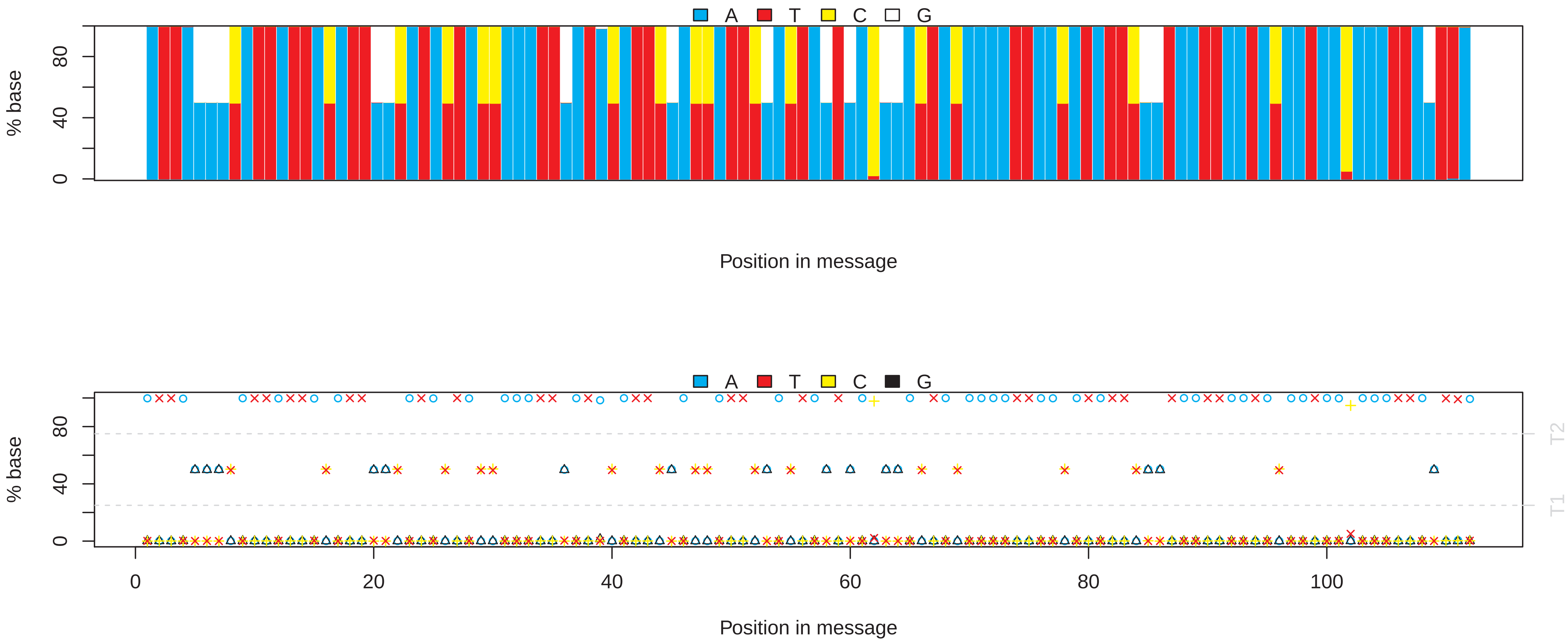

0110000101101101011001010110110001100101011100110111001100100000011010000110010101110010011001010010000001100110

ameless here f

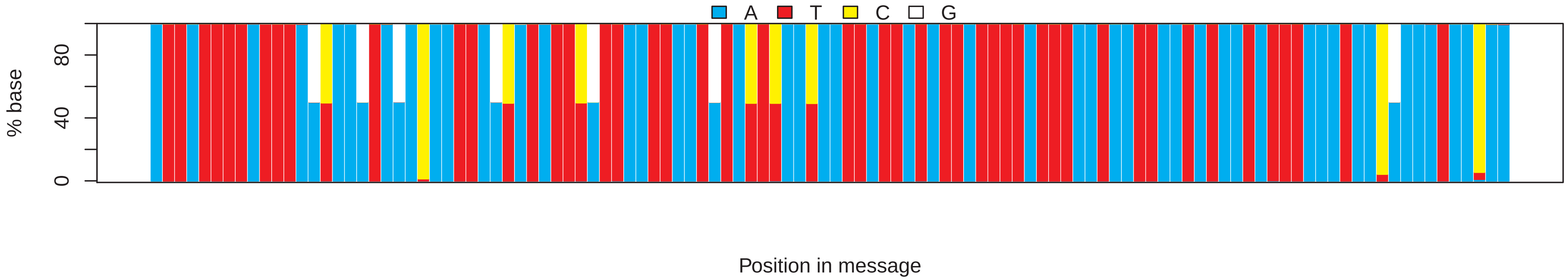

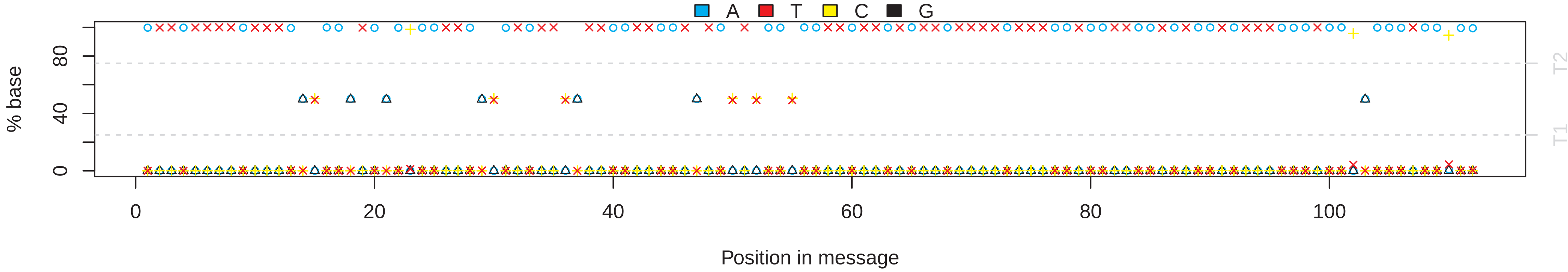

0110111101110010001000000110010101110110011001010111001001101101011011110111001001100101001011100010000000100000

or evermore.

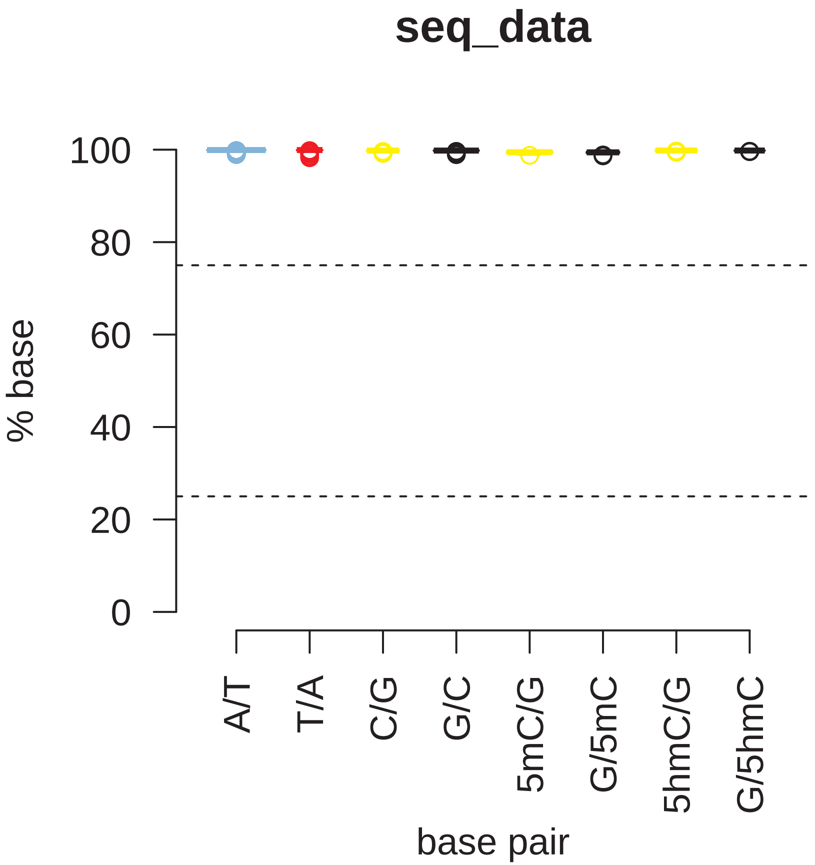

seq_data
% base
100
80
60
40
20
0
A/T
T/A
C/G
G/C
5mC/G
G/5mC
5hmC/G
G/5hmC
base pair

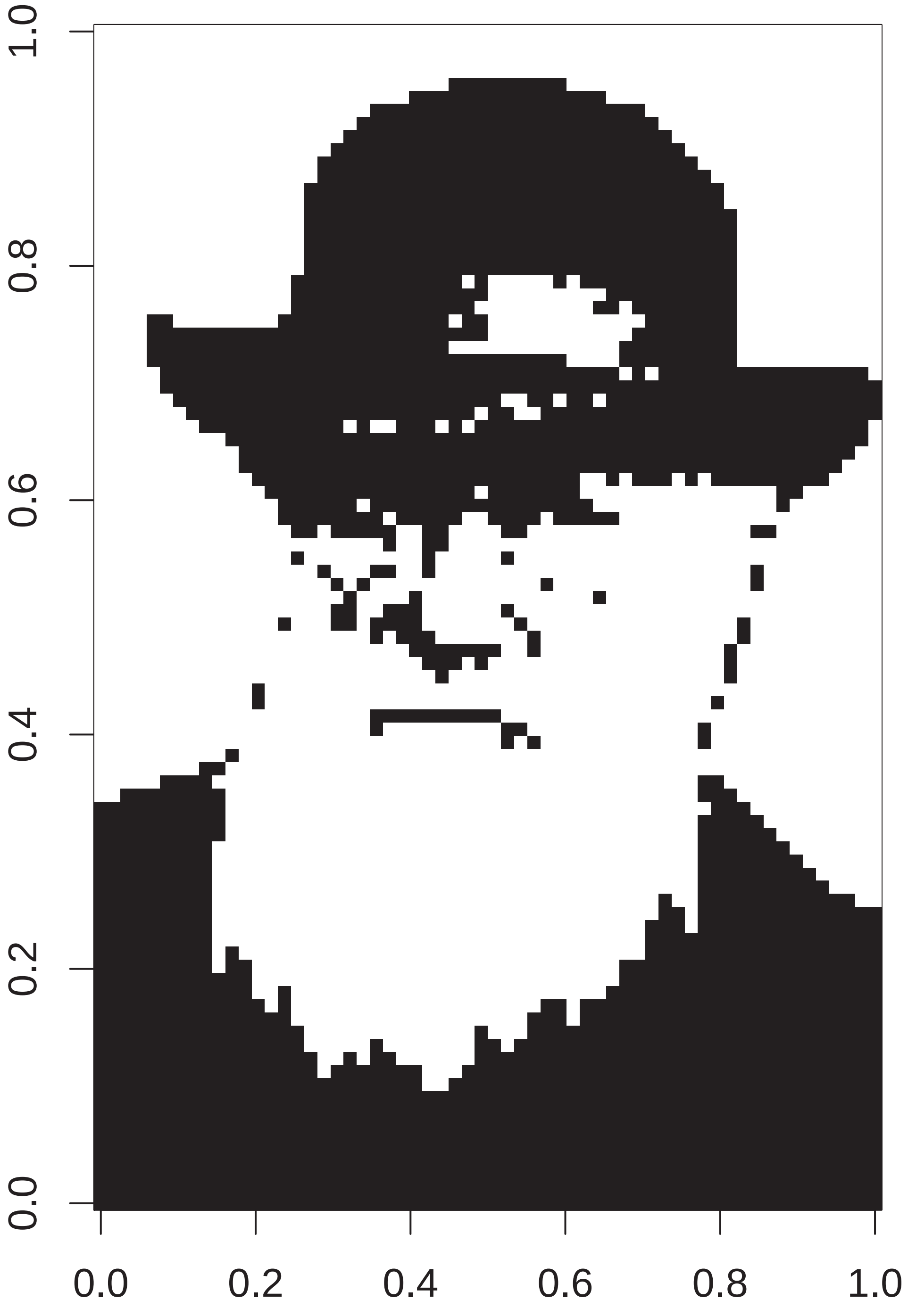

1.0
0.8
0.6
0.4
0.2
0.0
0.0
0.2
0.4
0.6
0.8
1.0

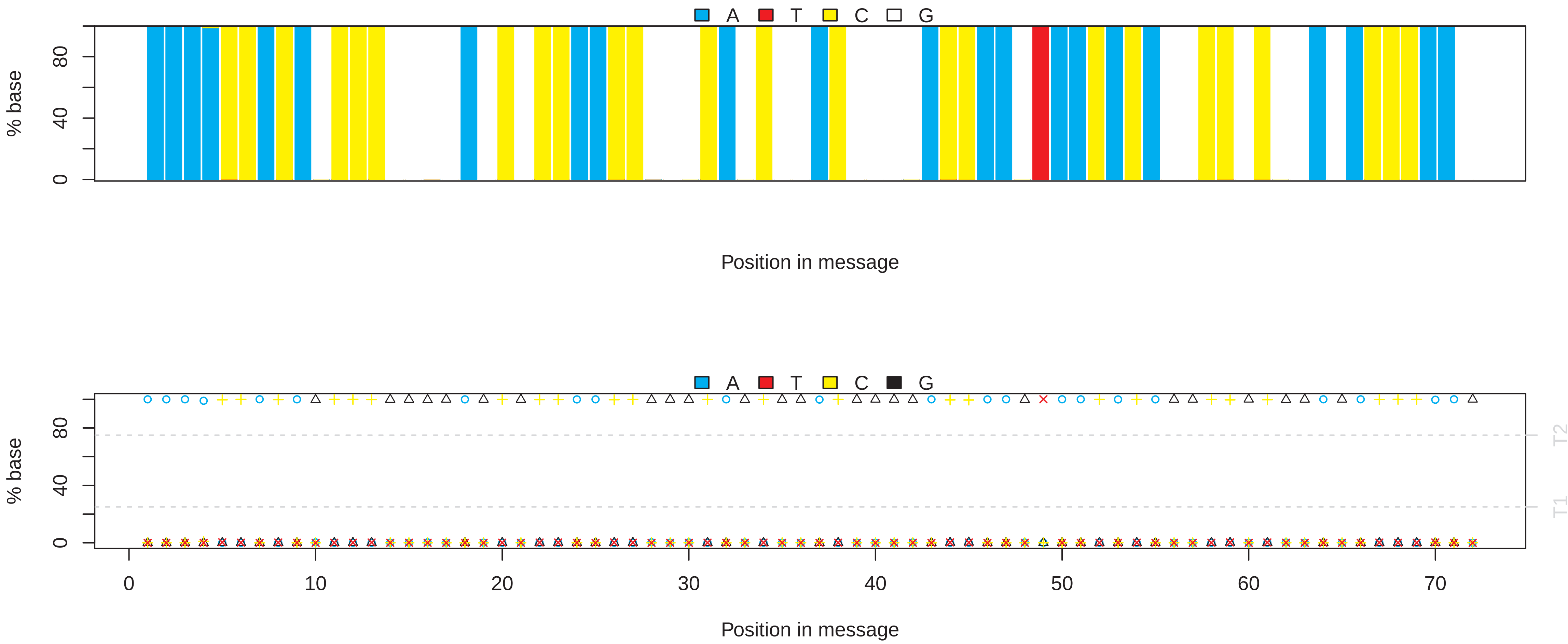

000000000100011110101000000111001011001111000001100000011001011010000001

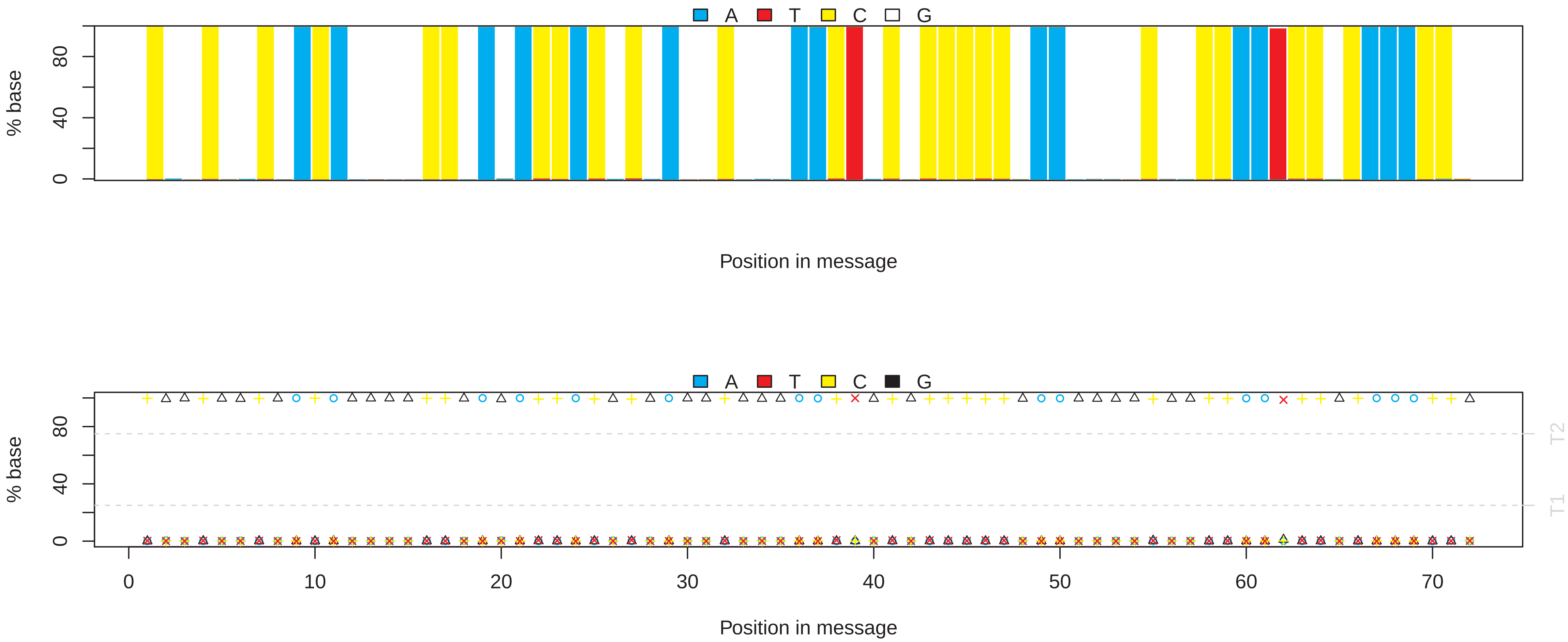

011011010001111001010000010101101110001101000001001111011000010010000001

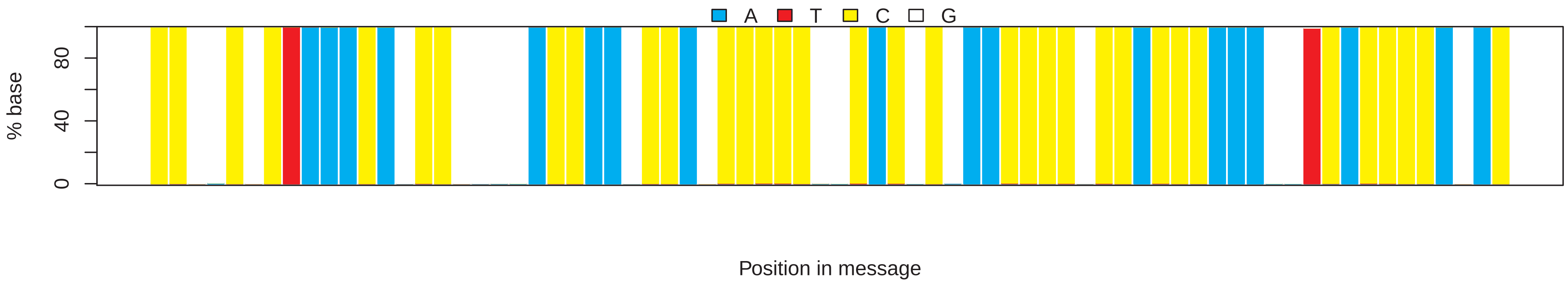

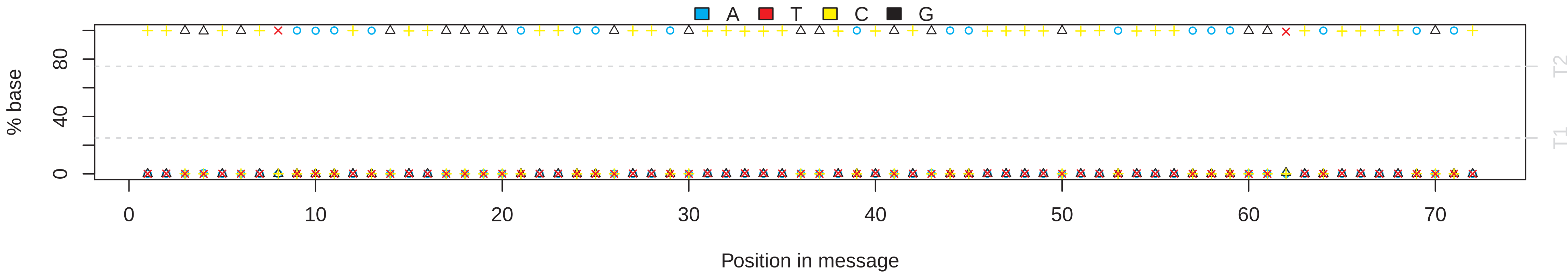

001101010000010011110000010001000001100010100000010000000001110000000100

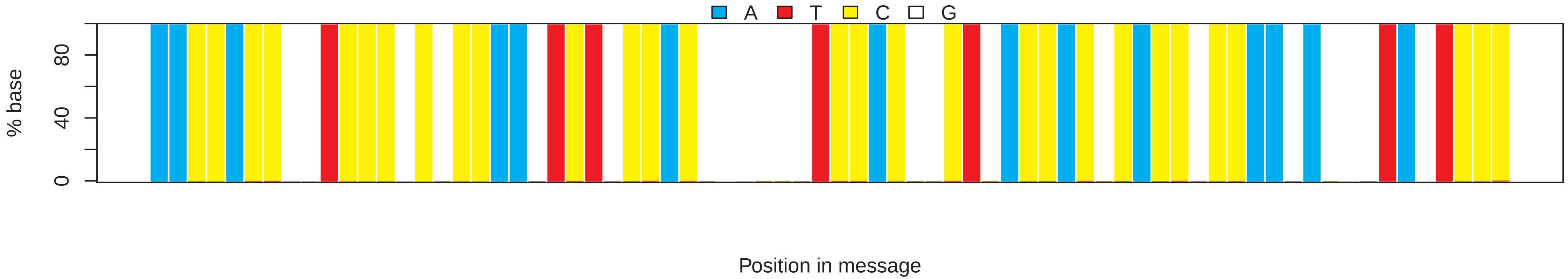

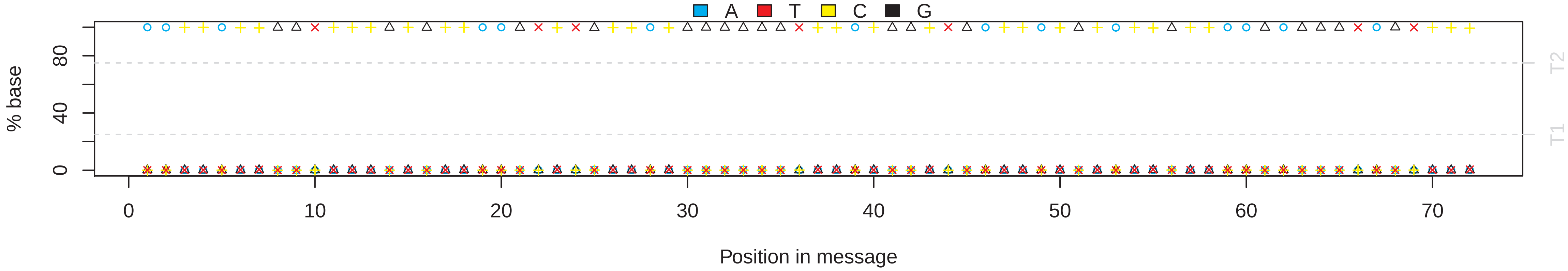

000000011100010100001101100001111111000011011000001000010000101111011000

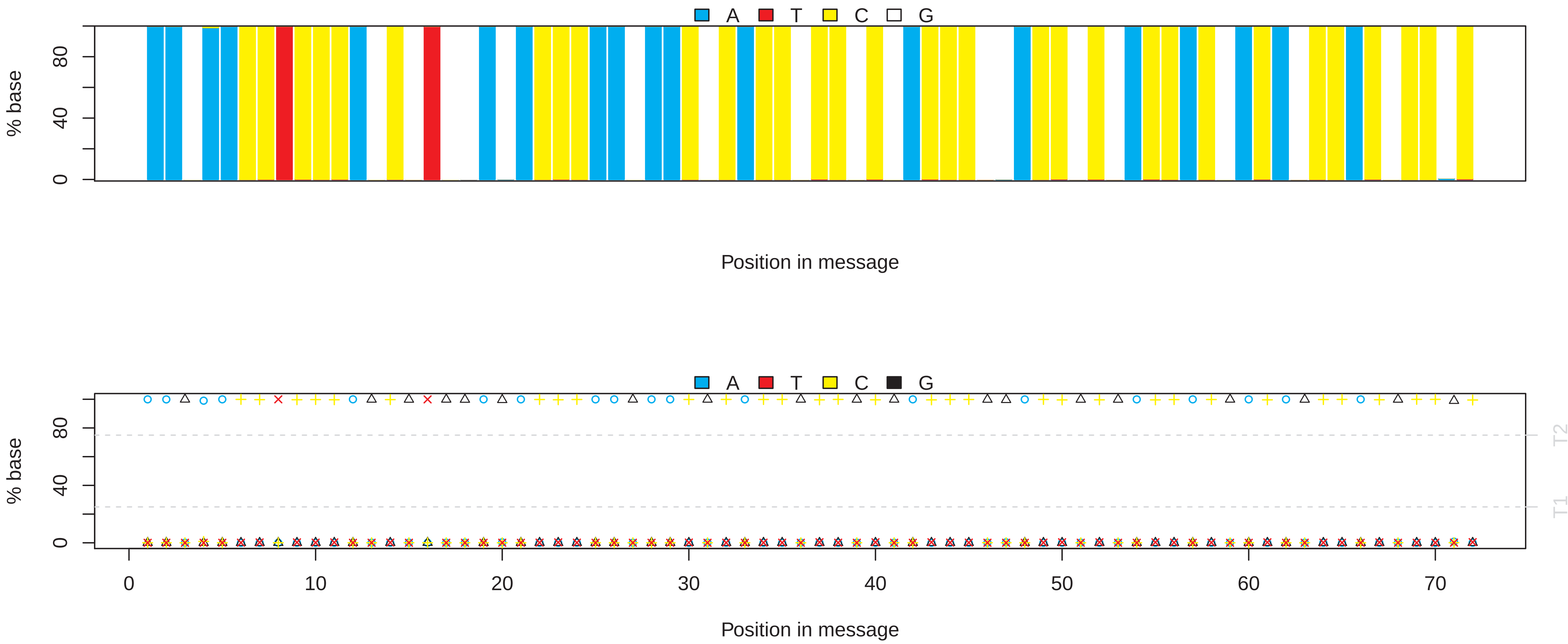

001000010000101111010000001000100001001010000110001010000010001000010010

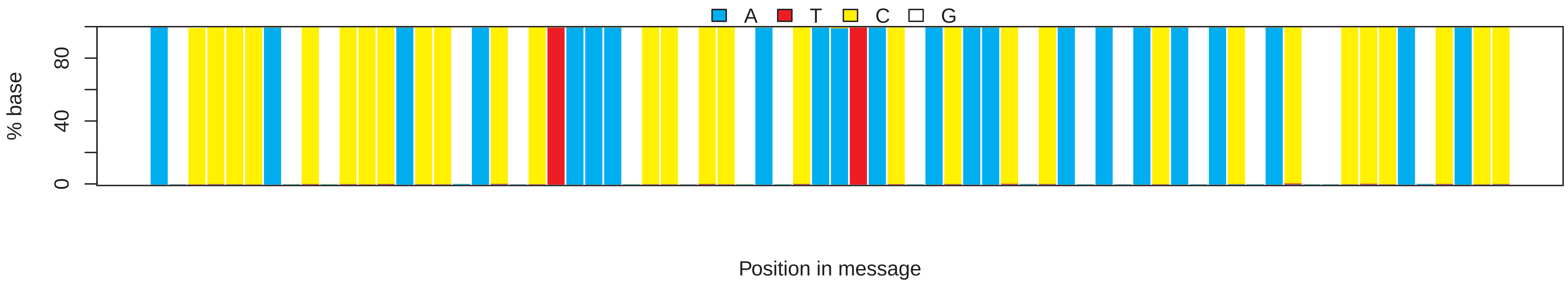

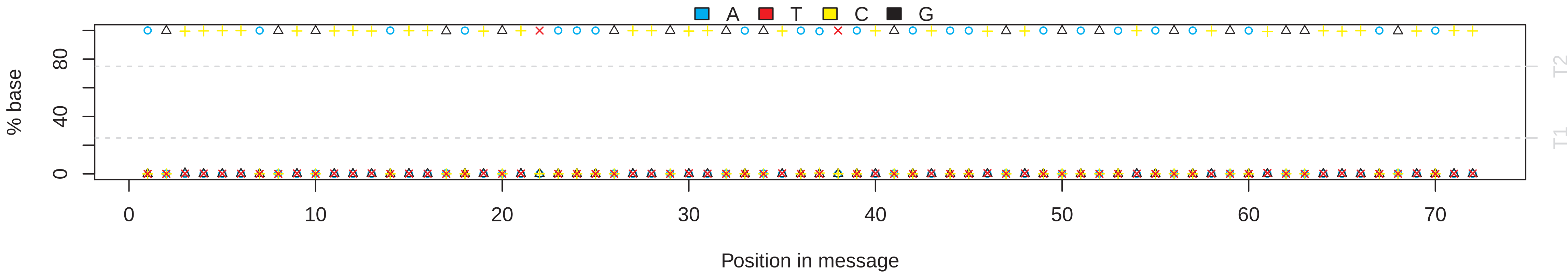

0100000101000000100101000100100101000100100000100101000100100110000010000

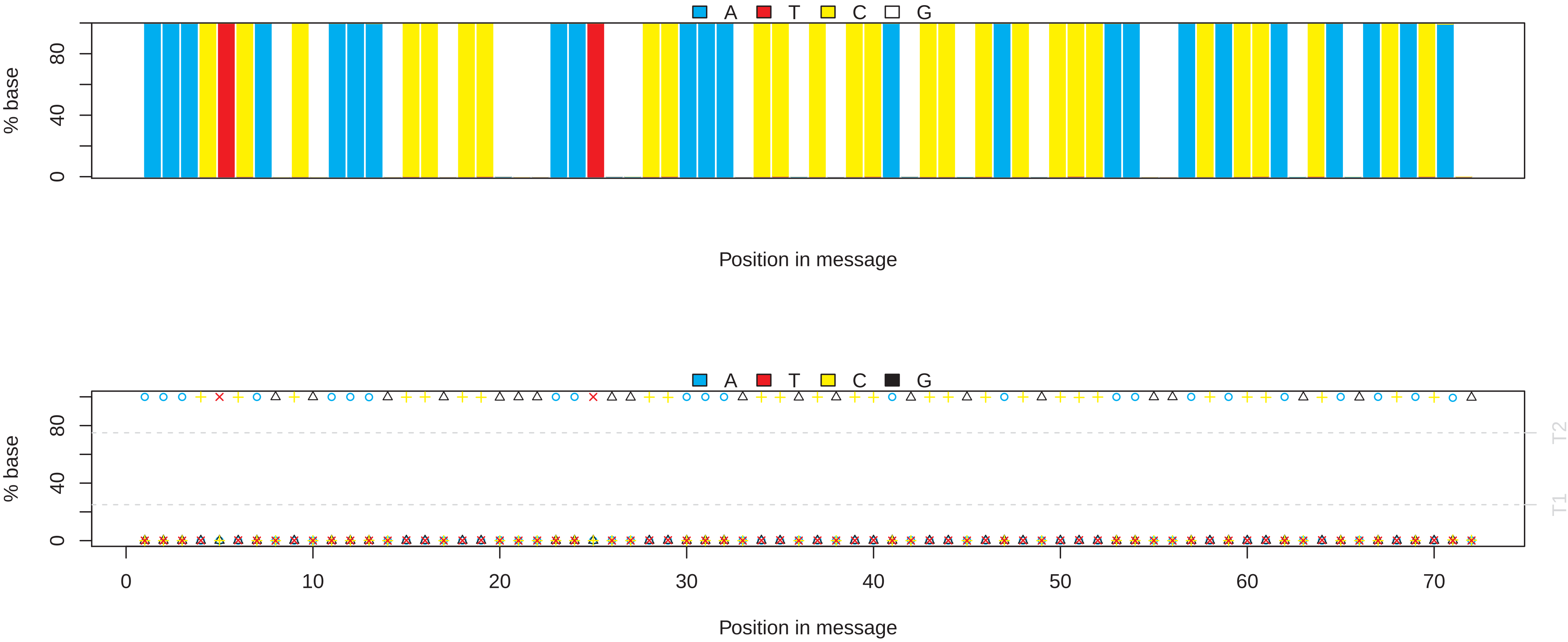

000010010100010010011100111000001001010001001000100000110000001001000001

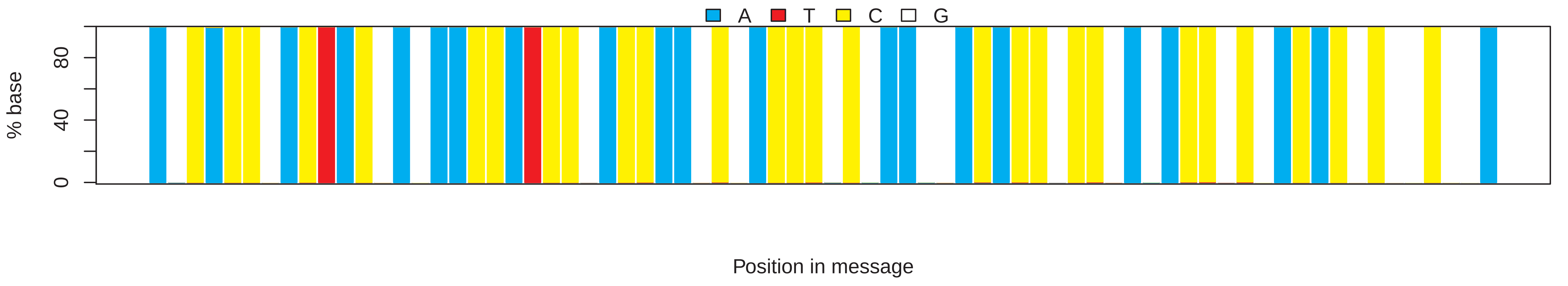

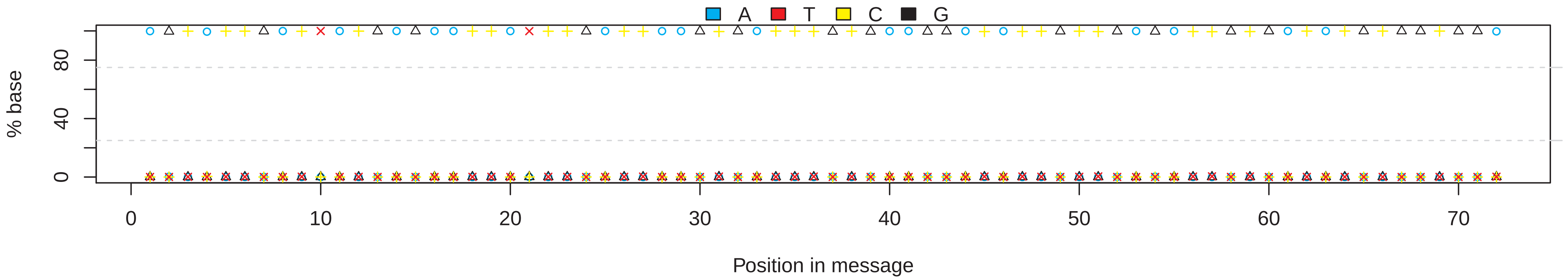

01000010010010100000100100000101000010100110000010010100010100001010110110

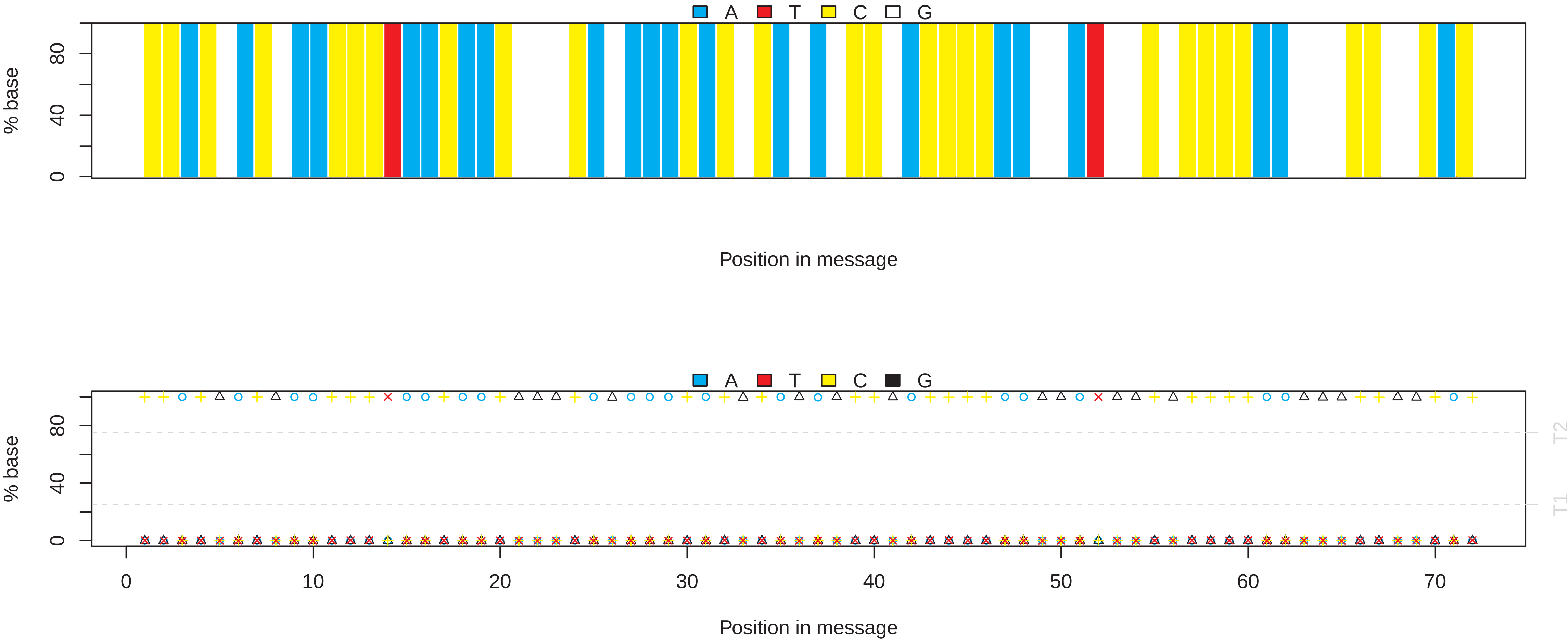

0000100100000100000011100100000010010100100000001101110100000001110011000

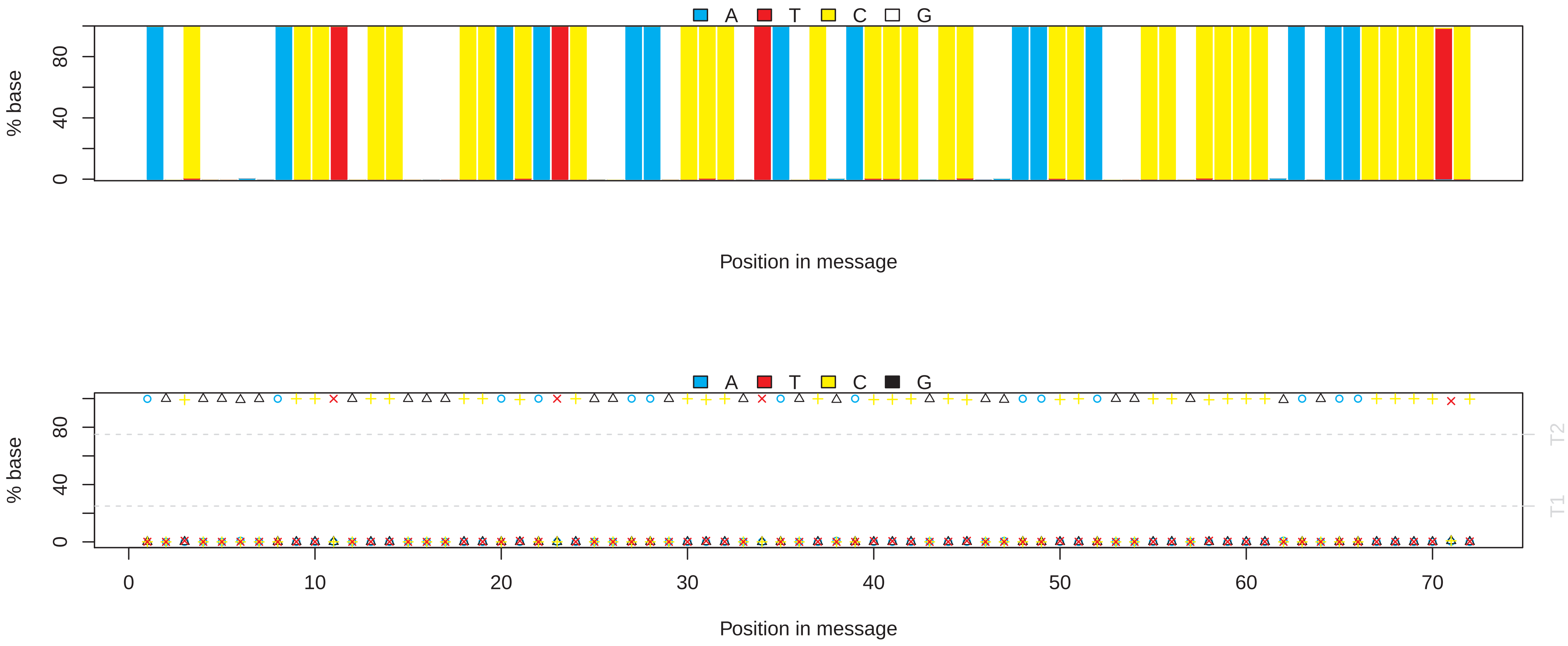

010111100011001110000010110010001101010000100110000011001000010100000010

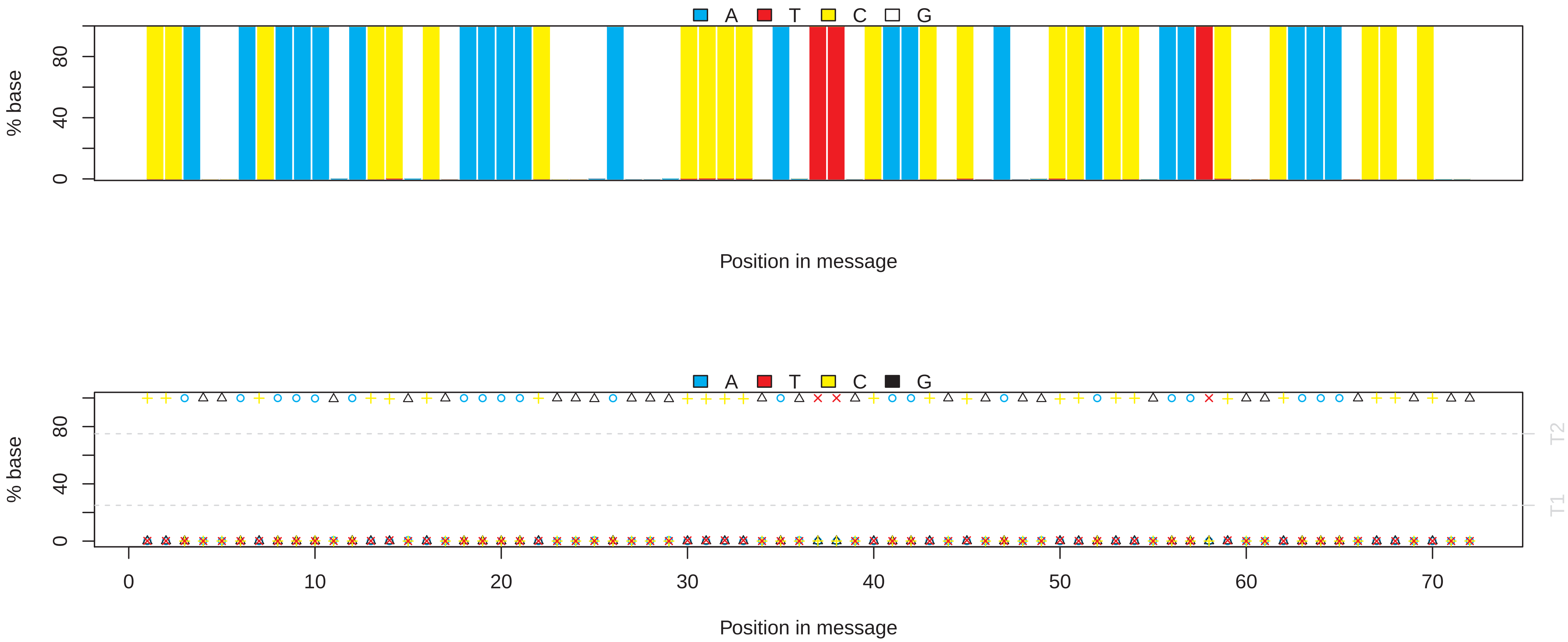

0001100000010001010000011101110000101111000010101100000100101100001001011

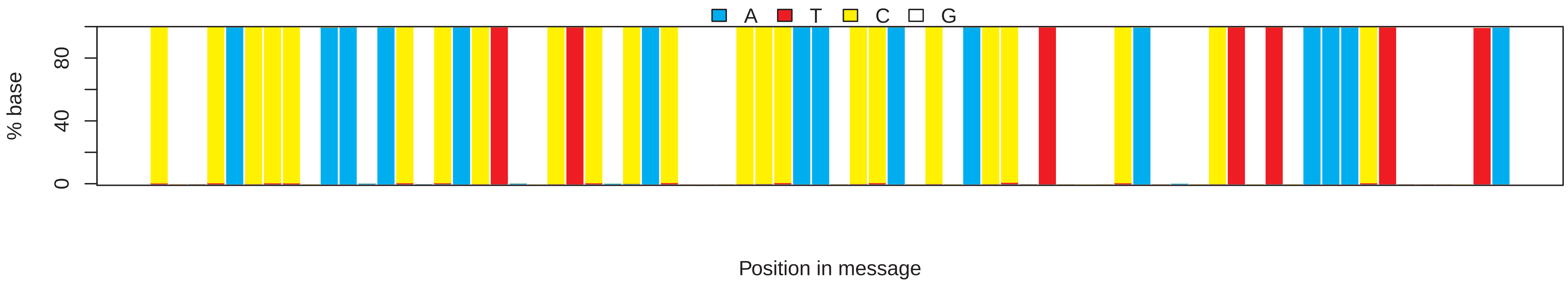

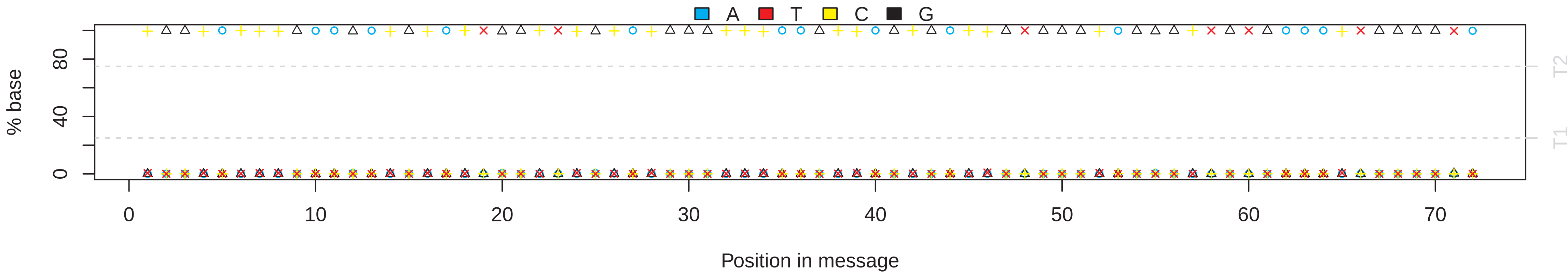

0110000010010010001110101000111000001000101000111110011101111100001111110

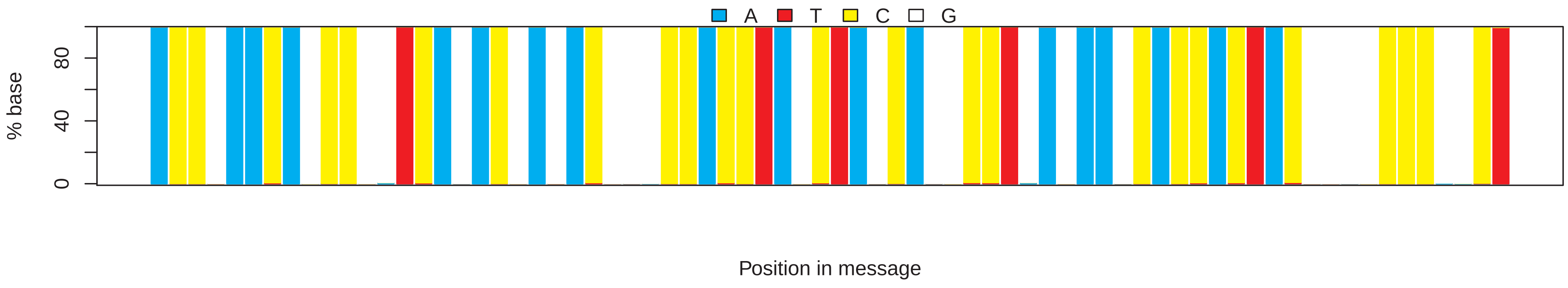

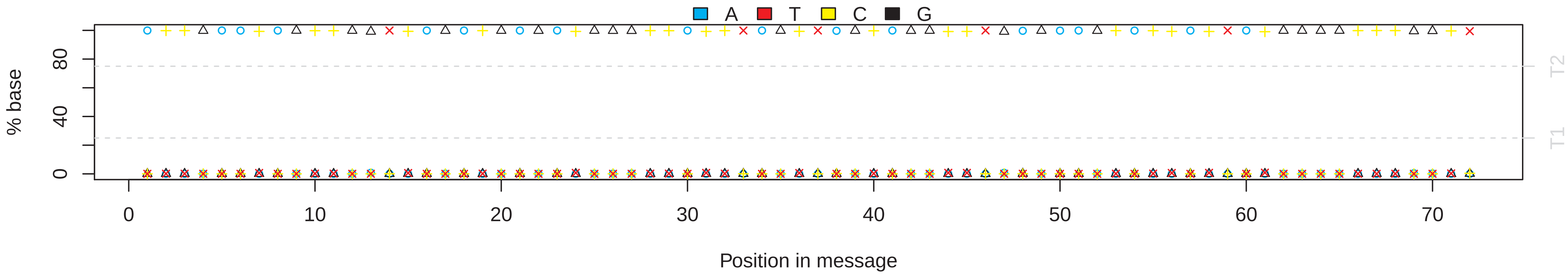

000100001001110010010100111000001010101001100110100100000010011110001101

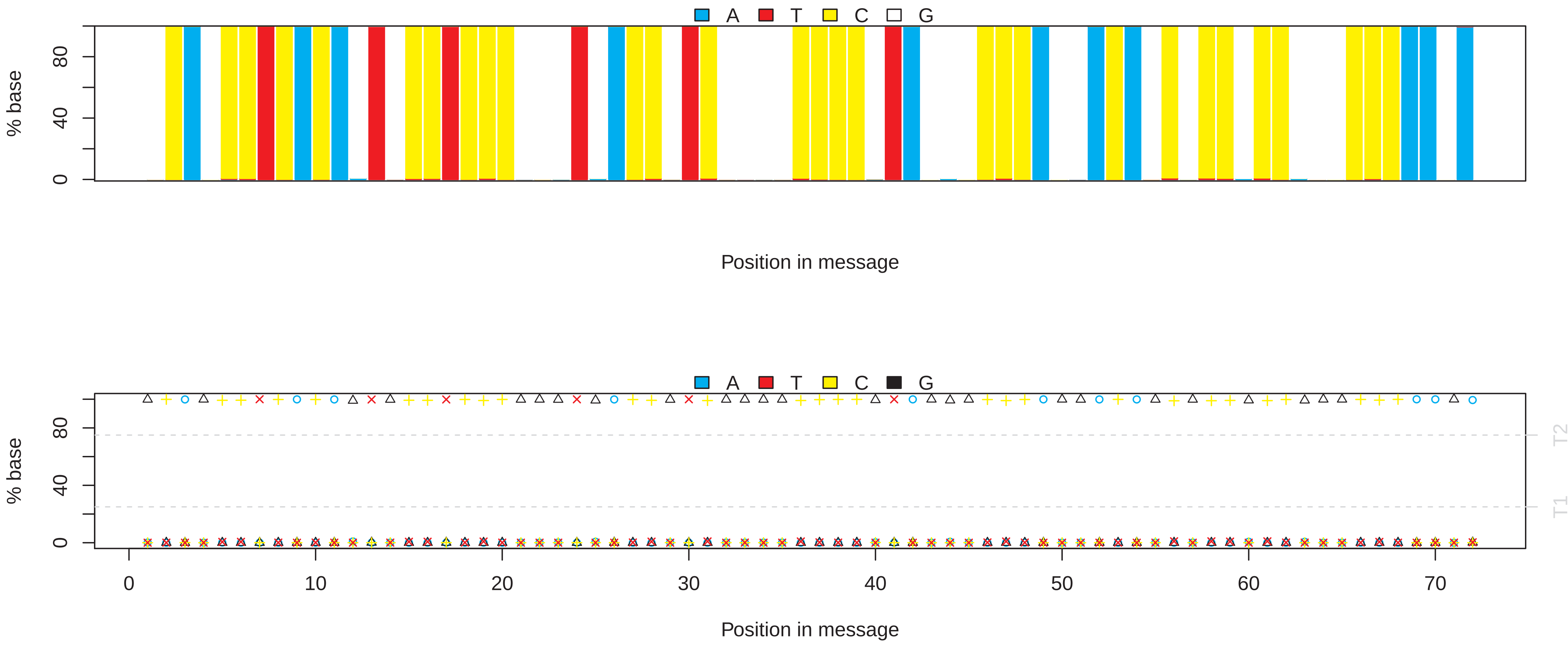

100100100001110010001111100011011110000110111000011000101001001110000010

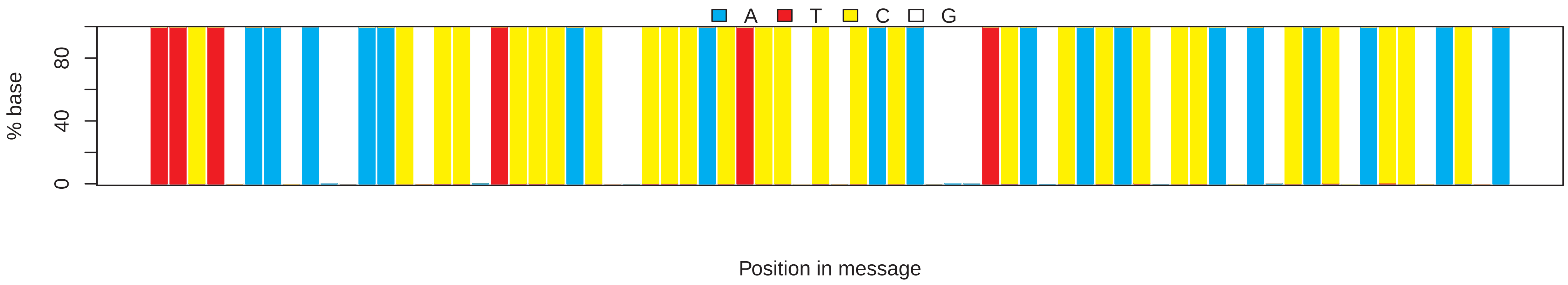

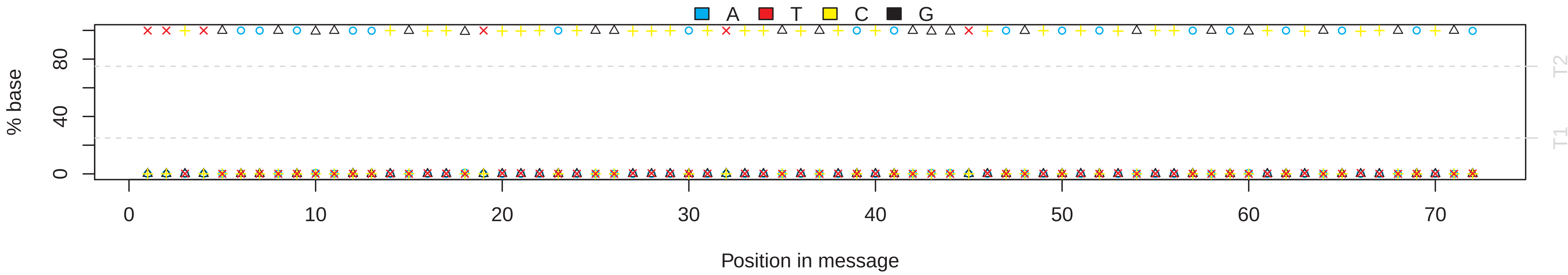

110110010110001001100000110000010010100001111001000001000101000100010010

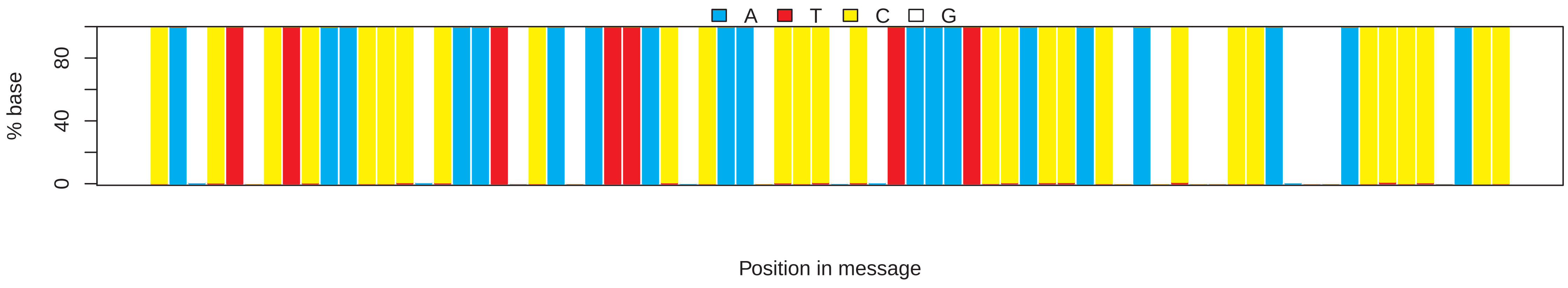

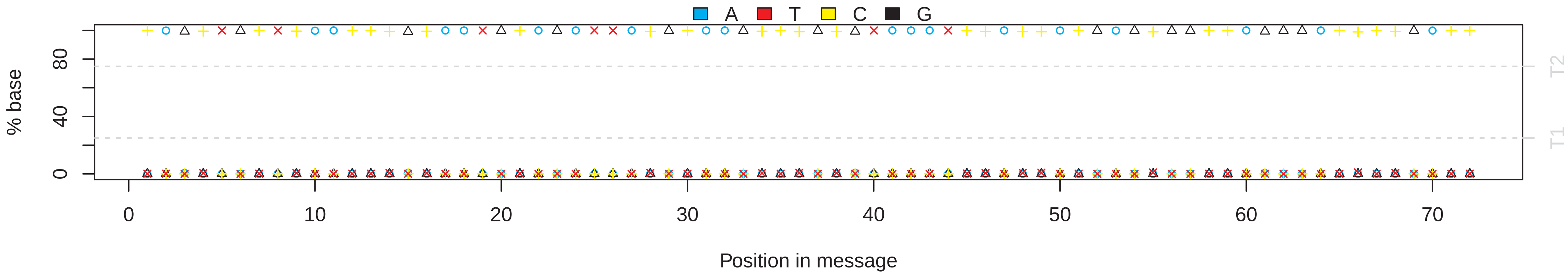

001011010000001000110010110010001000101100010000000101011000111000001000

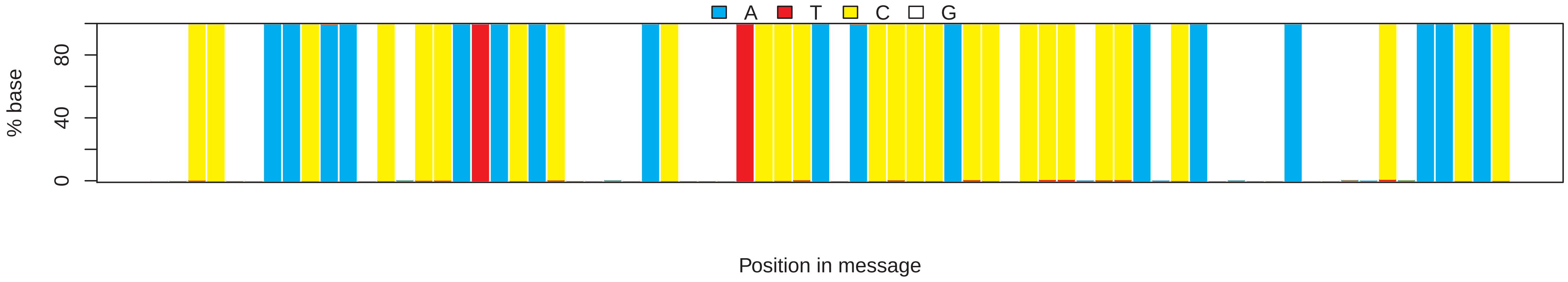

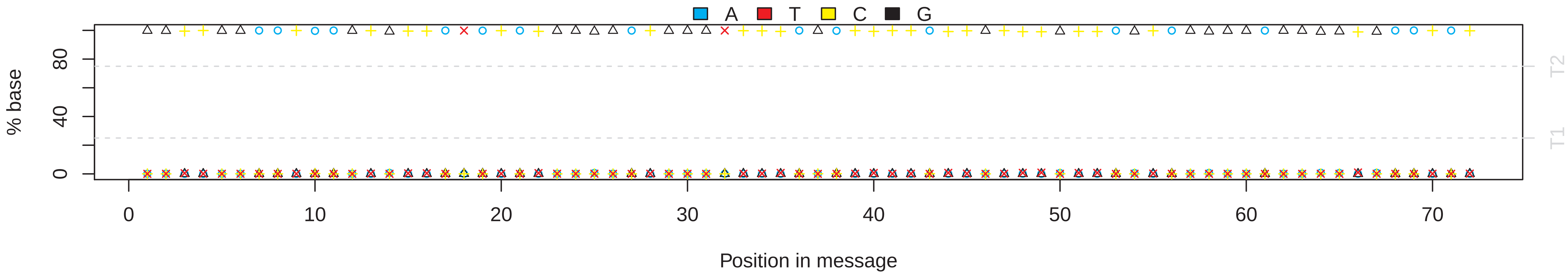

110011000001010001000011110011110000100000000100010001001111011110100000

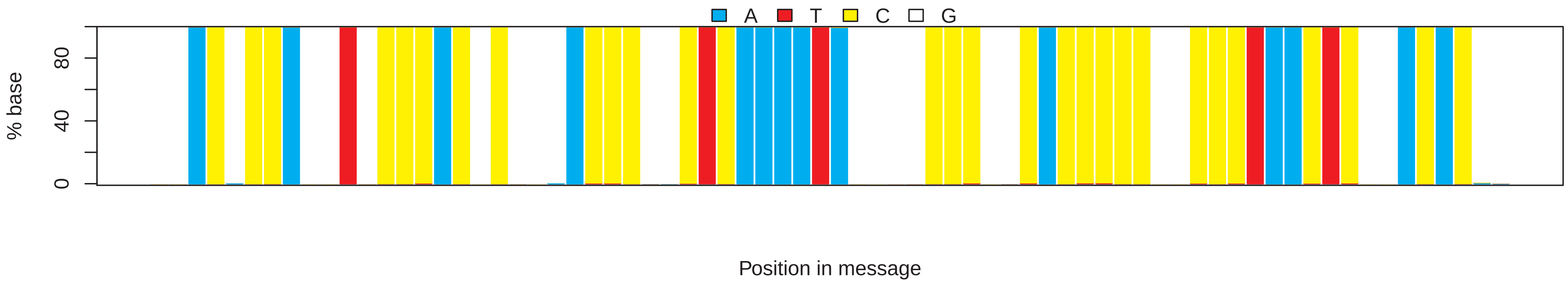

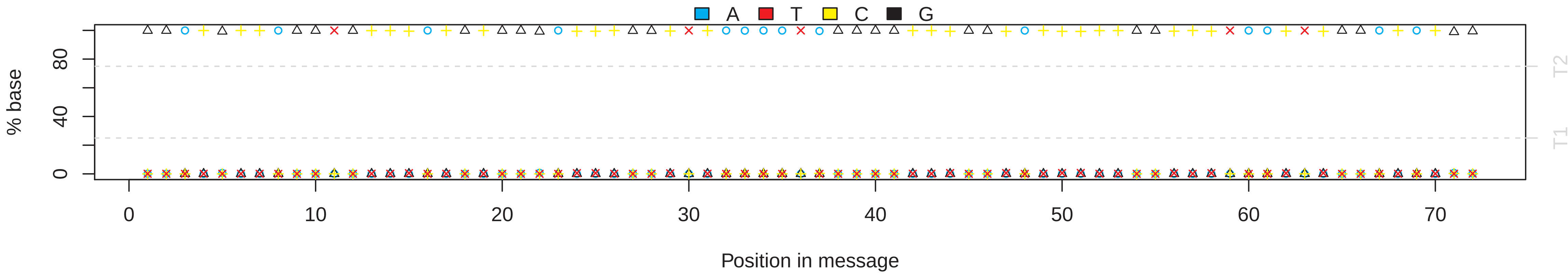

110010001111000001011100001101000001011110001100000001100010001011000011

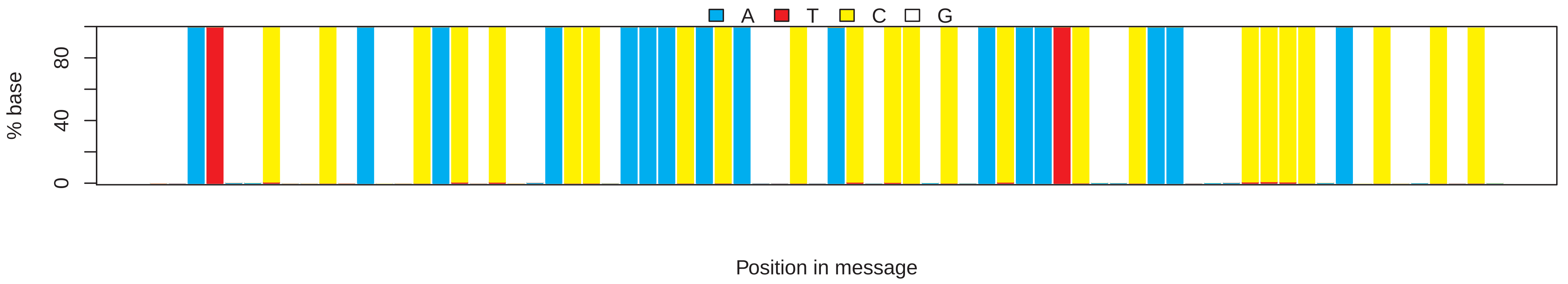

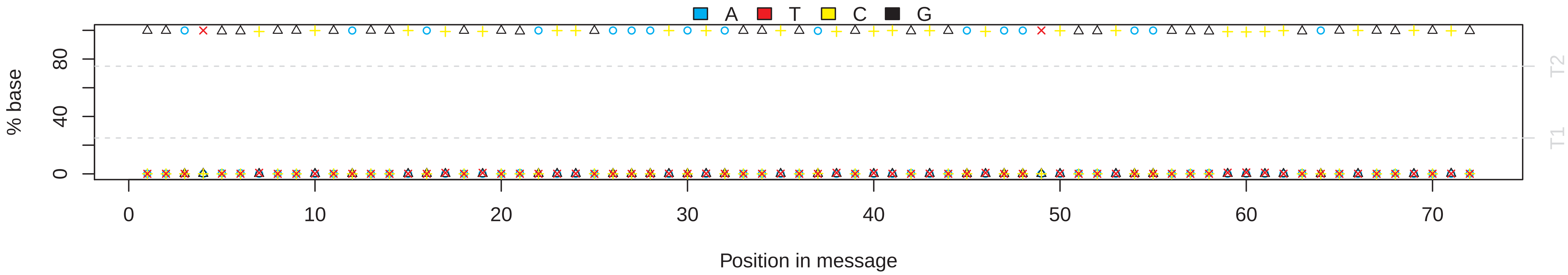

110111011010110001011000100000001101001001010000101100011100001010110101

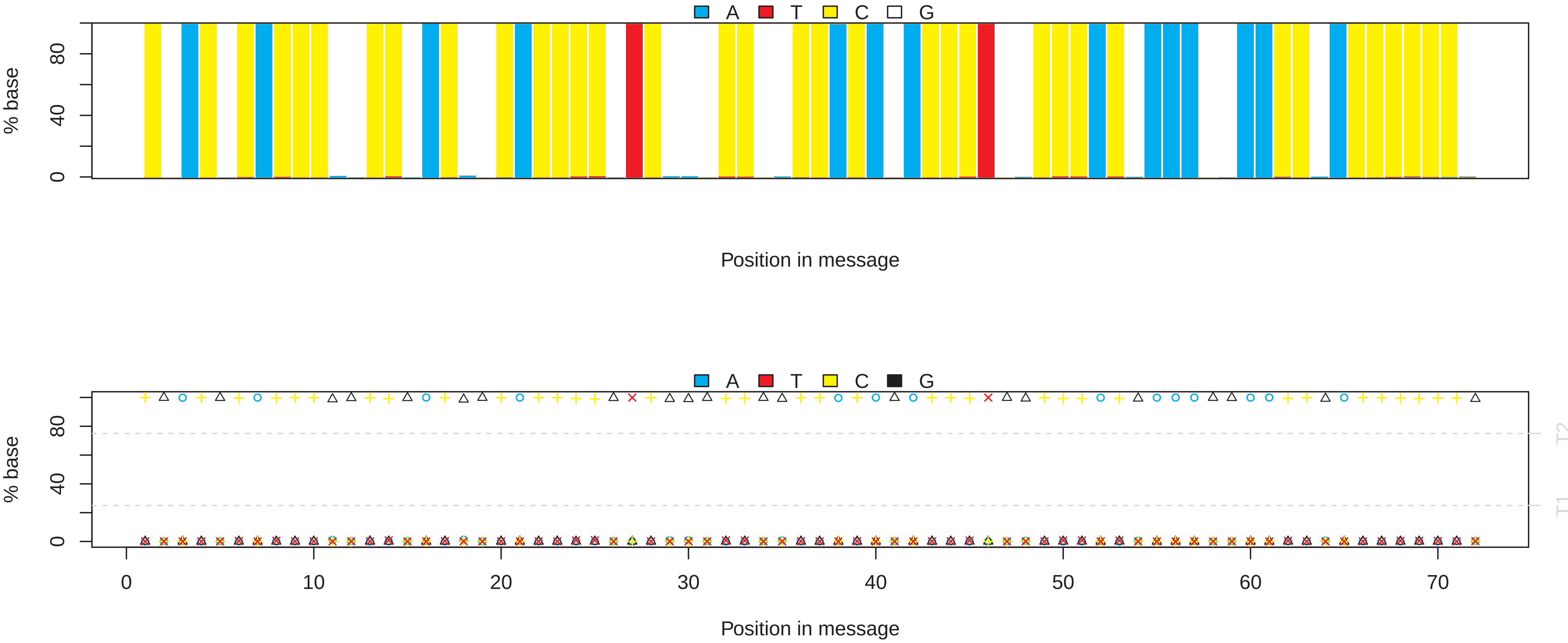

01001000001100100110000001101110011000001000011100000100011000010000000 1

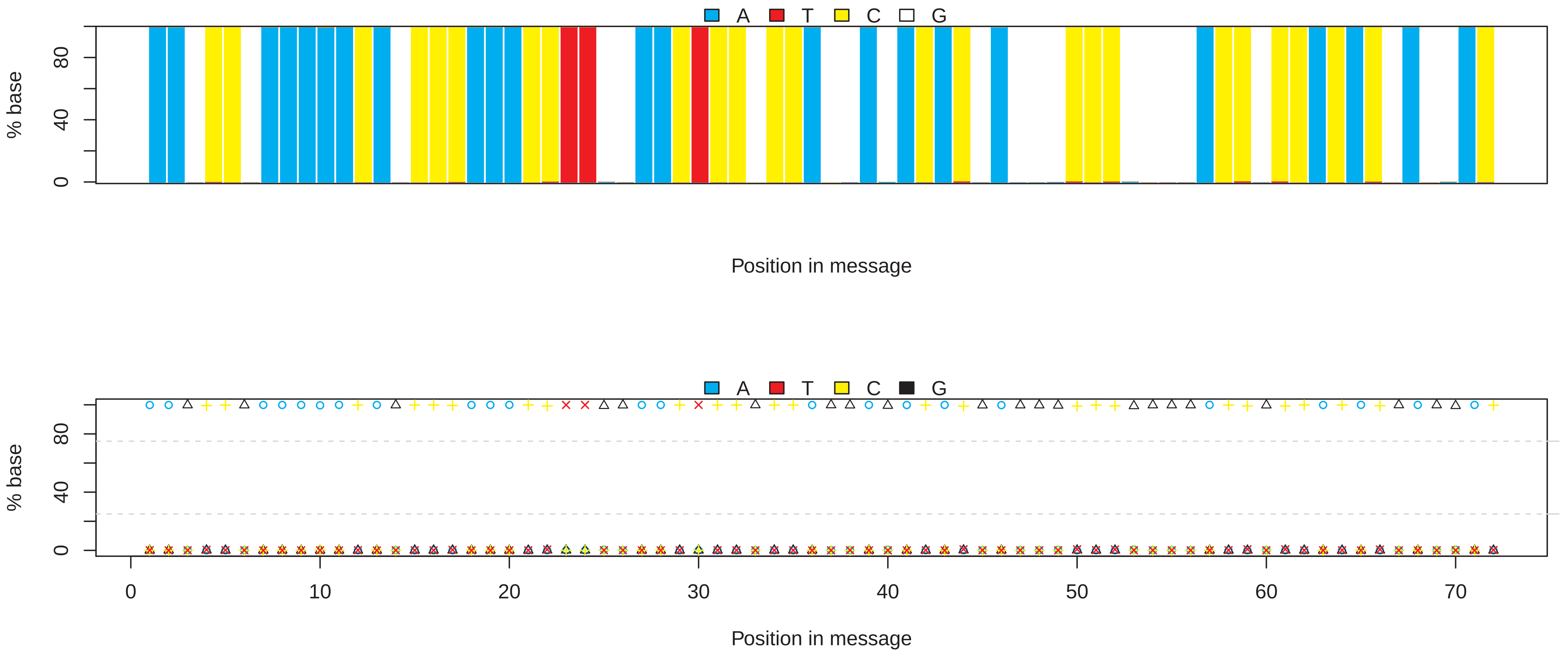

0010010000000100000000111100010010001101000010111000111100010000000101100

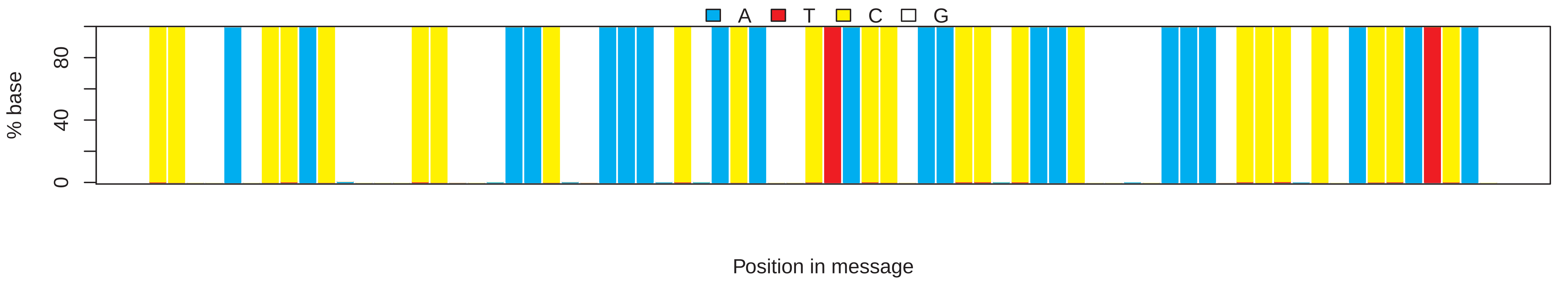

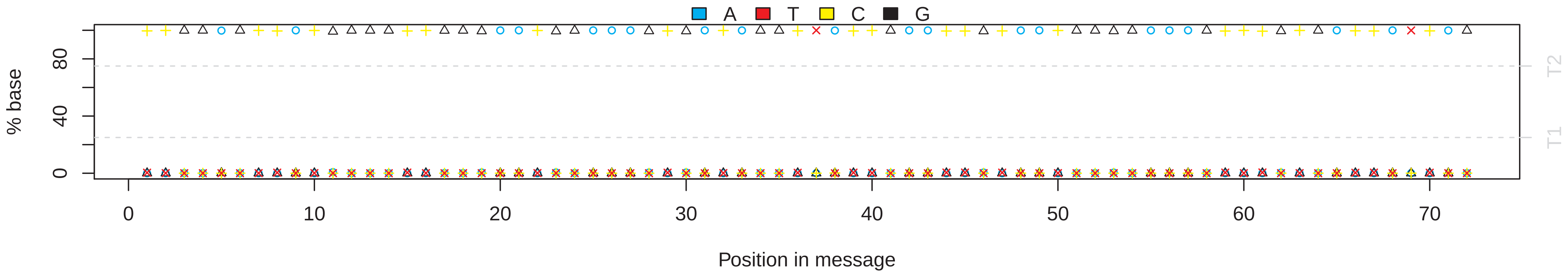

0011010000111100111000110001010001101000100001000011110001000101000001001

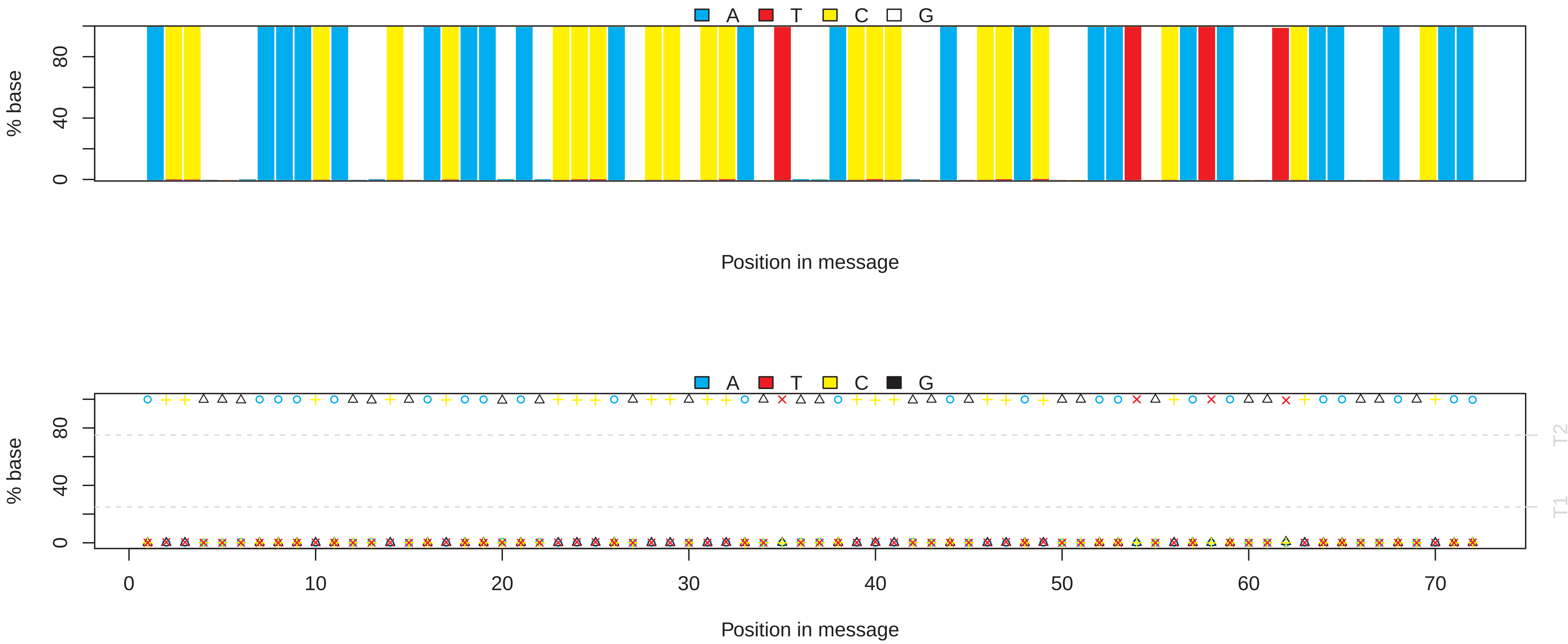

000111000001101000010100001001000111100001101000011001100101110001101000

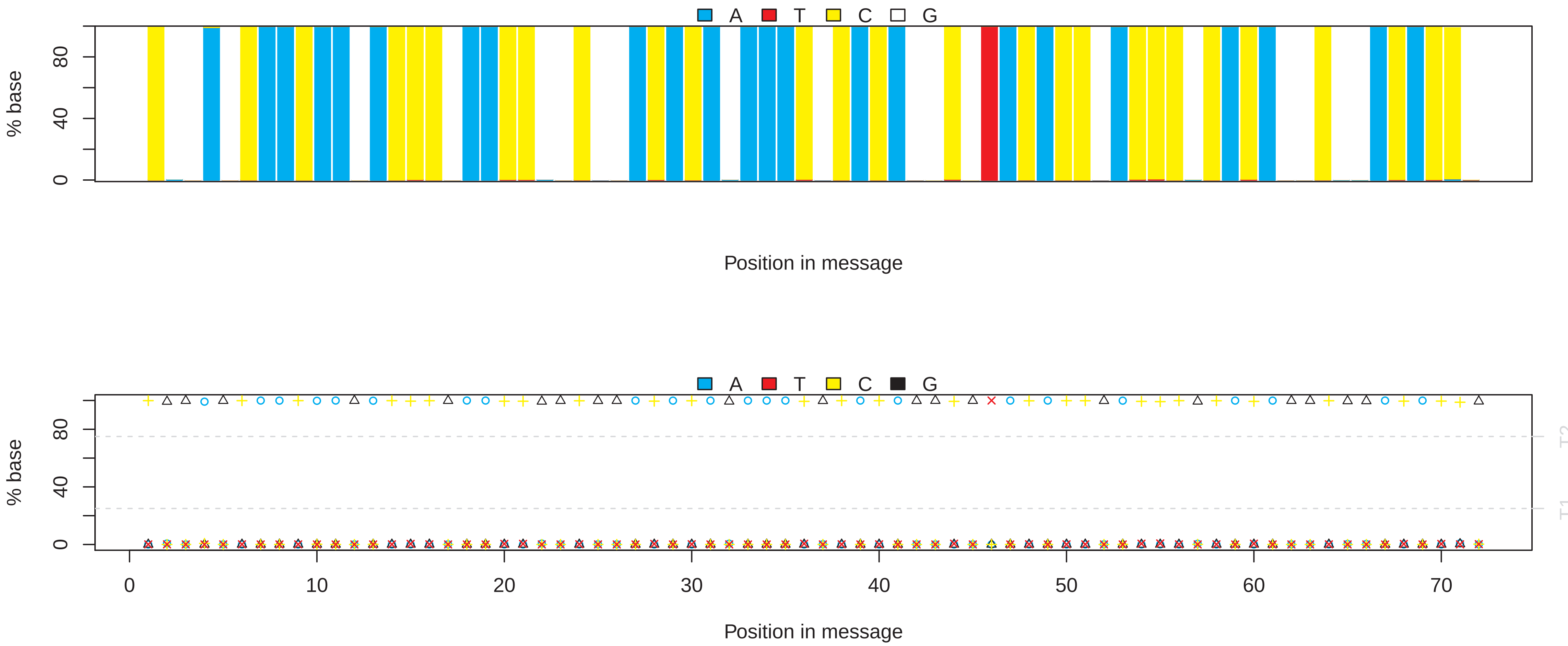

011010000001000010000110110000010000100001101100000100001000011011000001

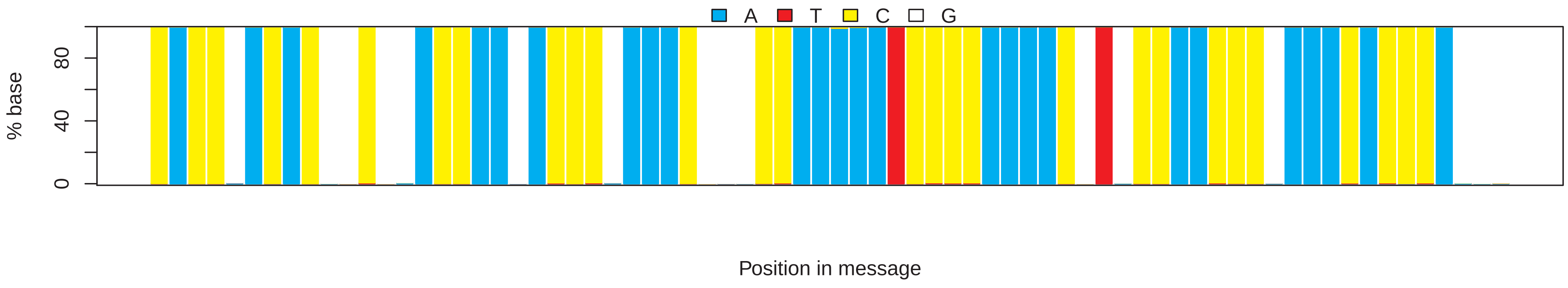

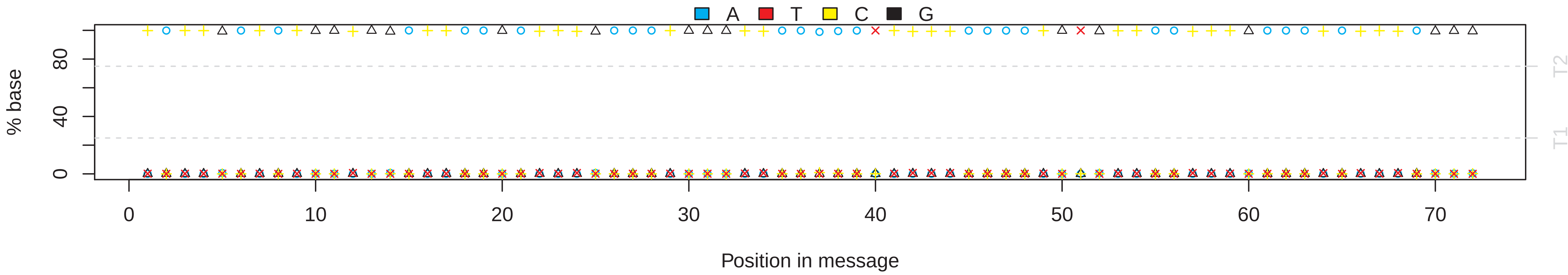

000010000110110000010000100001110000000100000000011100000001000000000111

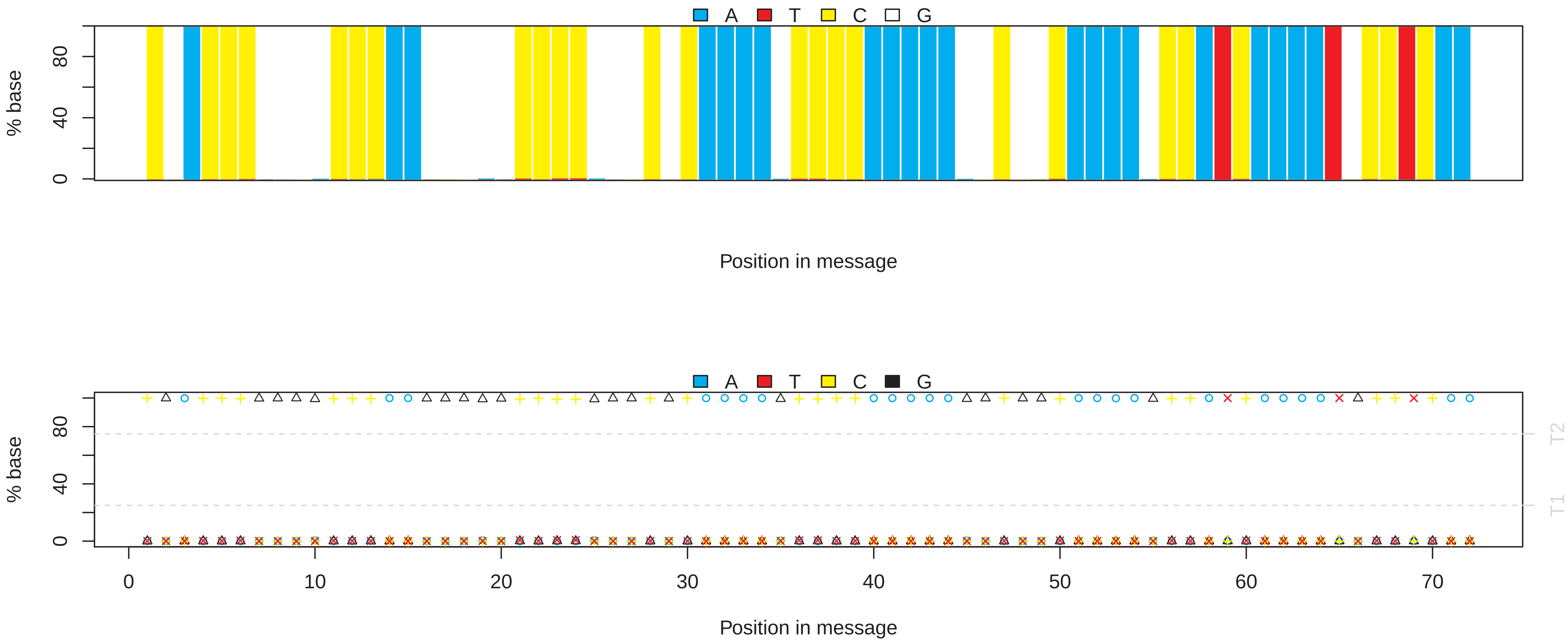

010000111100000111110000111010000010000000001101100000100010000011001000

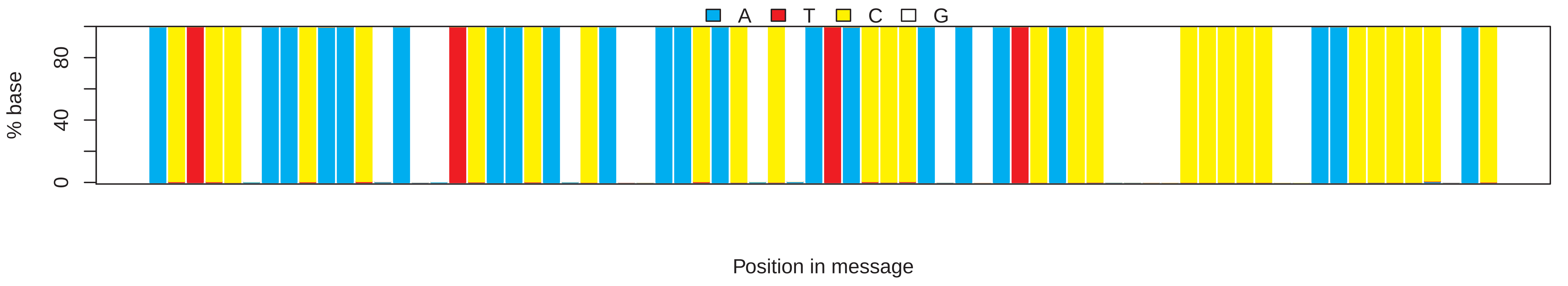

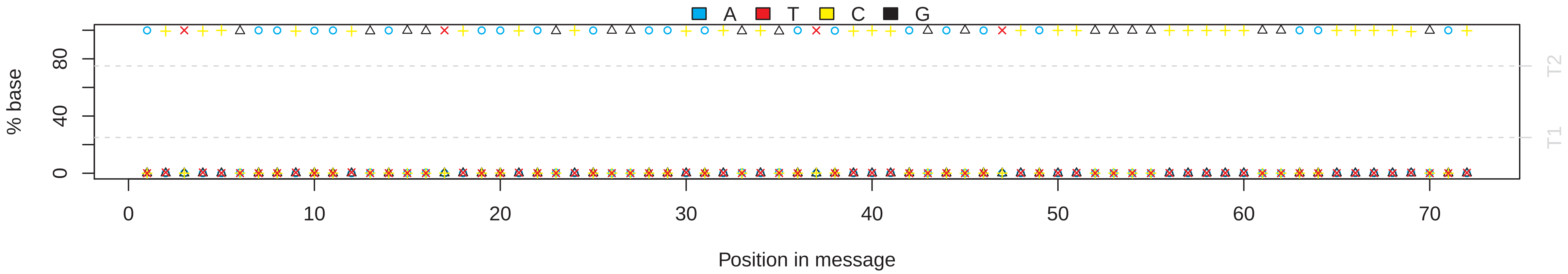

001001000000101110000010011000001010100000101010000111100000110000000100

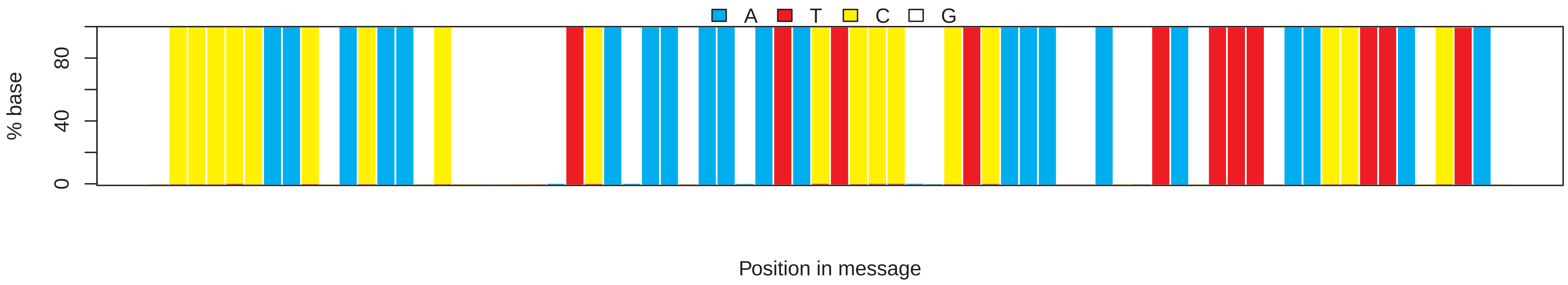

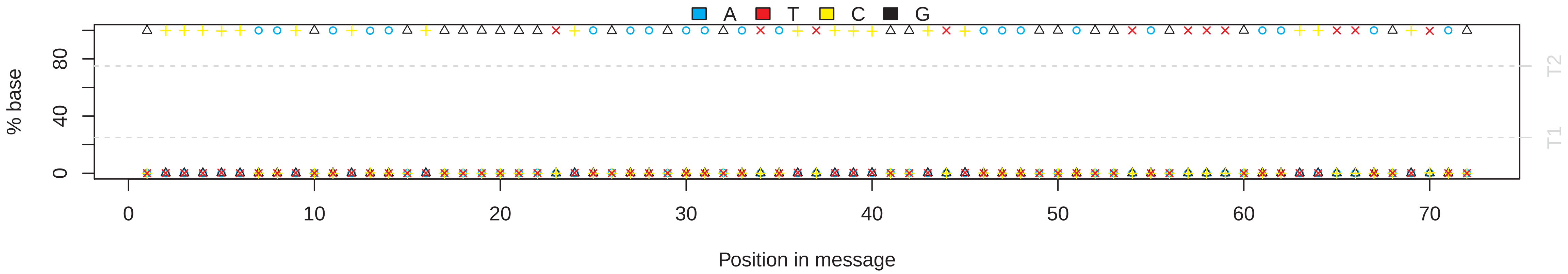

100000000100001011111110010010010100100011010000110111011111000011010101

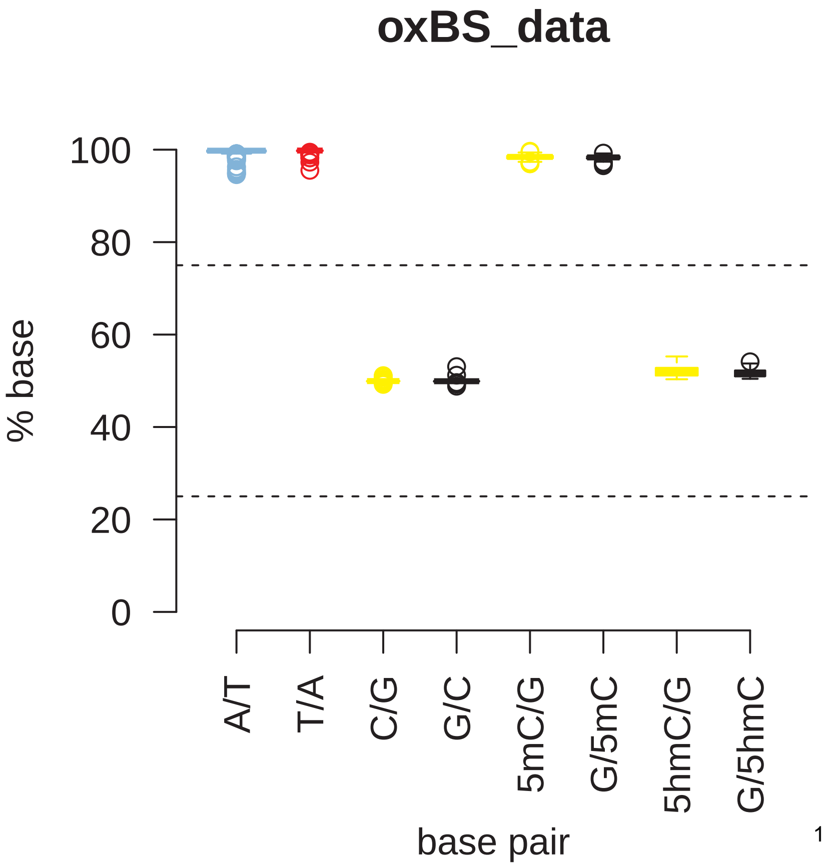
oxBS_data
% base
100
80
60
40
20
0
A/T
T/A
C/G
G/C
5mC/G
G/5mC
5hmC/G
G/5hmC
base pair

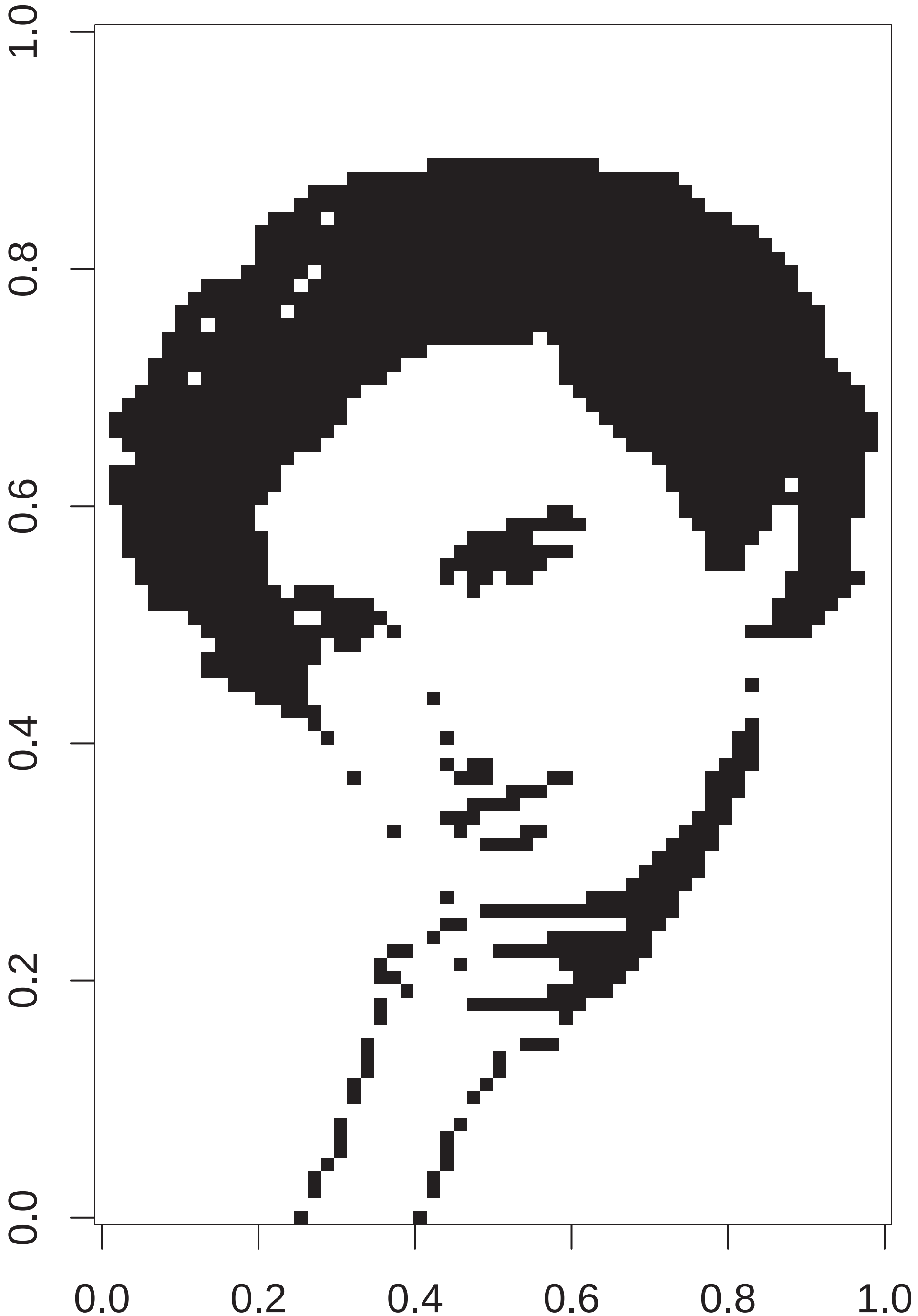
0.0
0.2
0.4
0.6
0.8
1.0
0.0
0.2
0.4
0.6
0.8
1.0

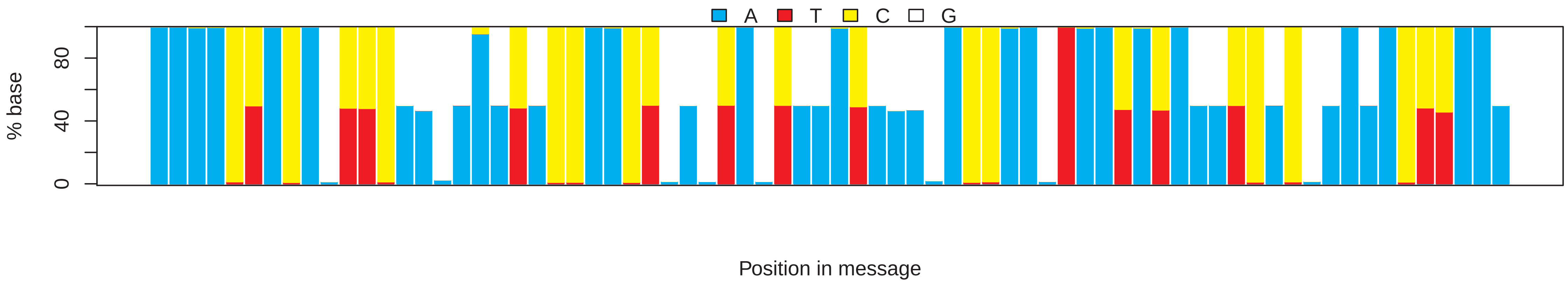

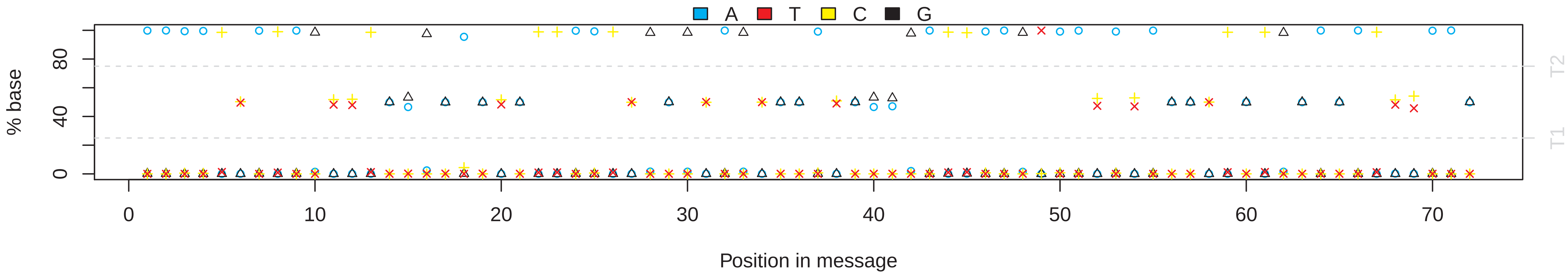

000001000111000100010000001101101100010001000001100101000100010000011000

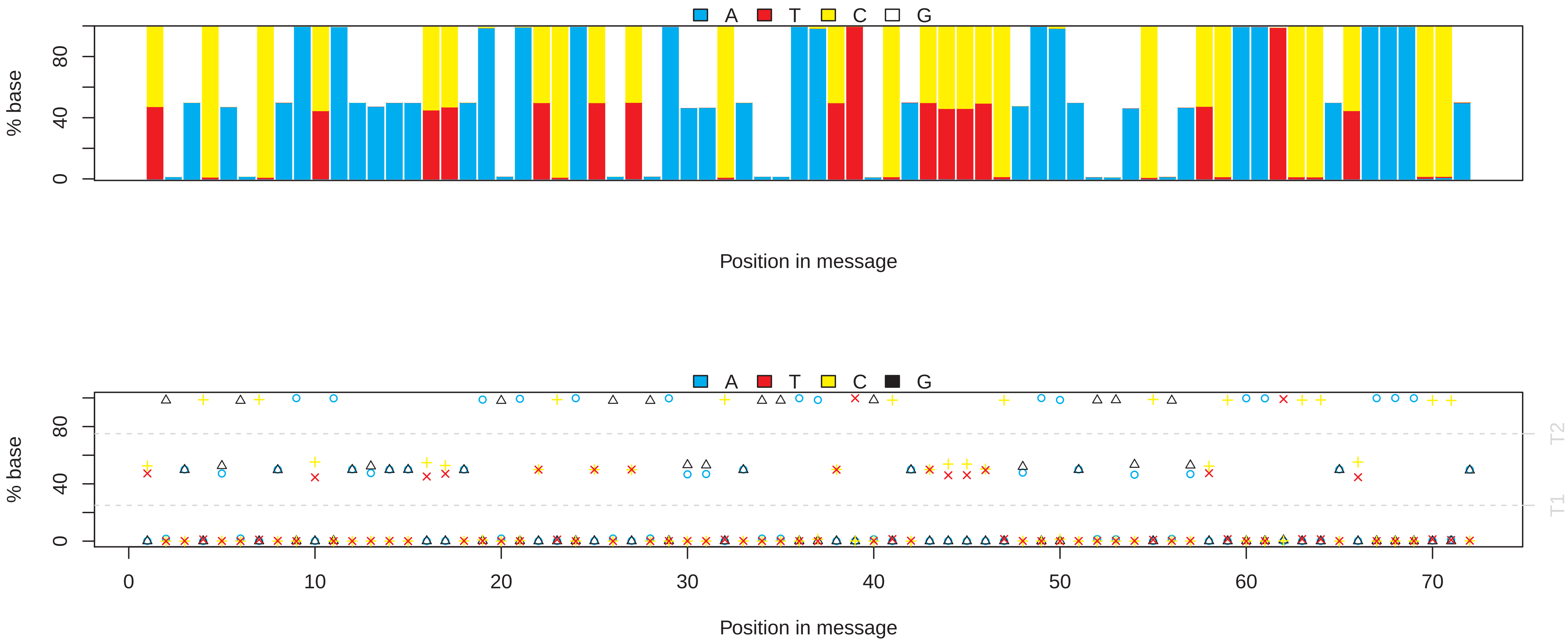

110001000100000110010100111100000110011100111100000110010100010001000000

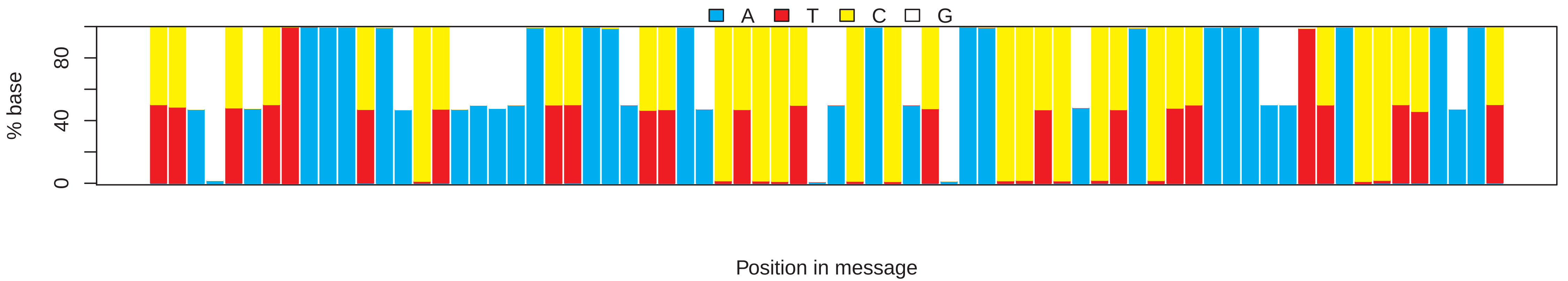

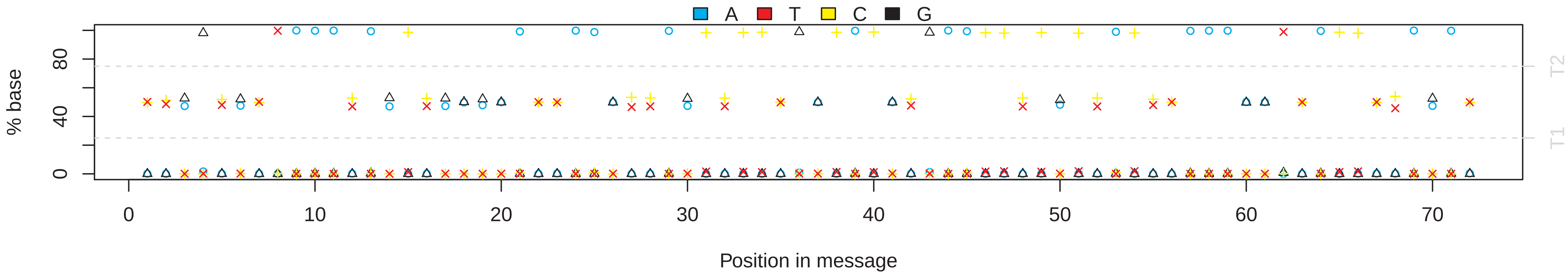

1101101100010001000001100011000100110000011000010001001100000110001100 01

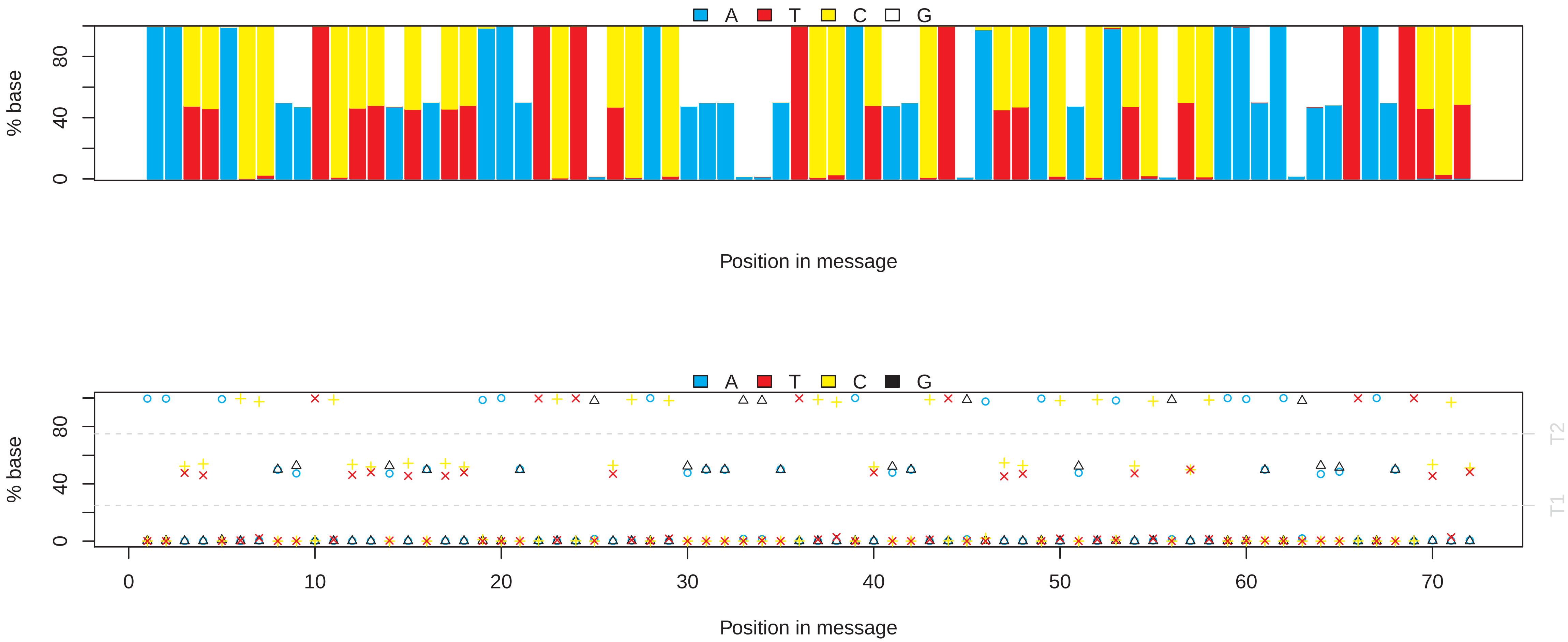

001100000101101011000101110000001101000100011011000001011000001001001101

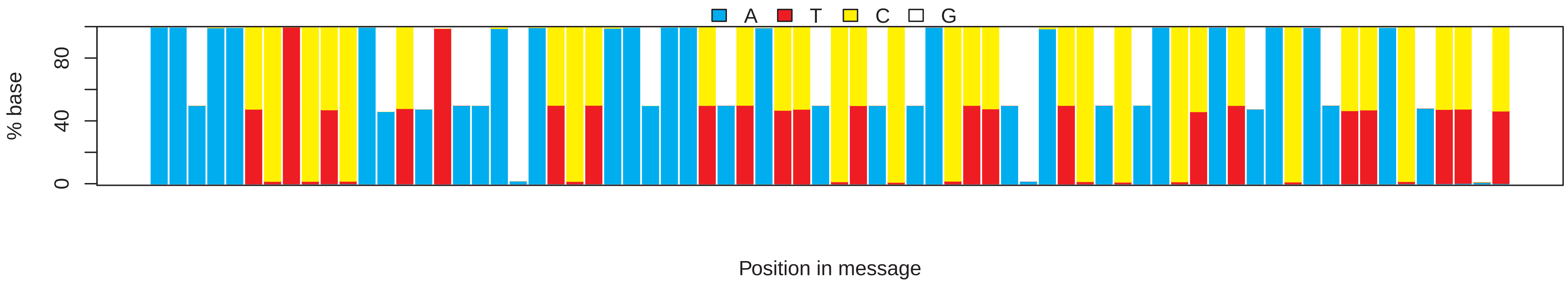

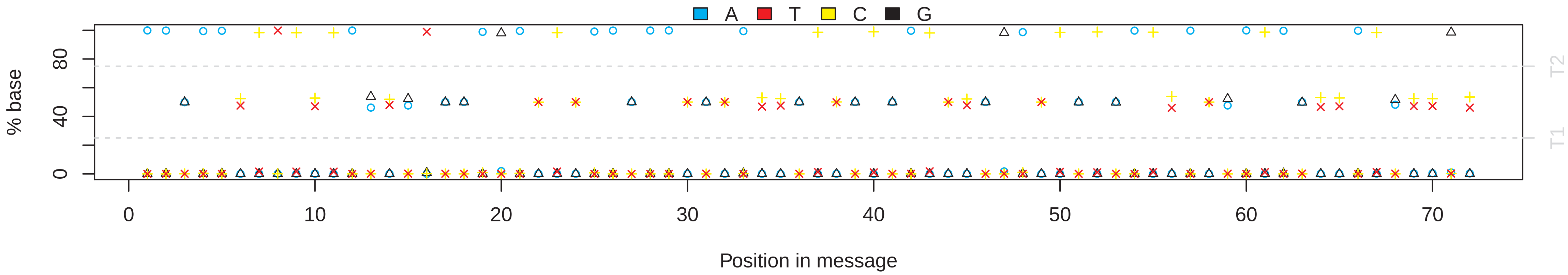

000001010100010100010101000001010110010000011010100000010100000110001111

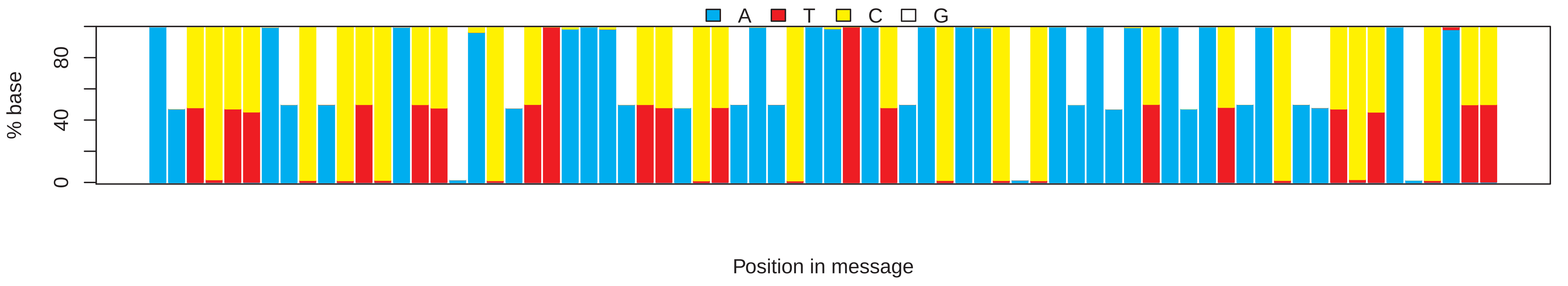

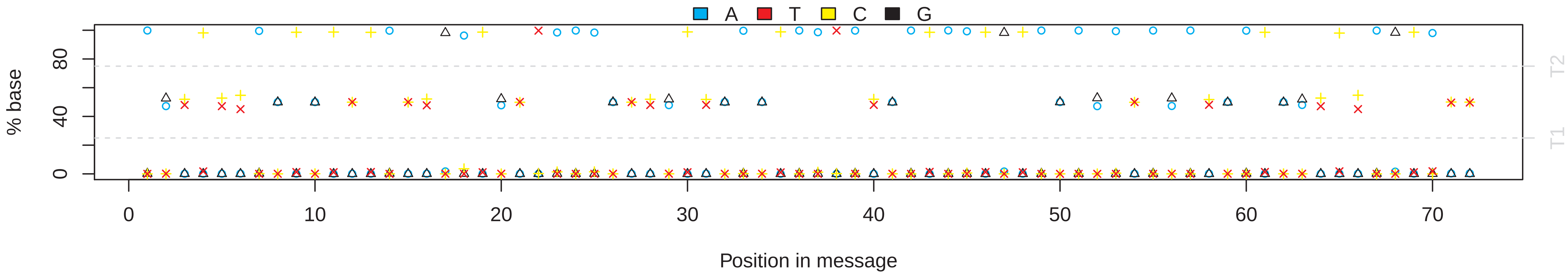

0010110000010011100011000011001000000101000000100000010001000001010100 11

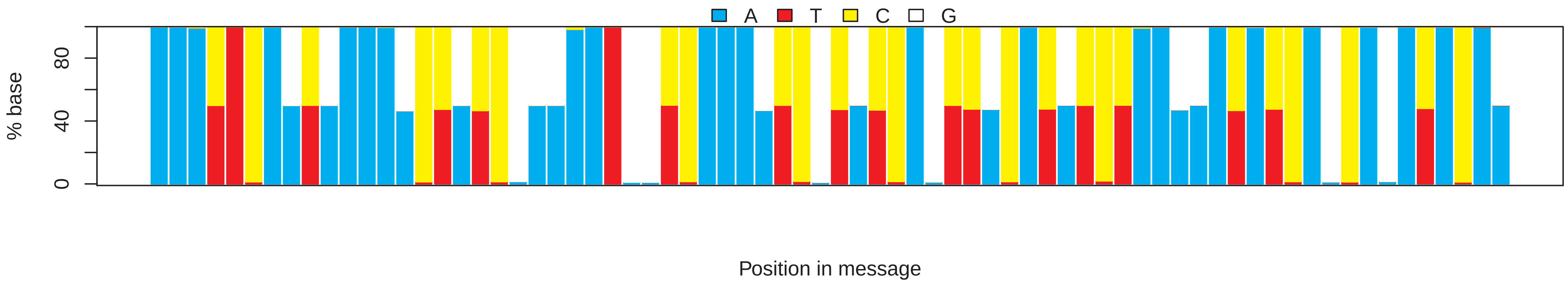

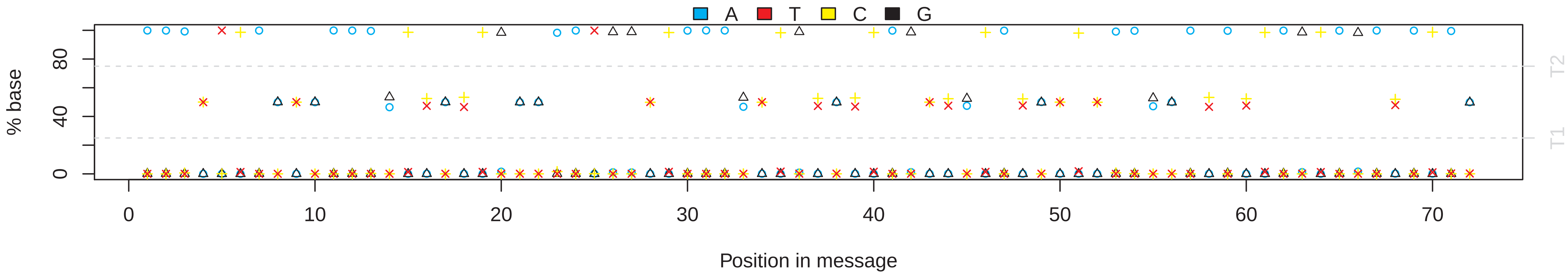

000110001000000101010000111100000101101001110001010100000101001001010000

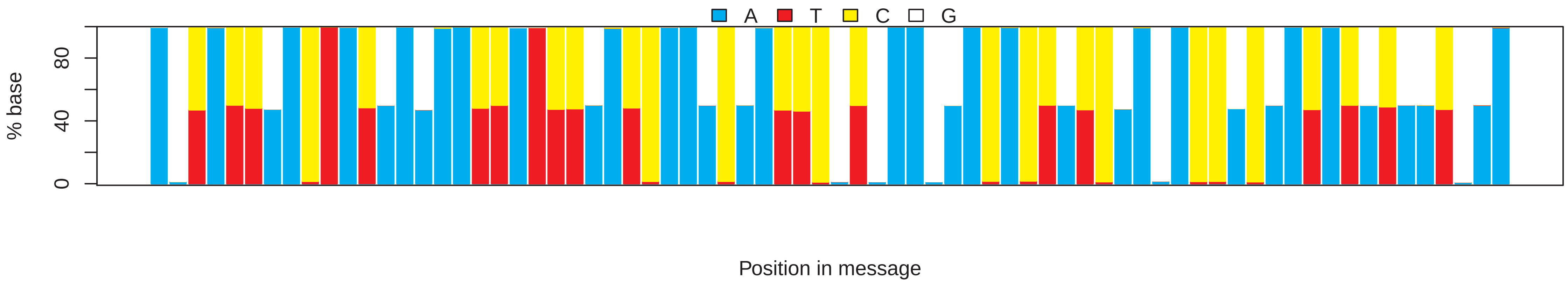

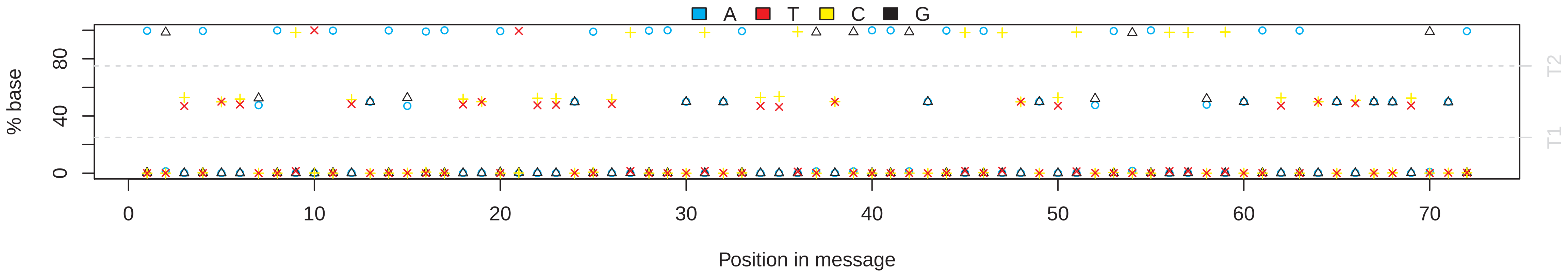

011011000101000001101110010000000110111001000001010001000000010101001100

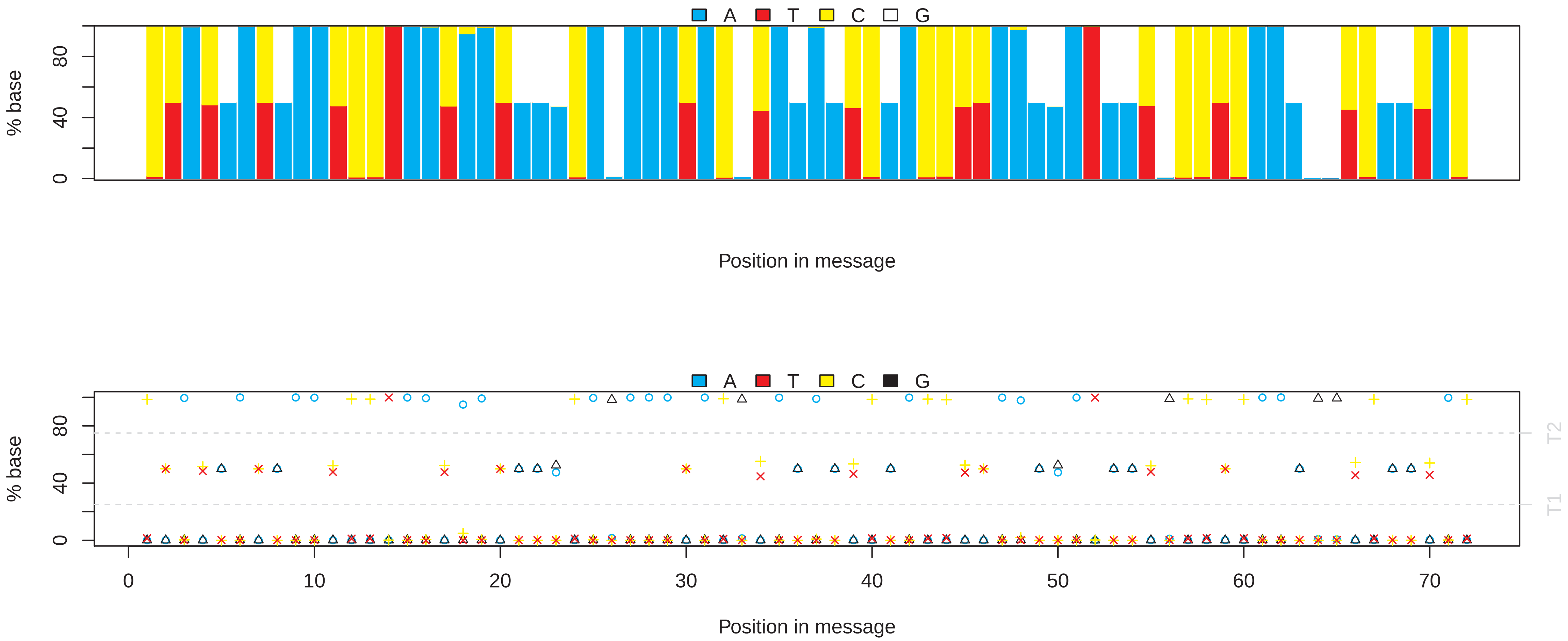

010100100010010010010000010001001100001000001100000100110010000111000100

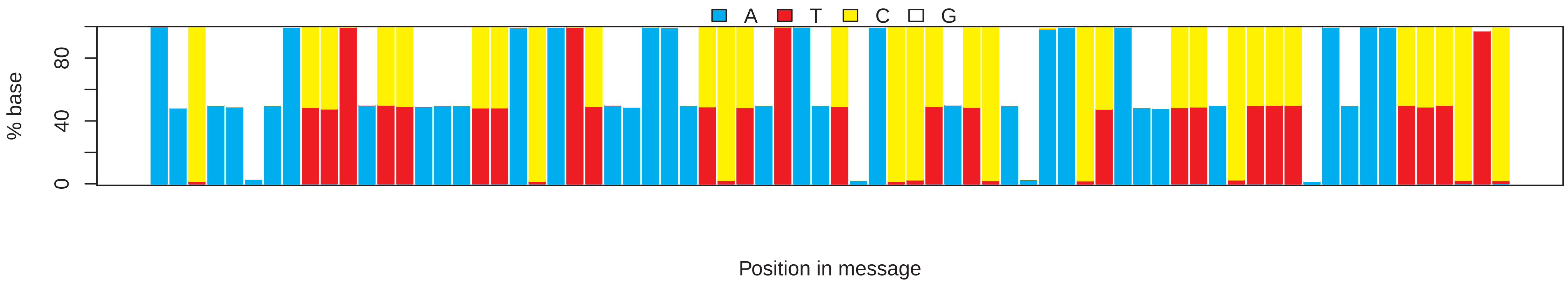

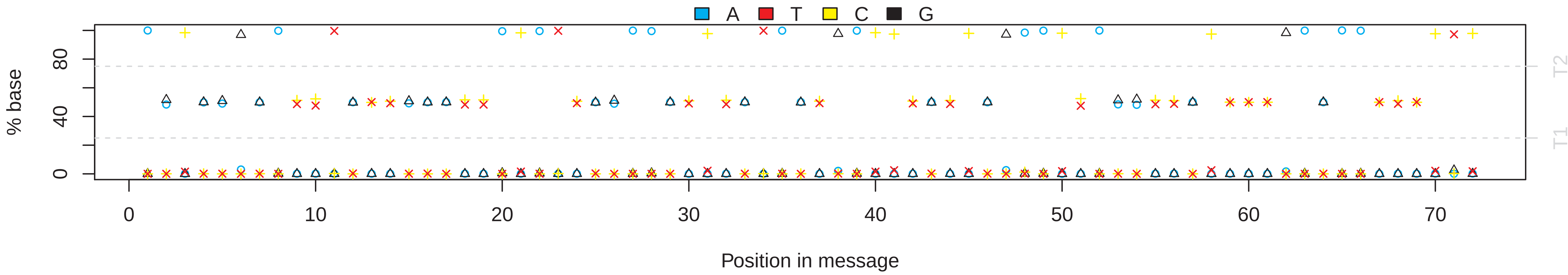

000001001110110001100011000001010100110001010010001000110011110000111010

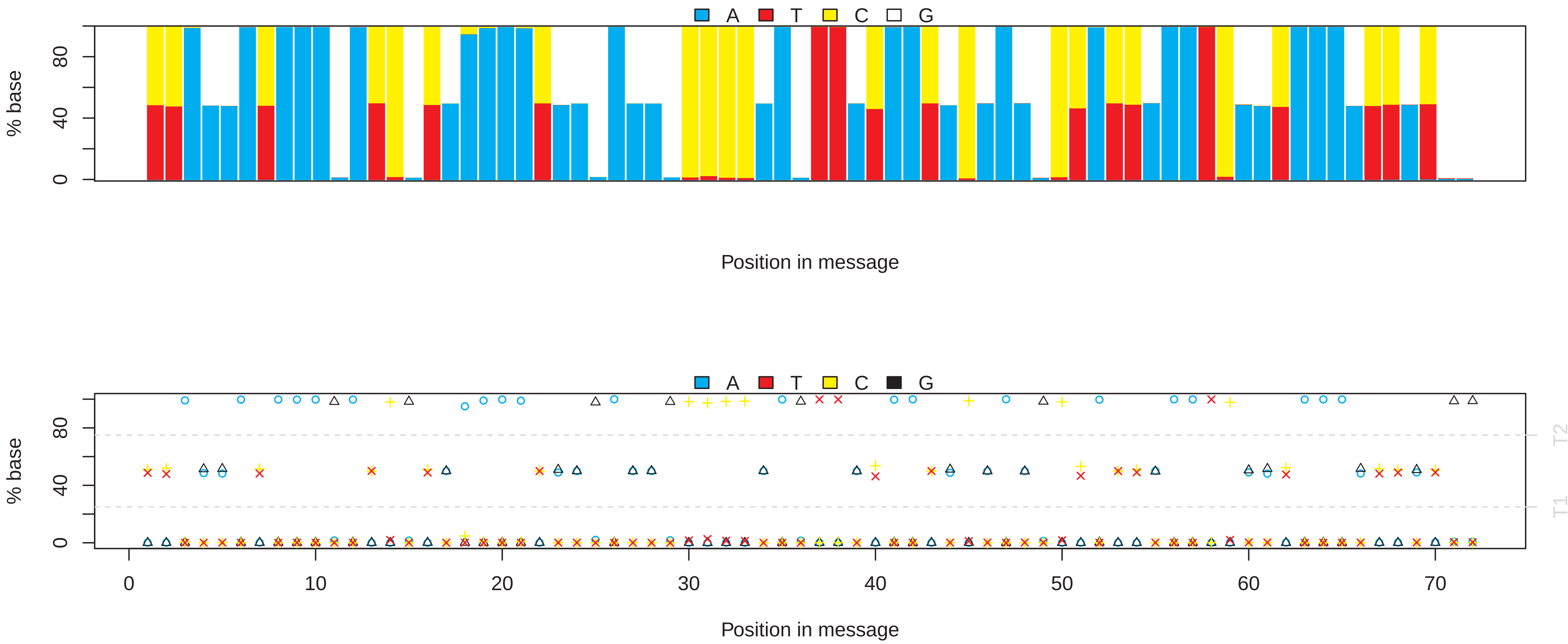

110000100010101100000100100010000001110100100000101011000100010000110111

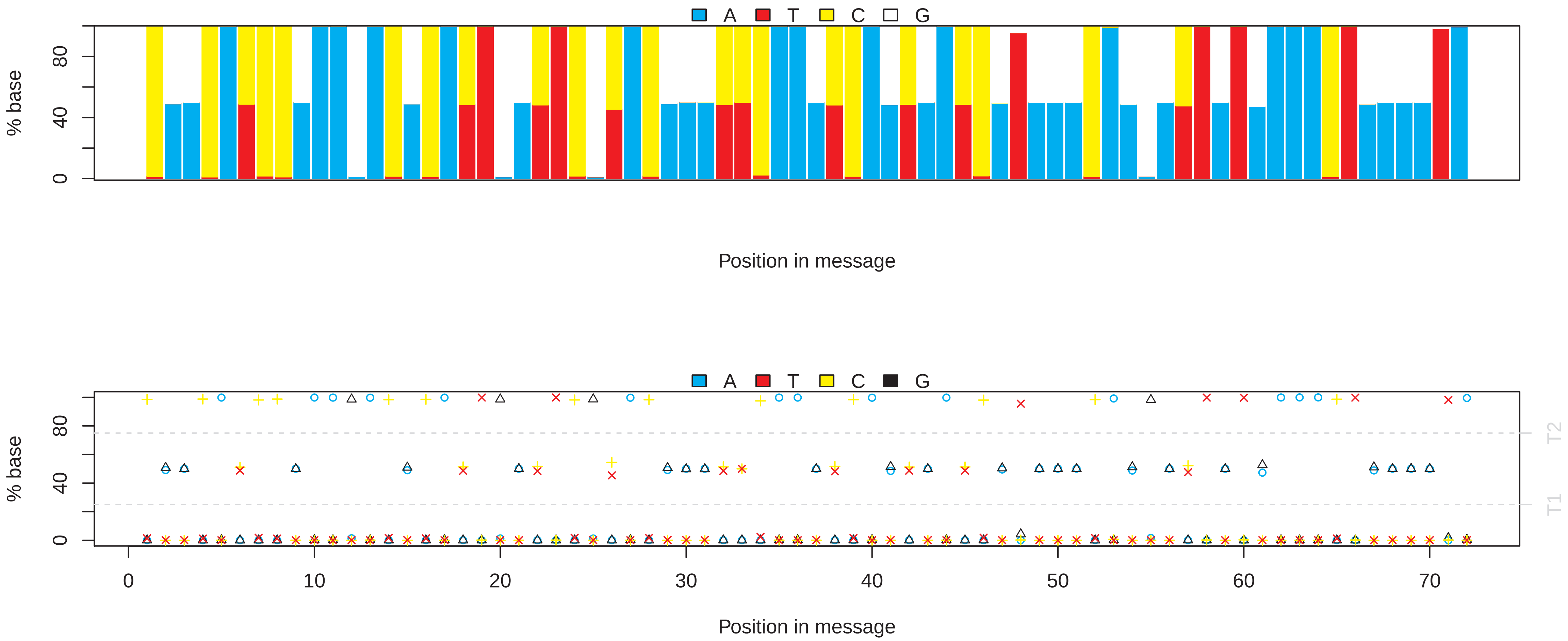

00000100000100000111011011000001100001000100100100000001011010000001000010

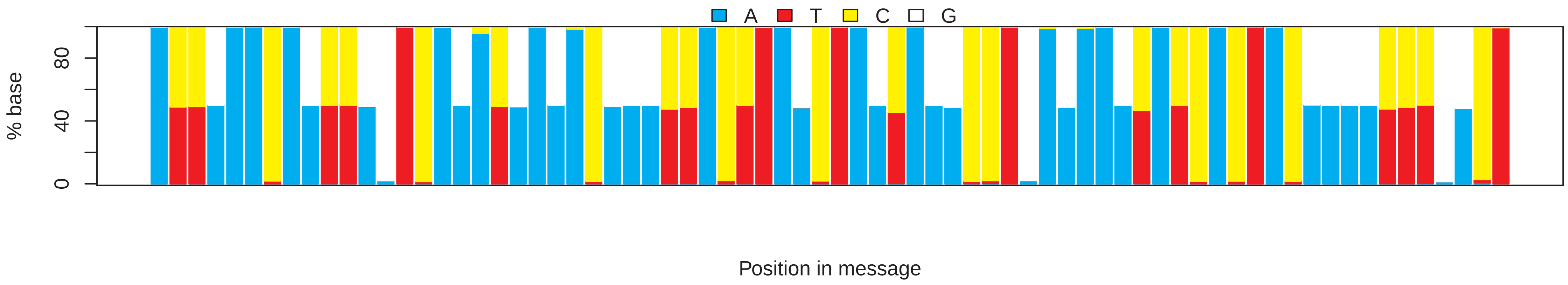

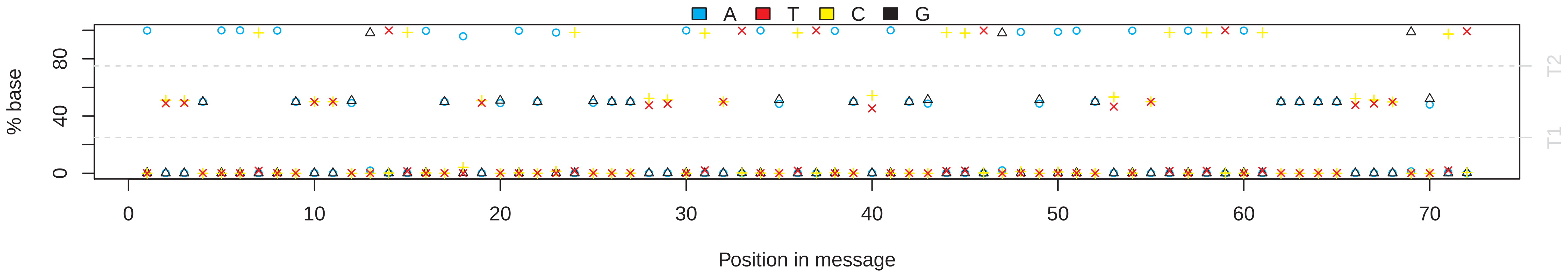

011000000110110000100000000110011000100100000110000010100010000001111001

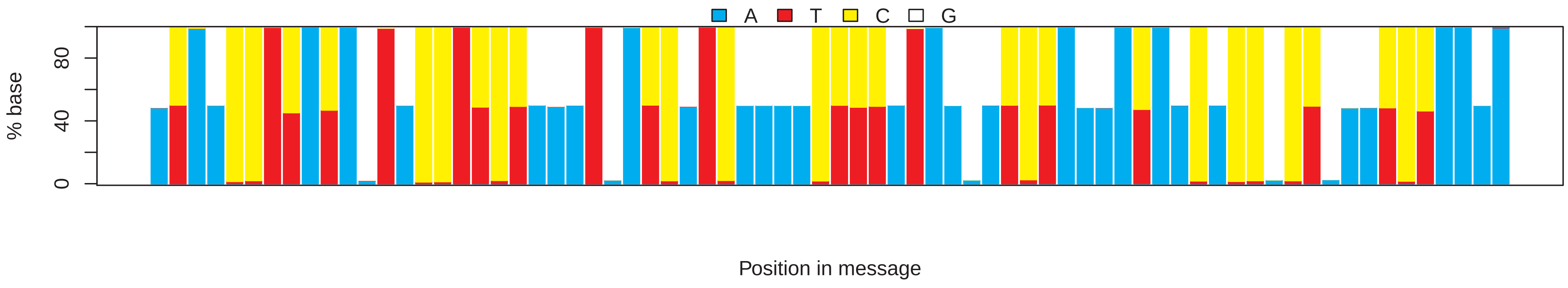

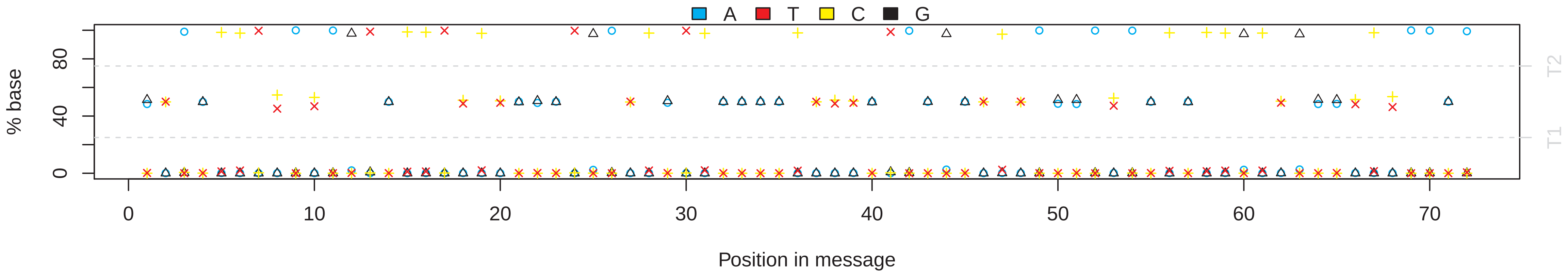

010000110101100011010001101001000000111010010101000010000001011001010000

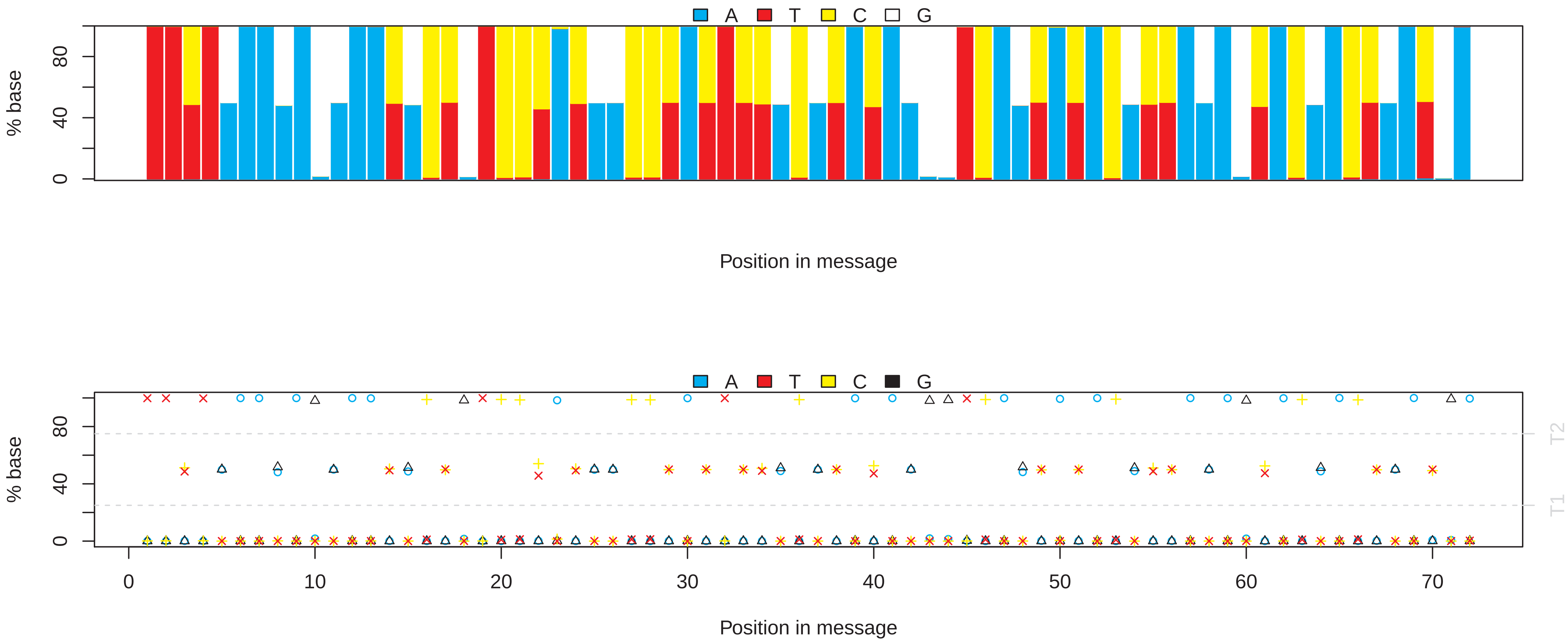

111100000100010011100101000010111100010100111000101000110001100000100110

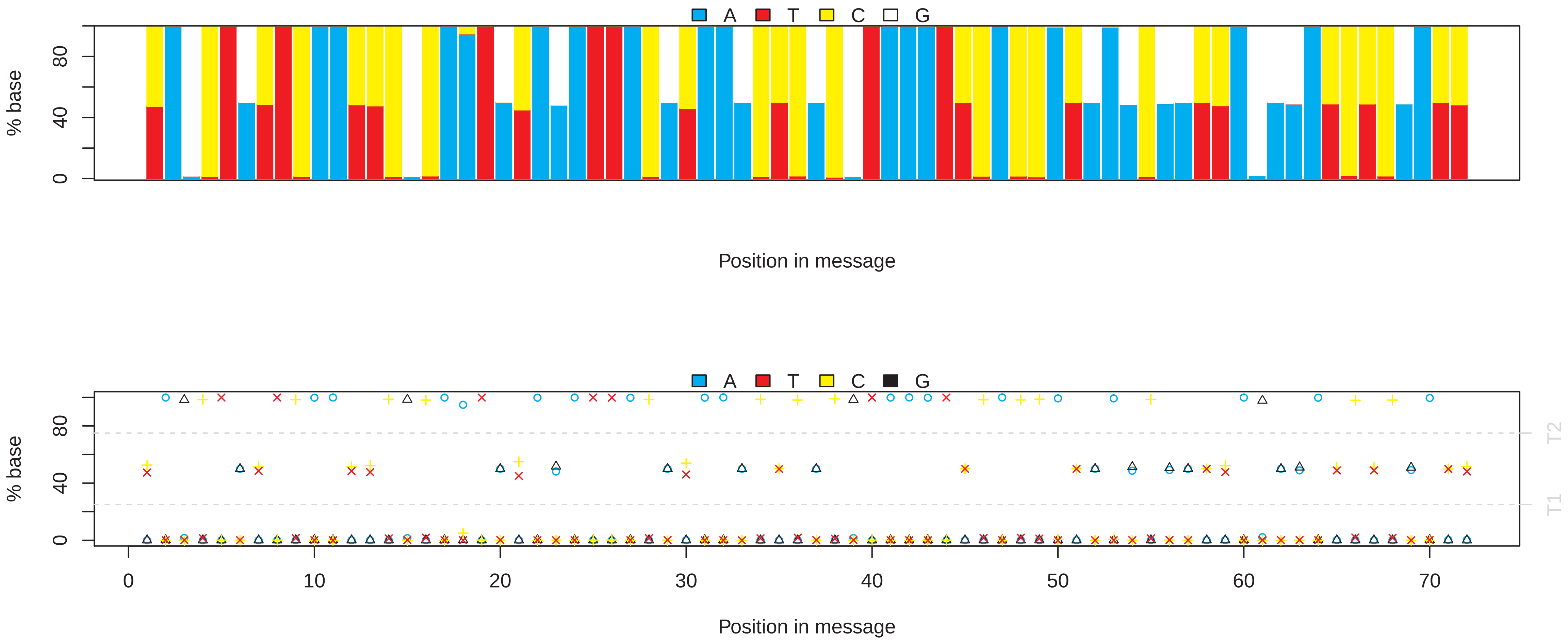

1010101100011010001010001100010000100011000110000010000001101000101000111

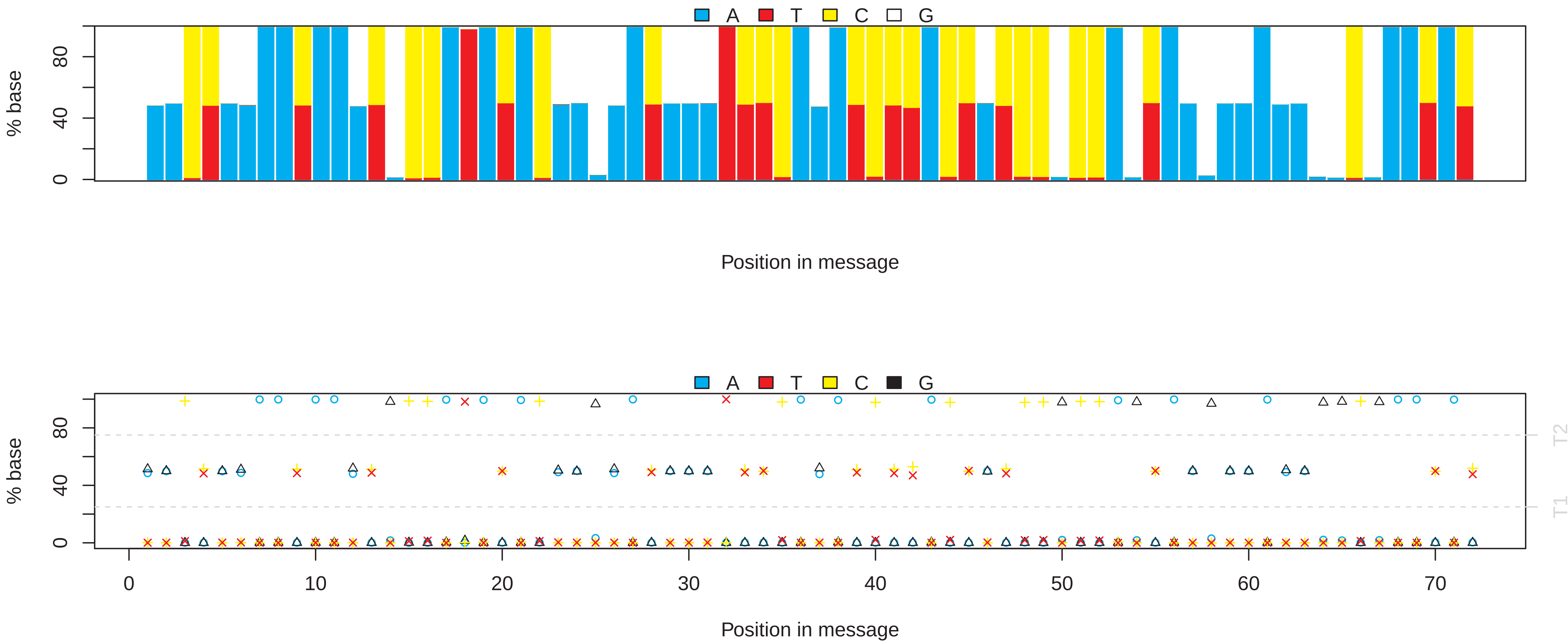

000100001000110001010000100100011100001011001010010001100100000110100101

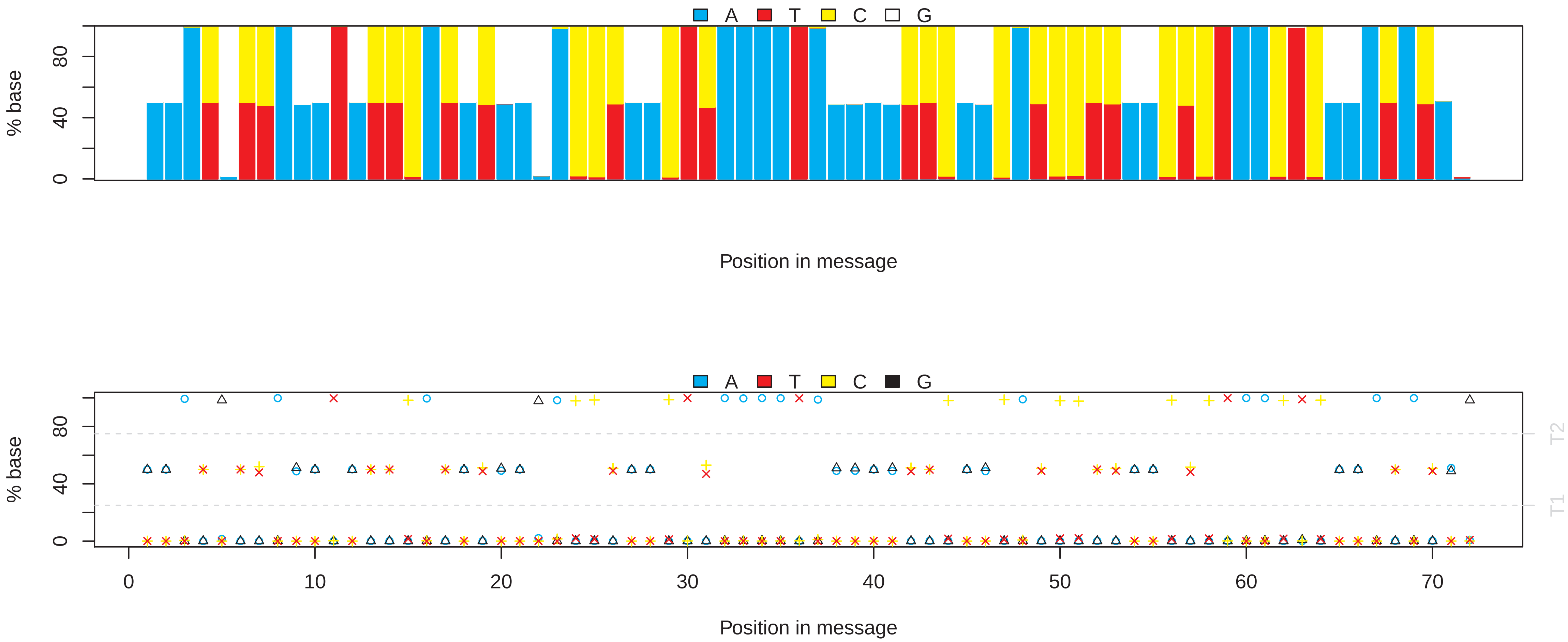

000111100010110010100100010001100001000001100000100110001010001000010101

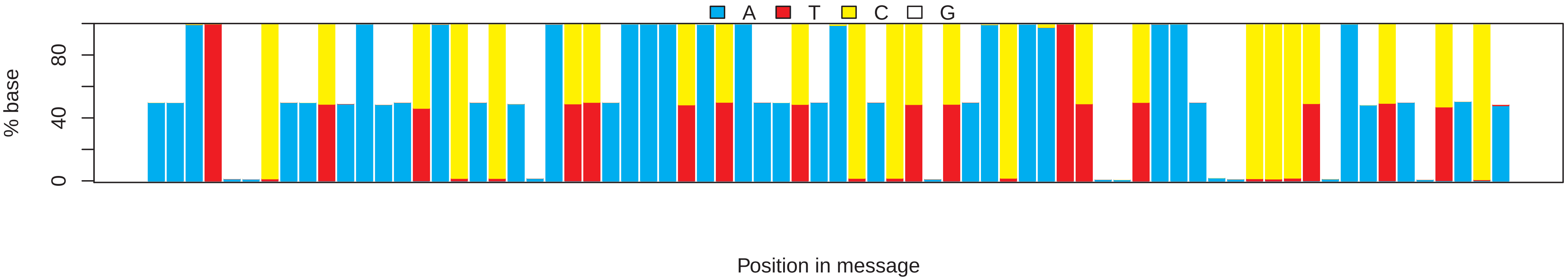

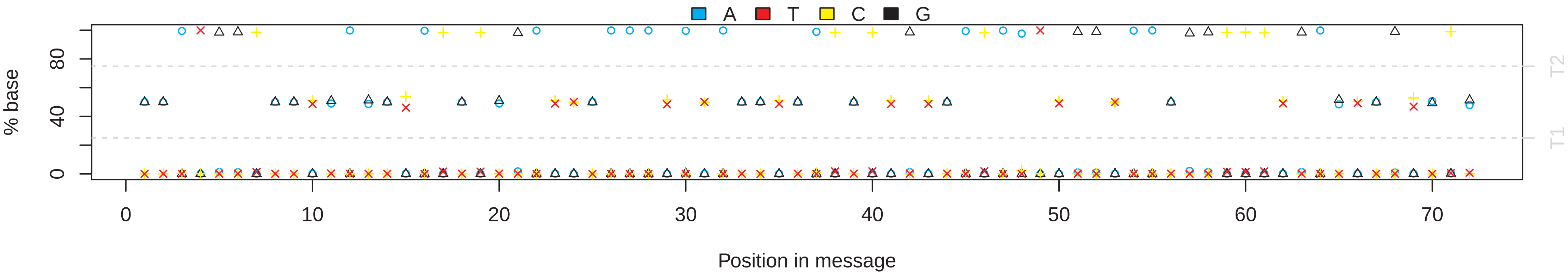

000111000100001000001011000010100010000011100000111110001100011001011000

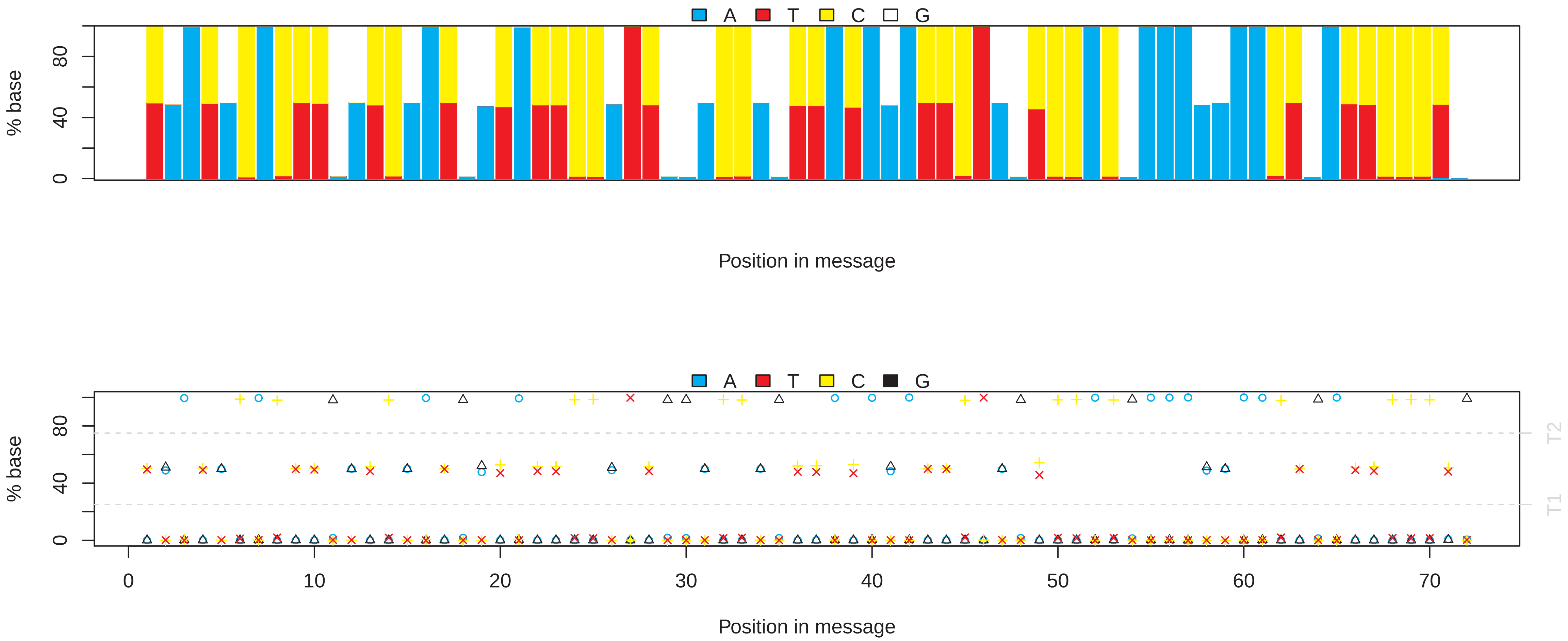

1001000011101000110101100011110000111010001101011000010000000001101100011

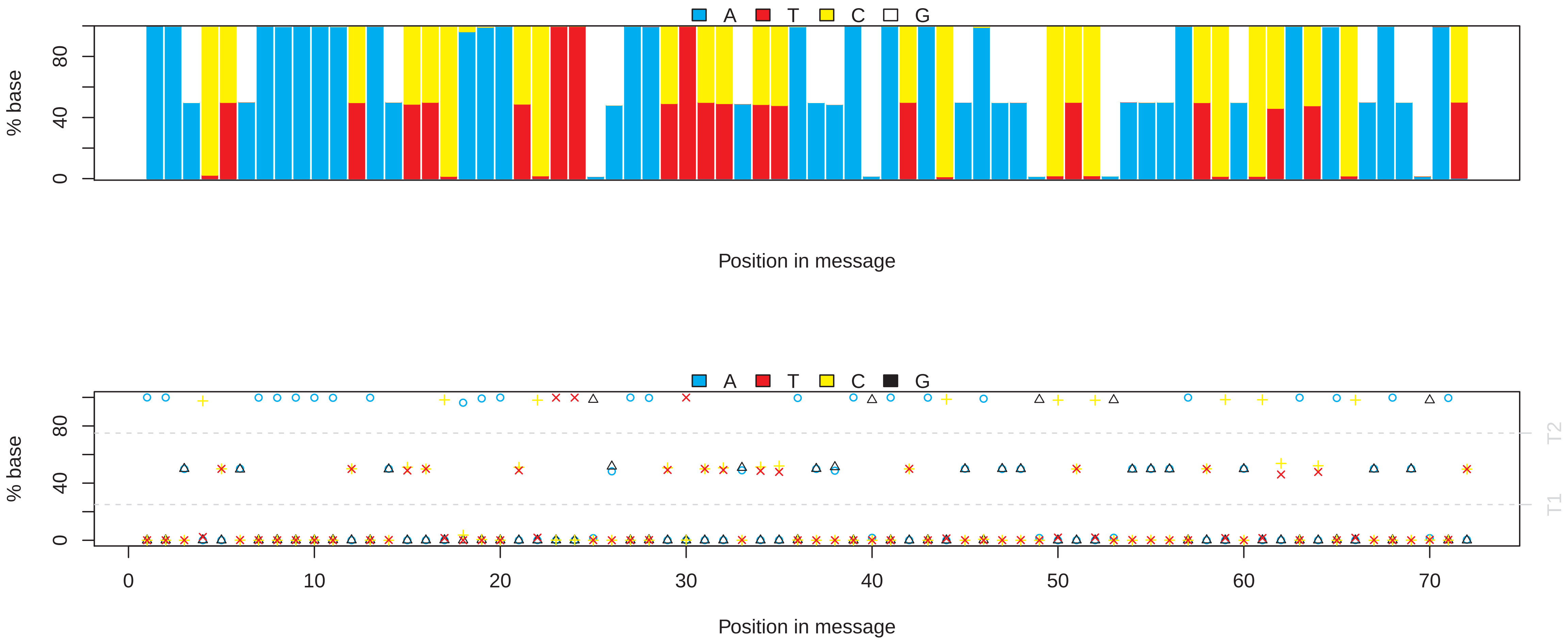

0000100000001001100001011100011110110000101000000101010000100010100000101

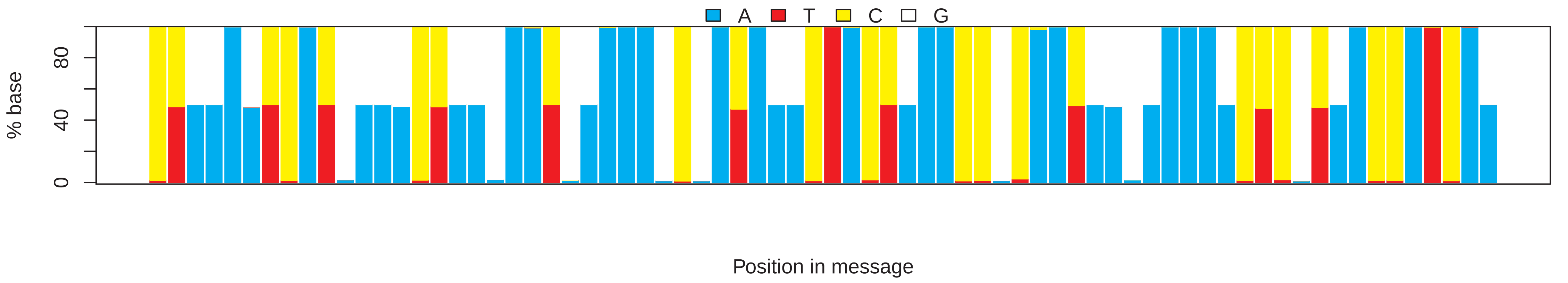

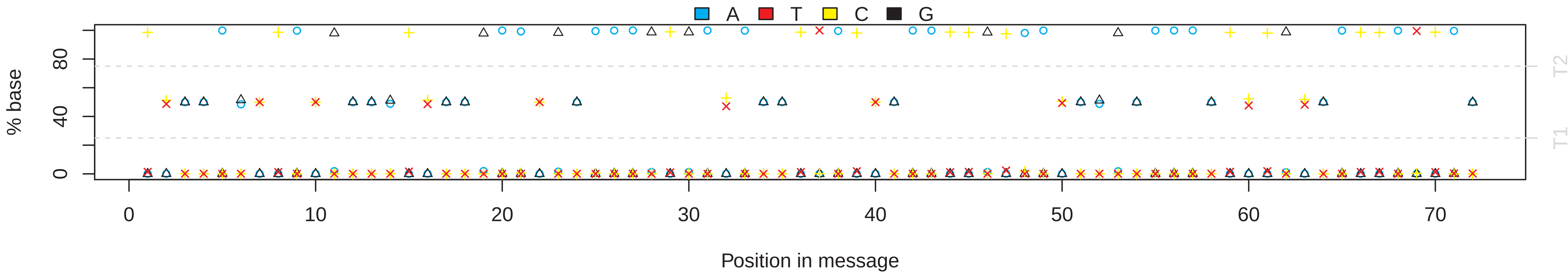

0100001001100001001001100001010100001001000001000100100000001011000001000

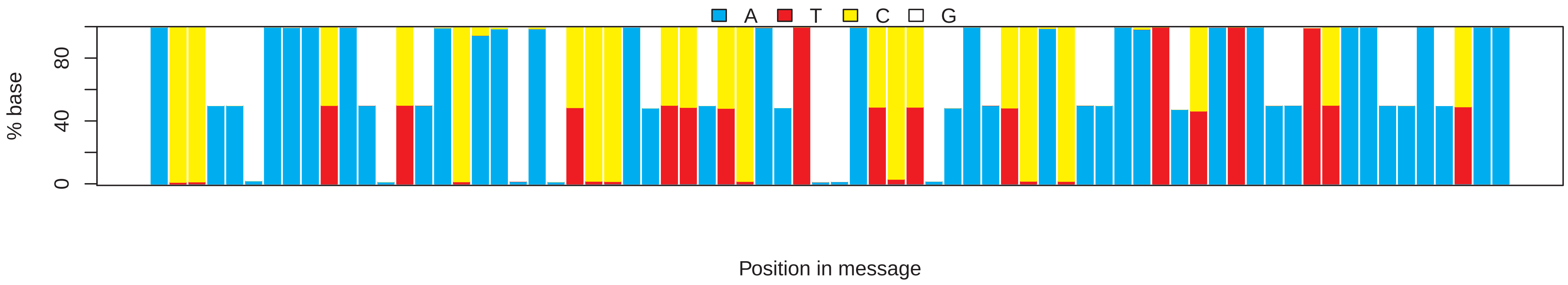

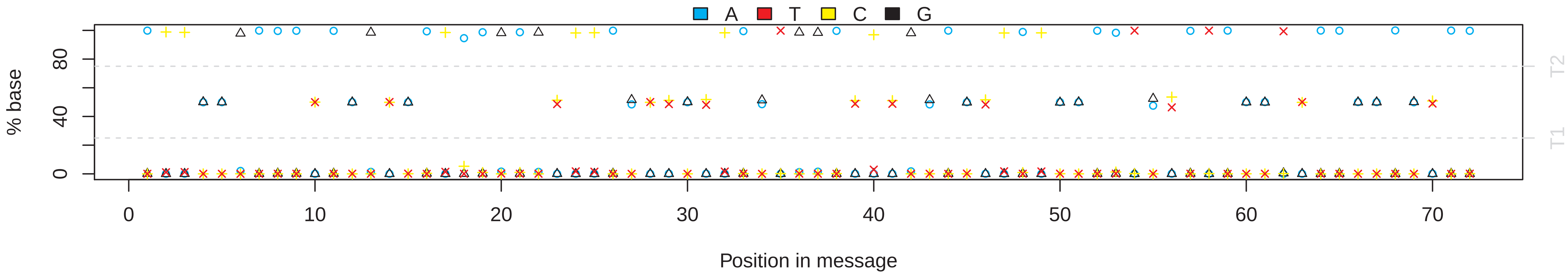

0000010001001100000010110000110100011101011000100000001010100011000000100

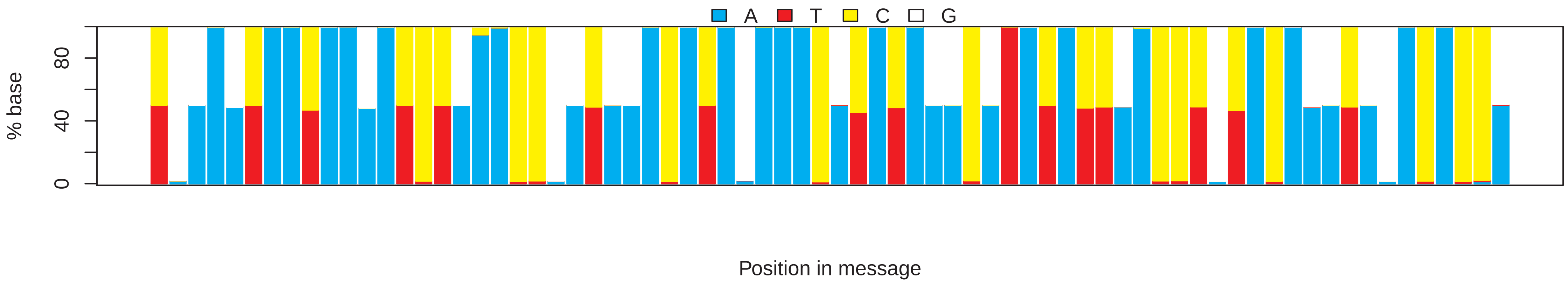

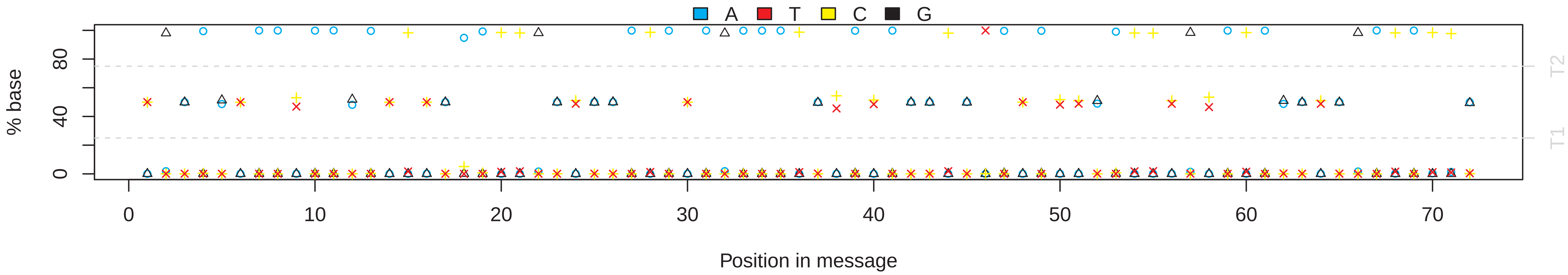

110001001000010100000101000001010000010100000101011000011100000101000000

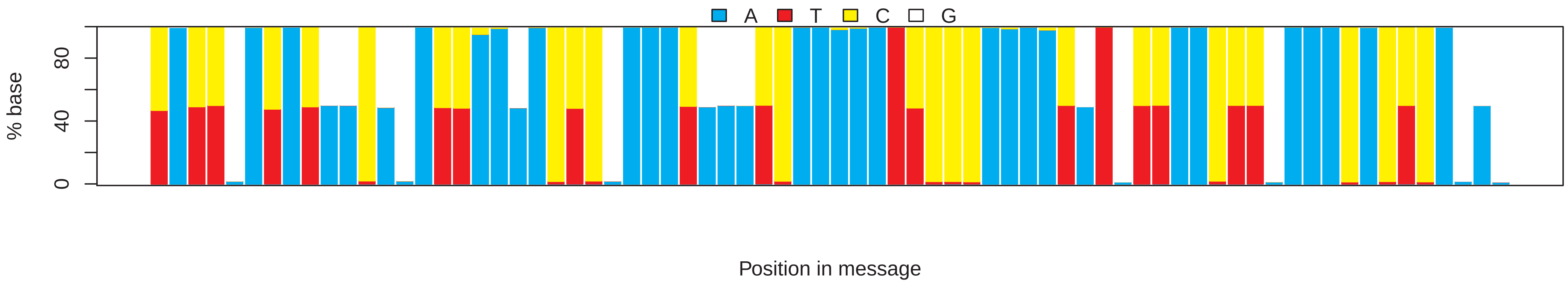

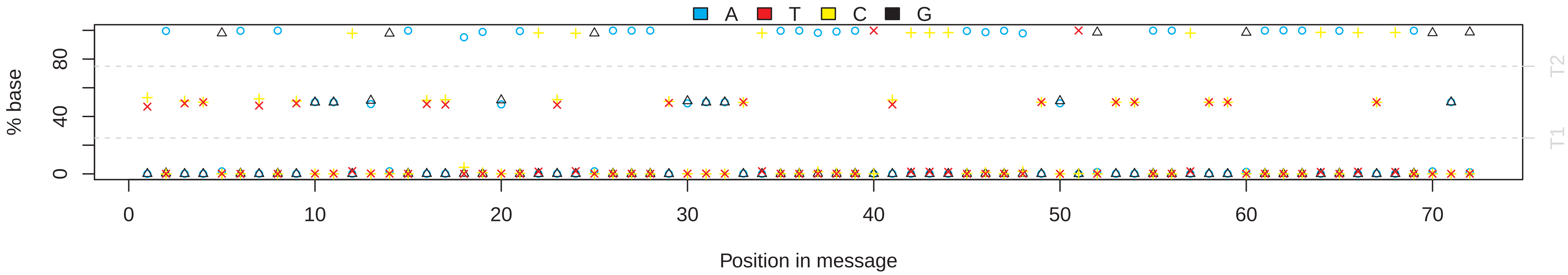

1011101010000101100000101000100010000001100000001011110001110000000100101

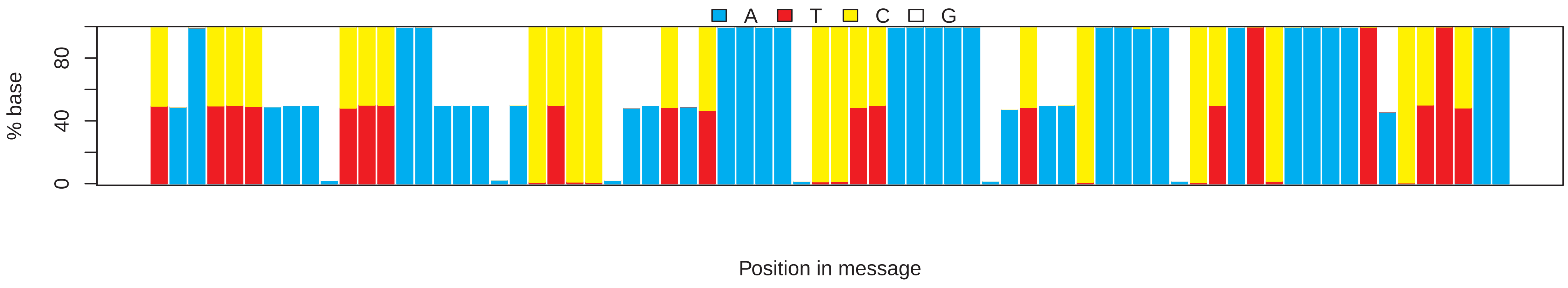

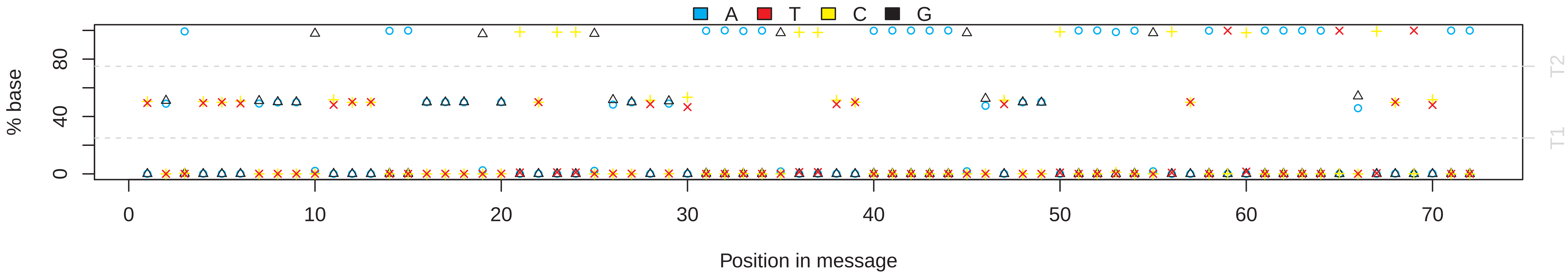

1001110001111000001001001001010000100110000010100000001010100000010011100

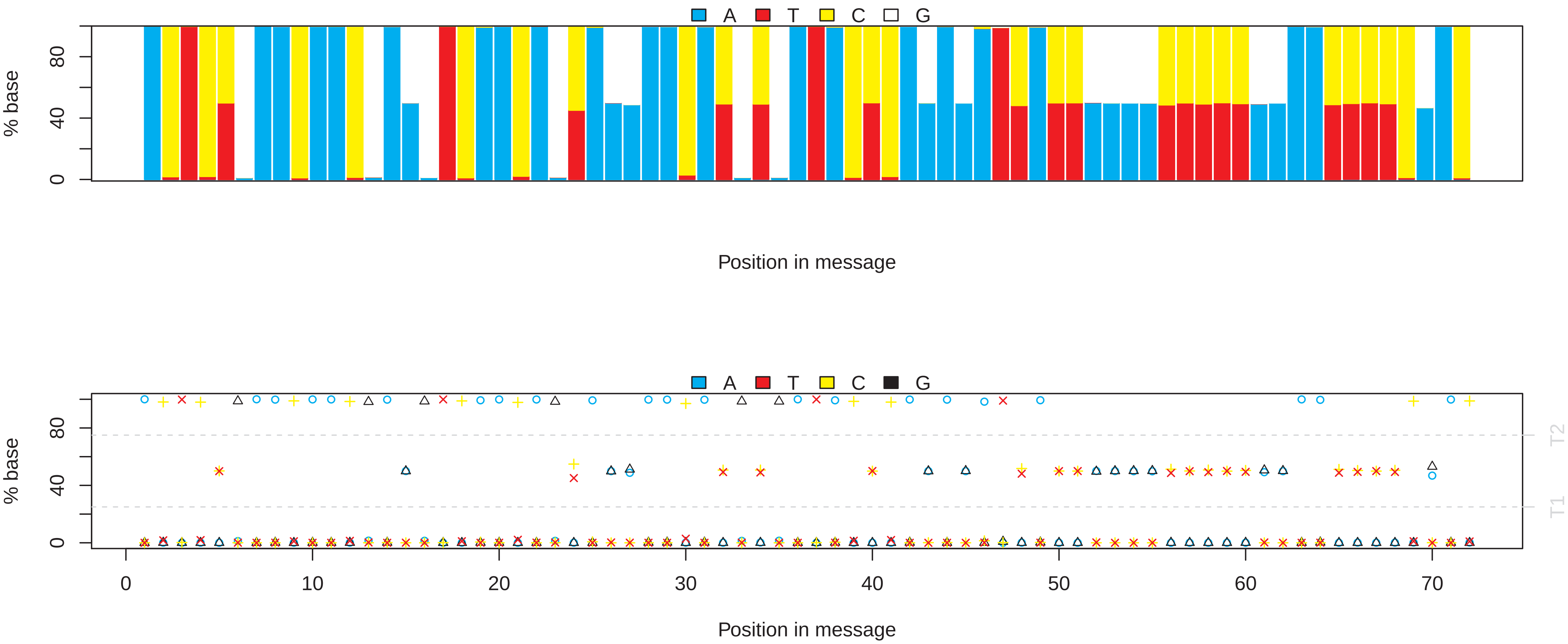

001011000000100110000011000000011110100100000011011000011111000011110000

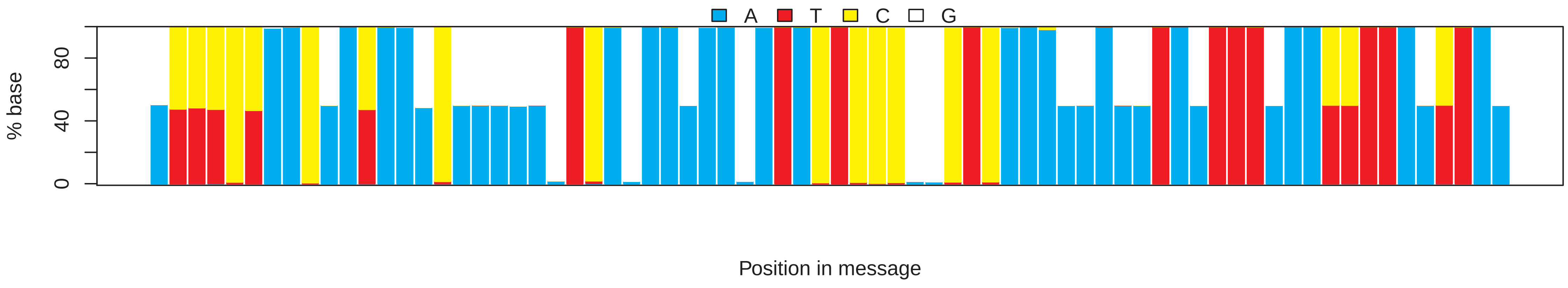

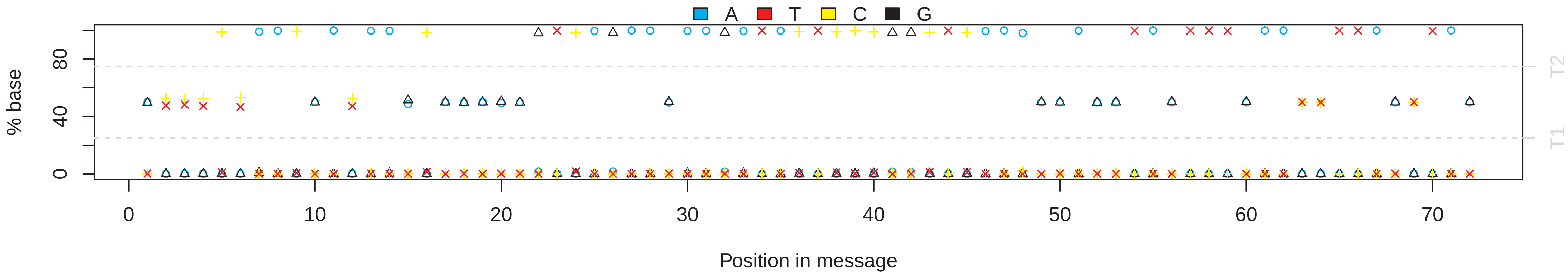

011101000001000000000110010000010100100011010000000001001110001111001100

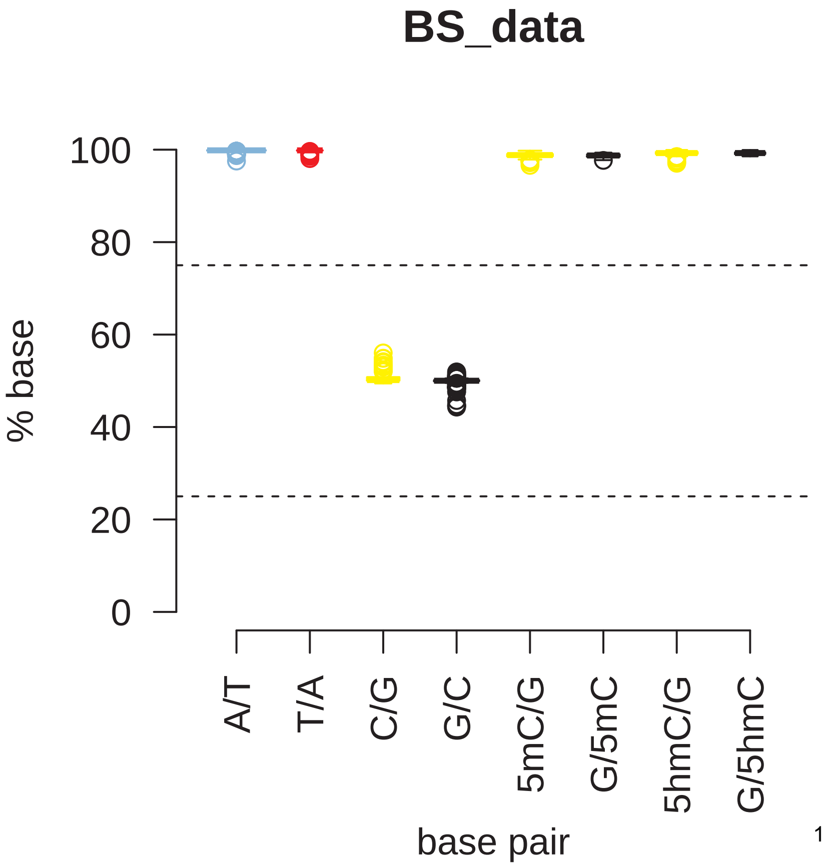
BS_data
% base
100
80
60
40
20
0
A/T
T/A
C/G
G/C
5mC/G
G/5mC
5hmC/G
G/5hmC
base pair

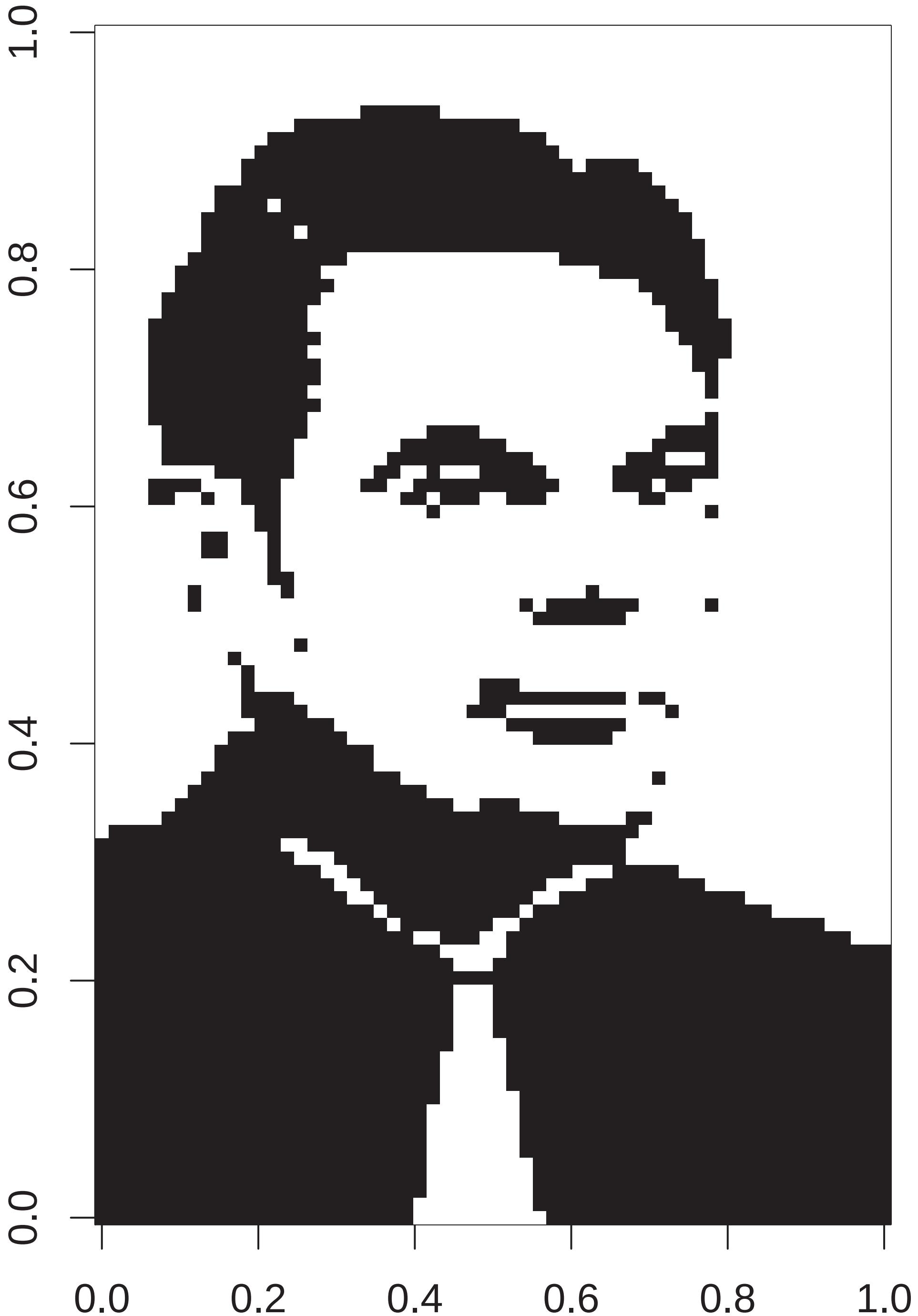
0.0
0.2
0.4
0.6
0.8
1.0
0.0
0.2
0.4
0.6
0.8
1.0

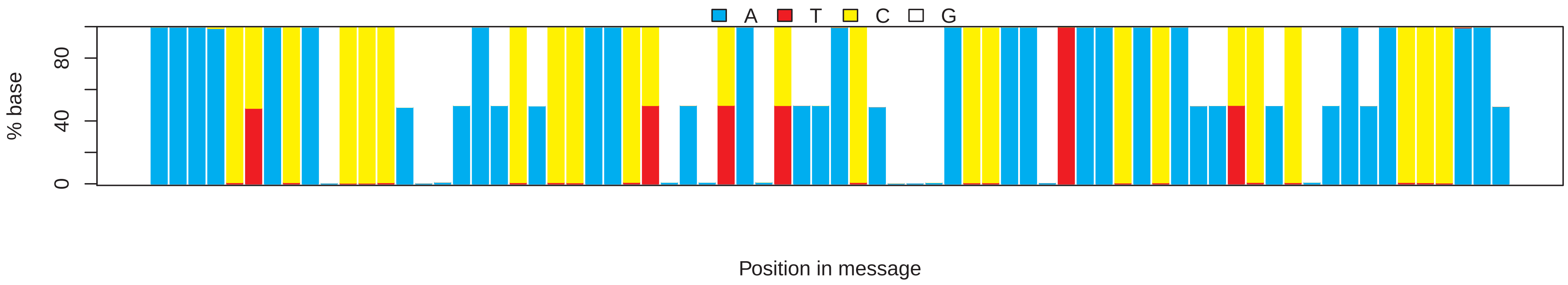

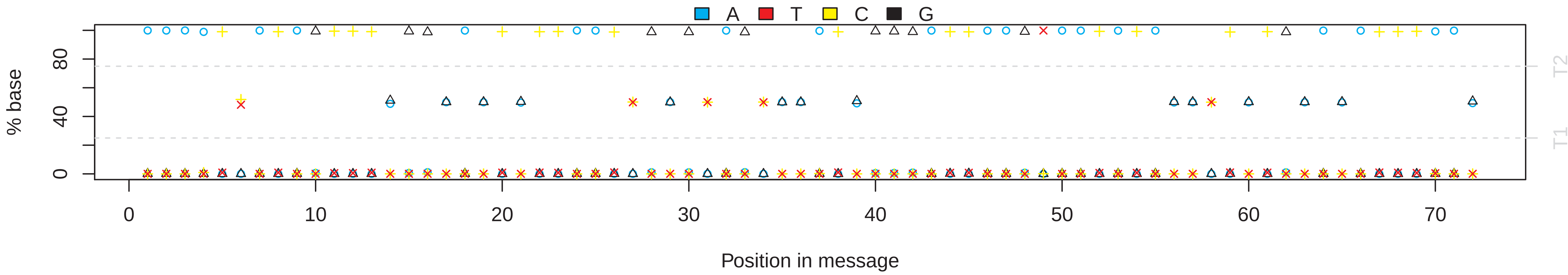

000011010000101000000110011000100100000110011000100000000110100000100000

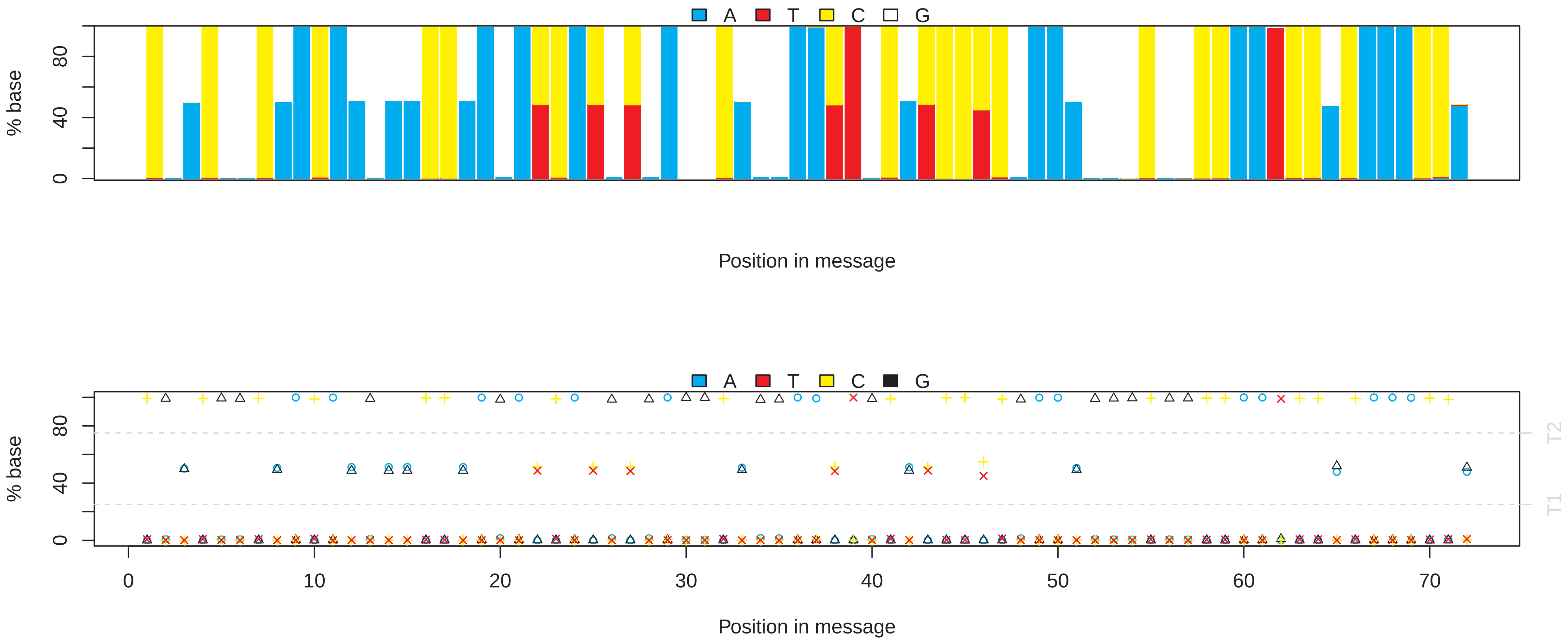

000110100000100000000110101001110000011010100111000001101010011100000110

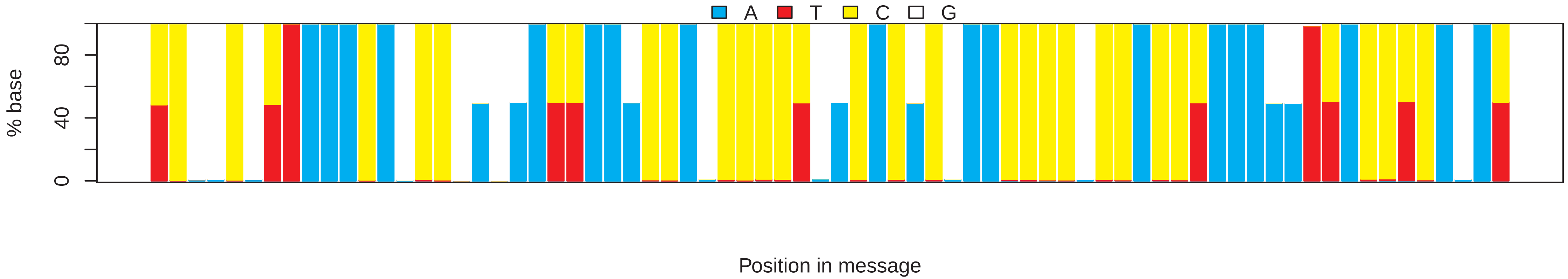

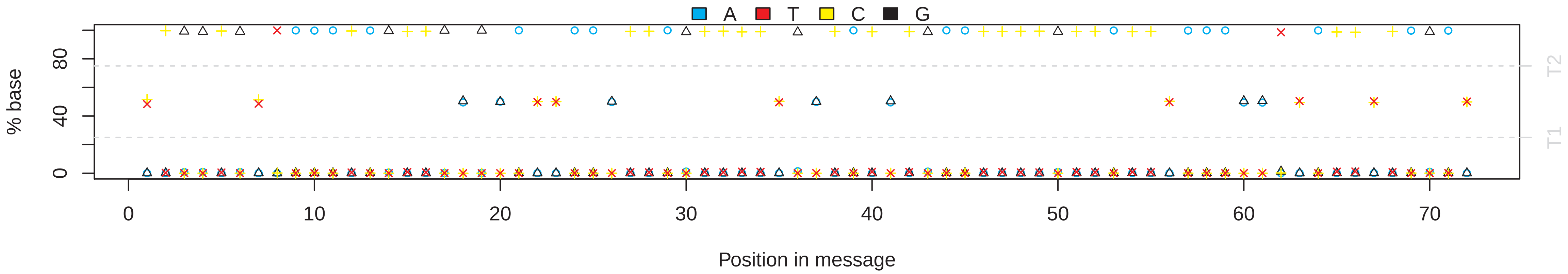

101001110000011010100110000001101110010100000110111001010000011011100101

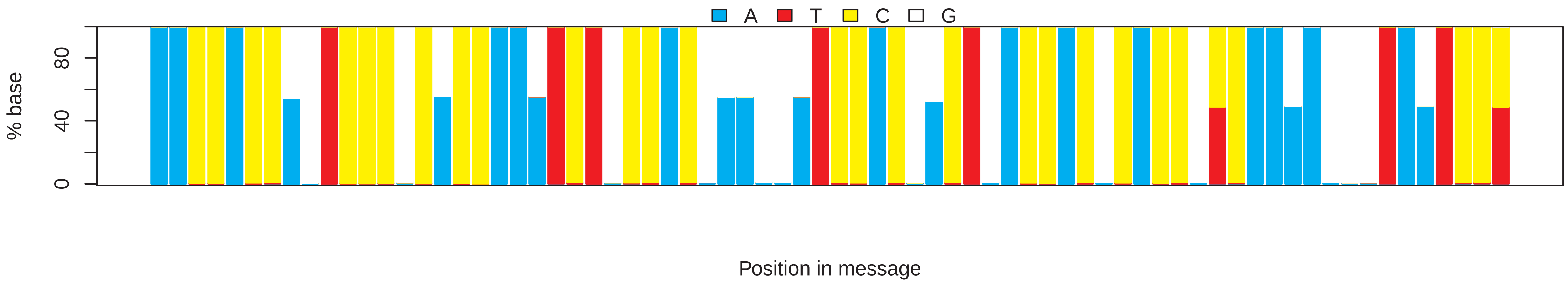

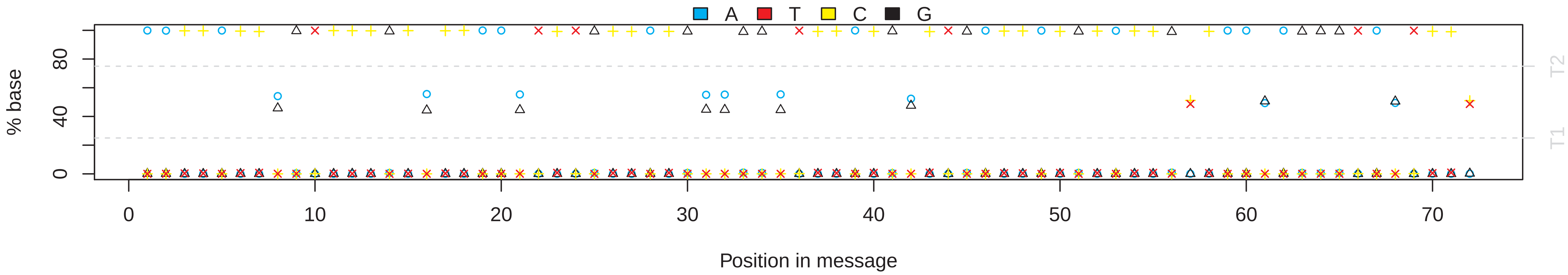

000001101110010000000111001011000001110010110000011100101100000111001011

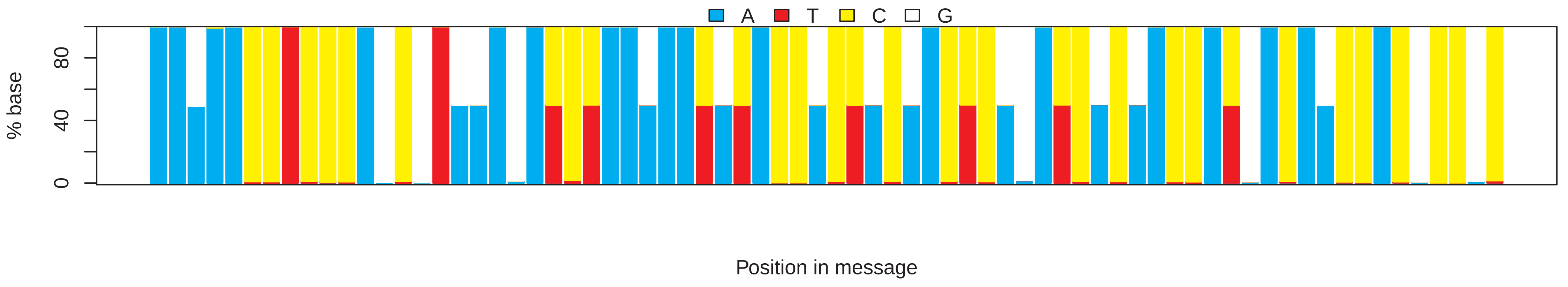

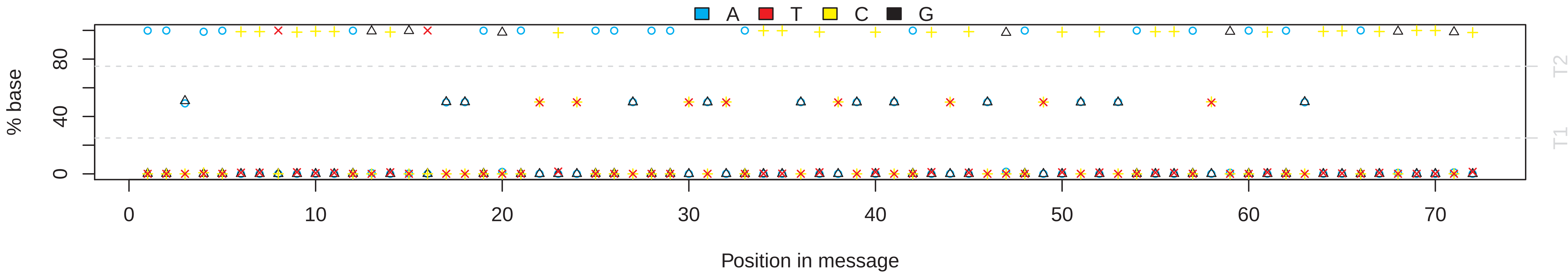

000000111010101100000111000001010000110100110000110100100110100000110000

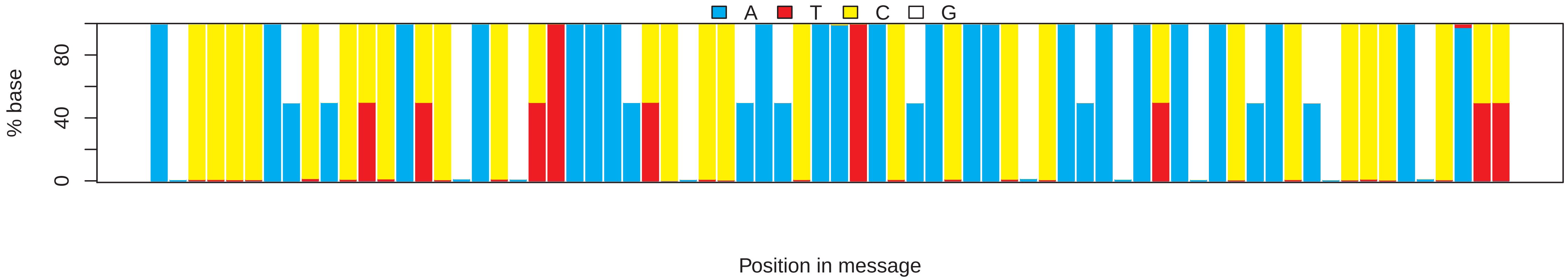

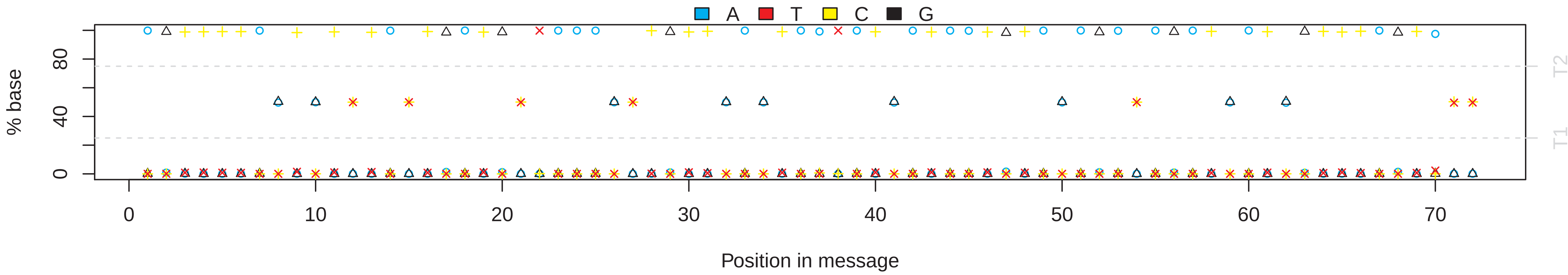

0101000010111010001111000010110000100100001001010001010100001010100010111

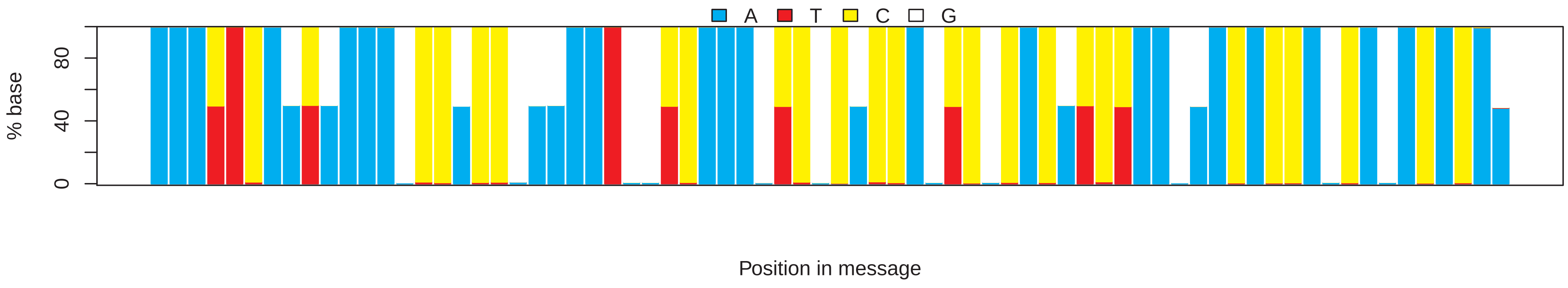

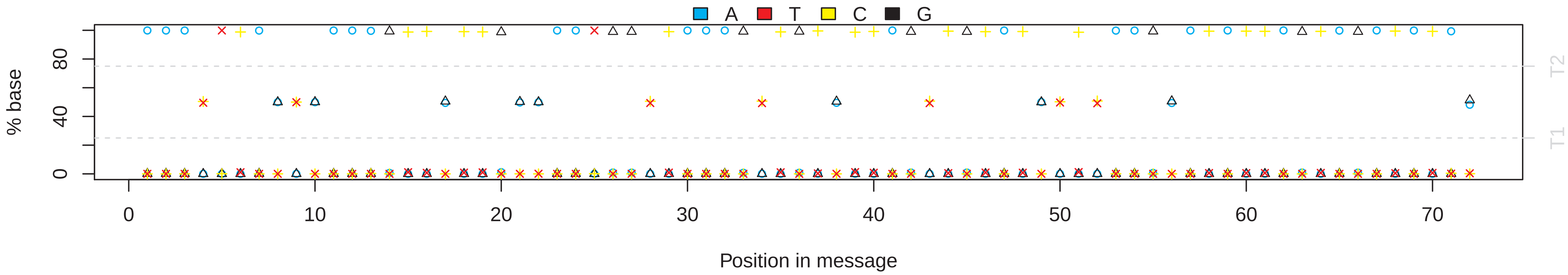

0001110010000110001000001001100011100001001011000111001000001001000000100

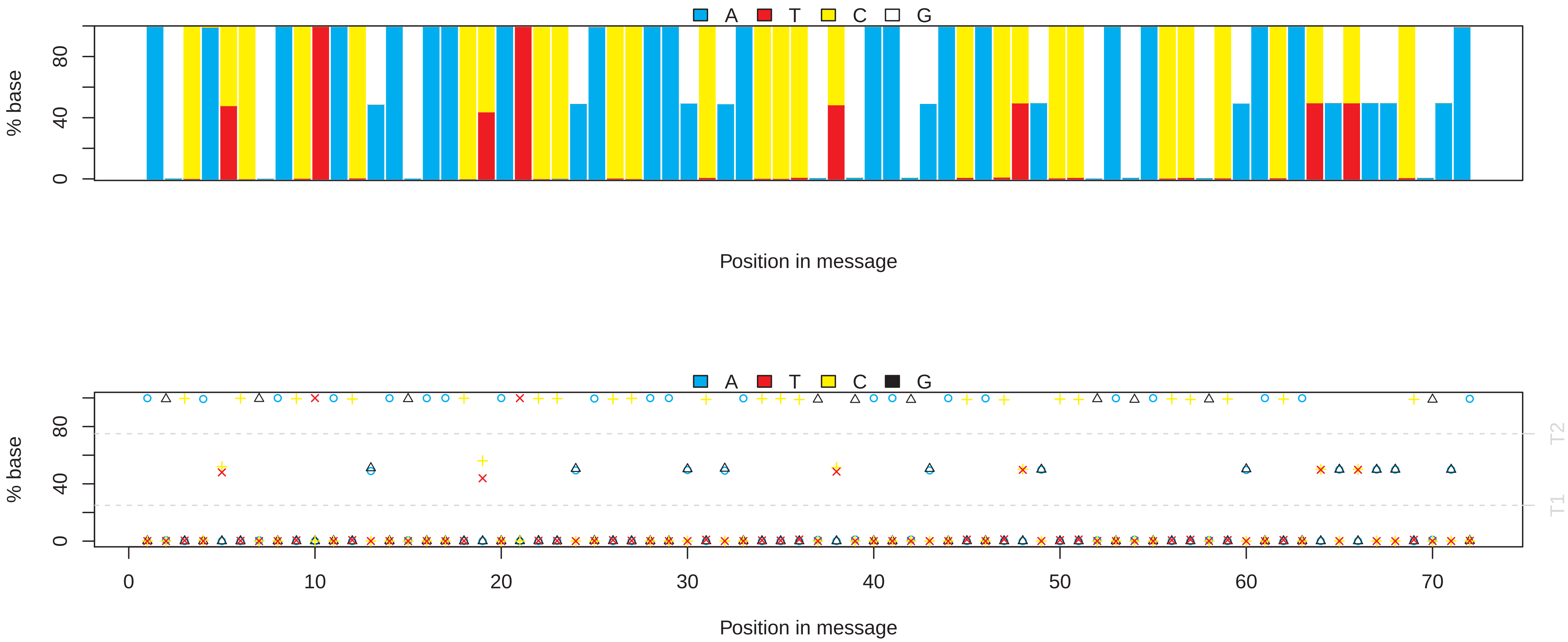

000010101100001000101000001000100001010000001011001100011110000101000000

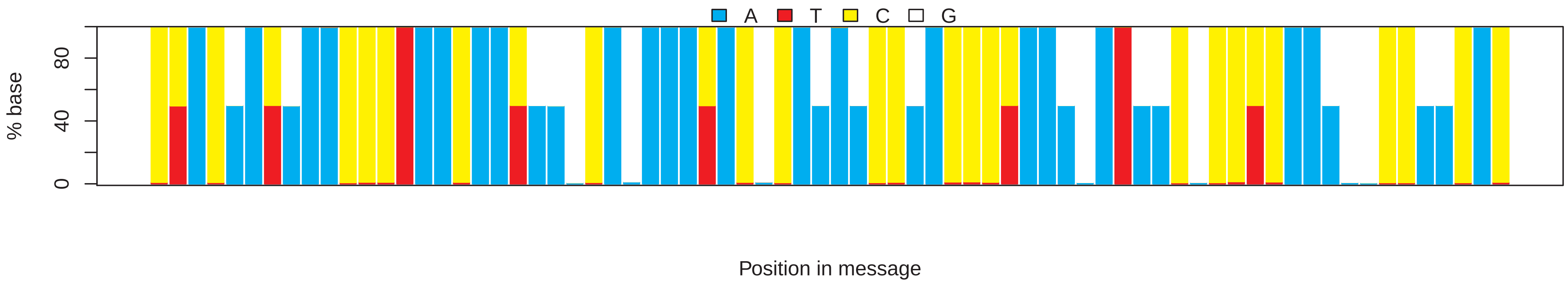

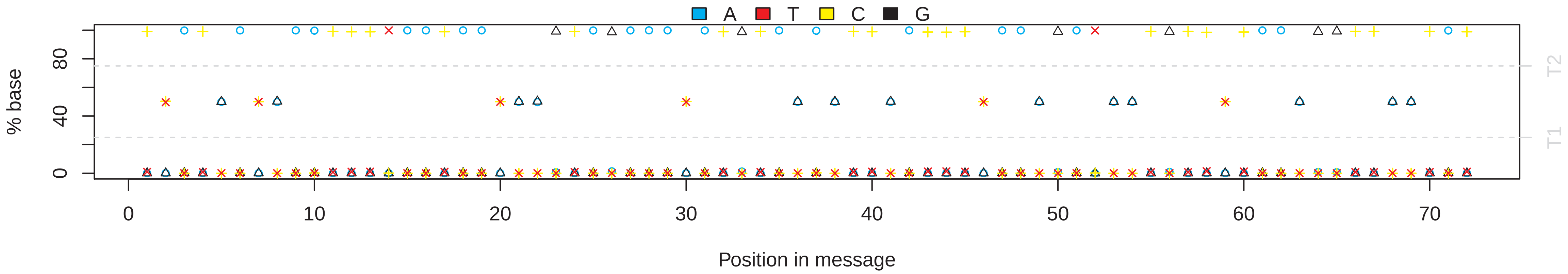

11000010000111000000100110000001010000000000001001101000101000011110000000100001

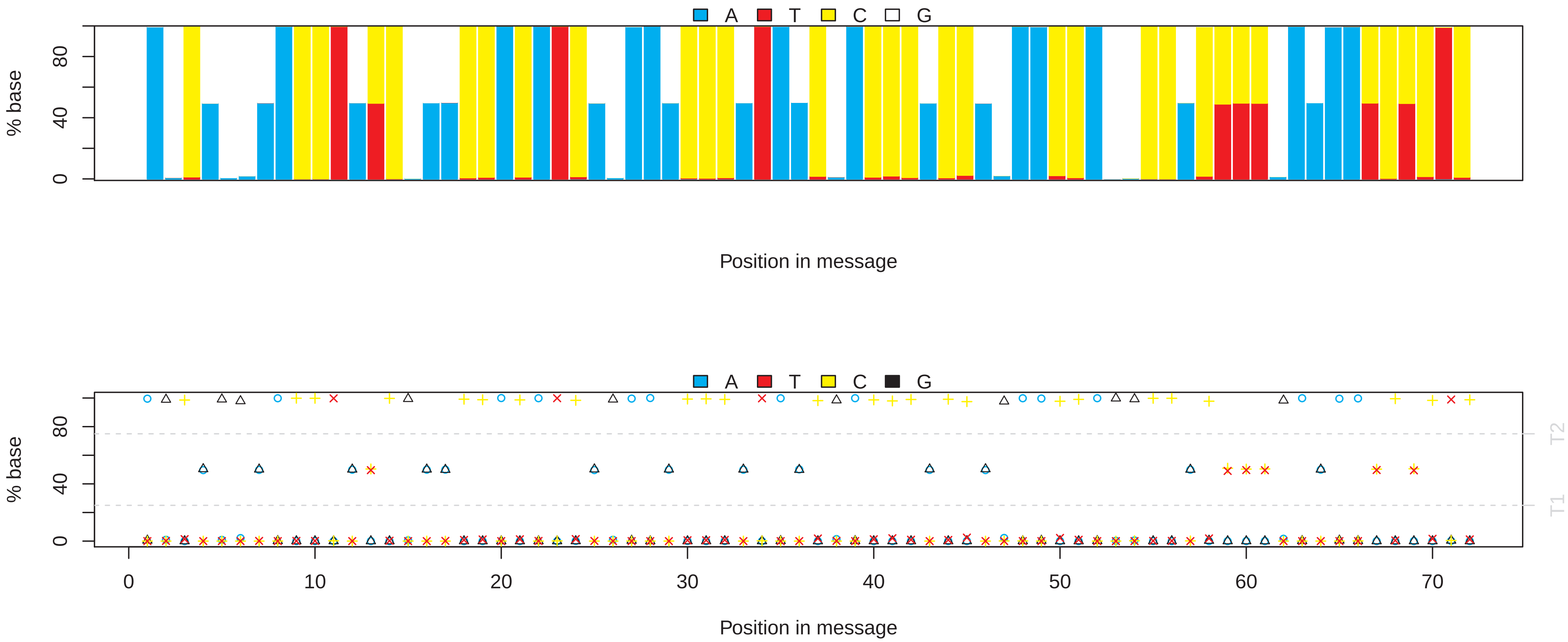

0110100000101010000010100100001001000001100010000100110001111100000101111

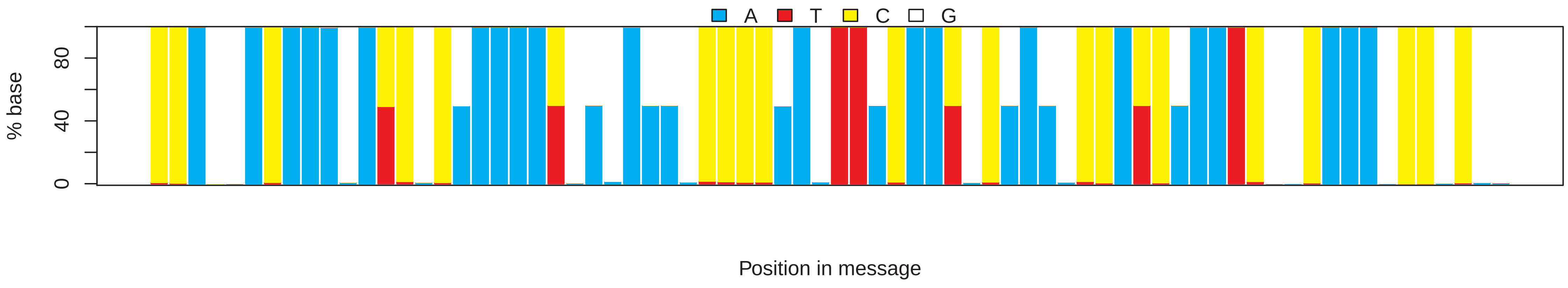

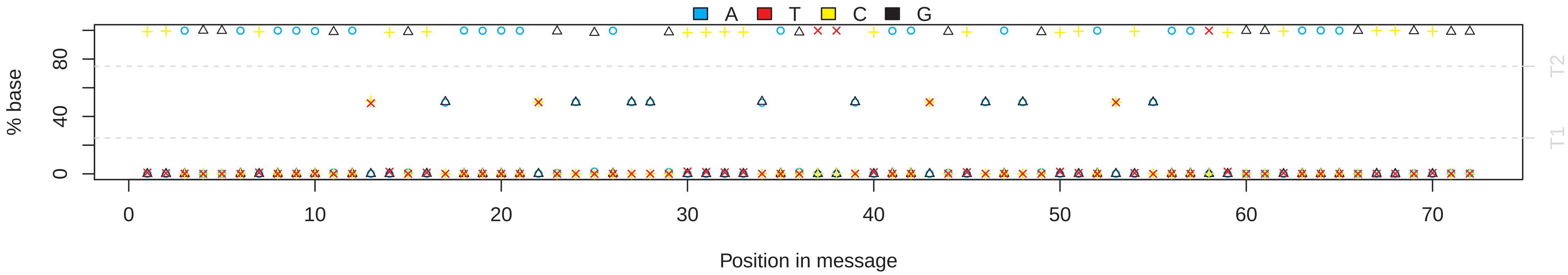

0001100000001100000000110000001111000110000111000010010000111100001001000

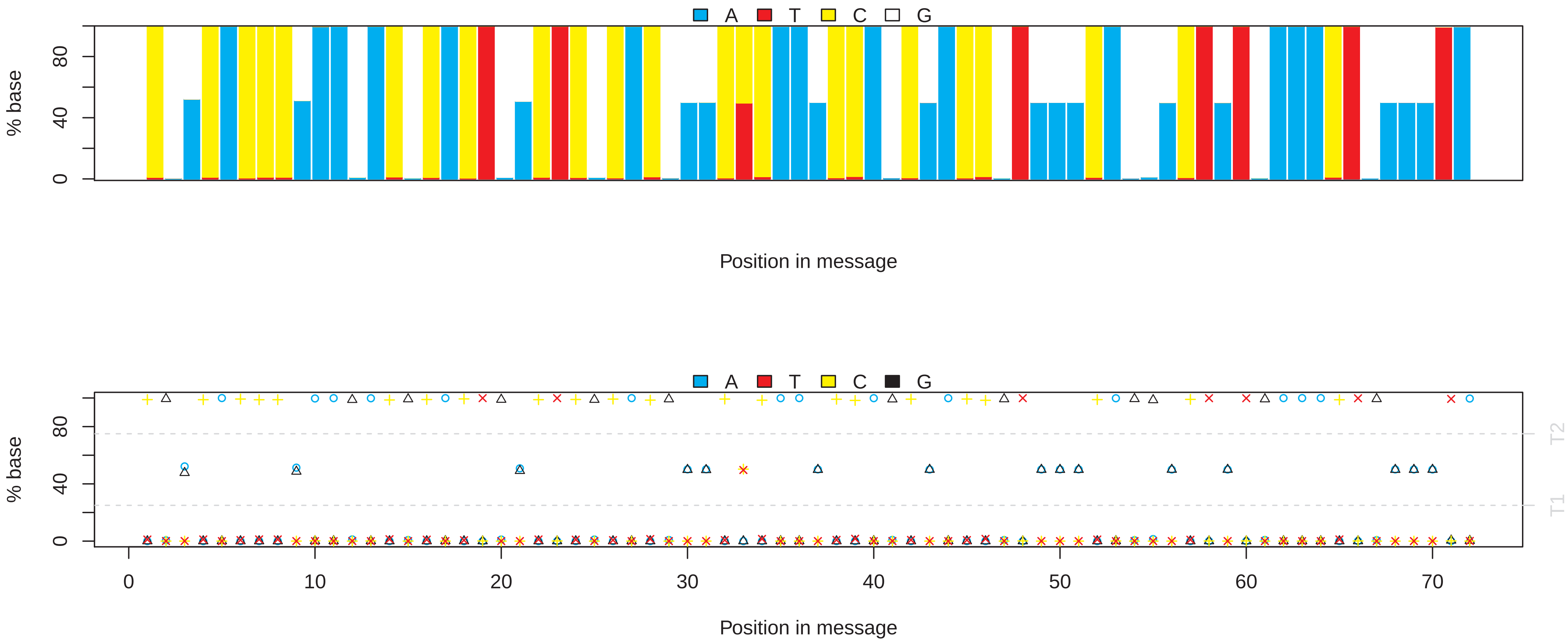

110100110000011100100011000110001100001010000111000101000101100011100010

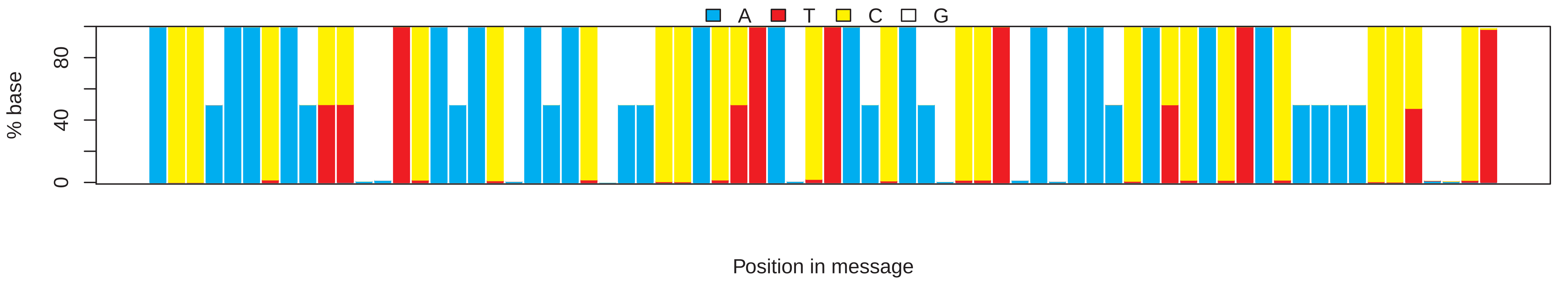

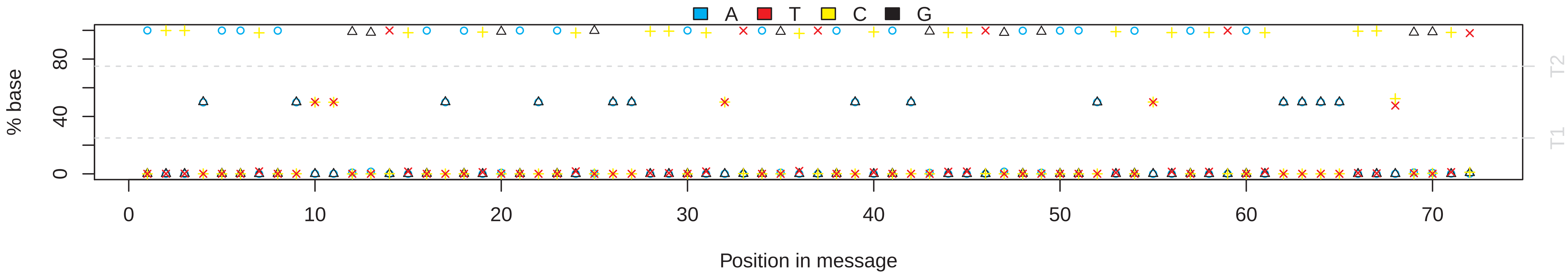

000000100111011000010001100000111011100000111100100000110110100000010111

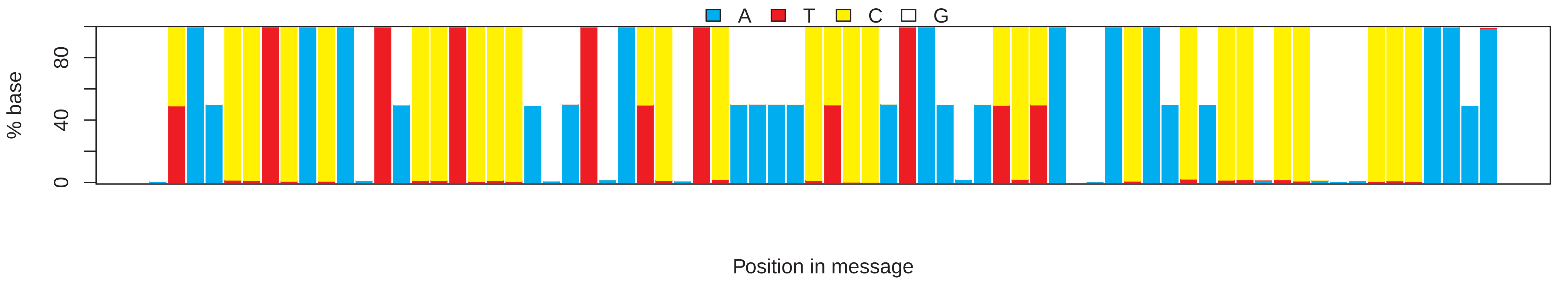

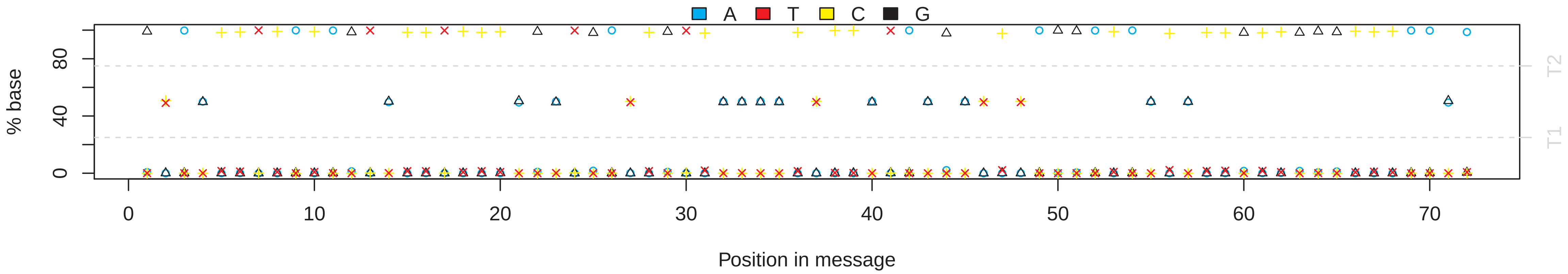

110011100000101110100101001111100001100010000111011000010110100110100000

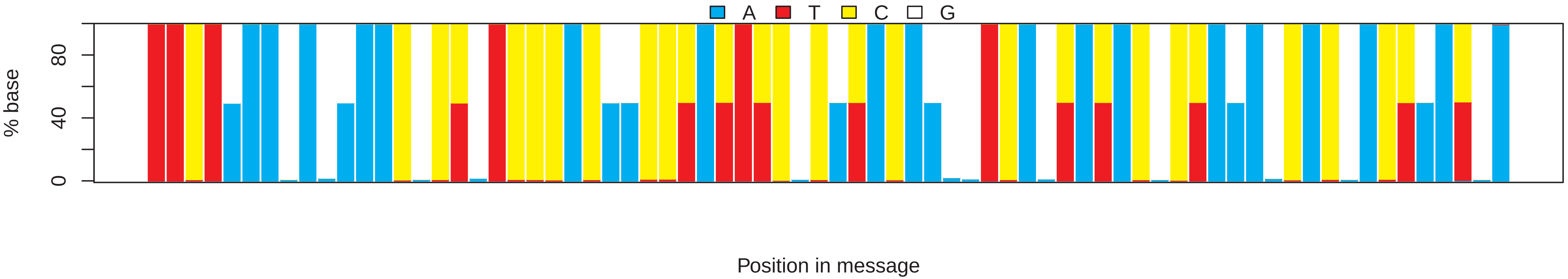

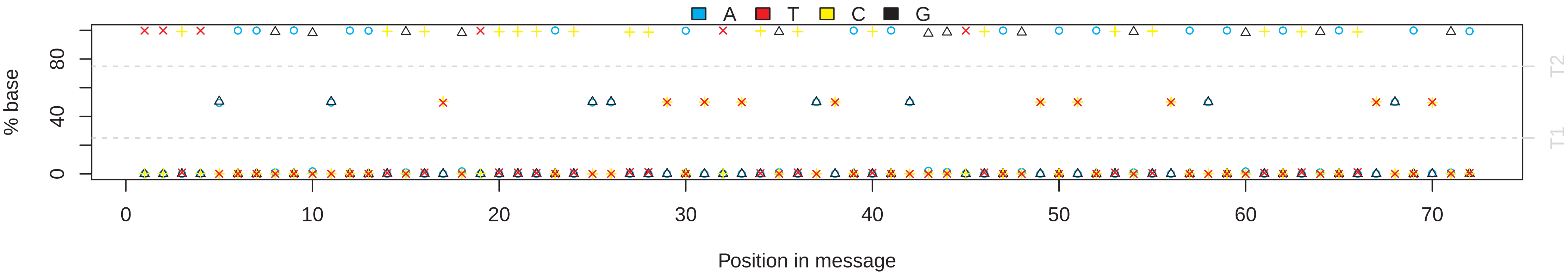

110100010000001110111000001110111011010000001101101011010000001101100100

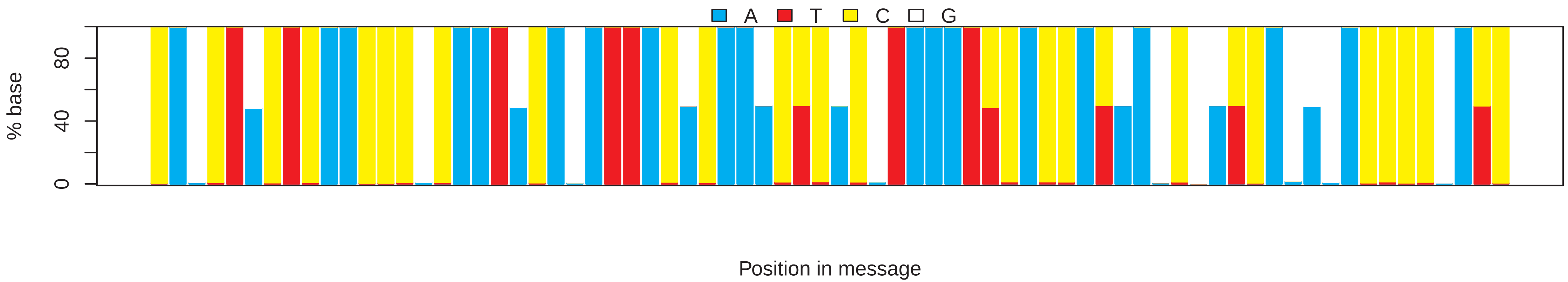

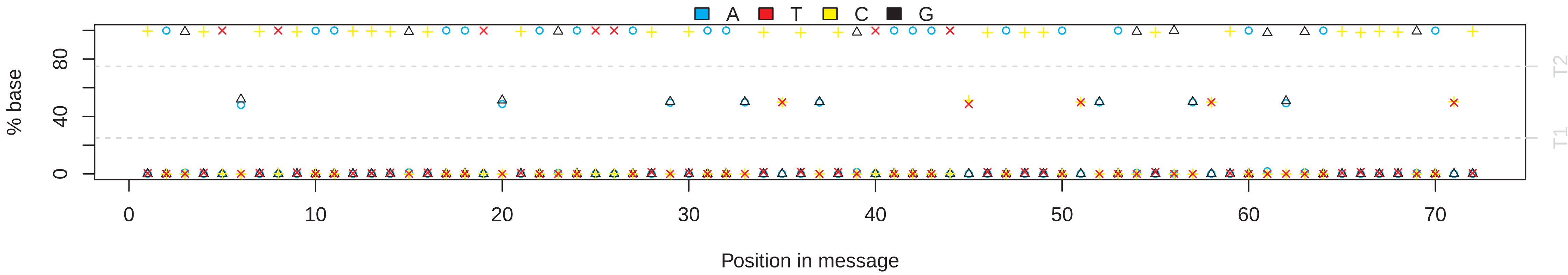

000110011000010100100010110100000111010100011101101001110100001001011010

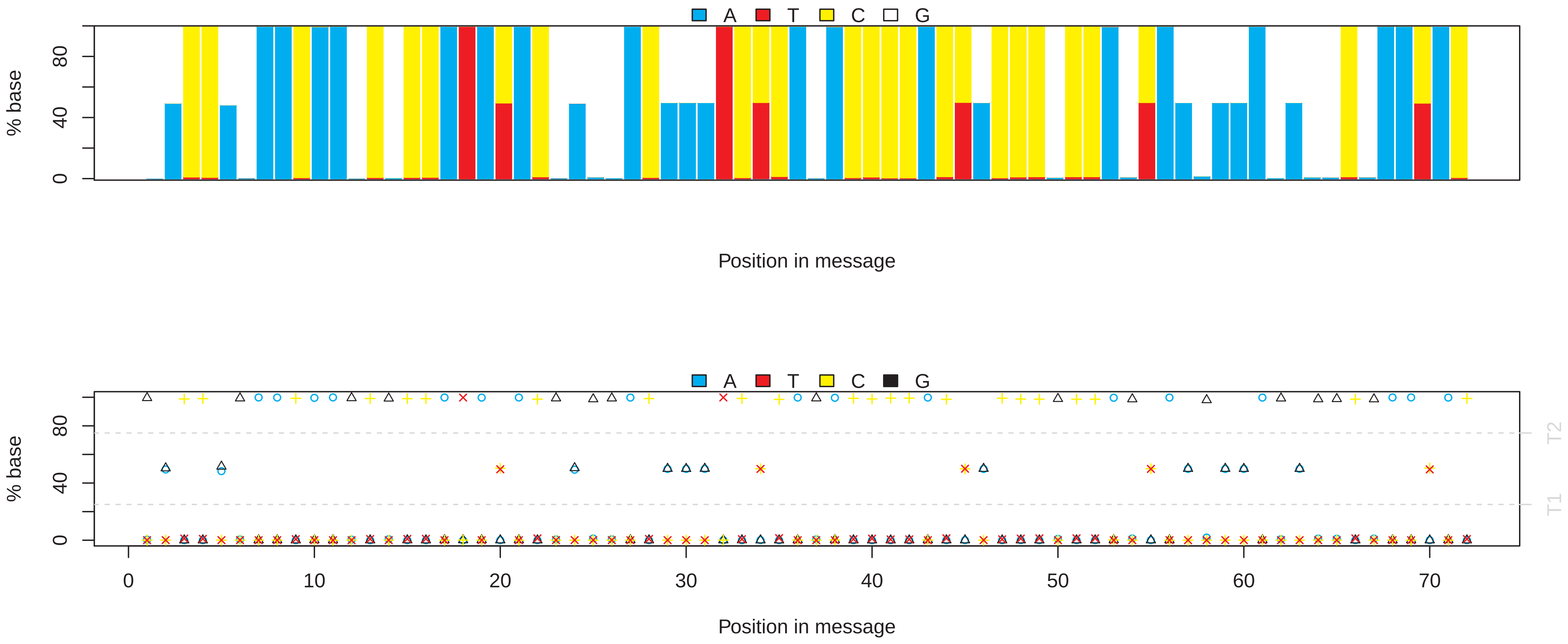

1010010000010011010101100100000101101001000110011011001000000010001000100

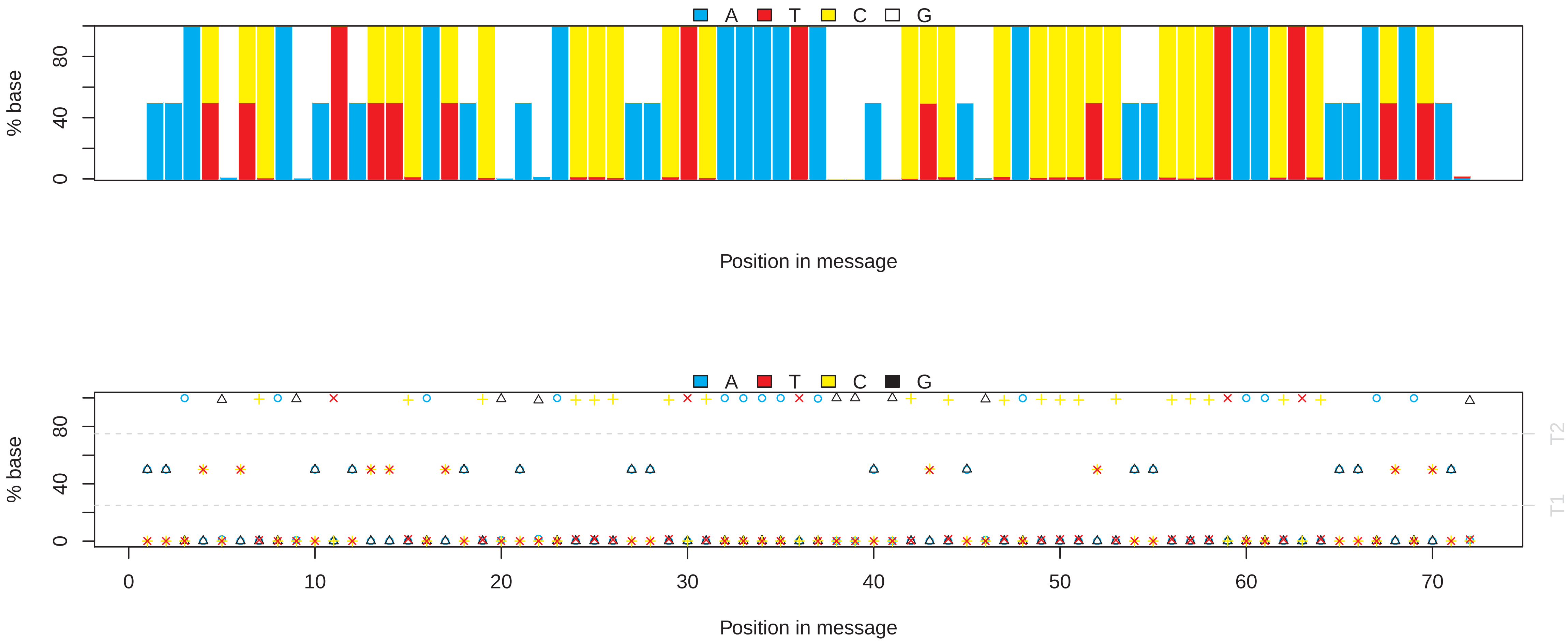

0001010010101110100100011000110000010110101101100111000101100111000010100

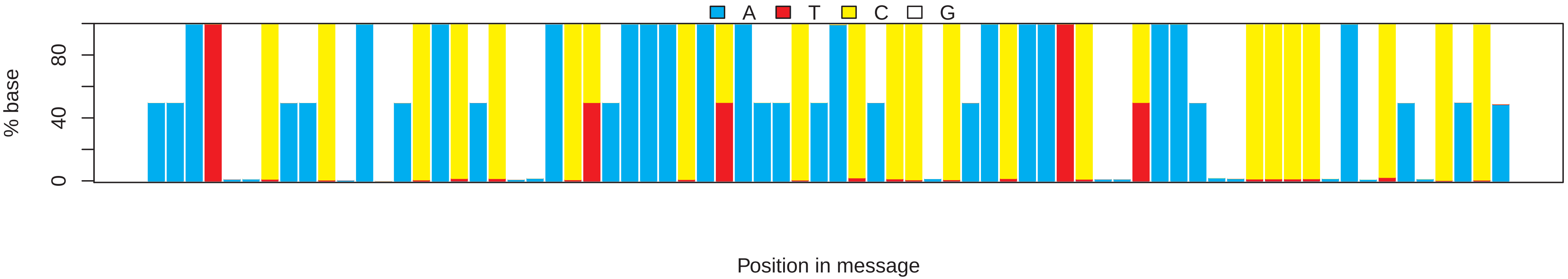

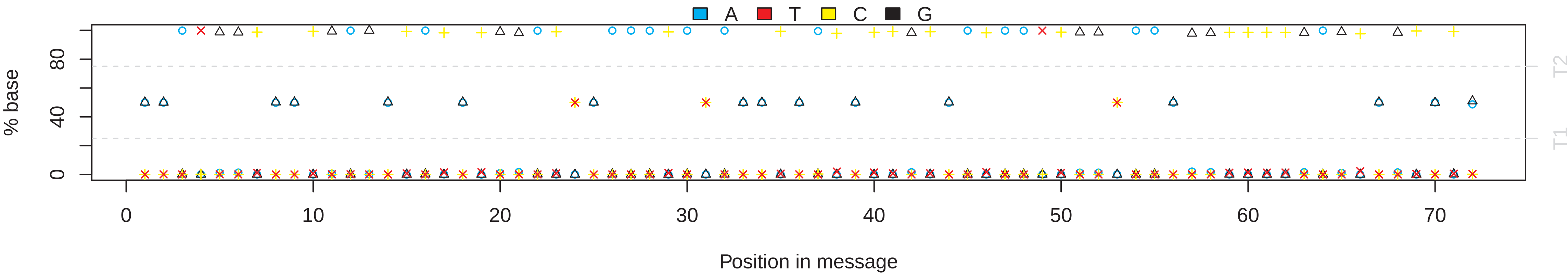

000100100010100010110001000000100000010100000100100010000011100010000010

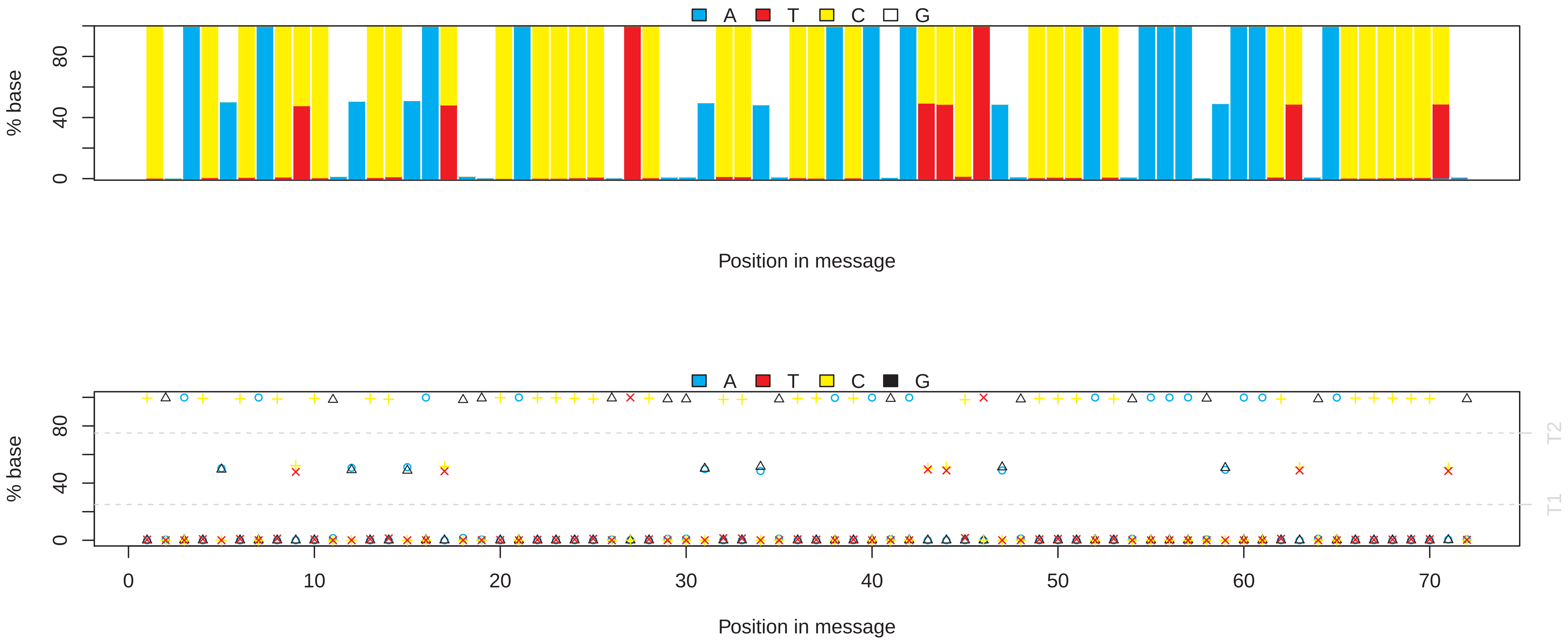

0100010110000100101000011110000110000000101111000110100001000110000011110

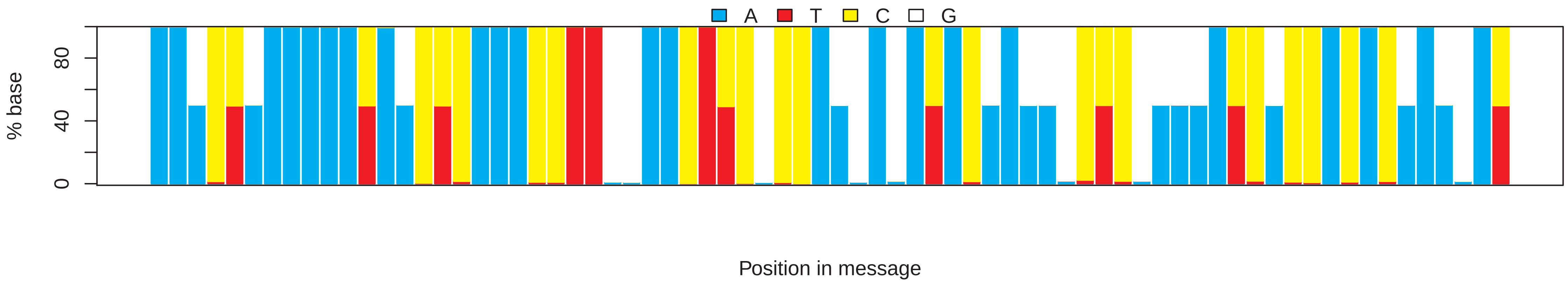

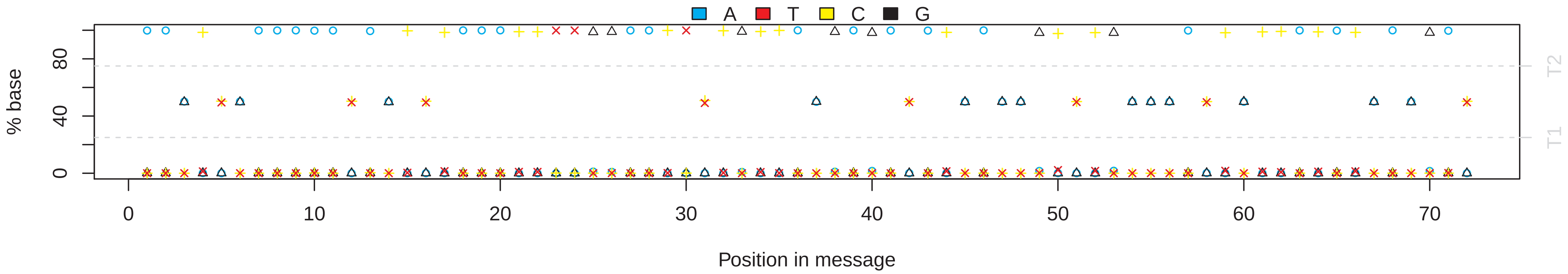

0001100000010001100001110100011010000100010100000111000000110100001000001

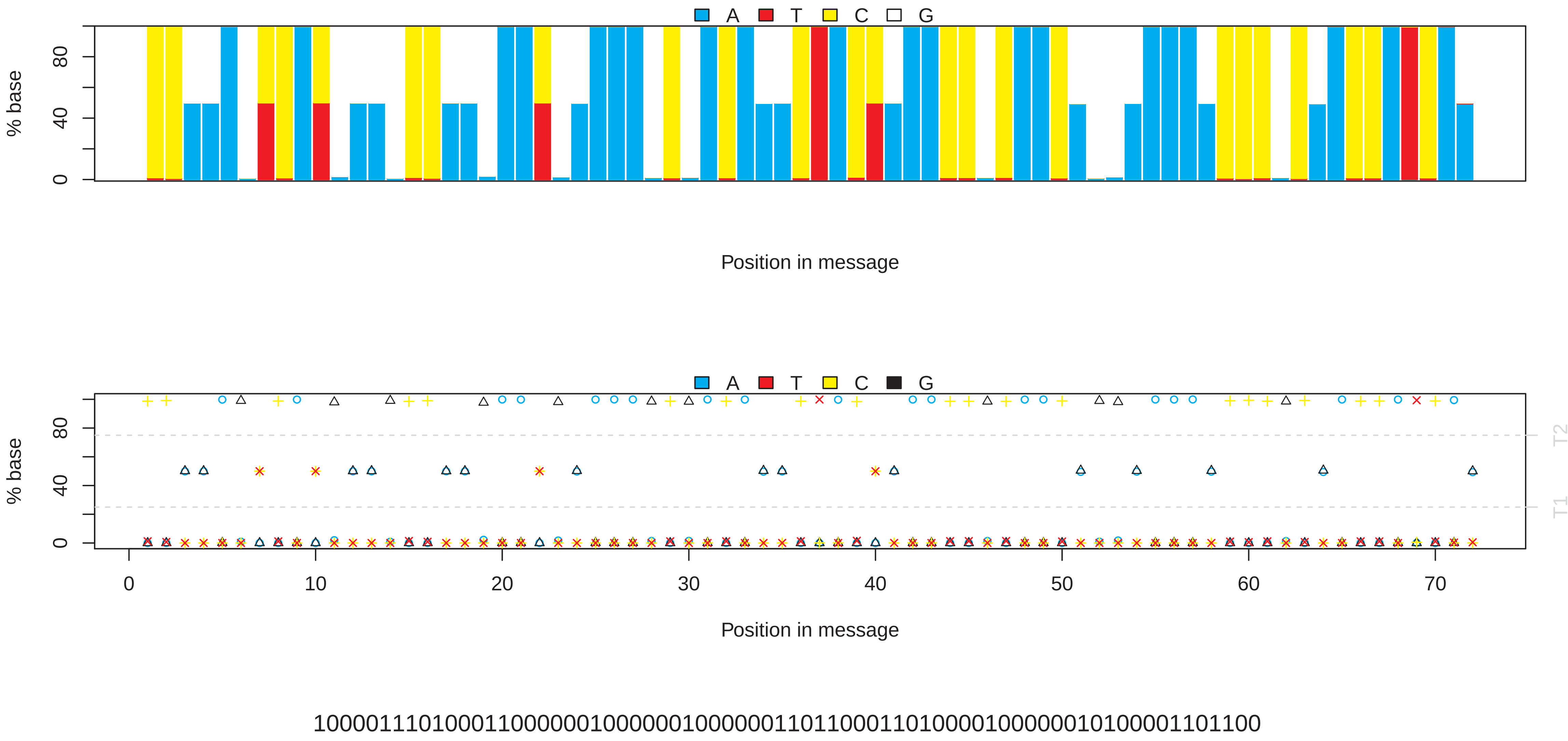
A
T
C
G
% base
0
40
80
Position in message
A
T
C
G
% base
0
40
80
T2
T1
0
10
20
30
40
50
60
70
Position in message
100001110100011000000100000010000001101100011010000100000010100001101100

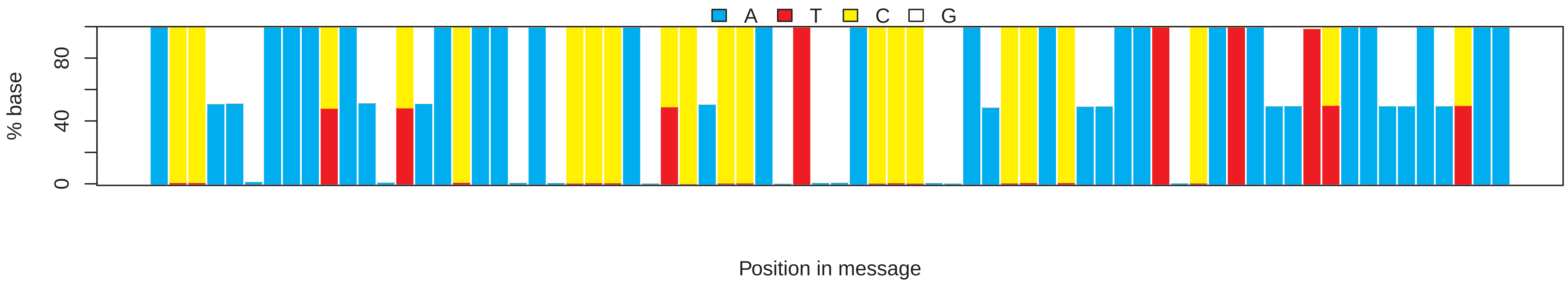

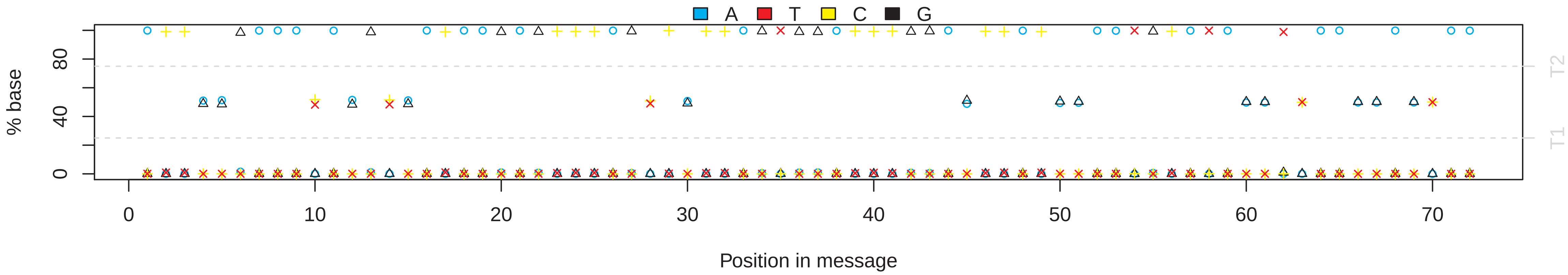

0110000001000100100000011011000101100001001000101000011001000110000000100

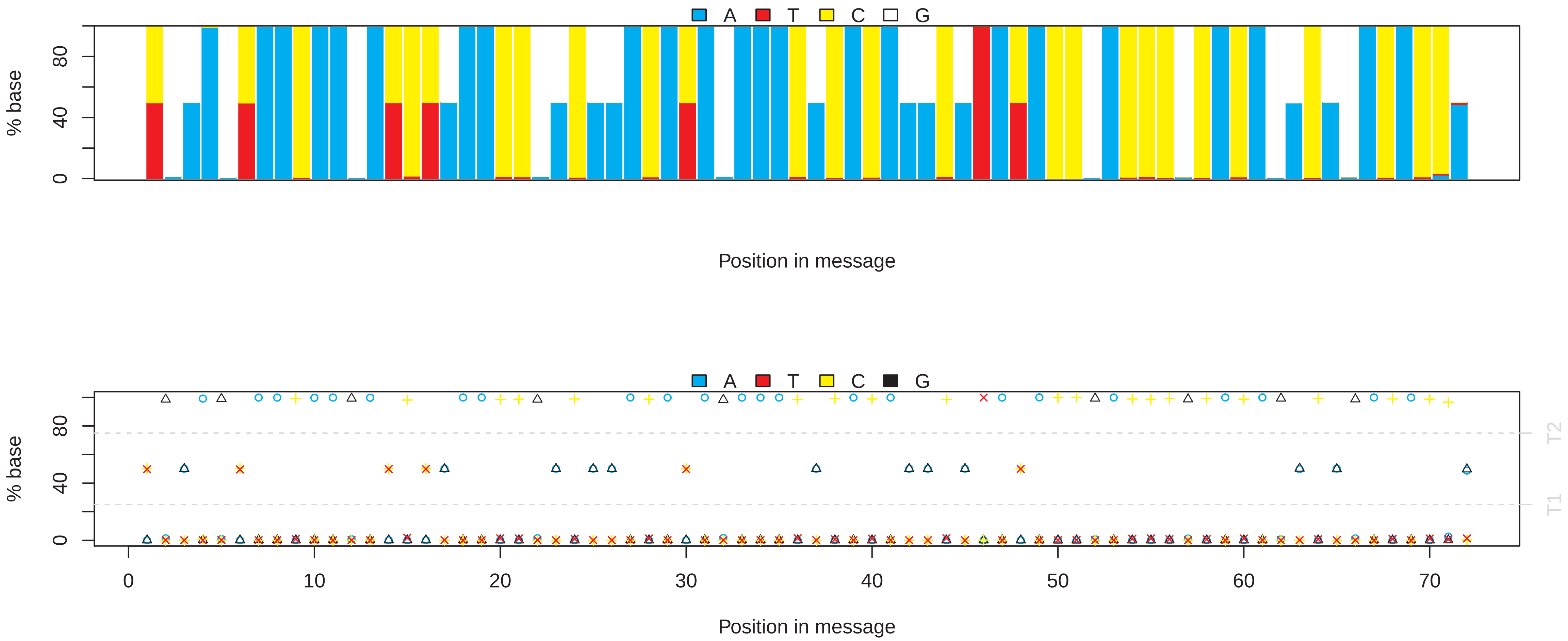

100011000001011100011000000101000001000000010101000101100001010000010110

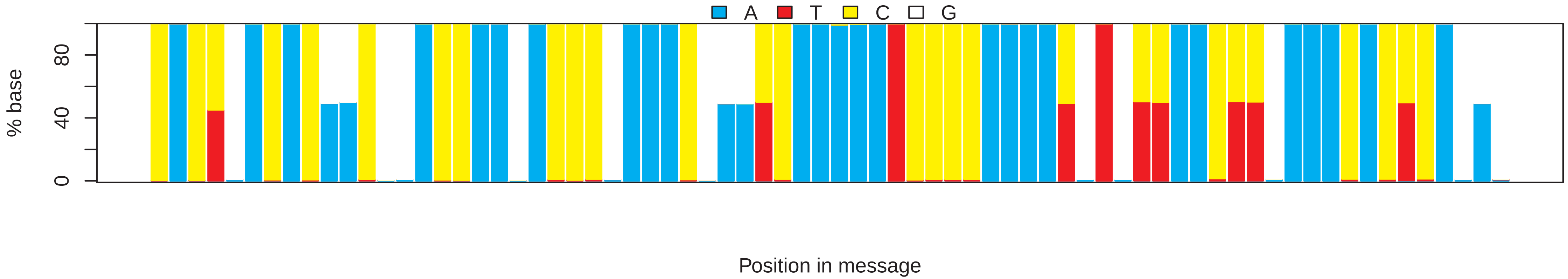

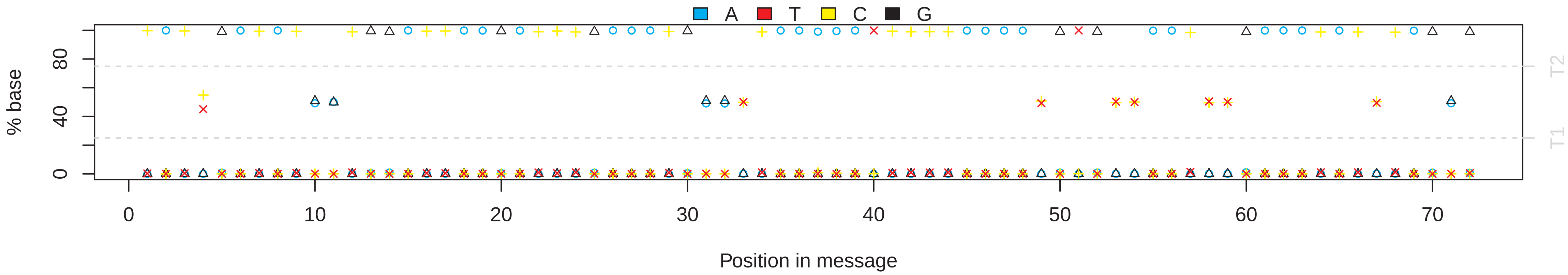

0001000000011000000101010000001001100000101110000111011001110000101110000

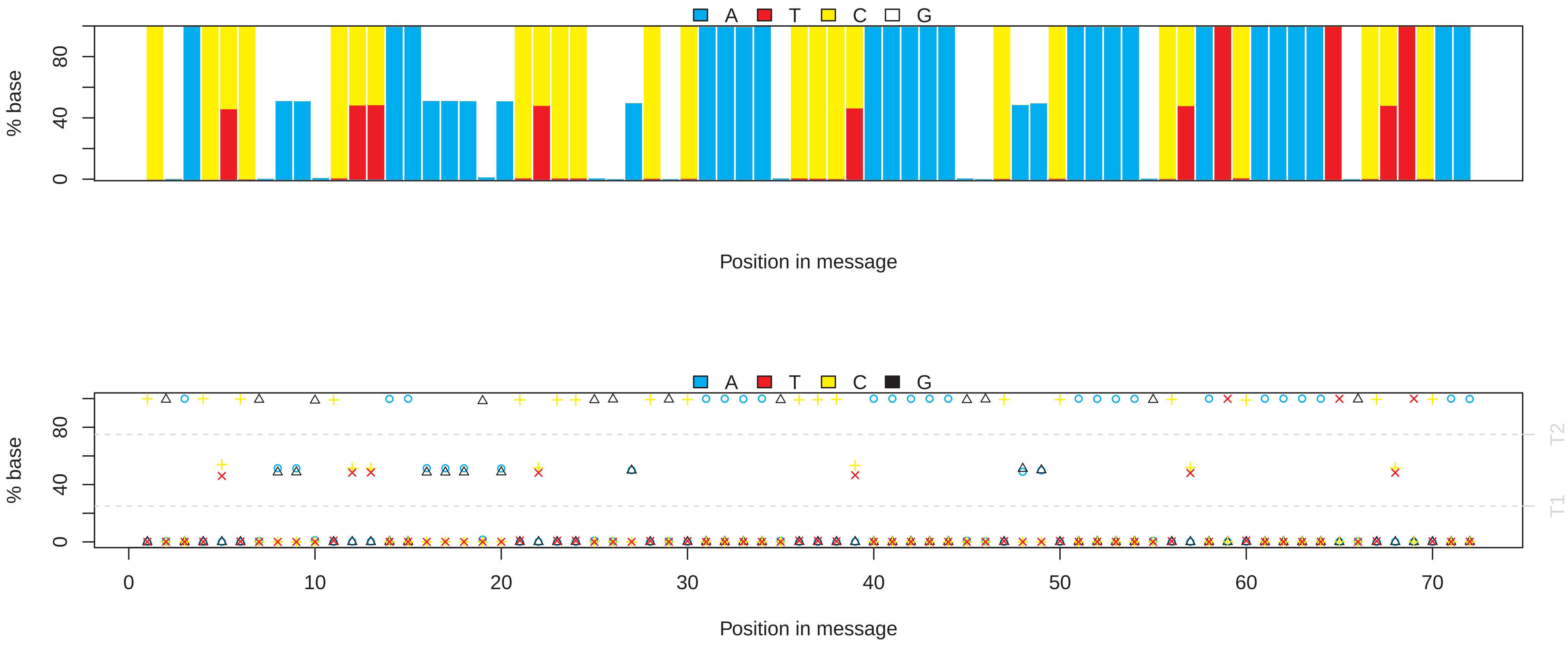

0100101000011000000011110100100000011010000000100010000011011000011111000

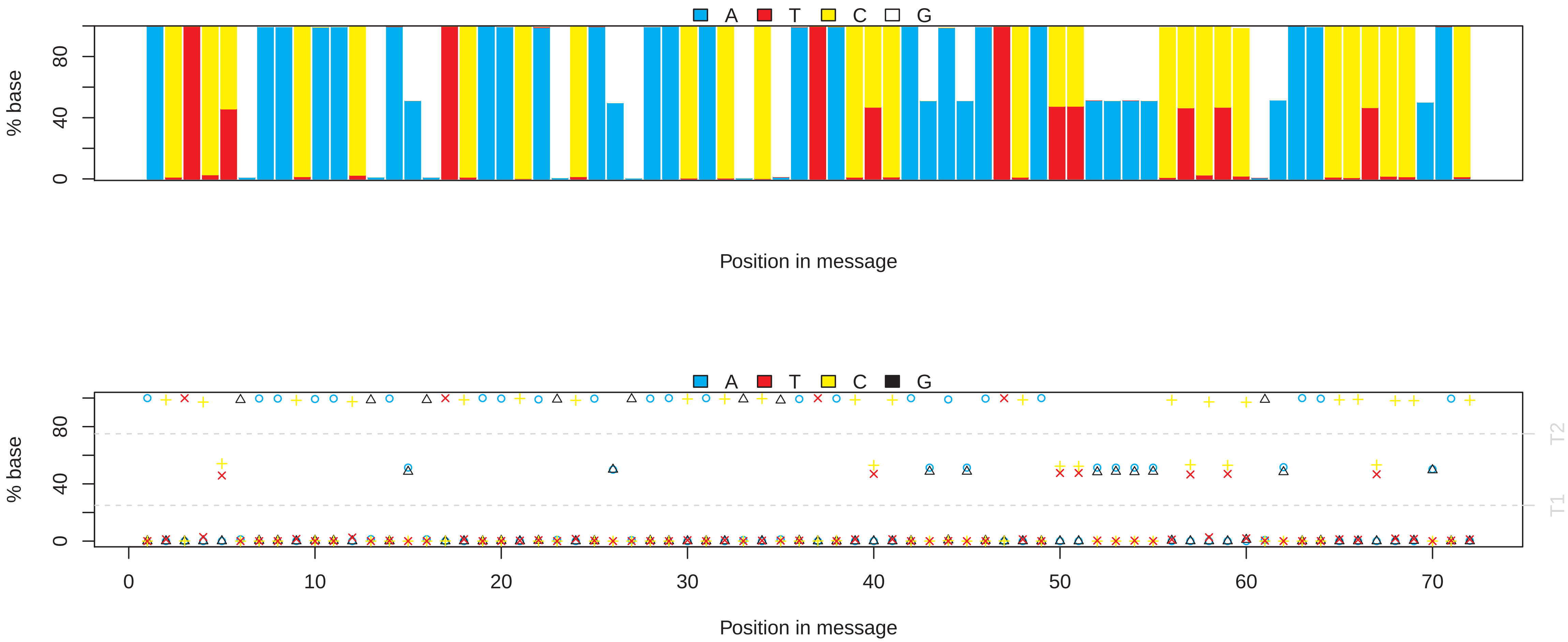

011110001001000011001000001001000000101110000010011000001010100000101001

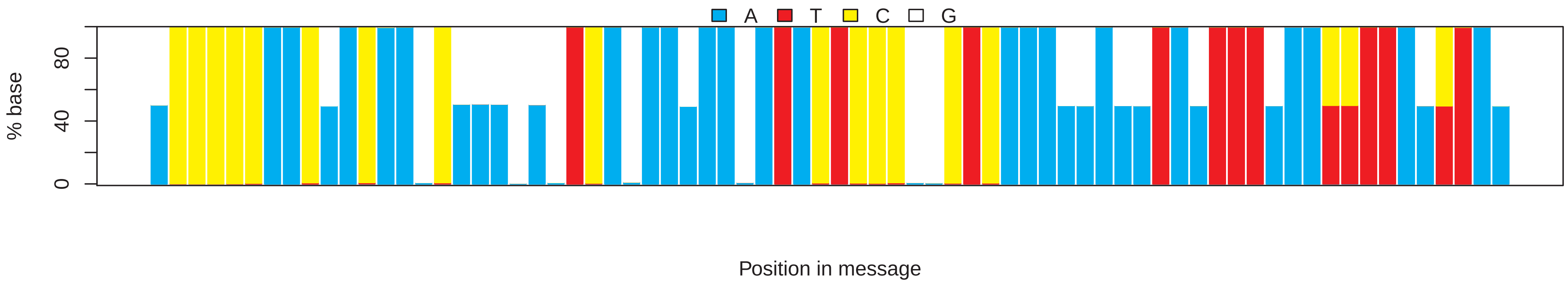

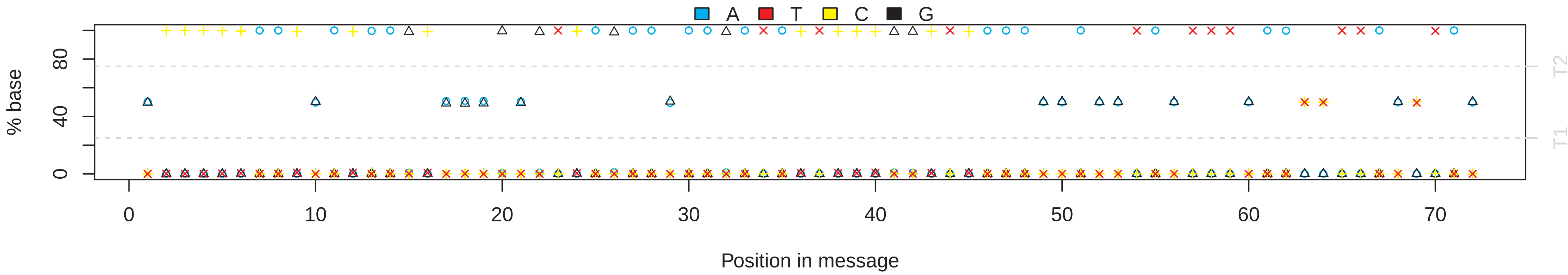

000010001000001100010011000000000101111100111000000001001110001111001100

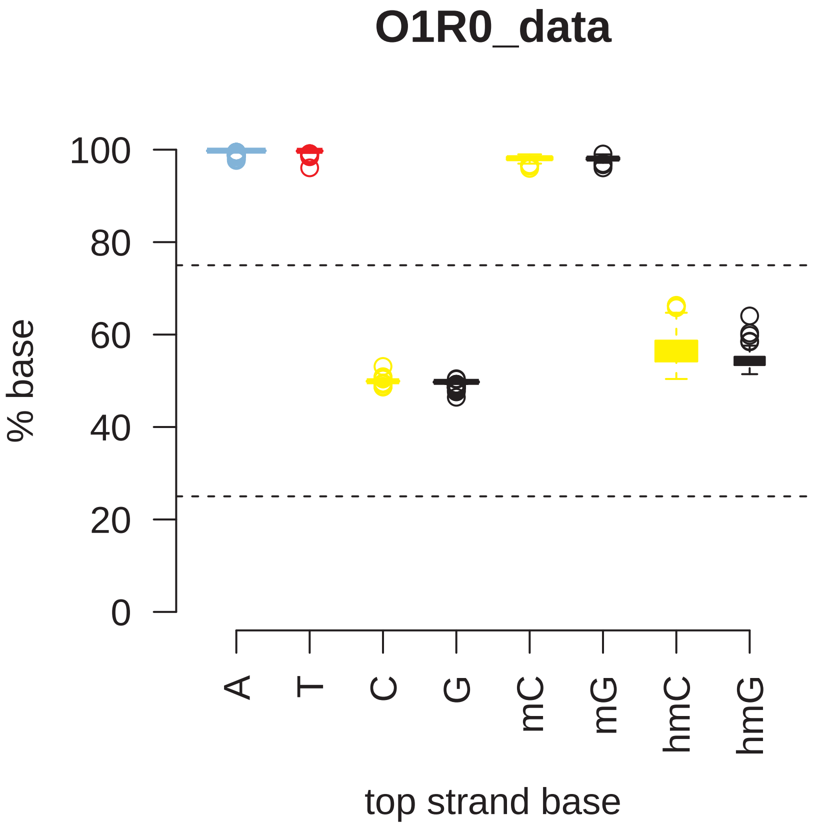
O1R0_data
% base
100
80
60
40
20
0
A
T
C
G
mC
mG
hmC
hmG
top strand base

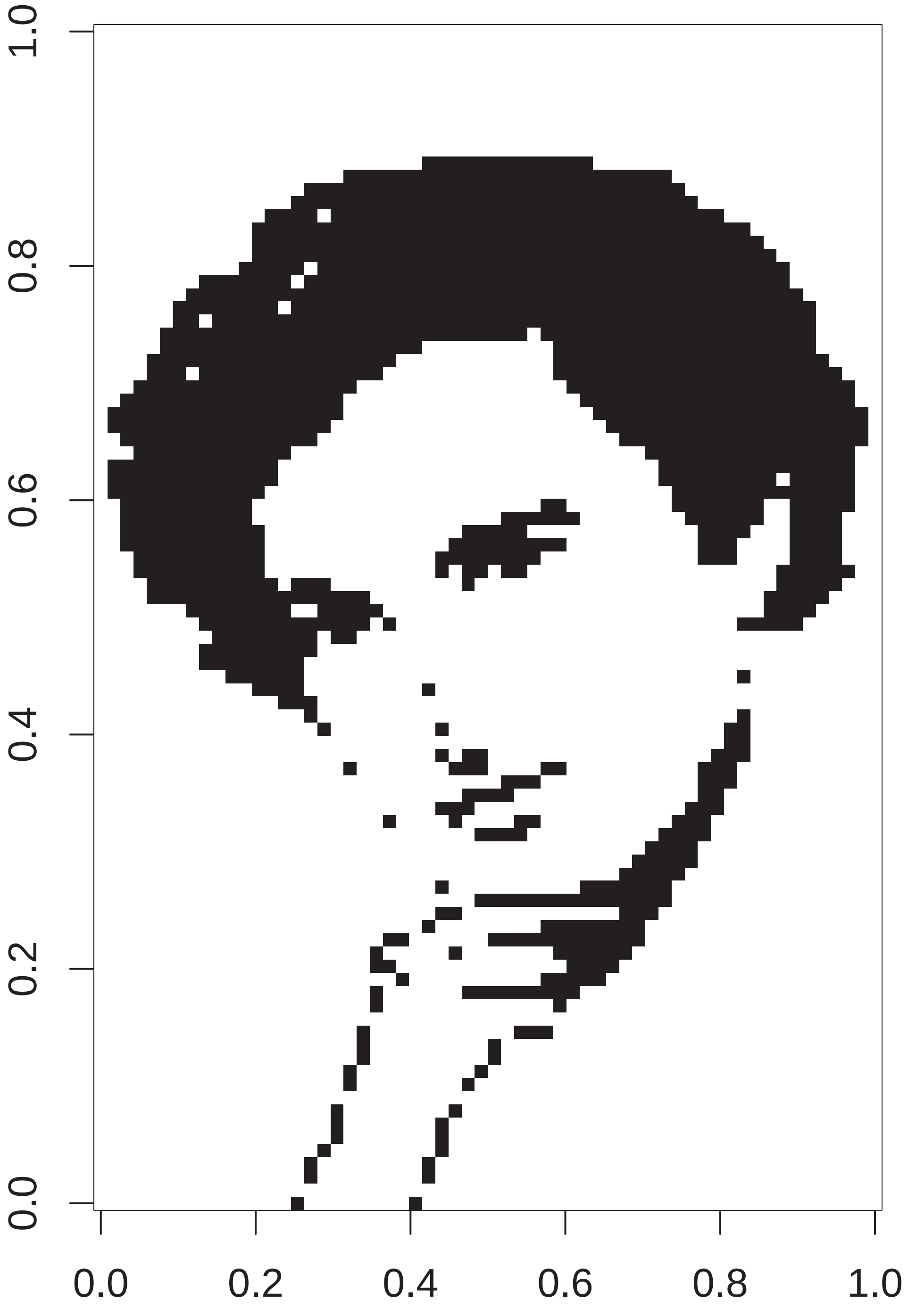

1.0
0.8
0.6
0.4
0.2
0.0
0.0
0.2
0.4
0.6
0.8
1.0

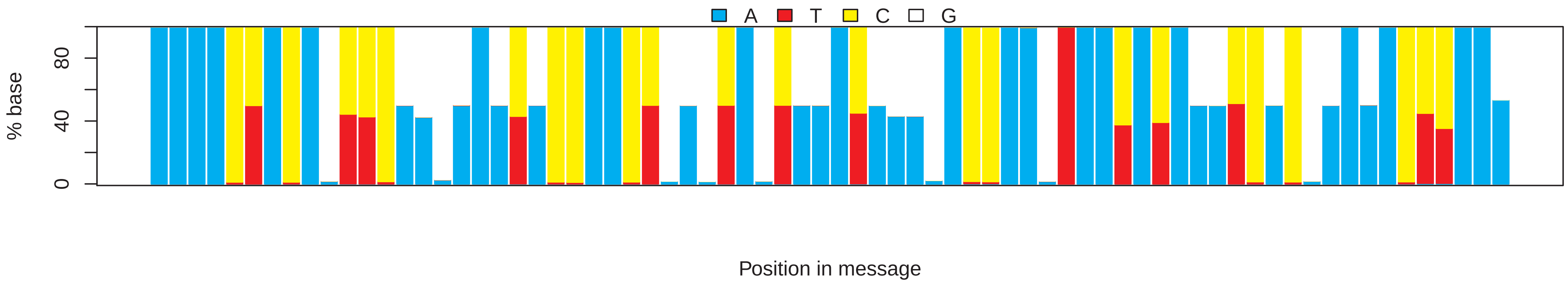

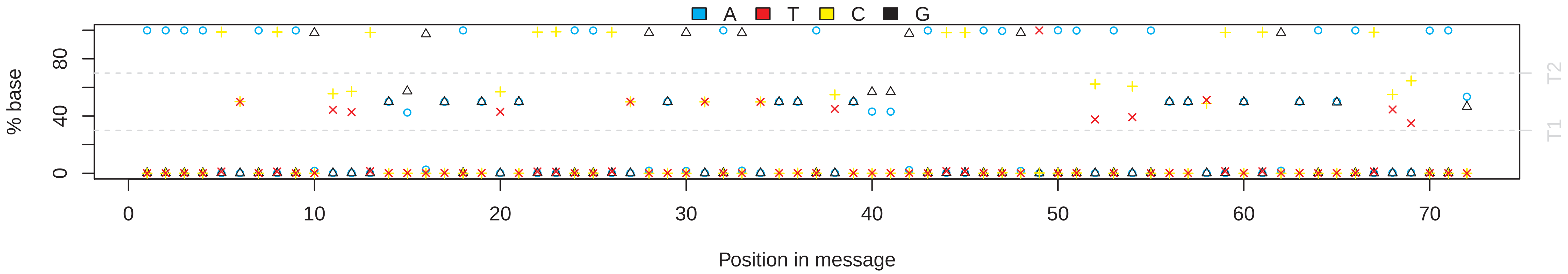

000001000111000100010000001101101100010001000001100101000100010000011000

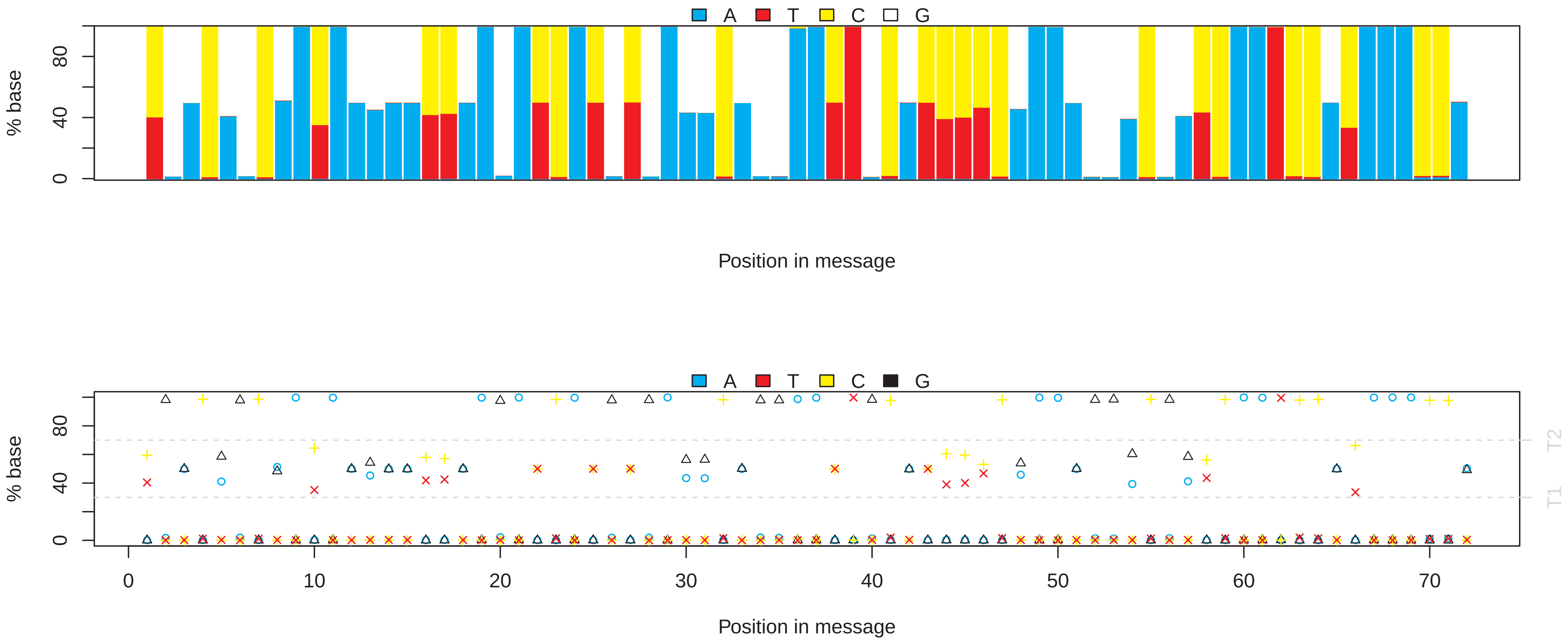

110001000100000110010100111100000110011100111100000110010100010001000000

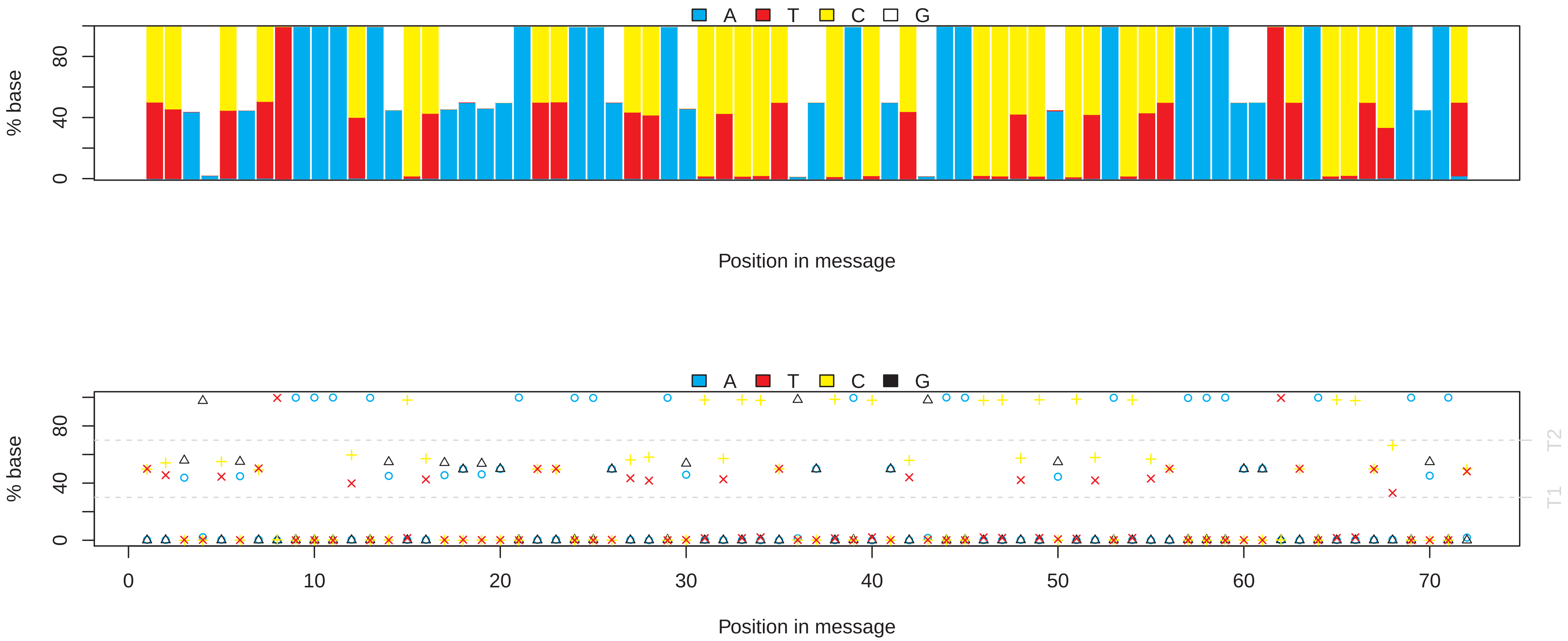

110110110001000100000110001100010011000001100001000100110000011000110001

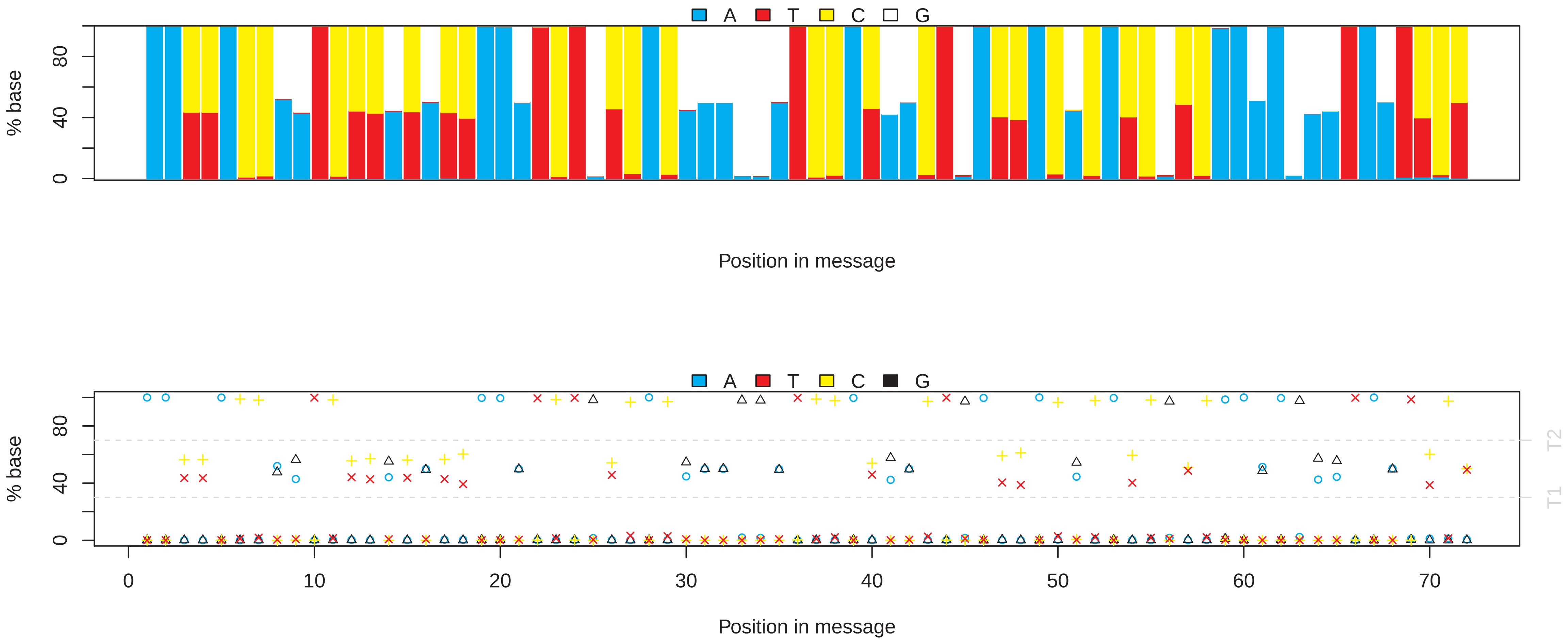

0011000001011010110001011100000011010001000110110000010110000001001001101

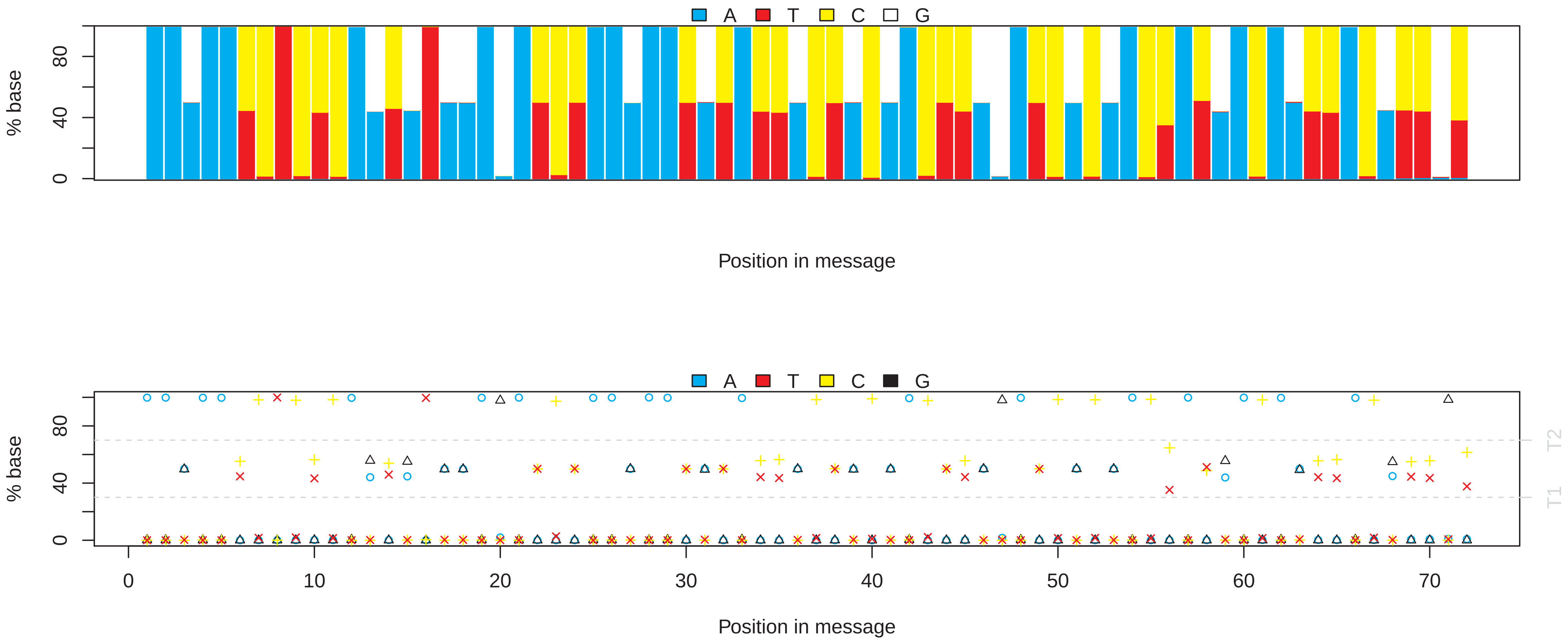

0000010101000101000101010000010101100100000110101000000010100000110001111

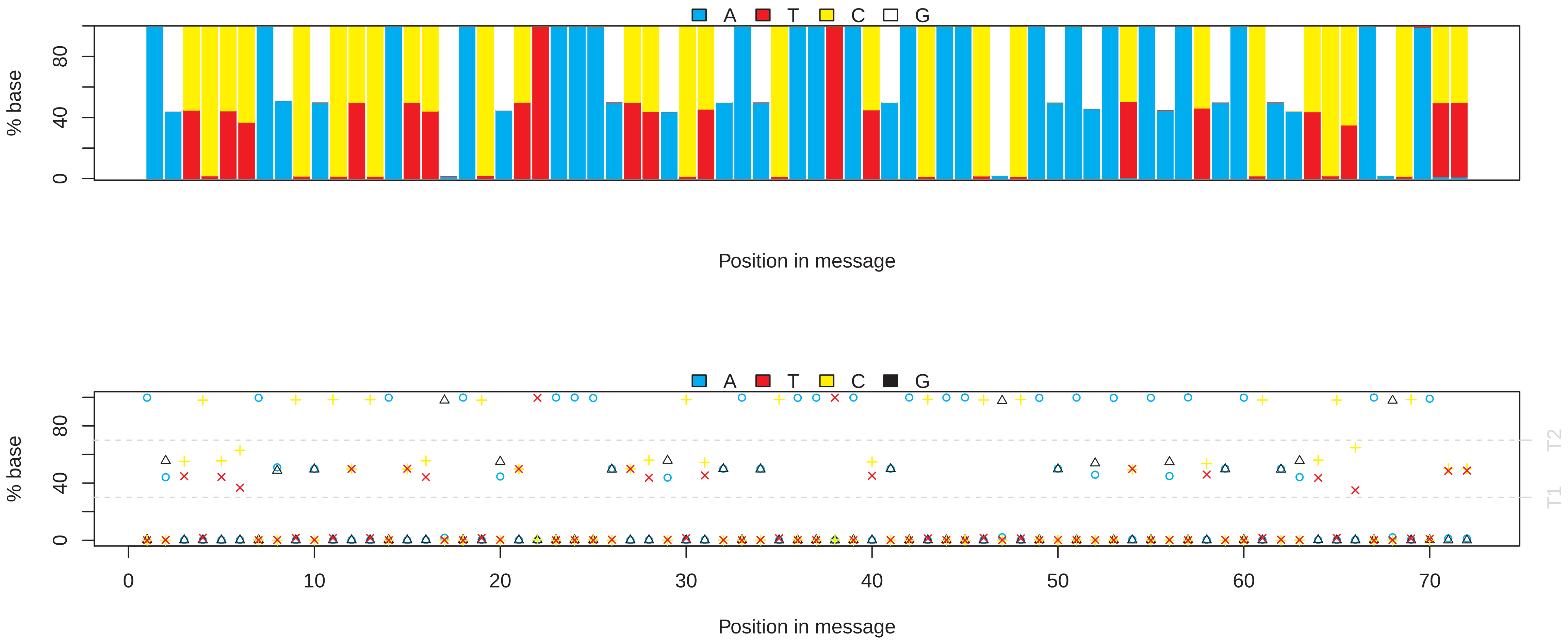

0010110000010011100011000011001000000101000000010000001000100000101010011

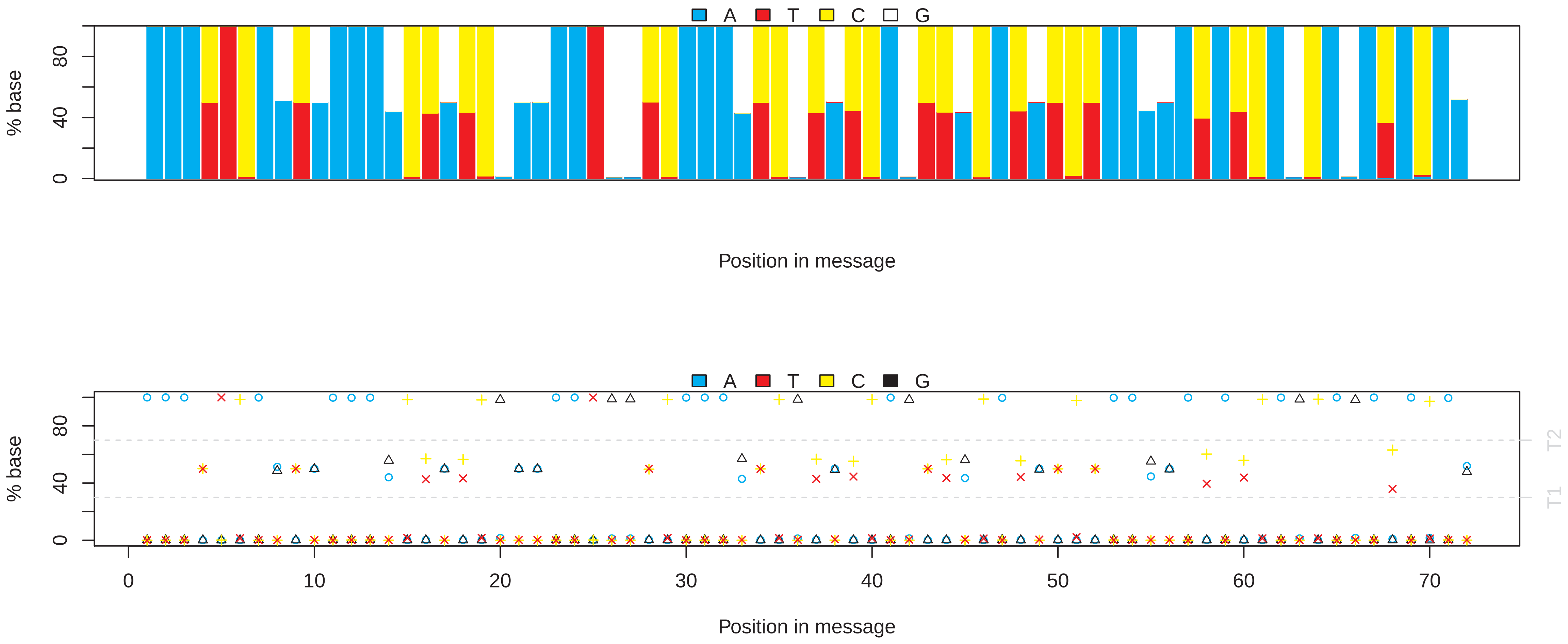

000110001000000101010000111100000101101001110001010100000101001001010000

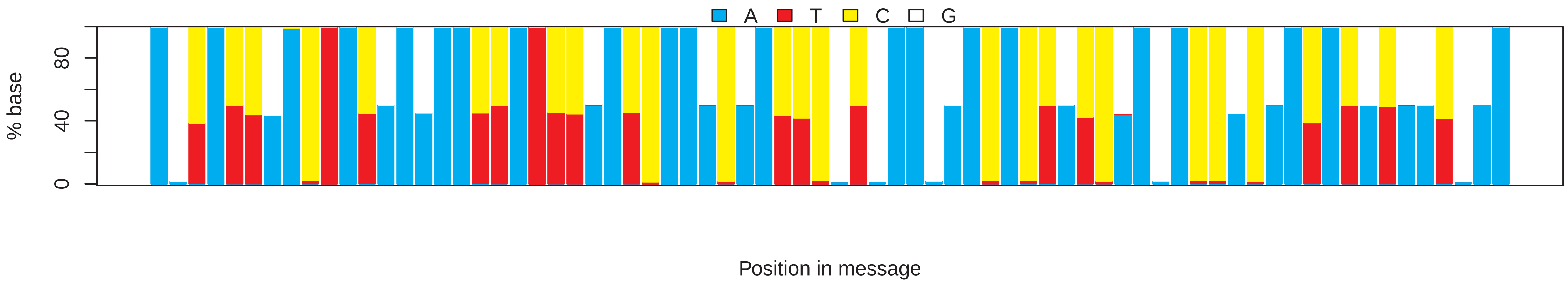

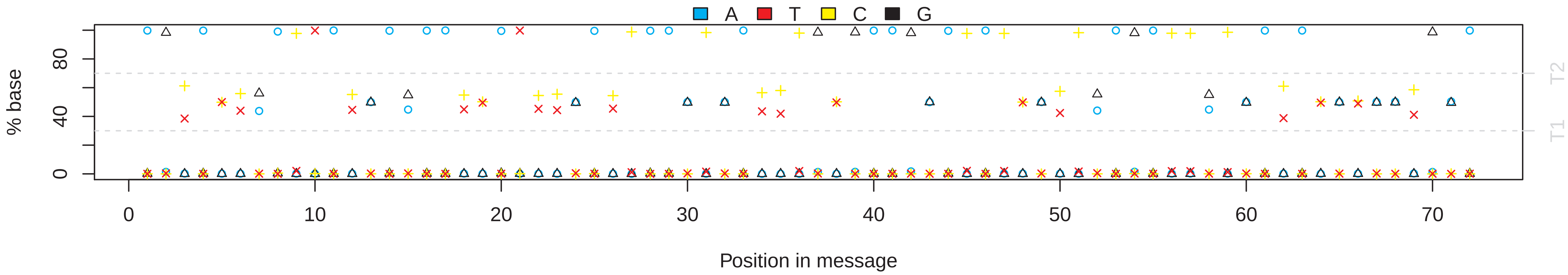

0110110001010000011011100100000001101110010000010100010000000010101001100

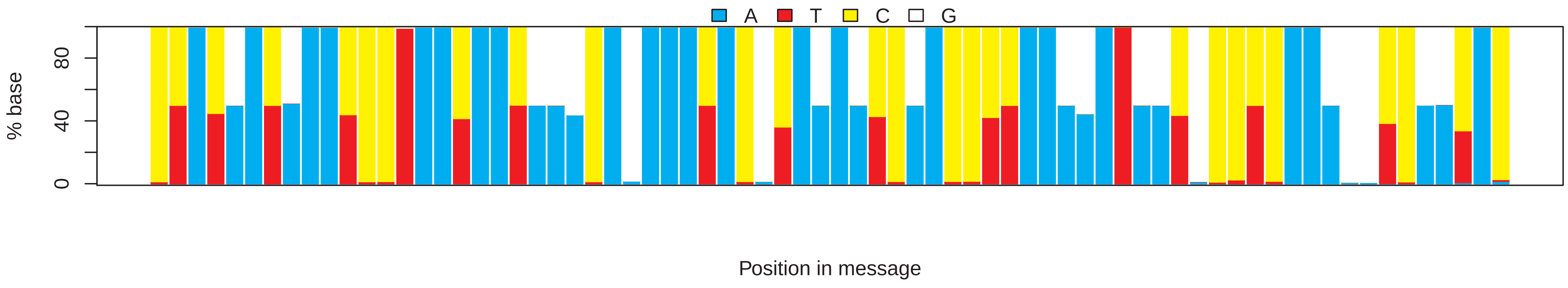

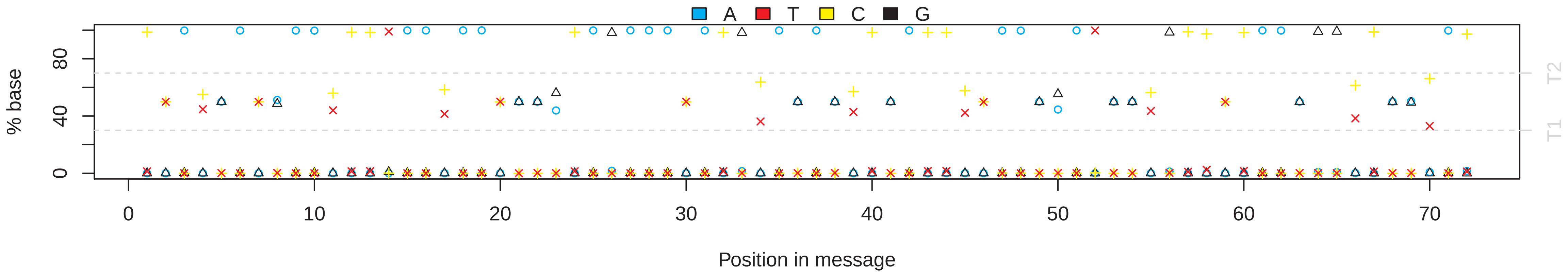

010100100010010010010000010001001100001000001100000100110010000111000100

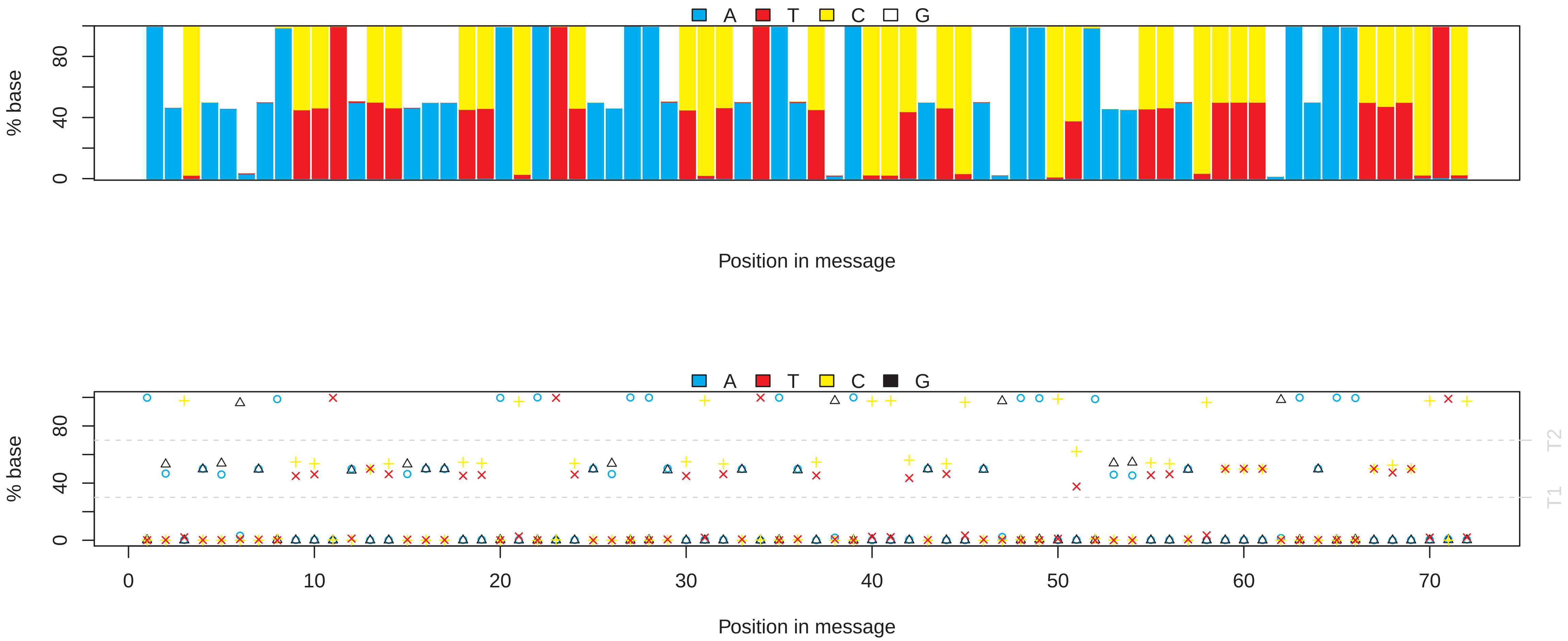

000001001110110001100011000001010100110001010010001000110011110000111010

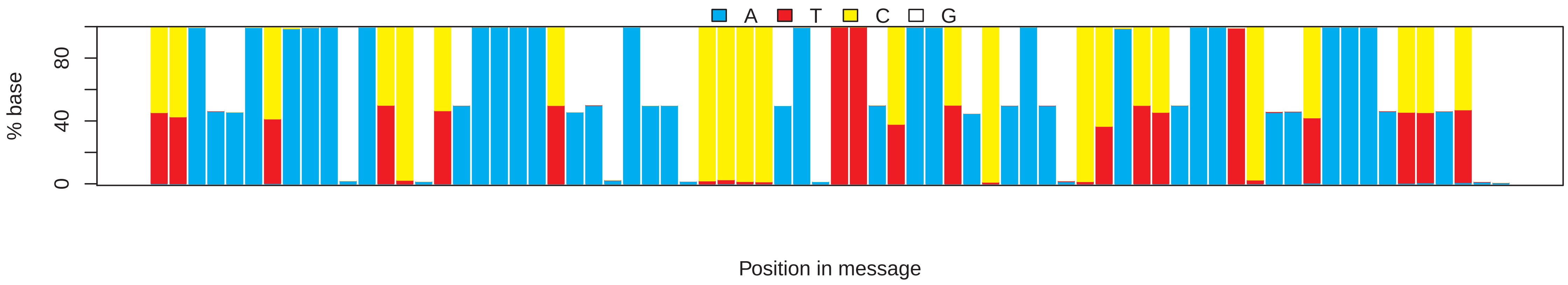

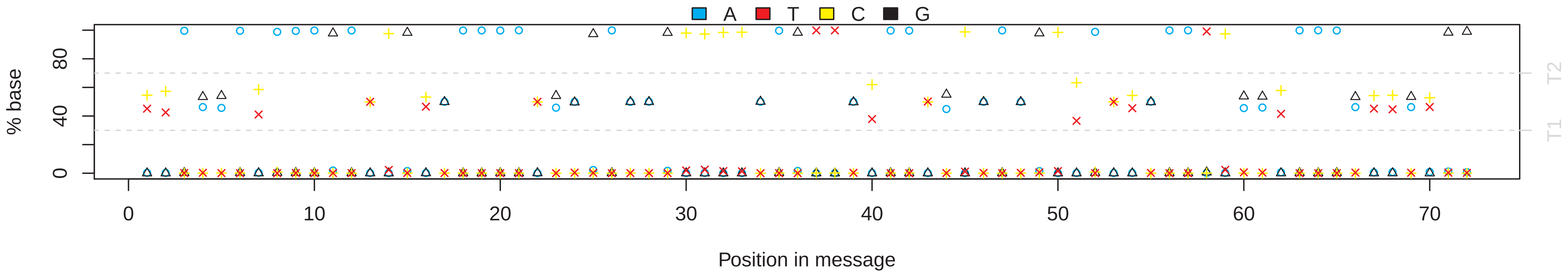

110000100010101100000100100010000001110100100000101011000100010000110111

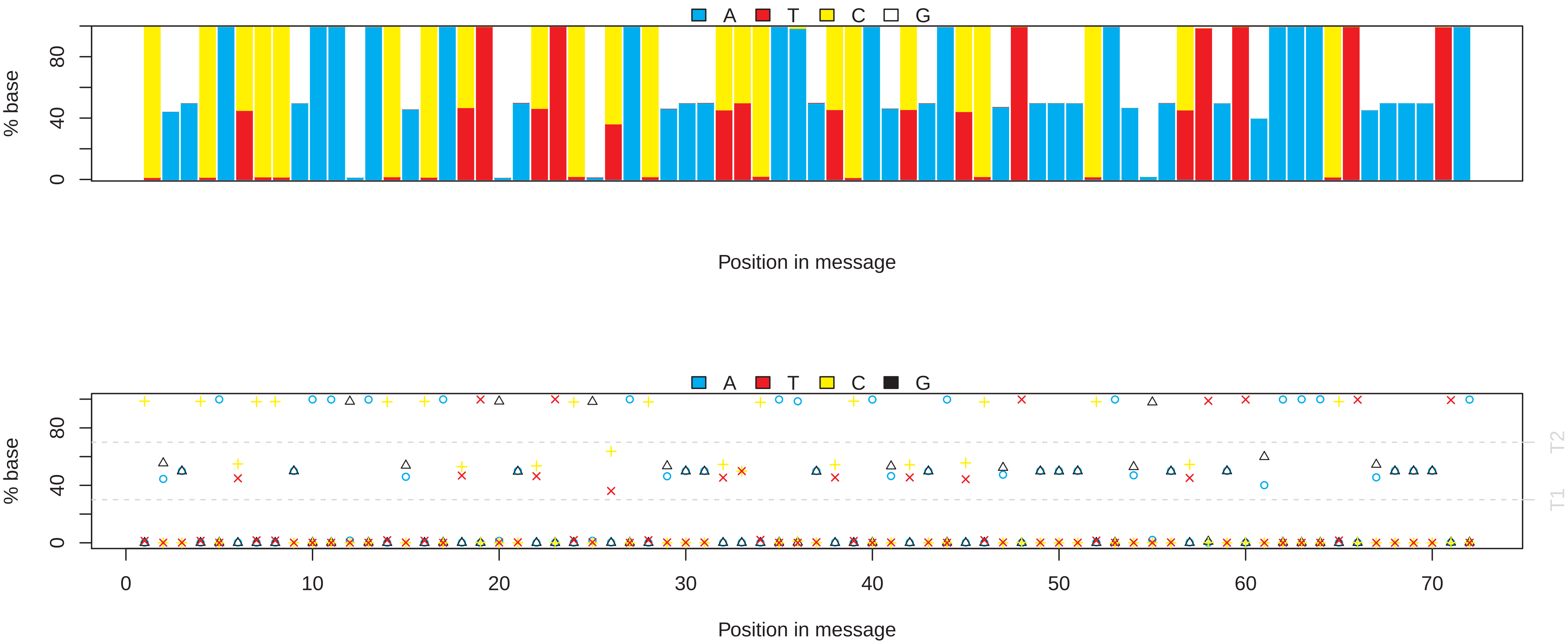

000001000001000001110110110000011000010001001001000000101101000001000010

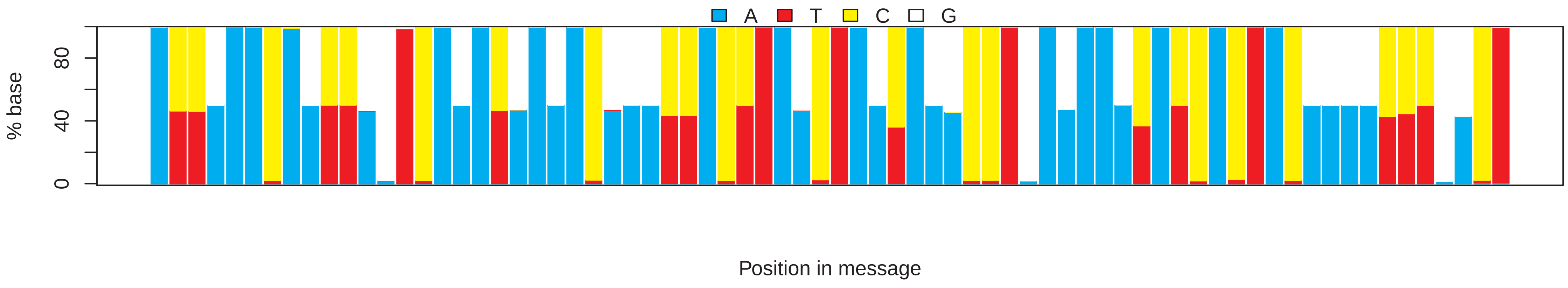

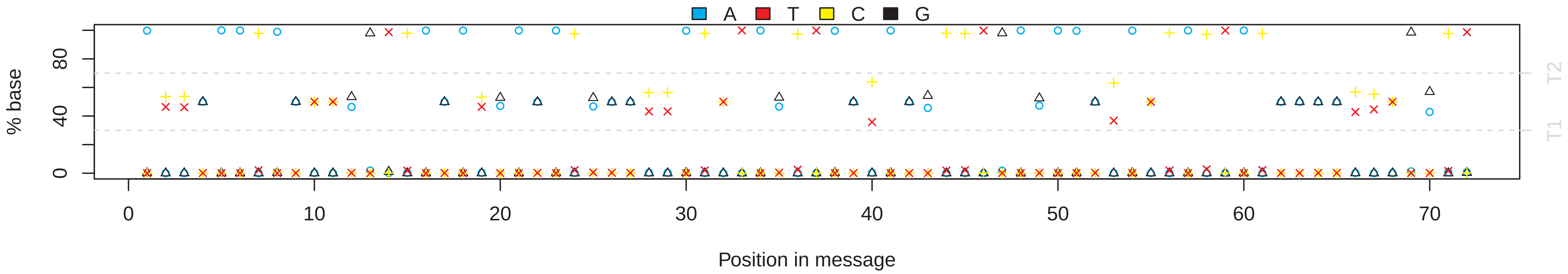

011000000110110000100000001100110001001000000110000010100010000001111001

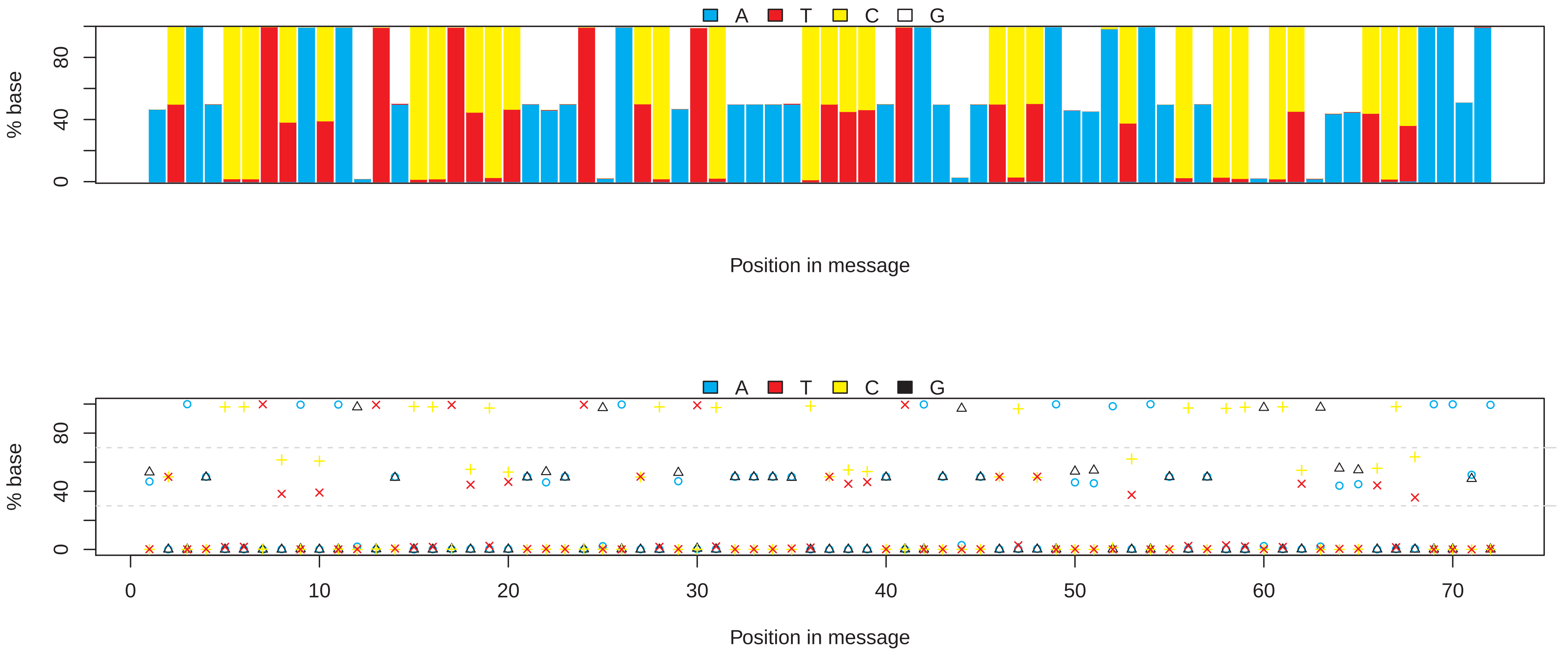

010000110101100011010001101001000000111010010101000010000001011001010000

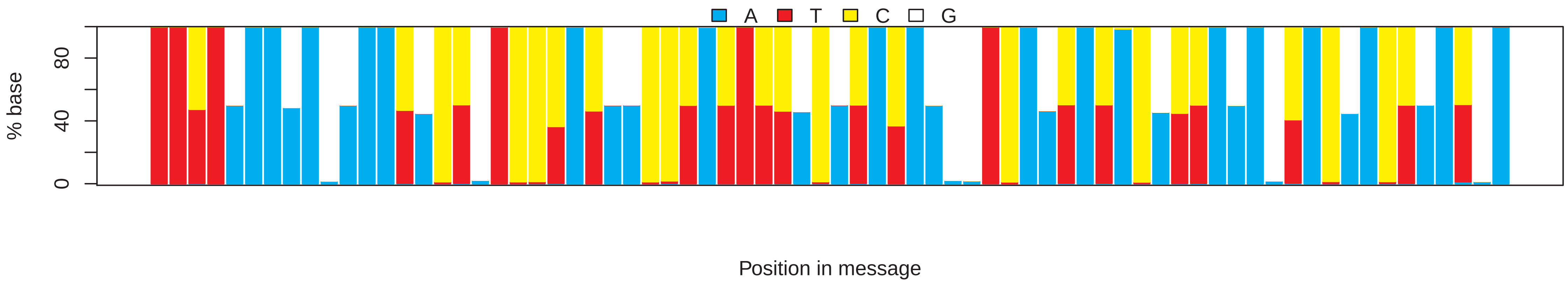

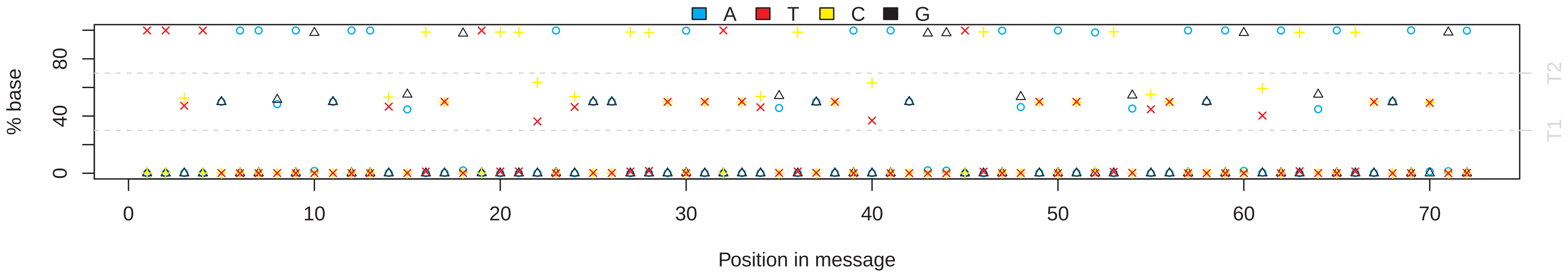

1111000001000100111001010000101111000101001110001010001100011000000100110

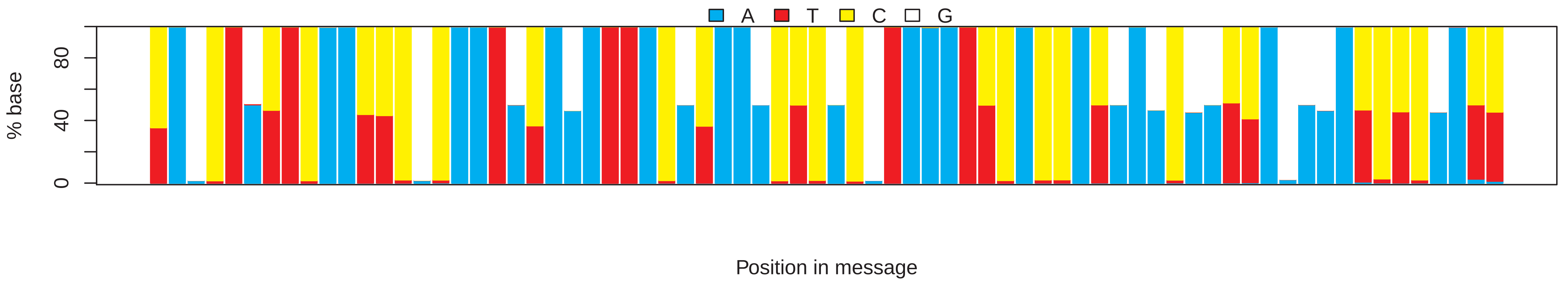

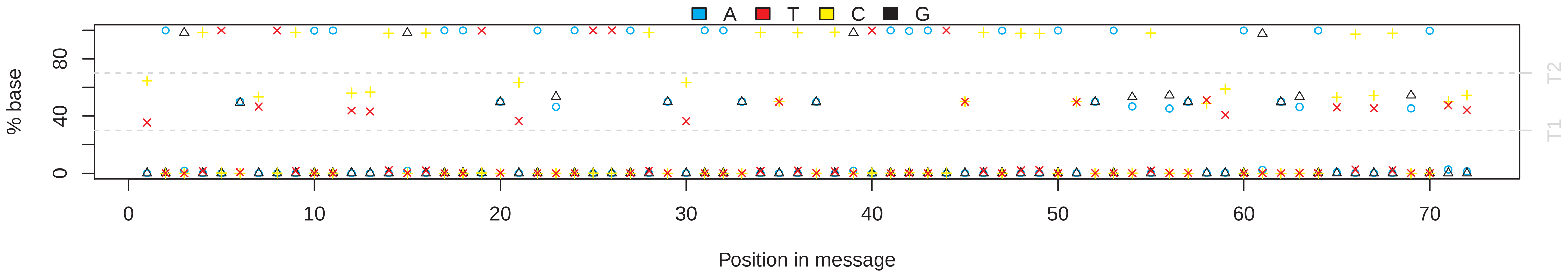

101010110001101000101000110001000010001100011000001000000110100010100011

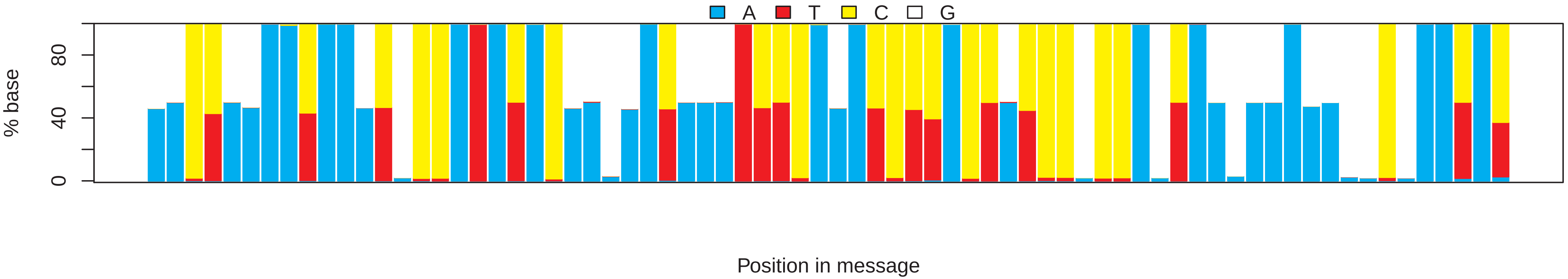

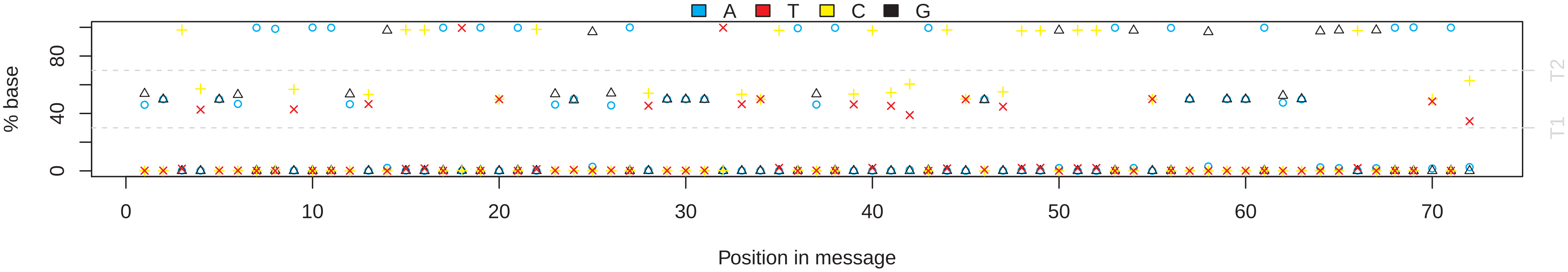

000100001000110001010000100100011100001011001010010001100100000110100101

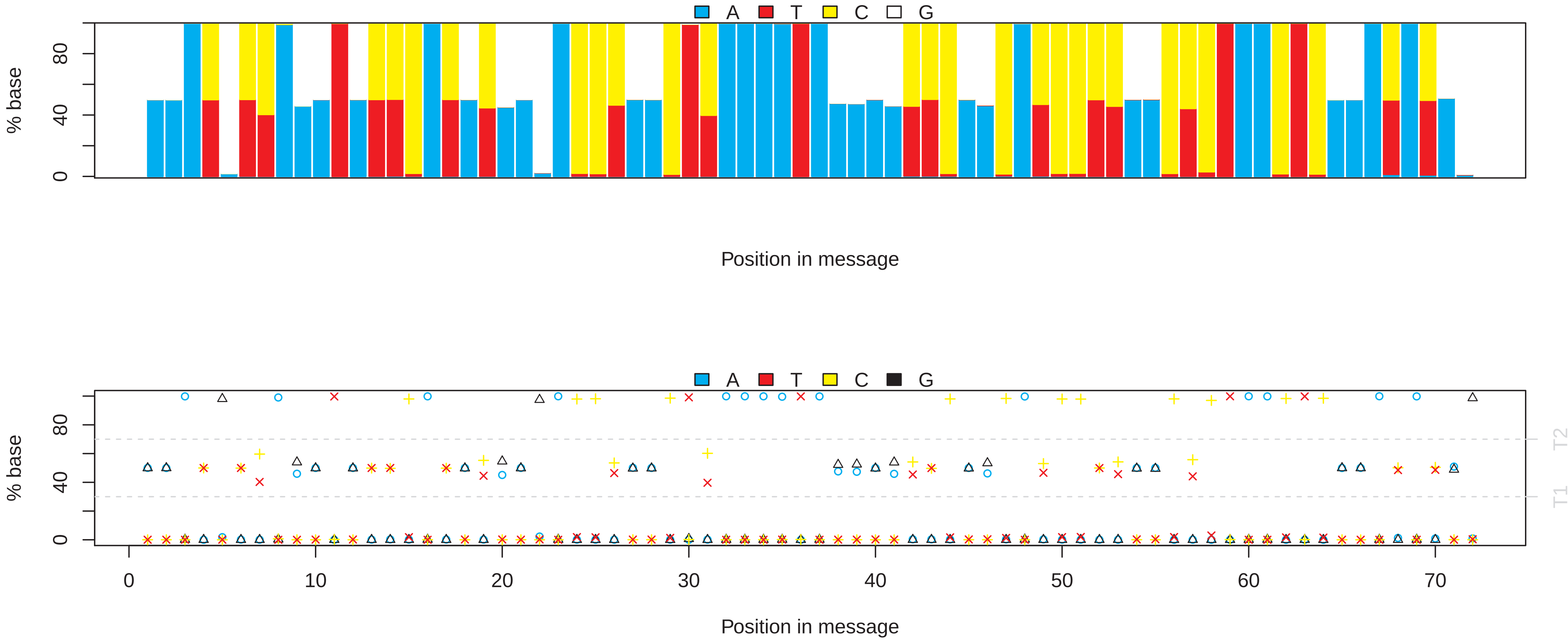

000111100010110010100100010001100001000001100000100110001010001000010101

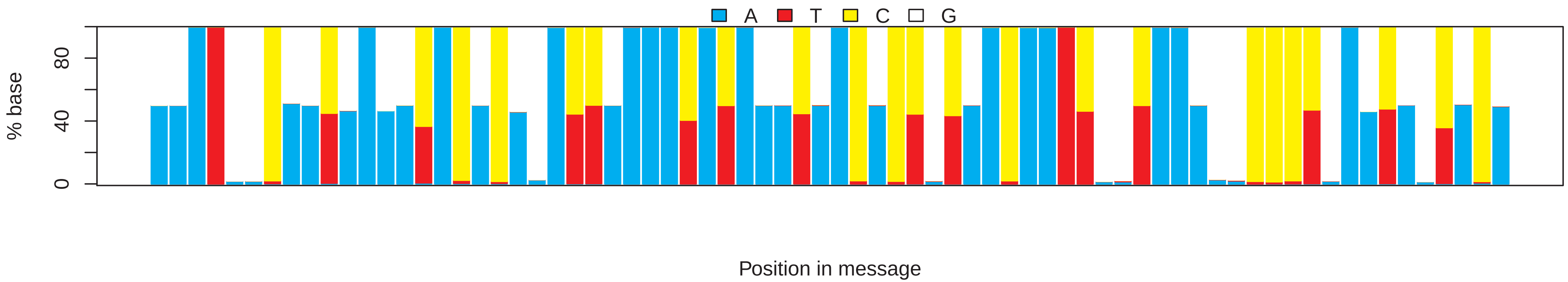

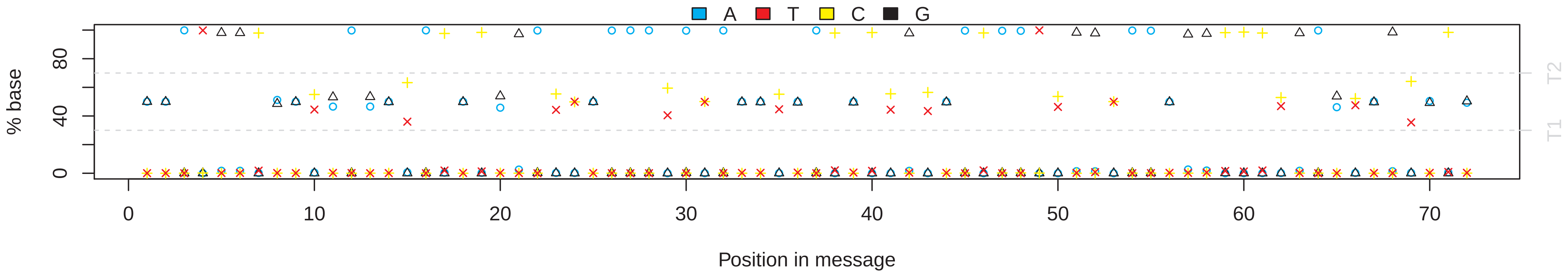

000111000100001000001011000010100010000011100000111110001100011001011000

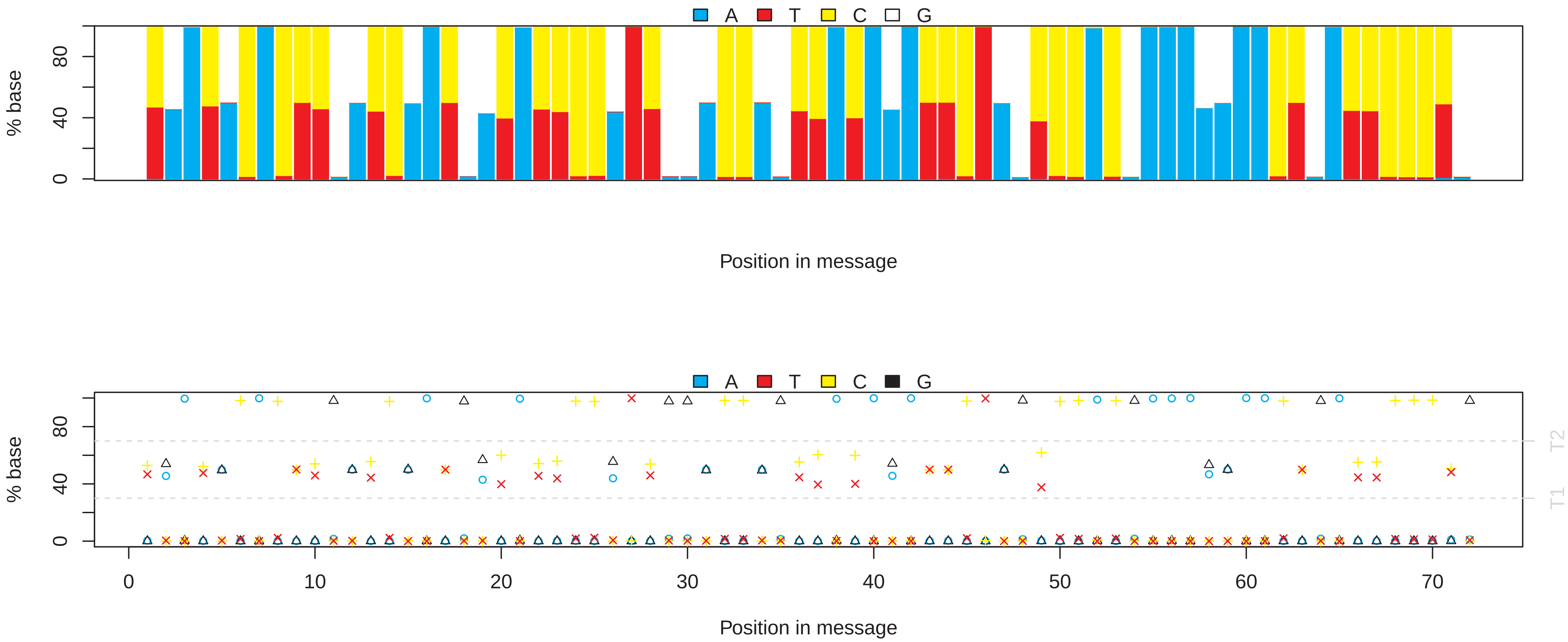

100100001110100011010110001111000011101000110101100001000000001101100011

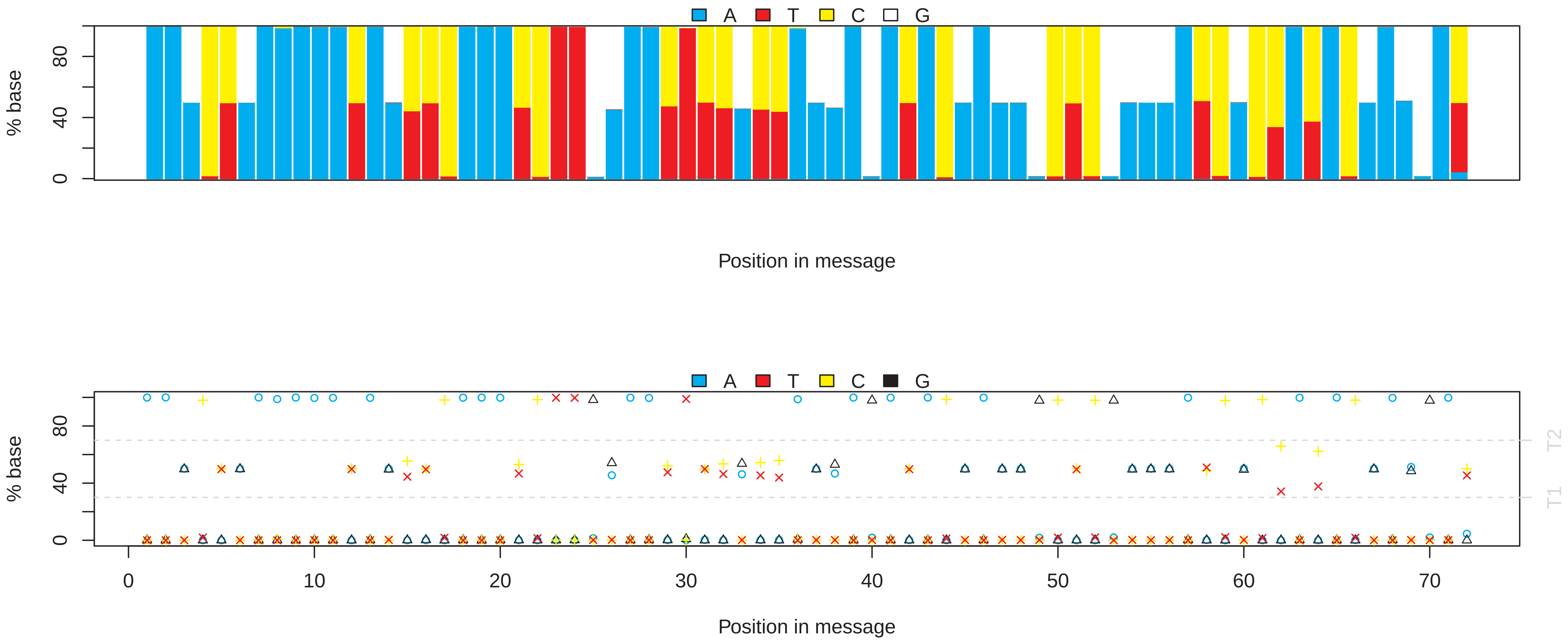

0000100000001001100001011100011110110000101000000101010000100010100000101

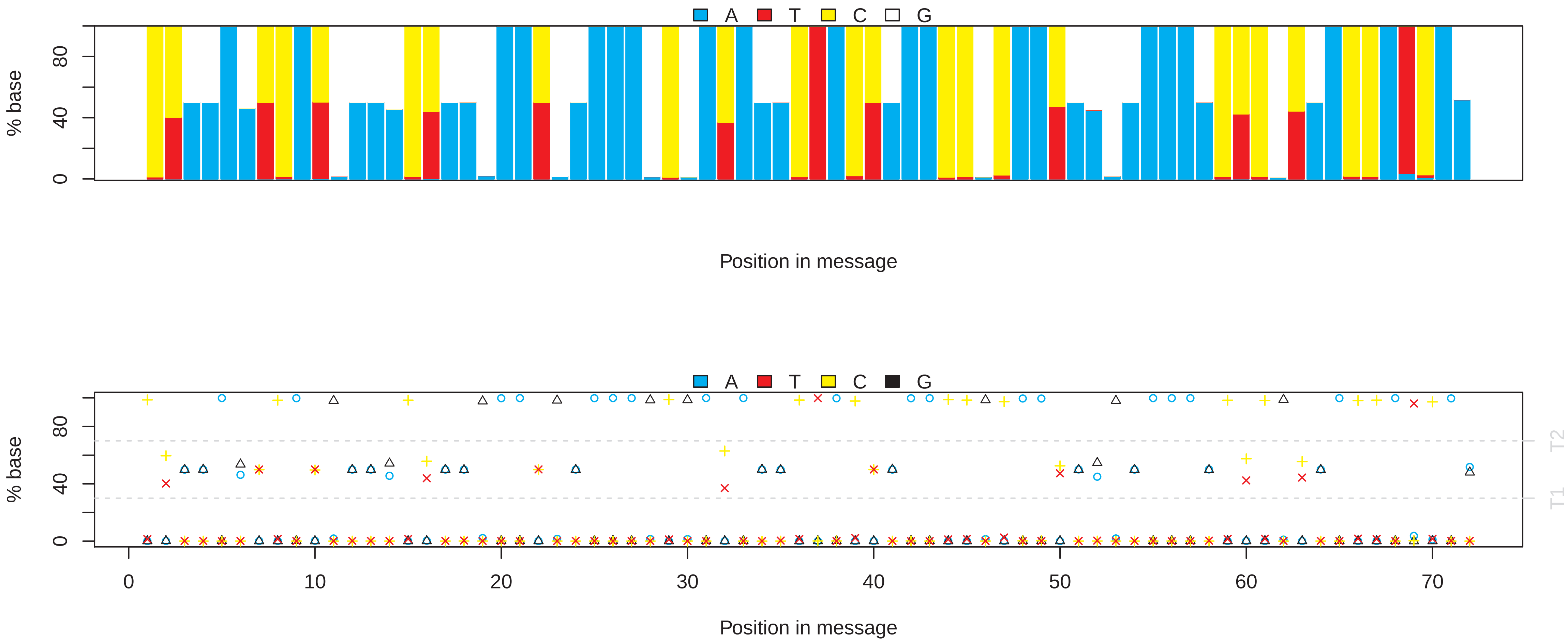

0100001001100001001001100001010100001001000001000100100000010110000001000

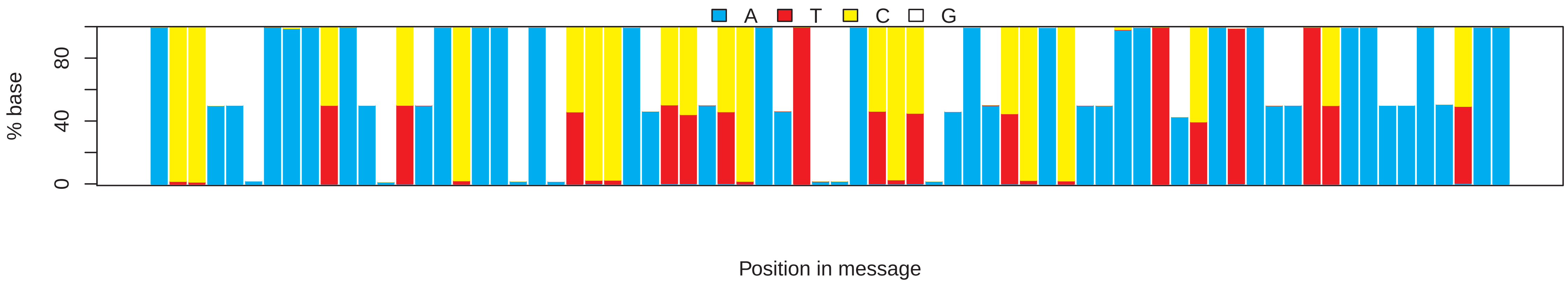

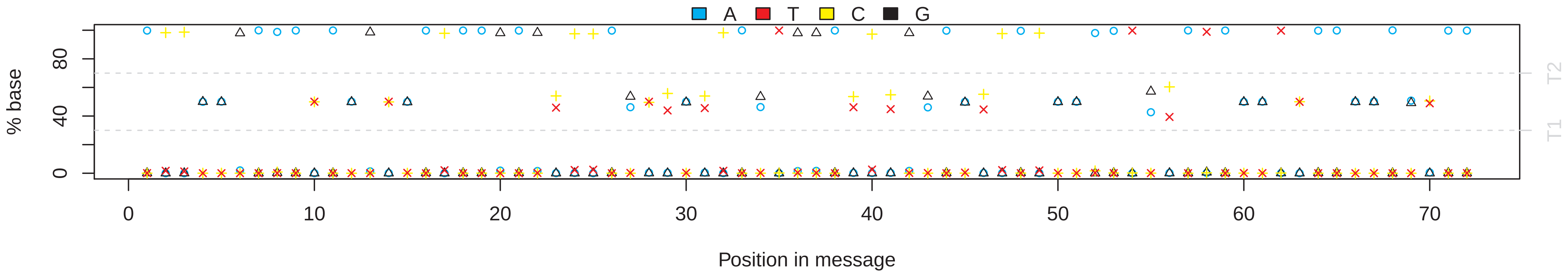

000001000100110000010110000110100011101011000100000001010100011000000100

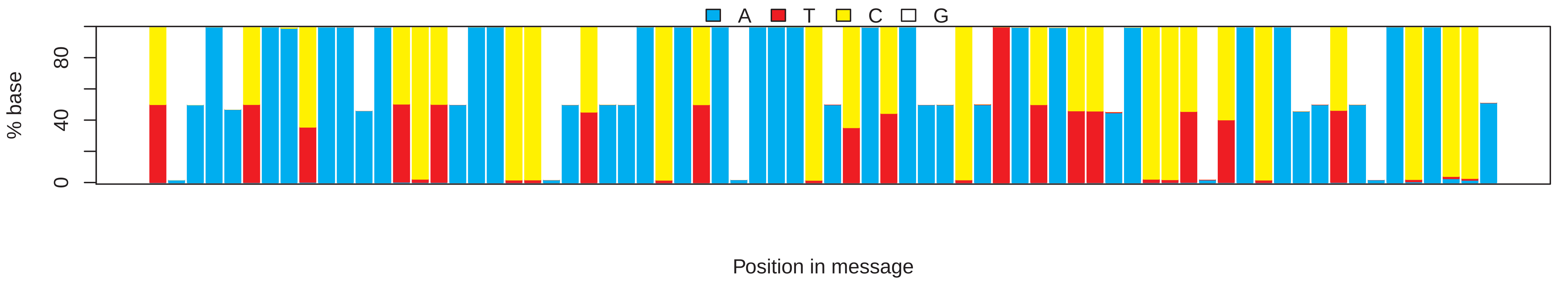

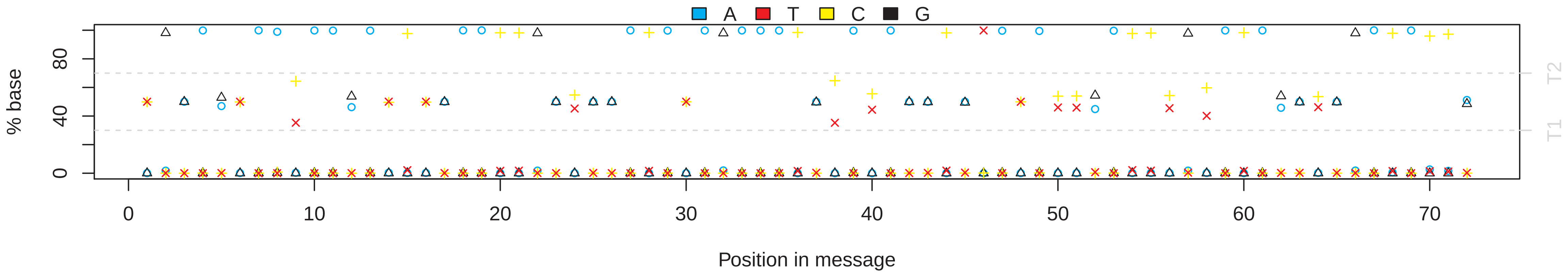

110001001000010100000101000001010000010100000101011000011100000101000000

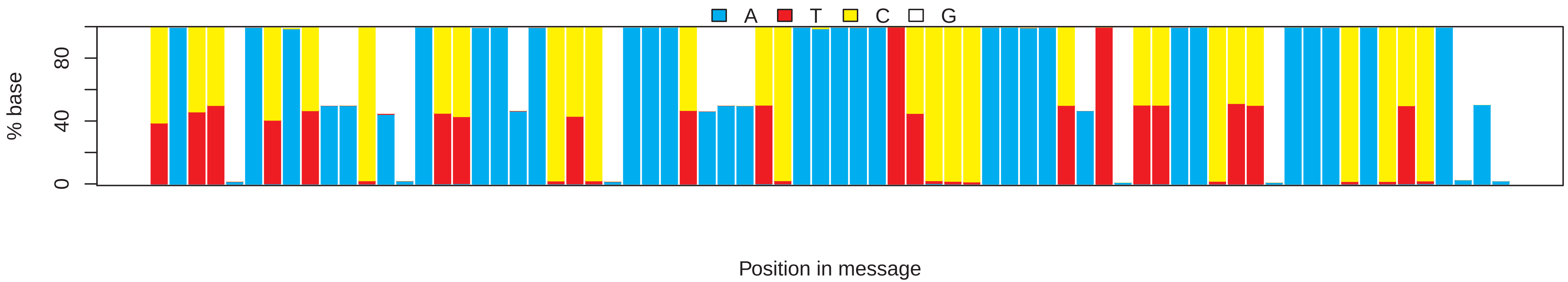

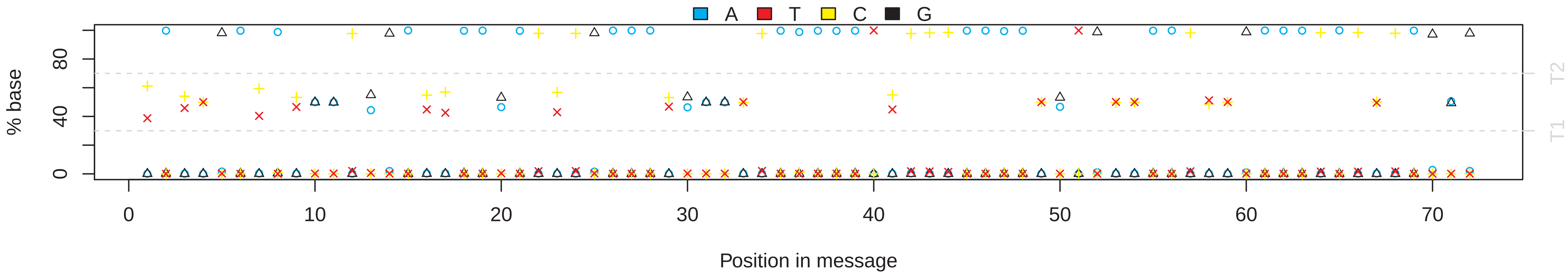

101110101000010110000010100010001000000110000000101111000111000000100101

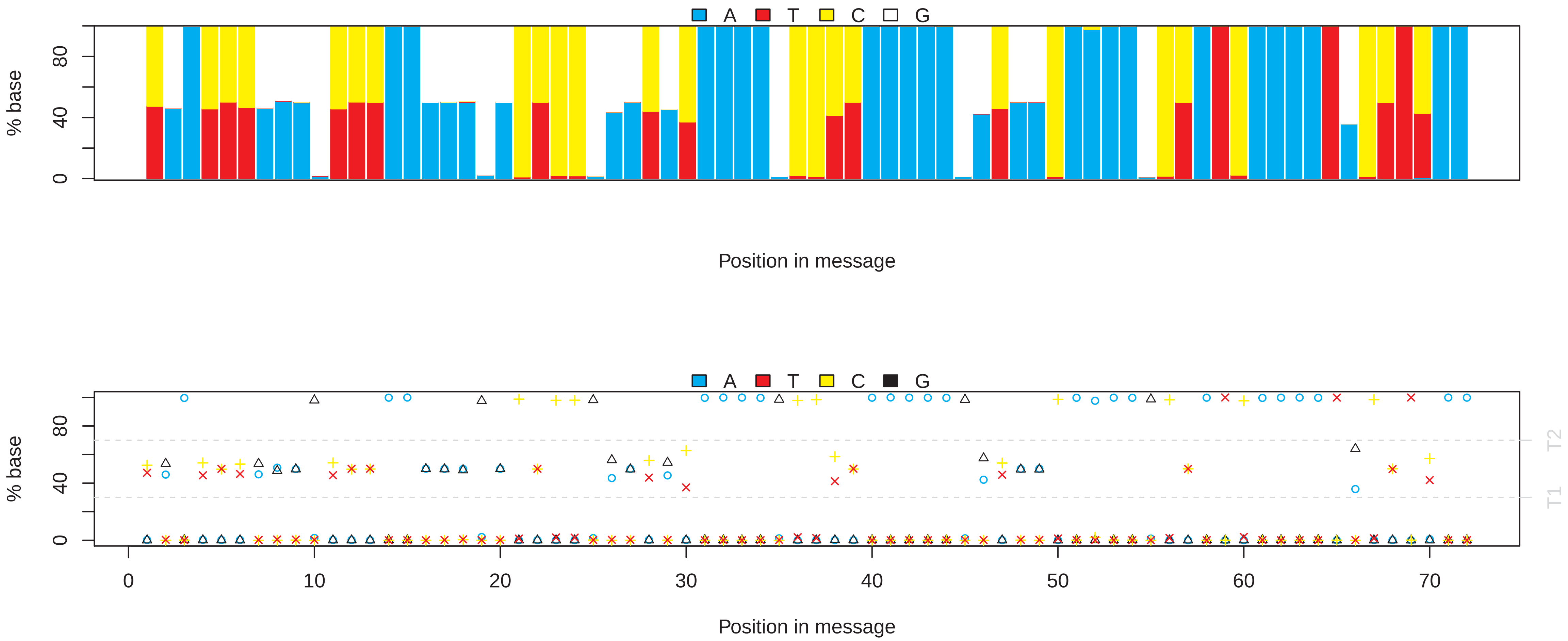

100111000111100000100100100101000010011000001010000000101010000010011100

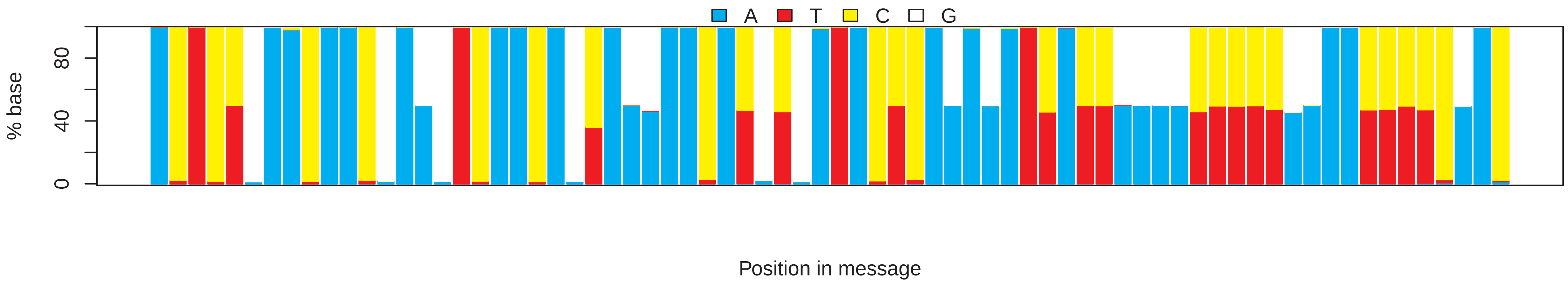

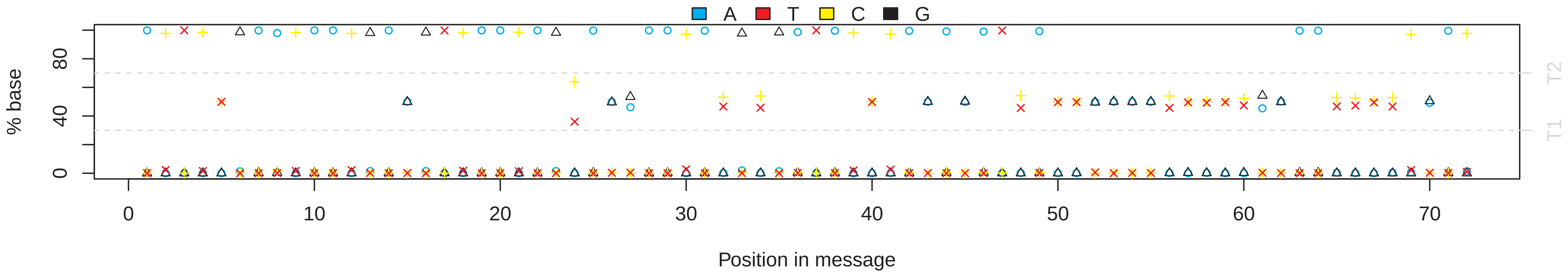

001011000000100110000011000000011110100100000011011000011111000011110000

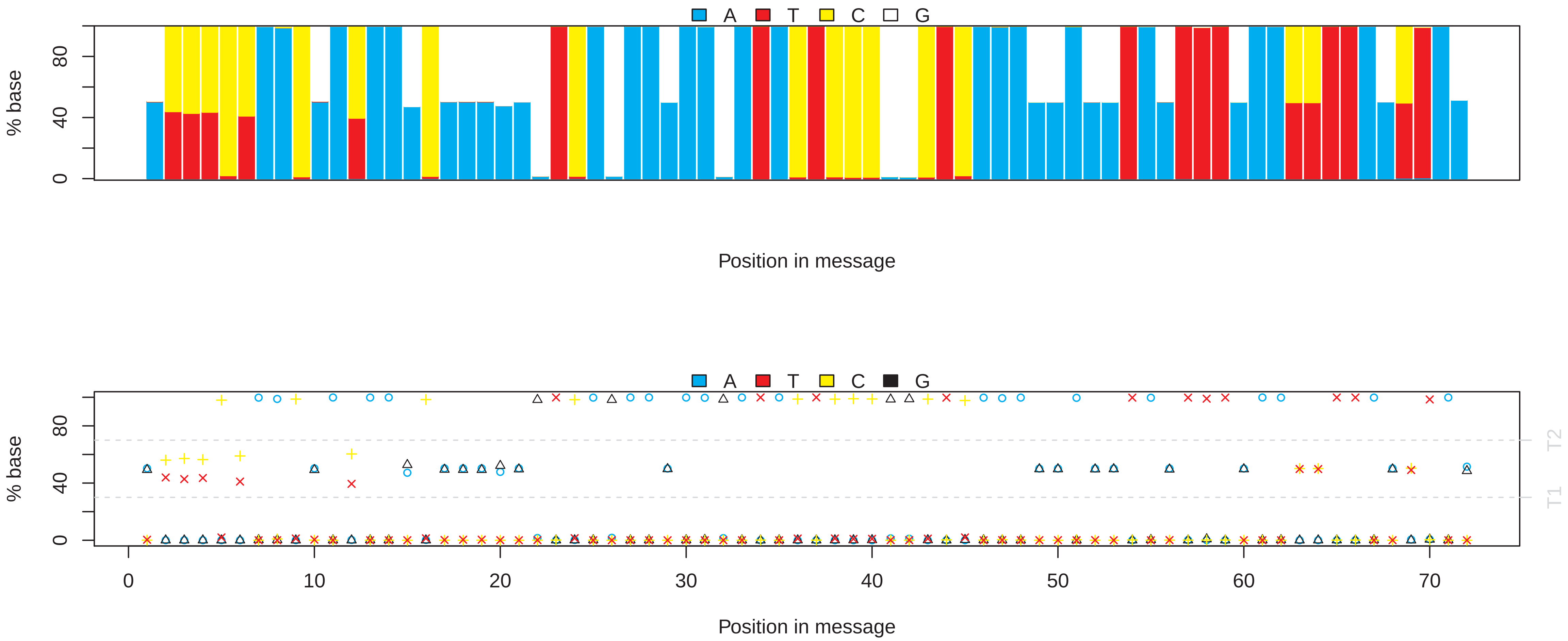

011101000001000000000110010000010100100100011010000000001001110001111001100

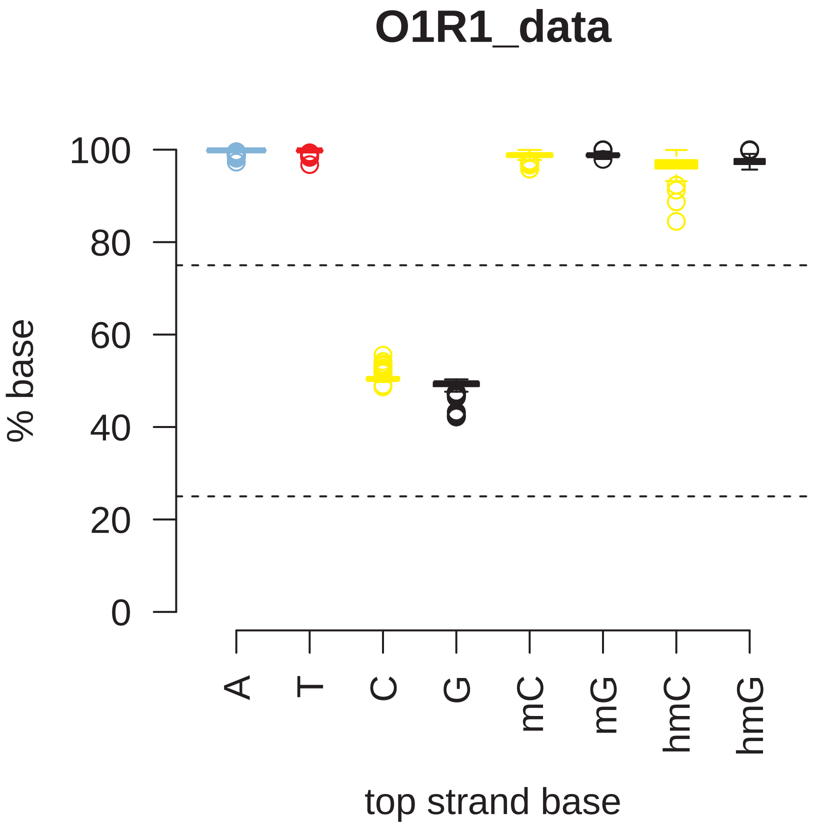
O1R1_data
% base
100
80
60
40
20
0
A
T
C
G
mC
mG
hmC
hmG
top strand base

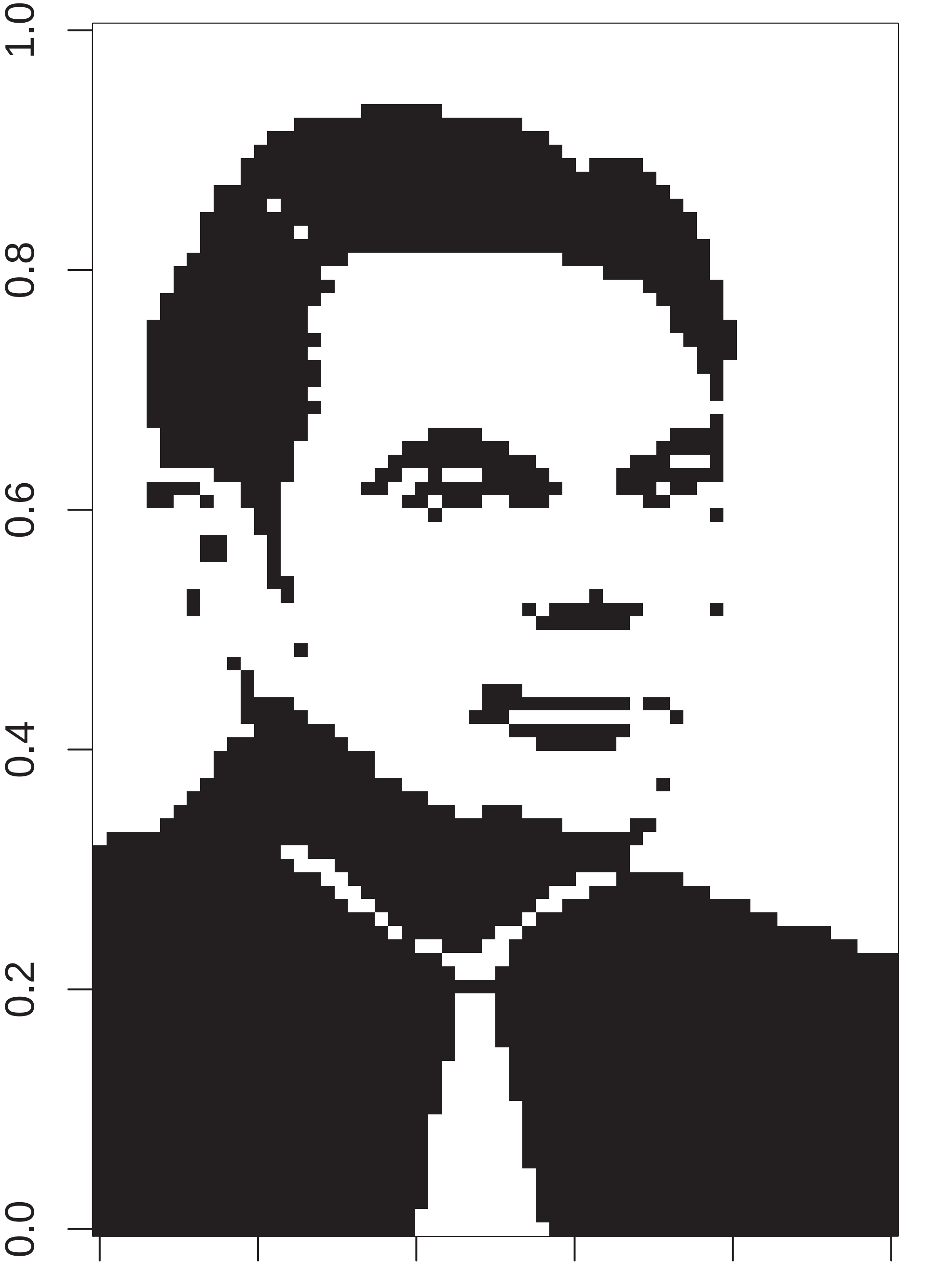
1.0
0.8
0.6
0.4
0.2
0.0
0.0
0.2
0.4
0.6
0.8
1.0

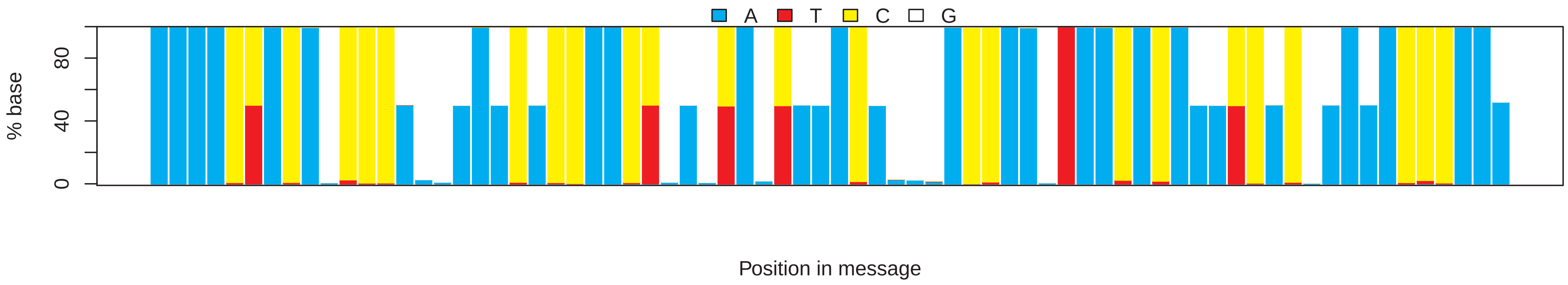

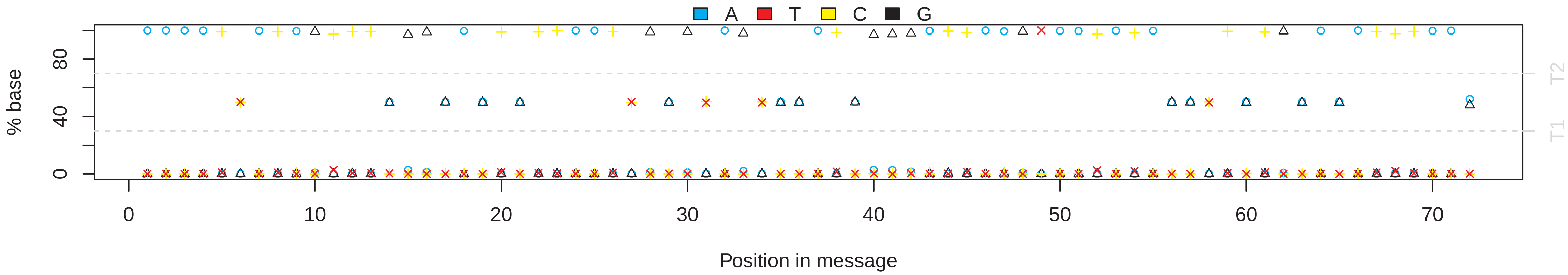

00001101000010100000011001100010010000011001100010000000011010000001000000

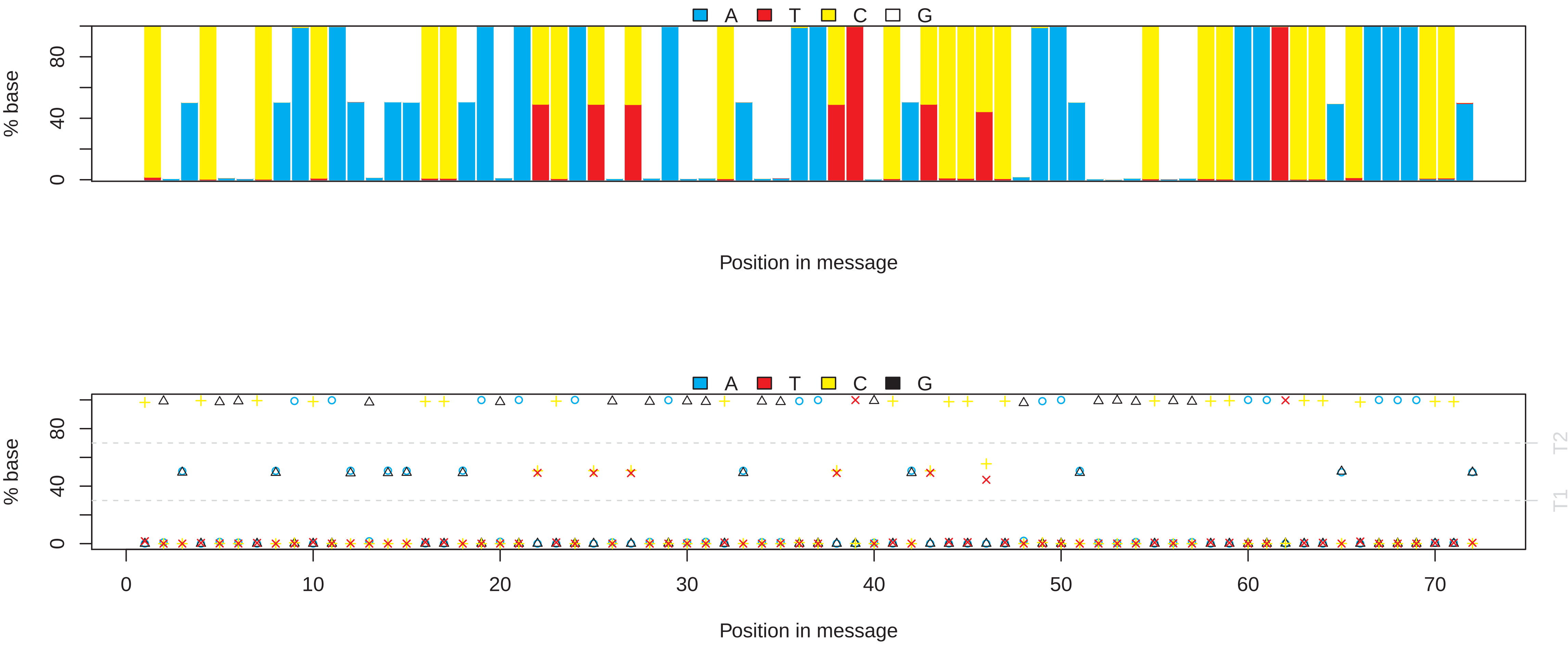

0001101000001000000001101010011100000110101001110000011010100111000000110

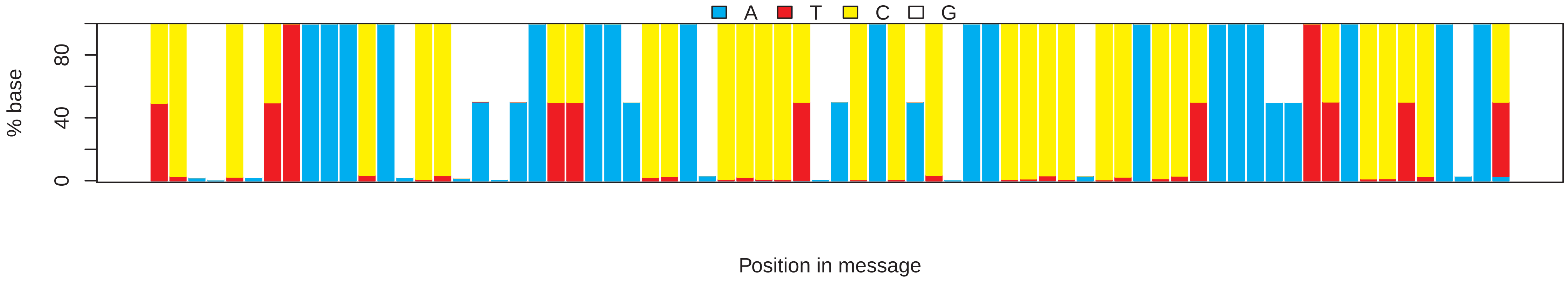

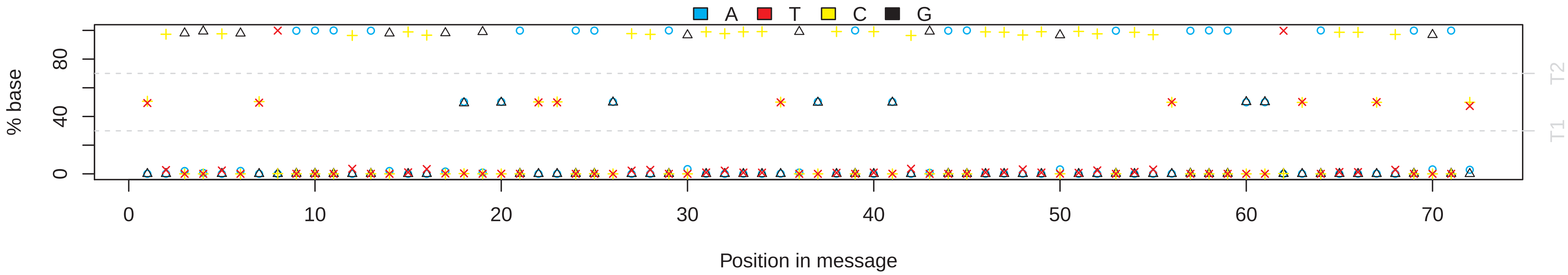

101001110000011010100110000001101110010100000110111001010000011011100101

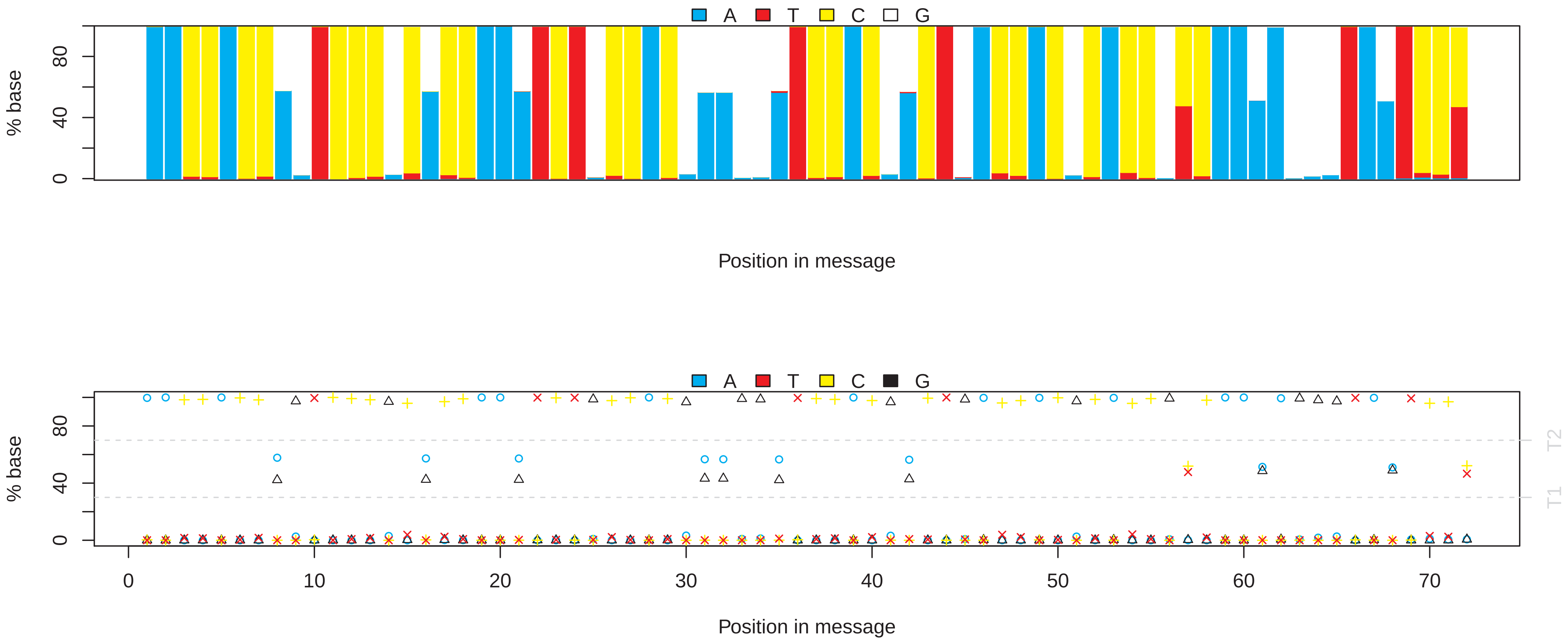

000001101110010000000111001011000001110010110000011100101100000111001011

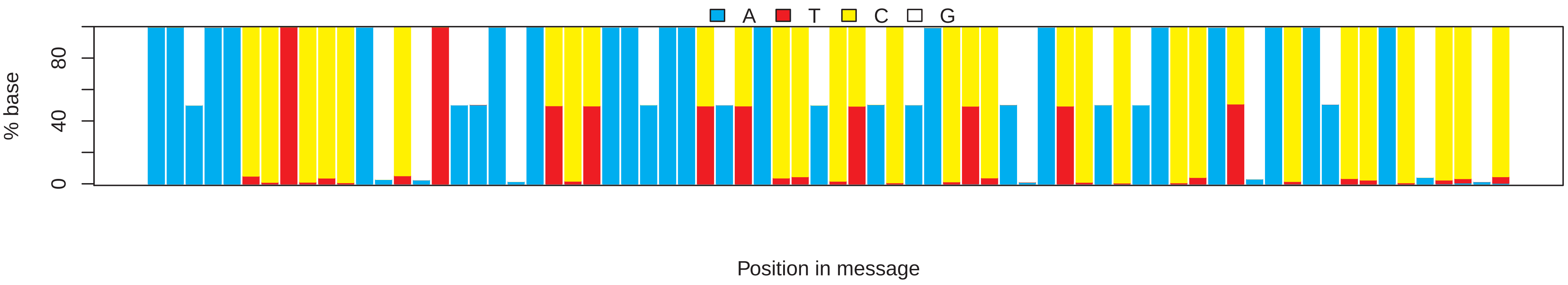

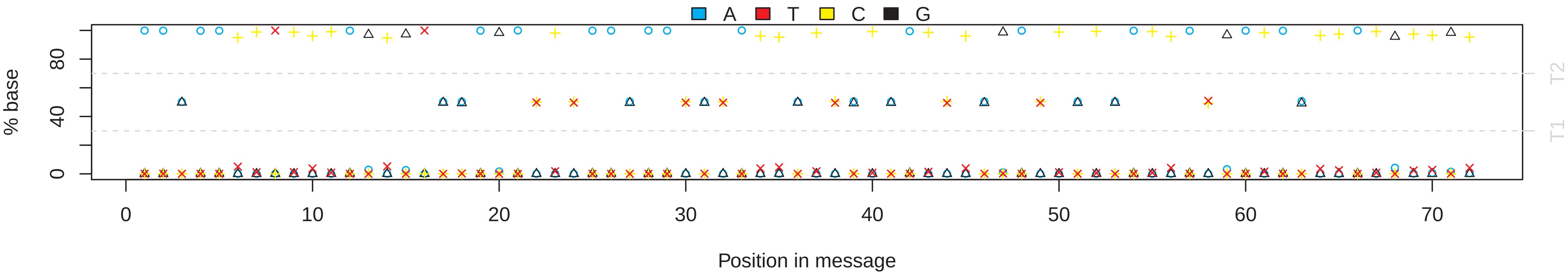

000000111010101100000111000001010000110100110000110100100110100000110000

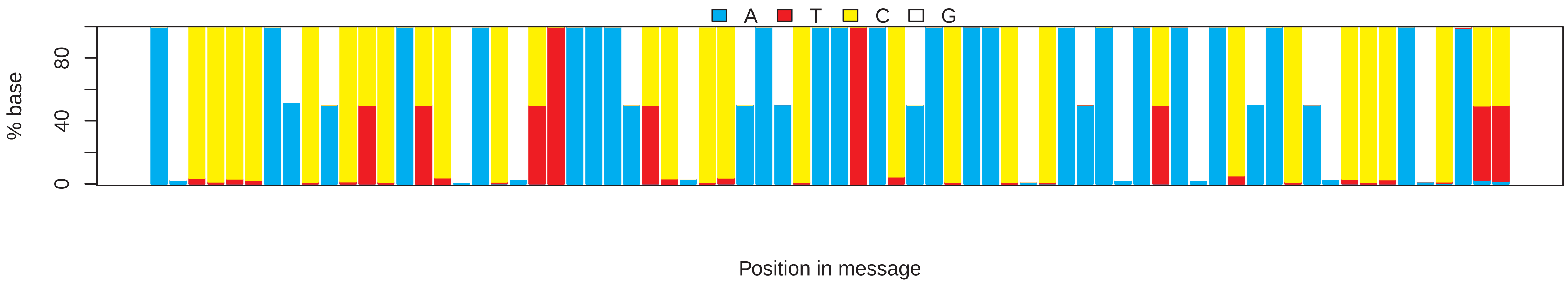

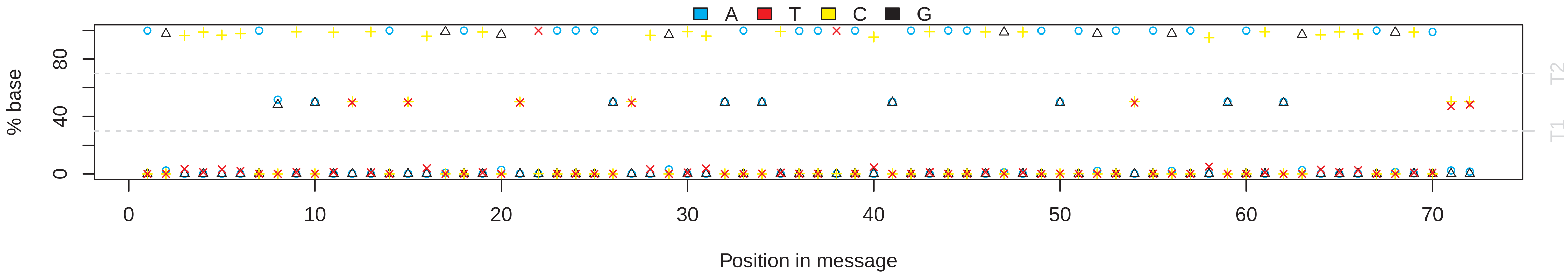

010100001011101000111100001011000010010000100101000101010000101010001011

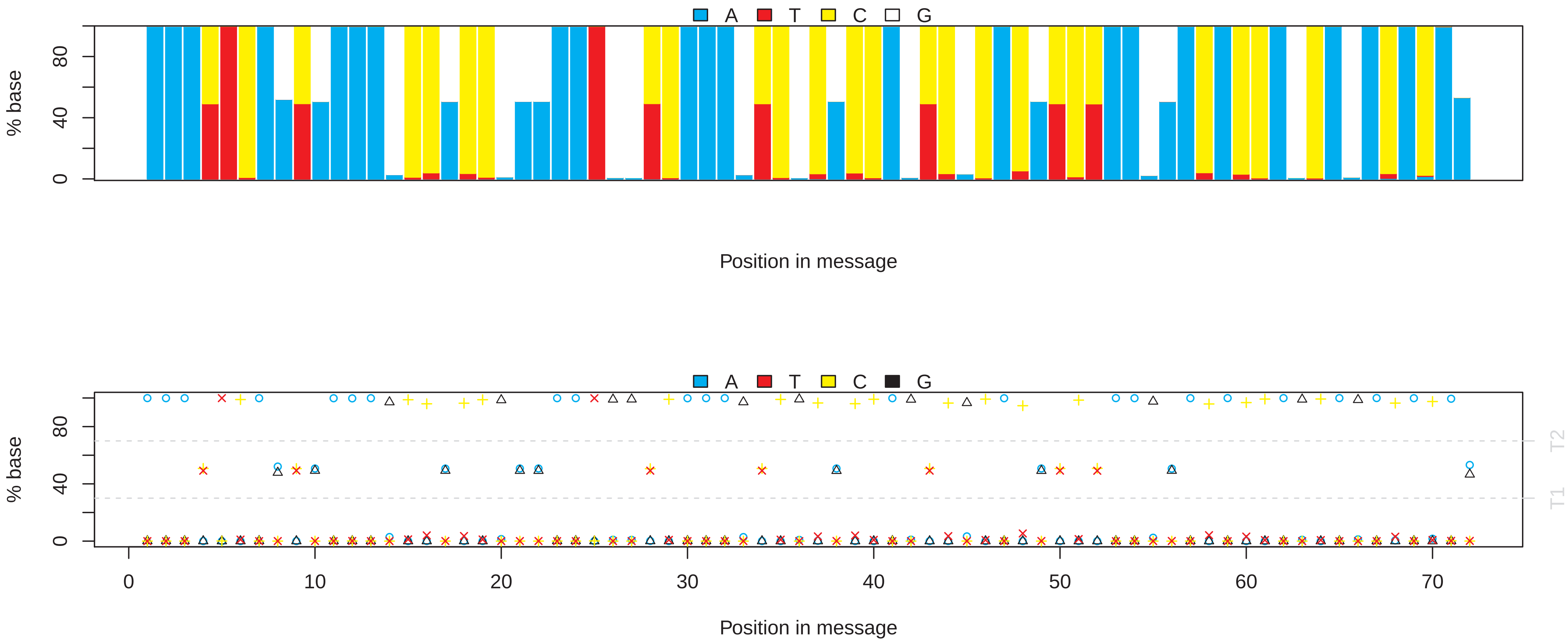

000111001000011000100000100110001110000100101100011100100000100100000100

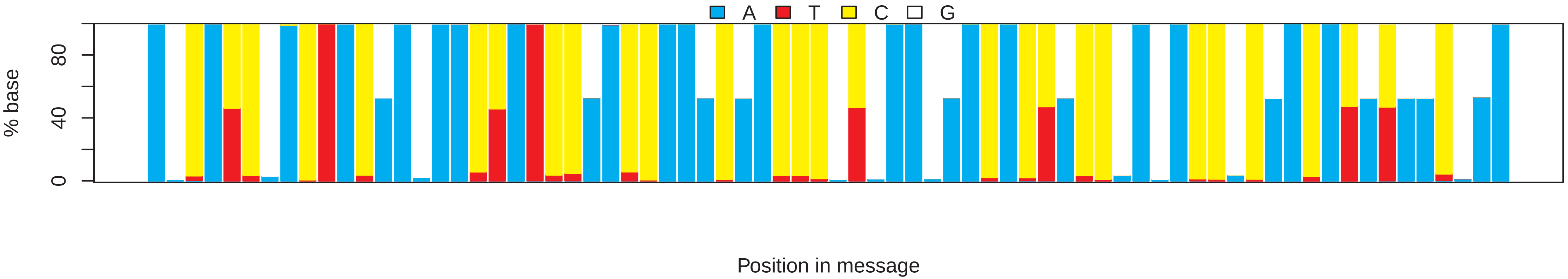

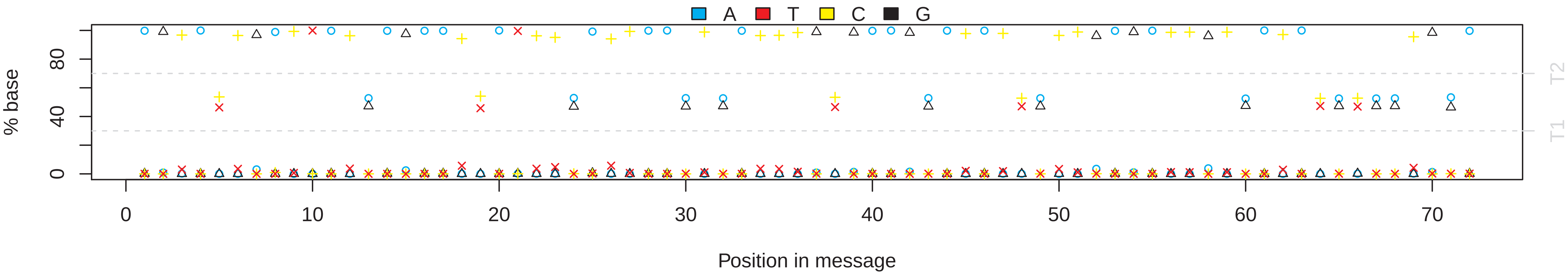

000010101100001000101000001000100001010000001011001100011110000101000000

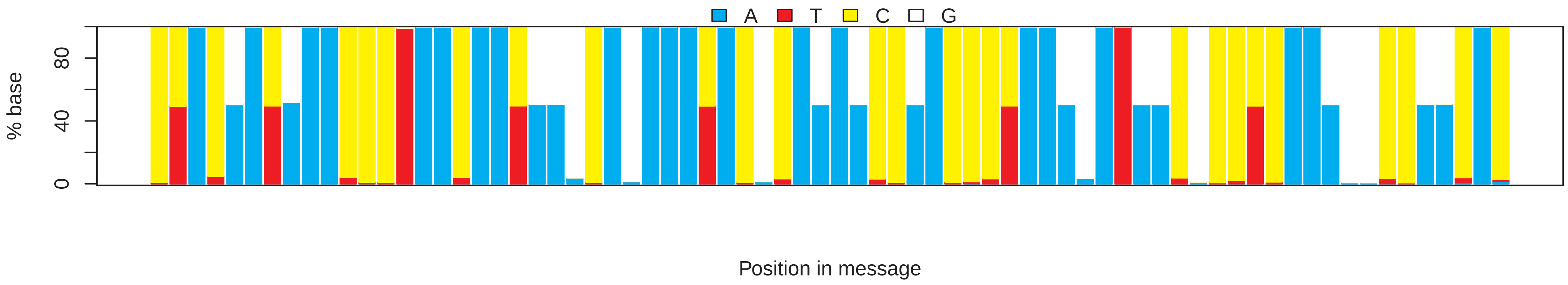

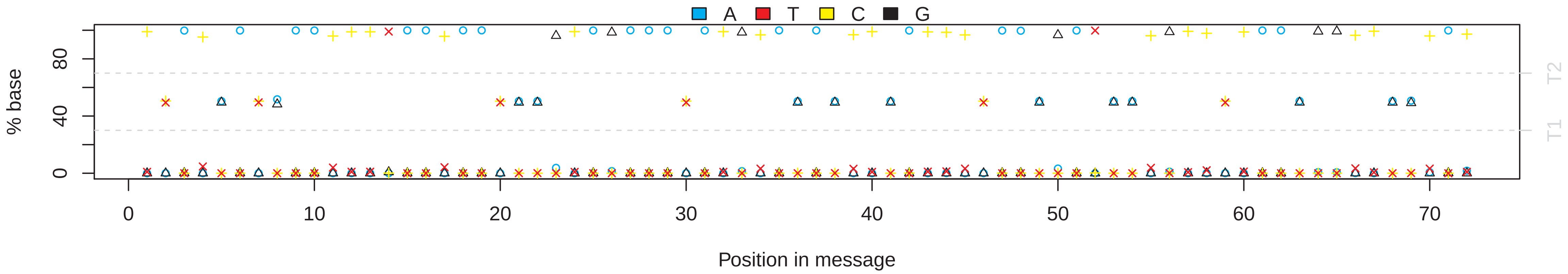

110000100001110000010011000001010000000100110100010100001111000000100001

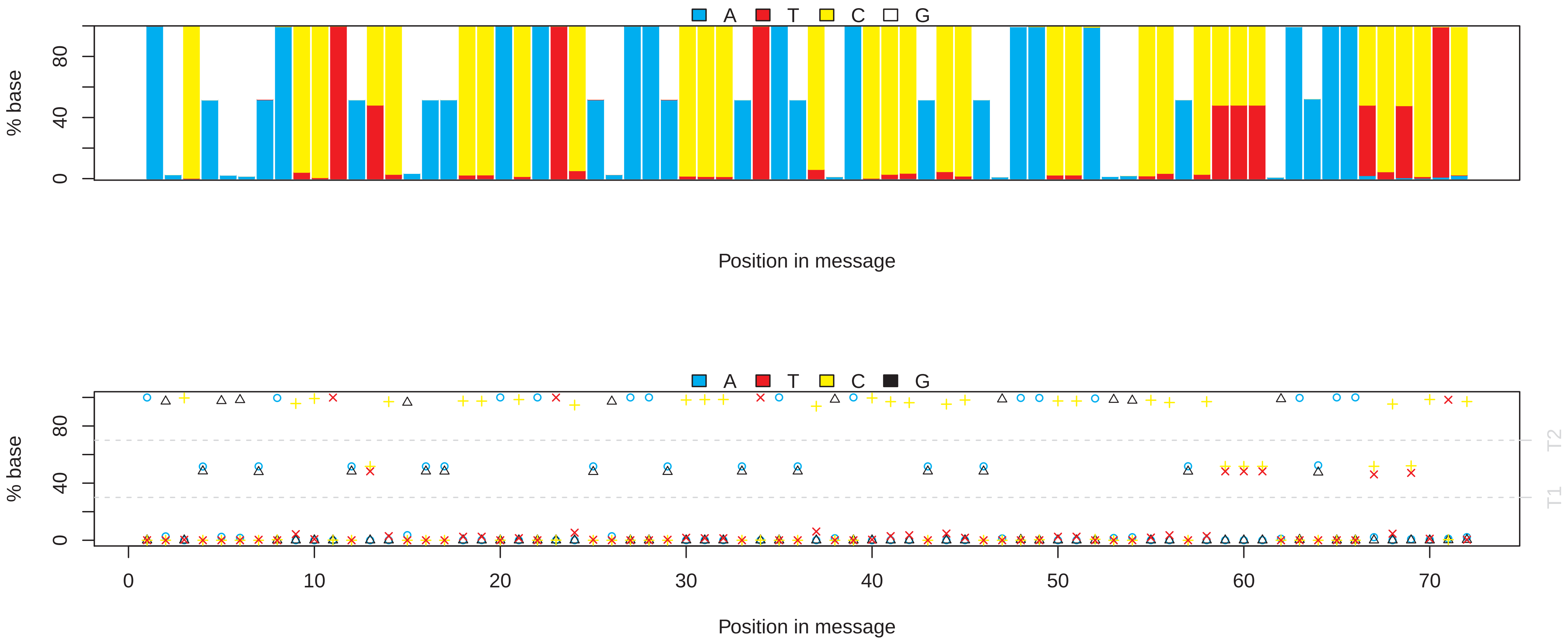

011010000010101000001010010000100100000110001000010011000111100000101111

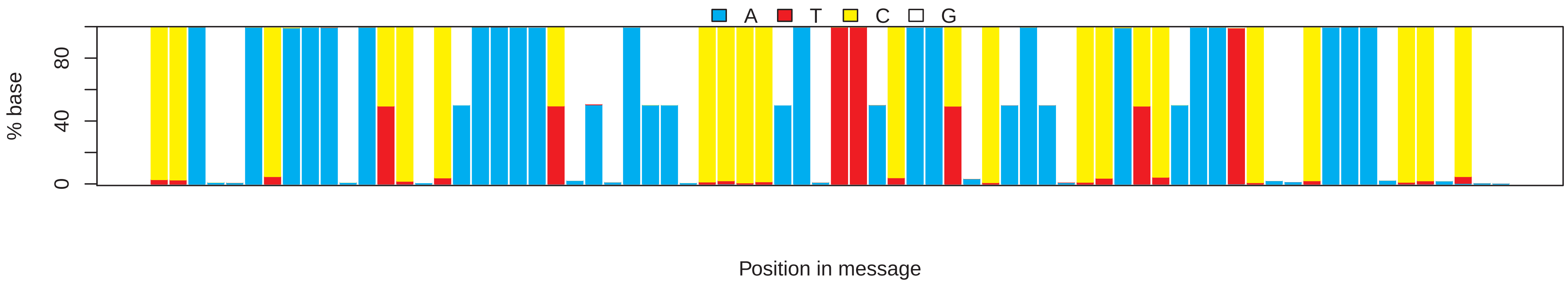

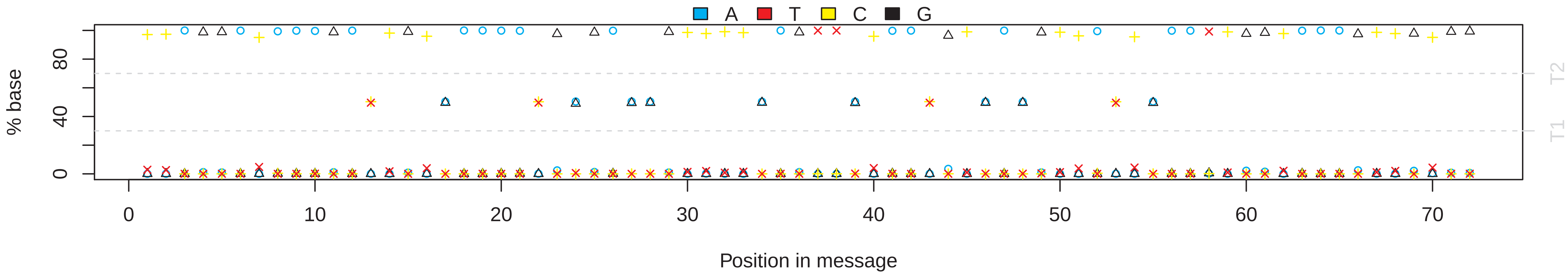

000110000000110000000110000001111000110000111000010010000111100001001000

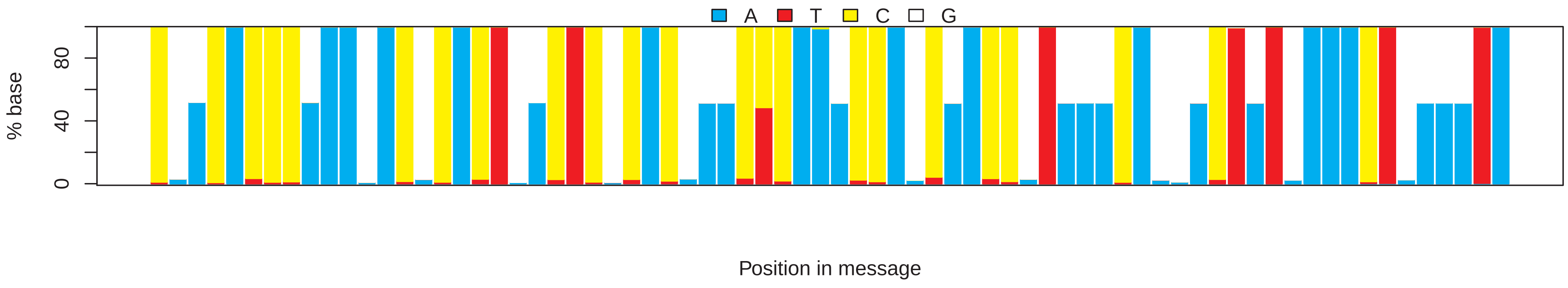

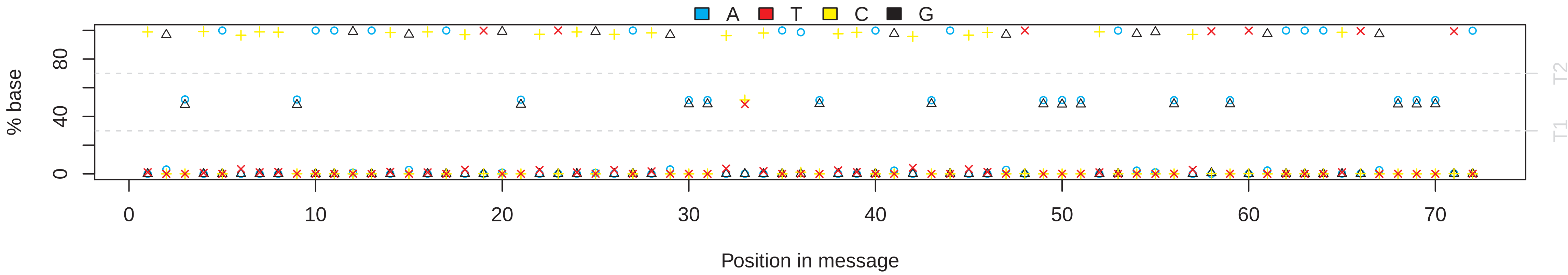

110100110000011100100011000110001100001010000111000101000101100011100010

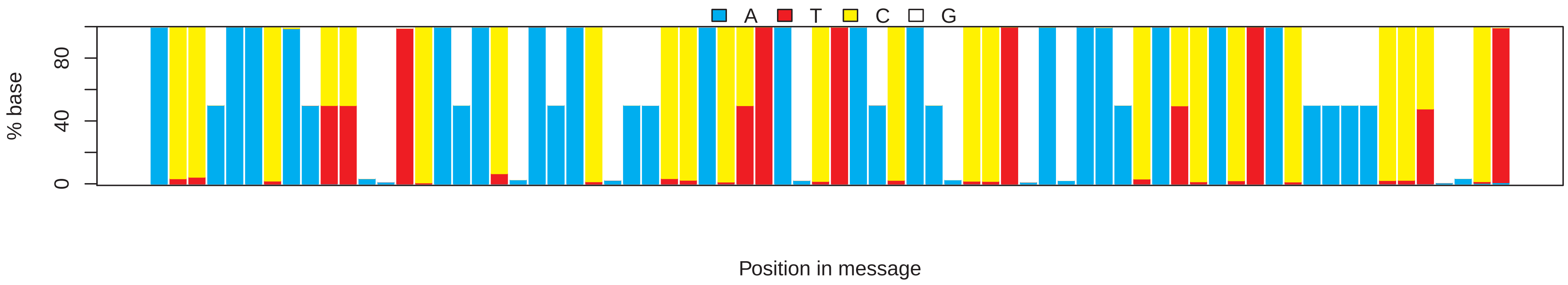

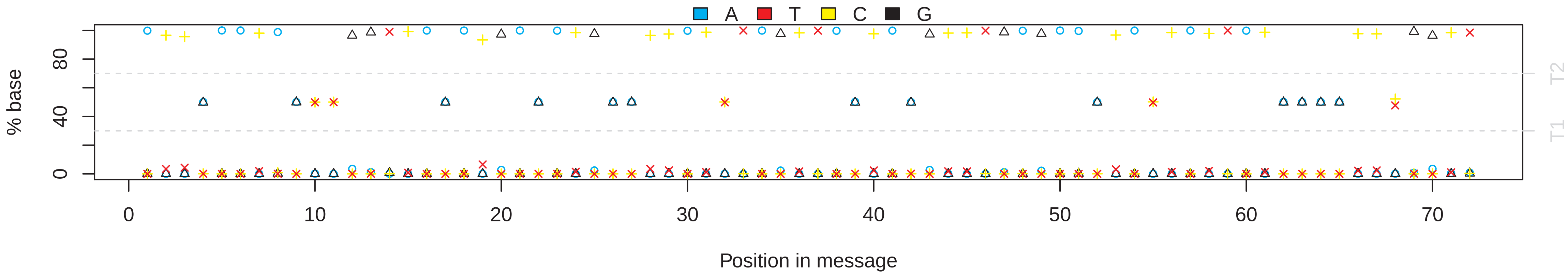

000000100111011000010001100000111011100000111100100000110110100000010111

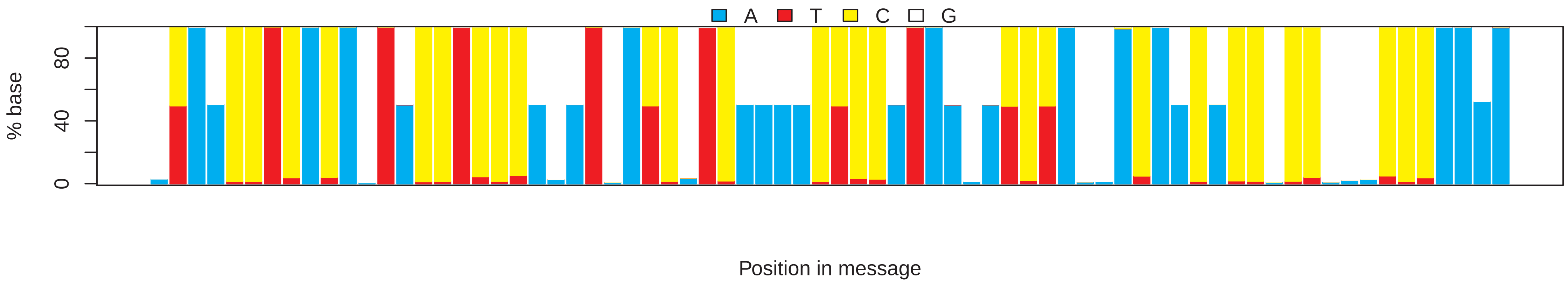

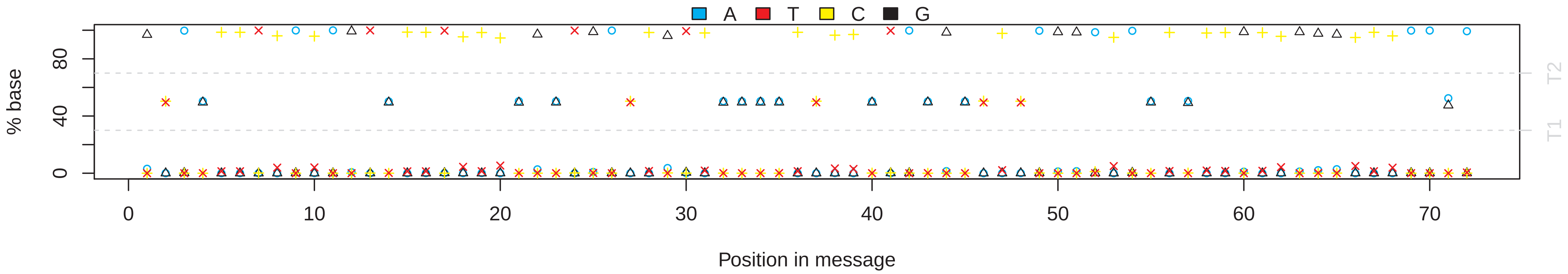

1100111000001011101001010011111000011000100001110110000101101001101000000

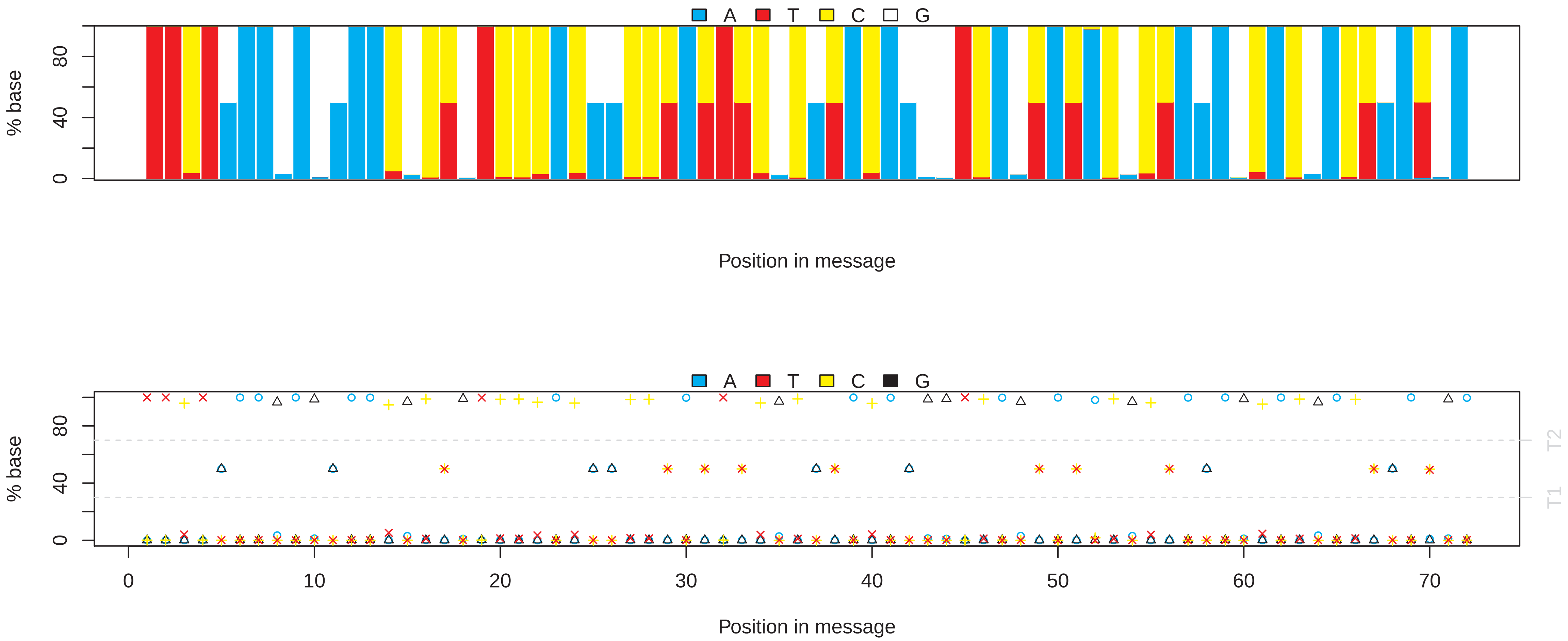

1101000100000011101110000011101110110100000011011010110100000001101100100

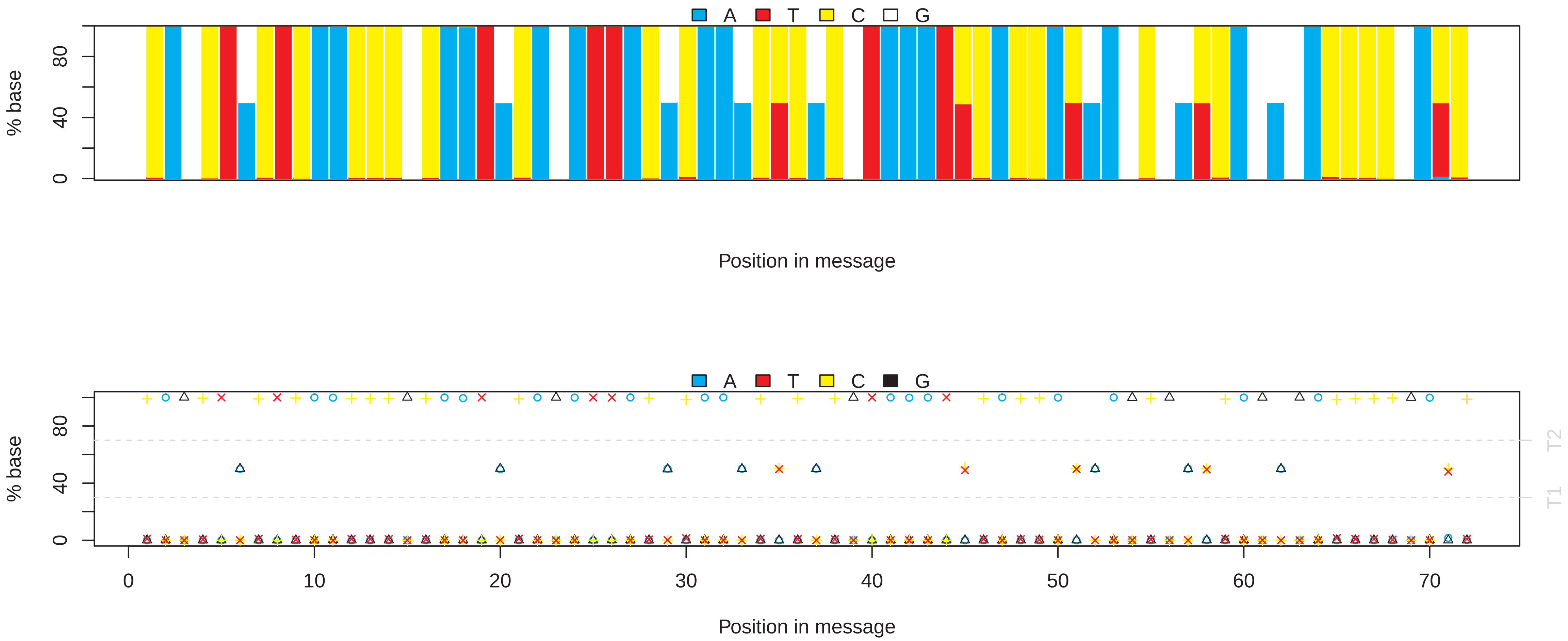

000110011000010100100010110100000111010100011101101001110100001001011010

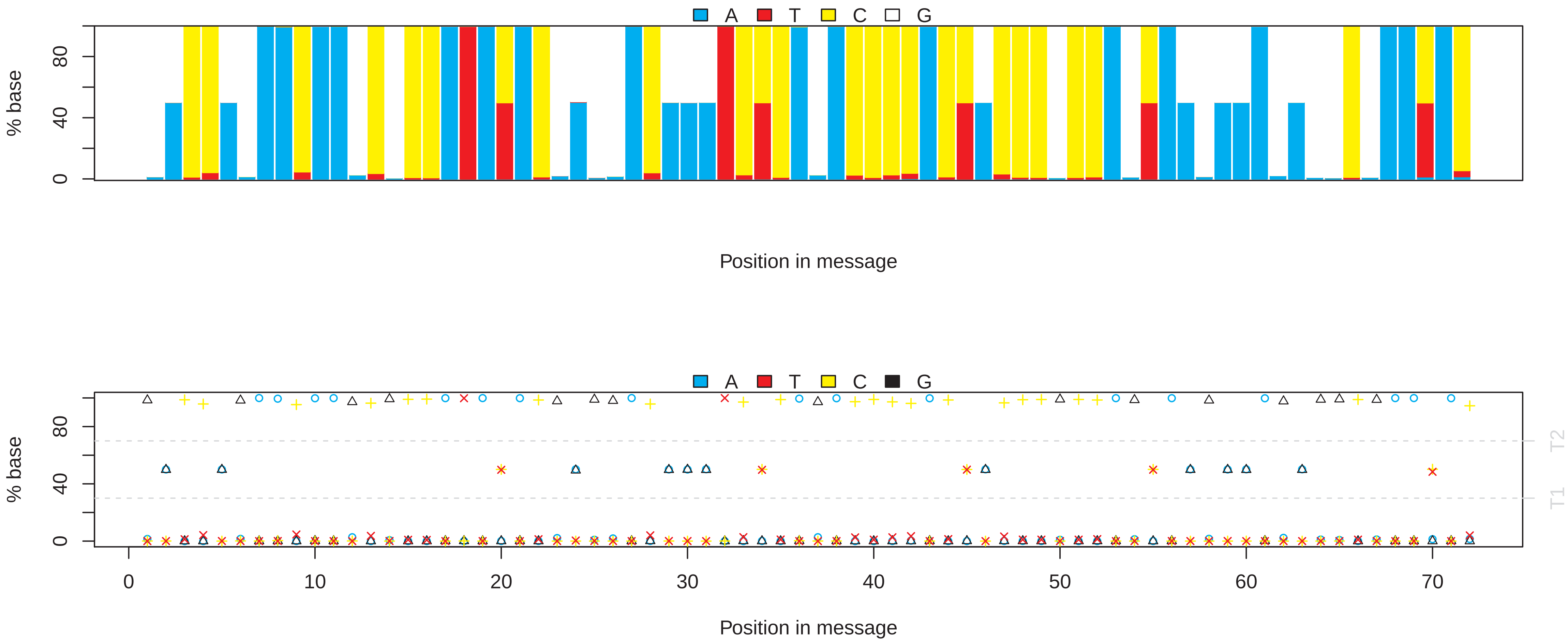

1010010000010011010101100100000101101001000110011011001000000010001000100

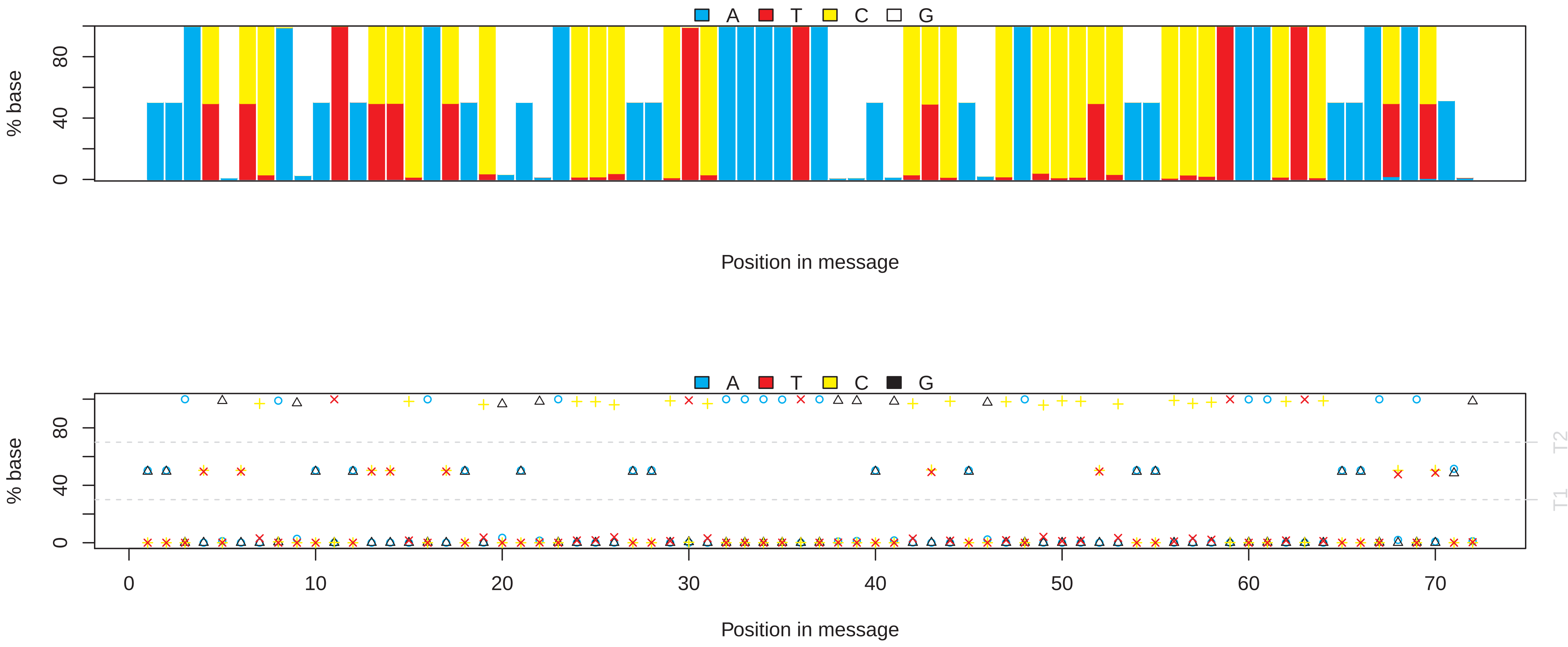

000101001010111010010001100011000001011010110110011100010110011100010100

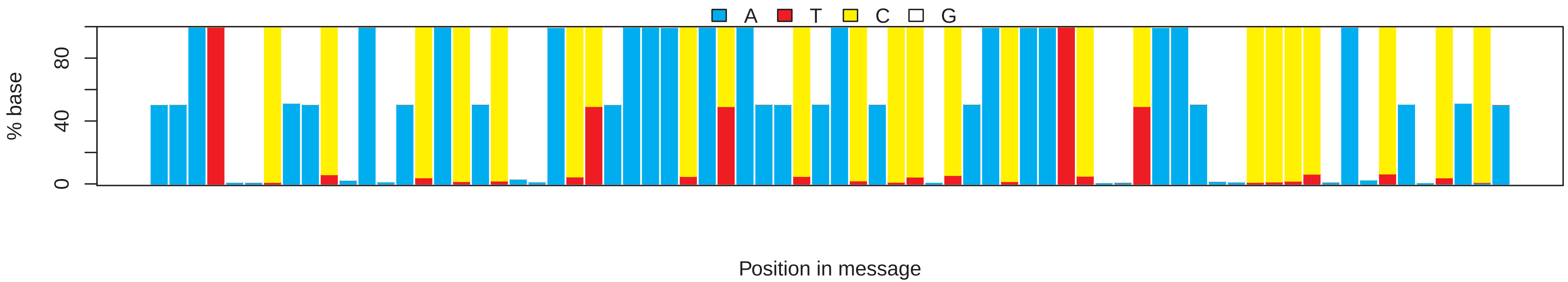

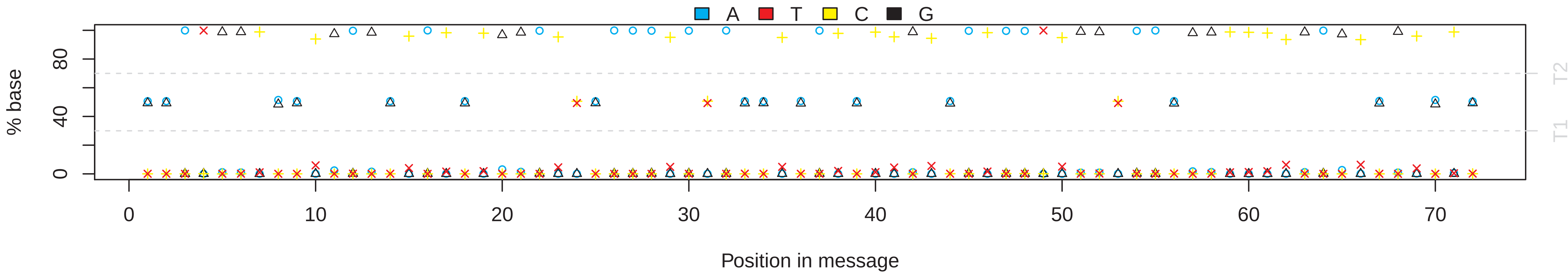

0001001000101000101100010000001000000101000001001000100000111000100000010

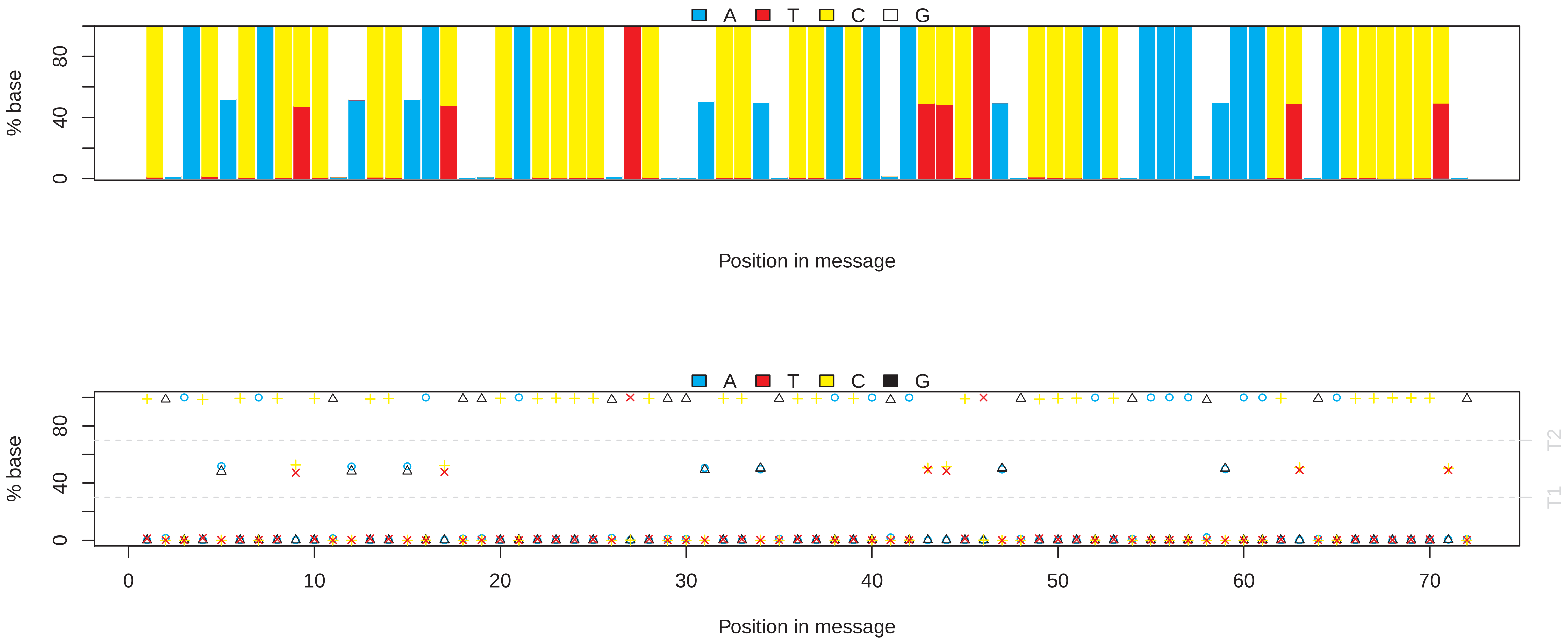

0100010110000100101000011110000110000000101111000110100001000110000011110

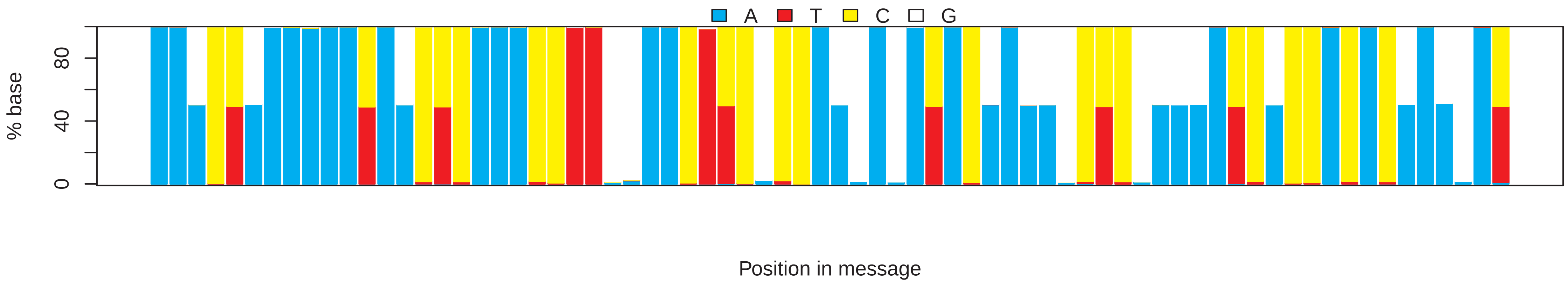

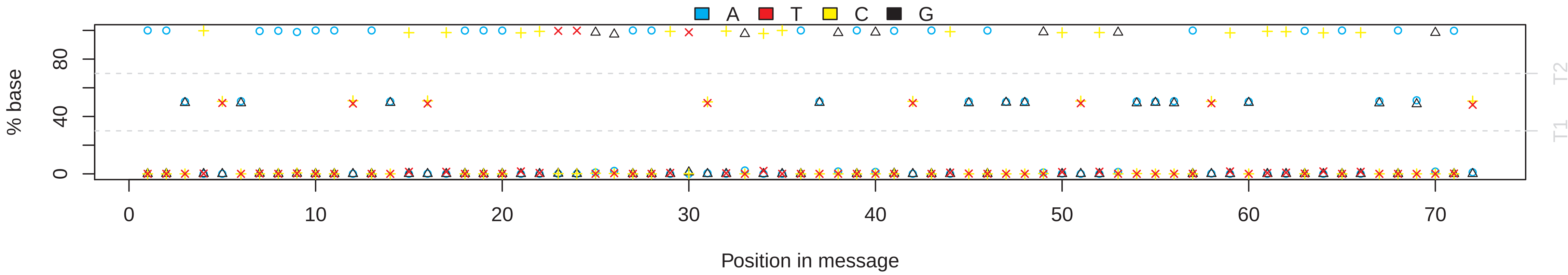

000110000001000110000111010001101000010001010000011100000110100001000001

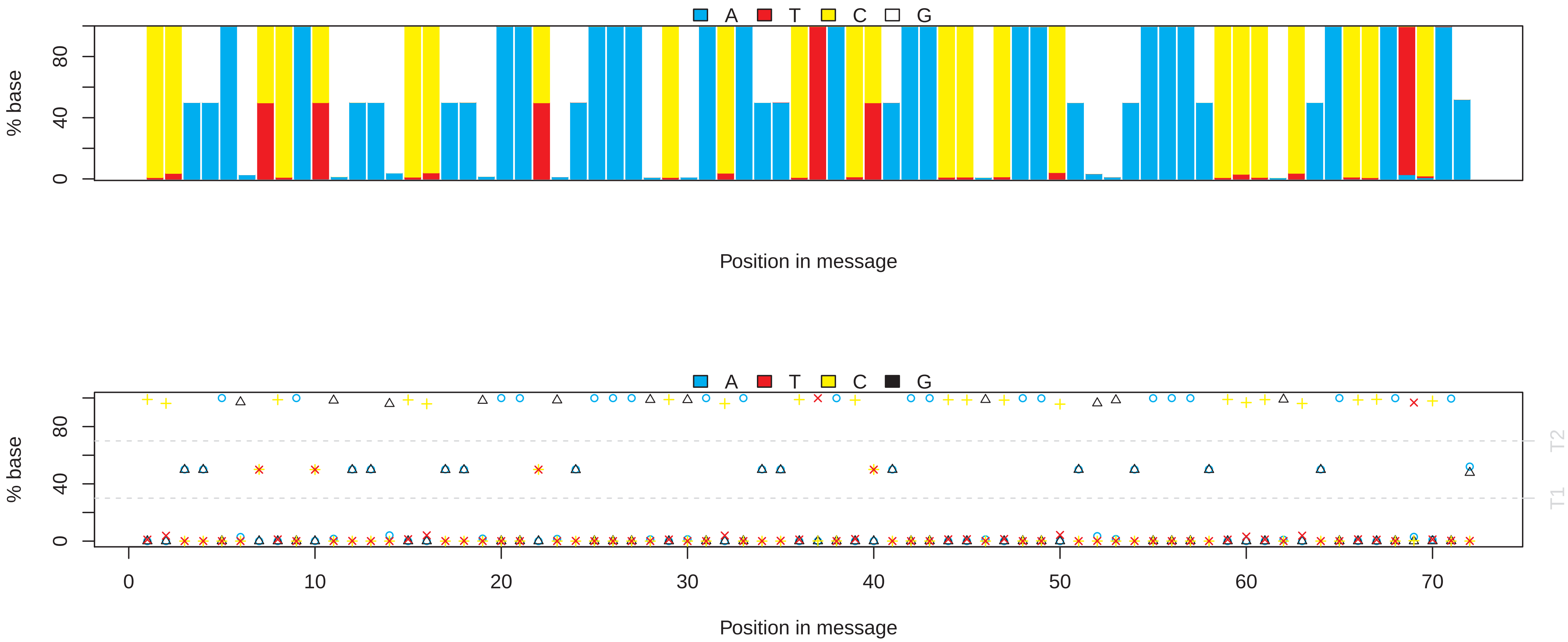

100001110100011000000100000010000001101100011010000100000010100001101100

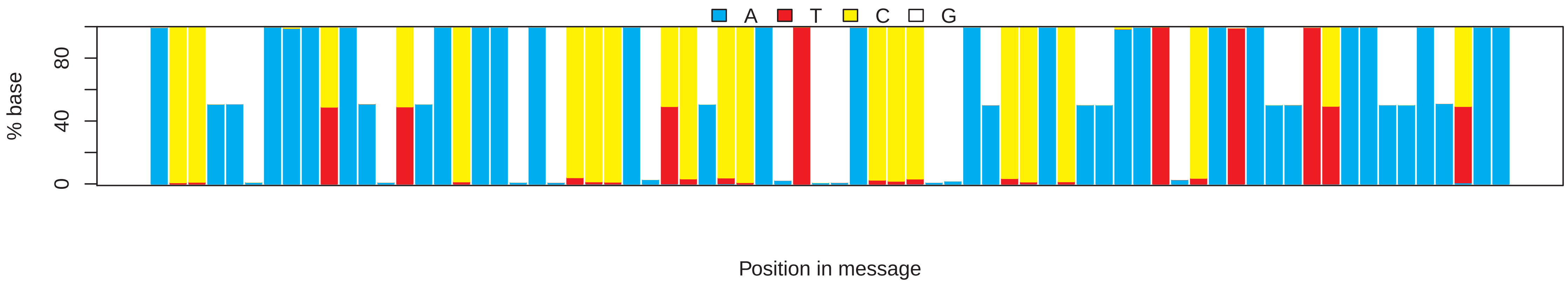

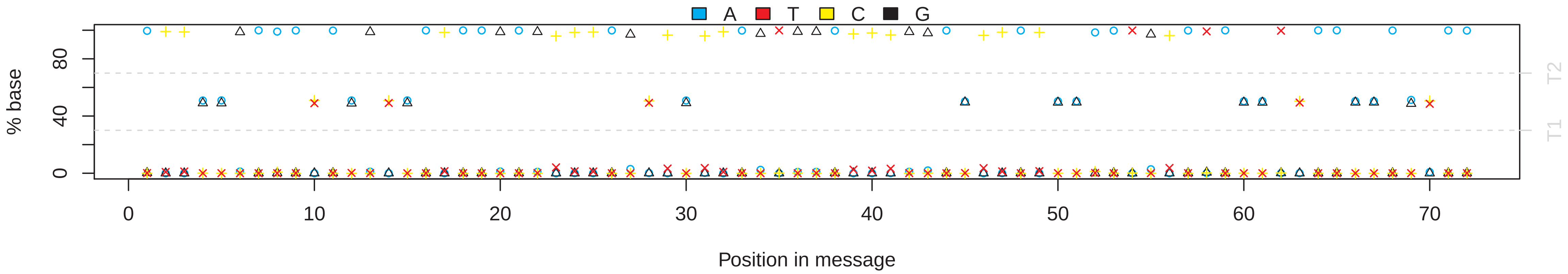

011000000100010010000001101100010110000100100010100001100100011000000100

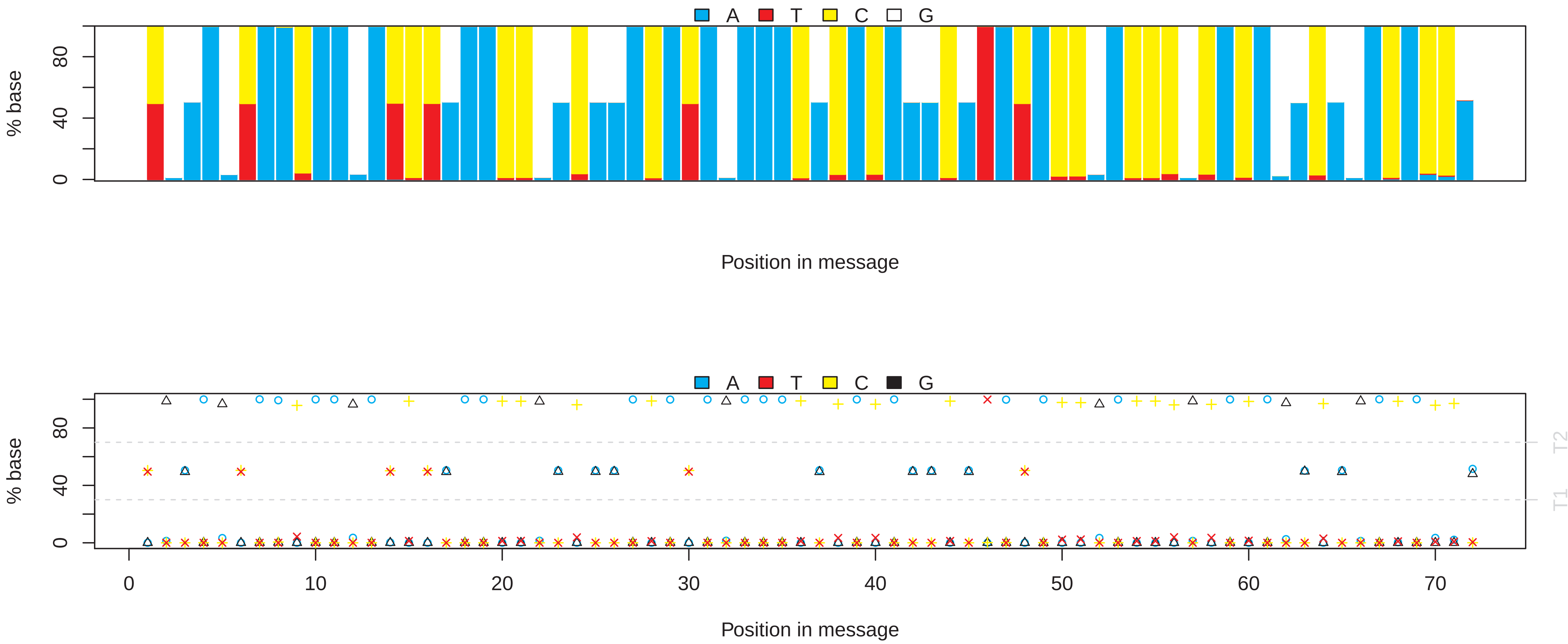

100011000001011100011000000101000001000000010101000101100001010000010110

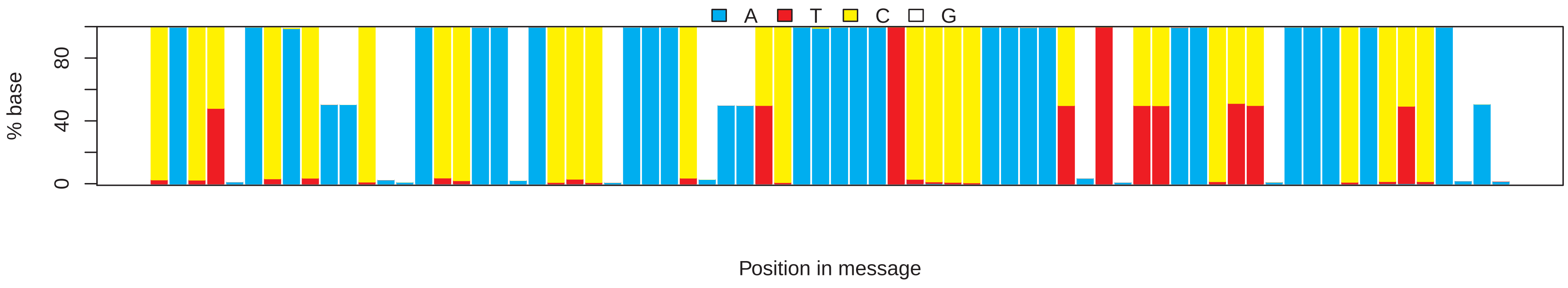

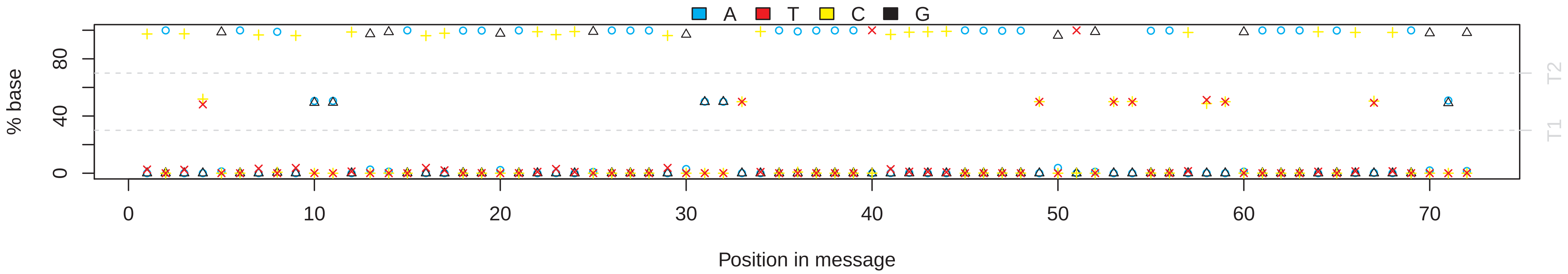

0001000000011000000101010000001001100000101110000111011001110000101110000

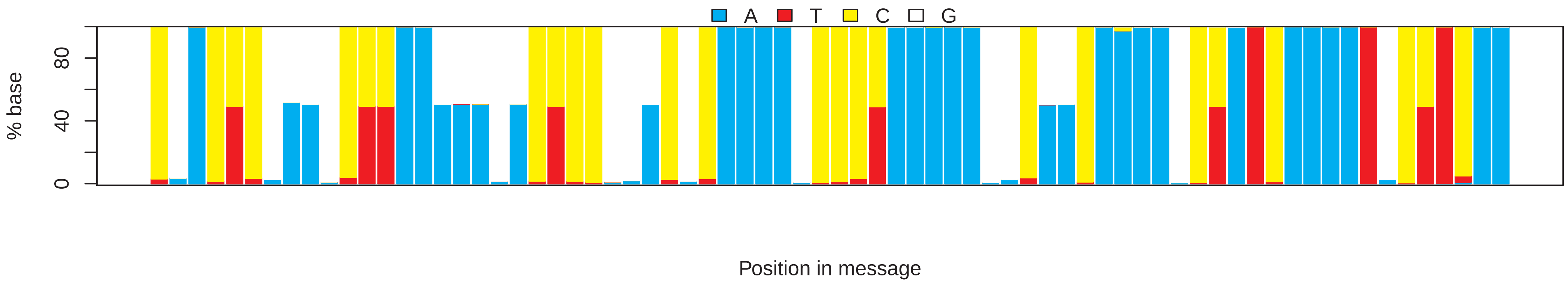

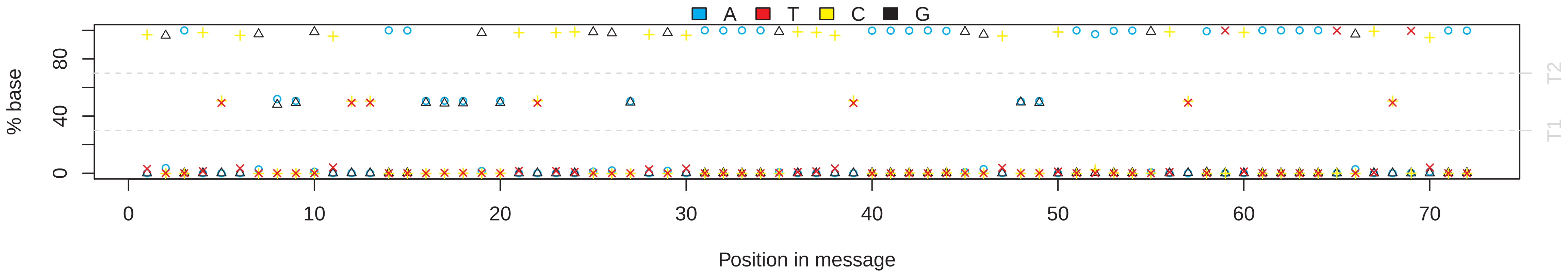

01001010000110000000111101001000000110100000001000100000110110000011111000

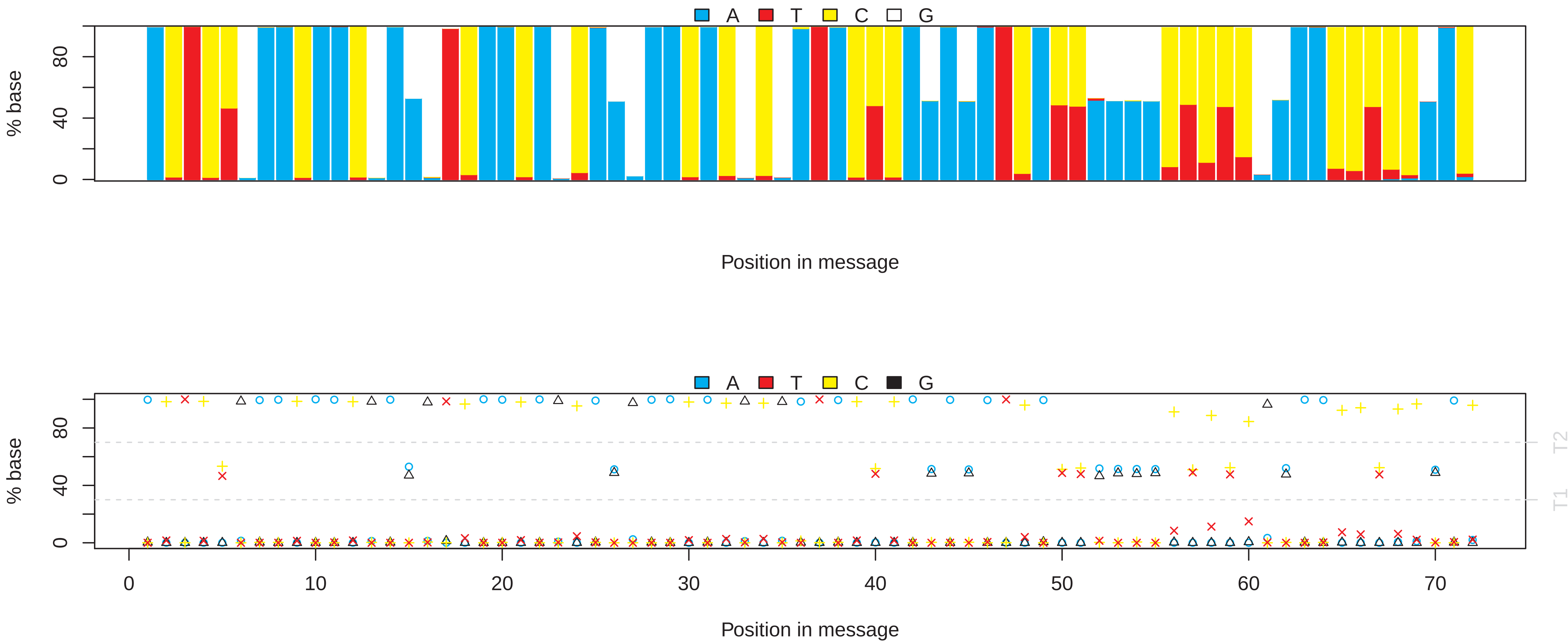

0111100010010000110010000010010000000101110000010011000001010100000101001

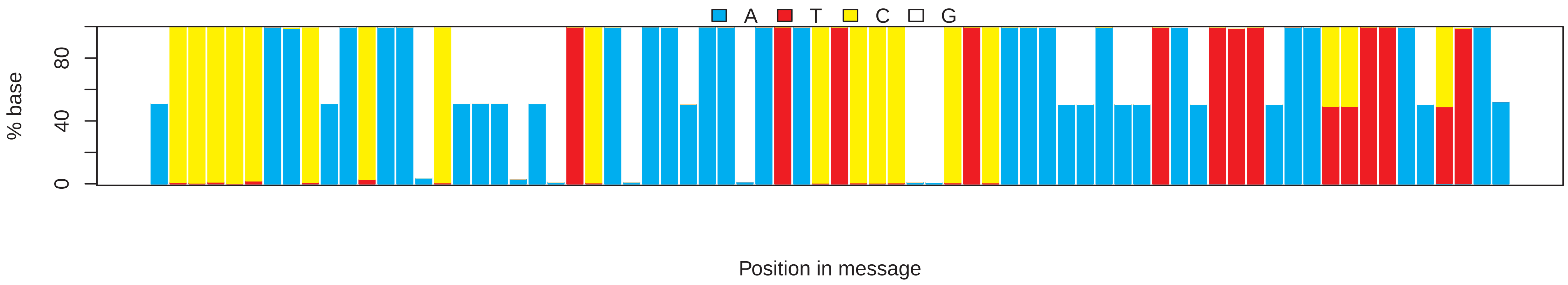

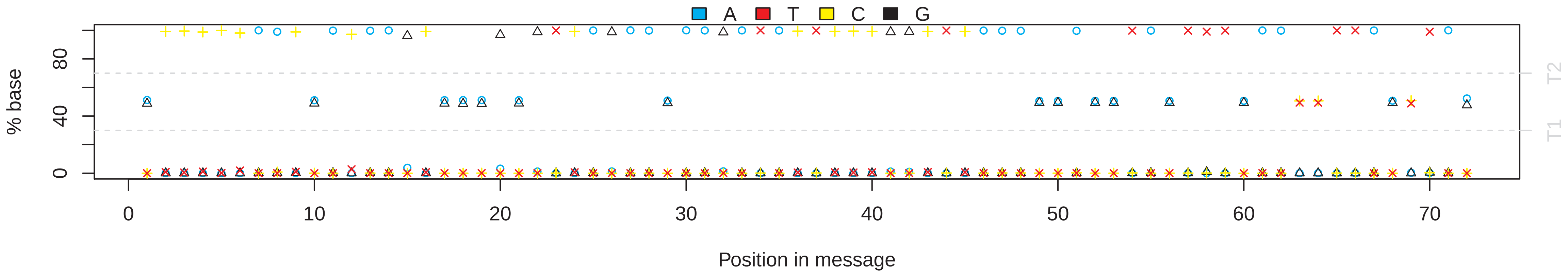

000010001000001100010011000000000101111100111000000001001110001111001100

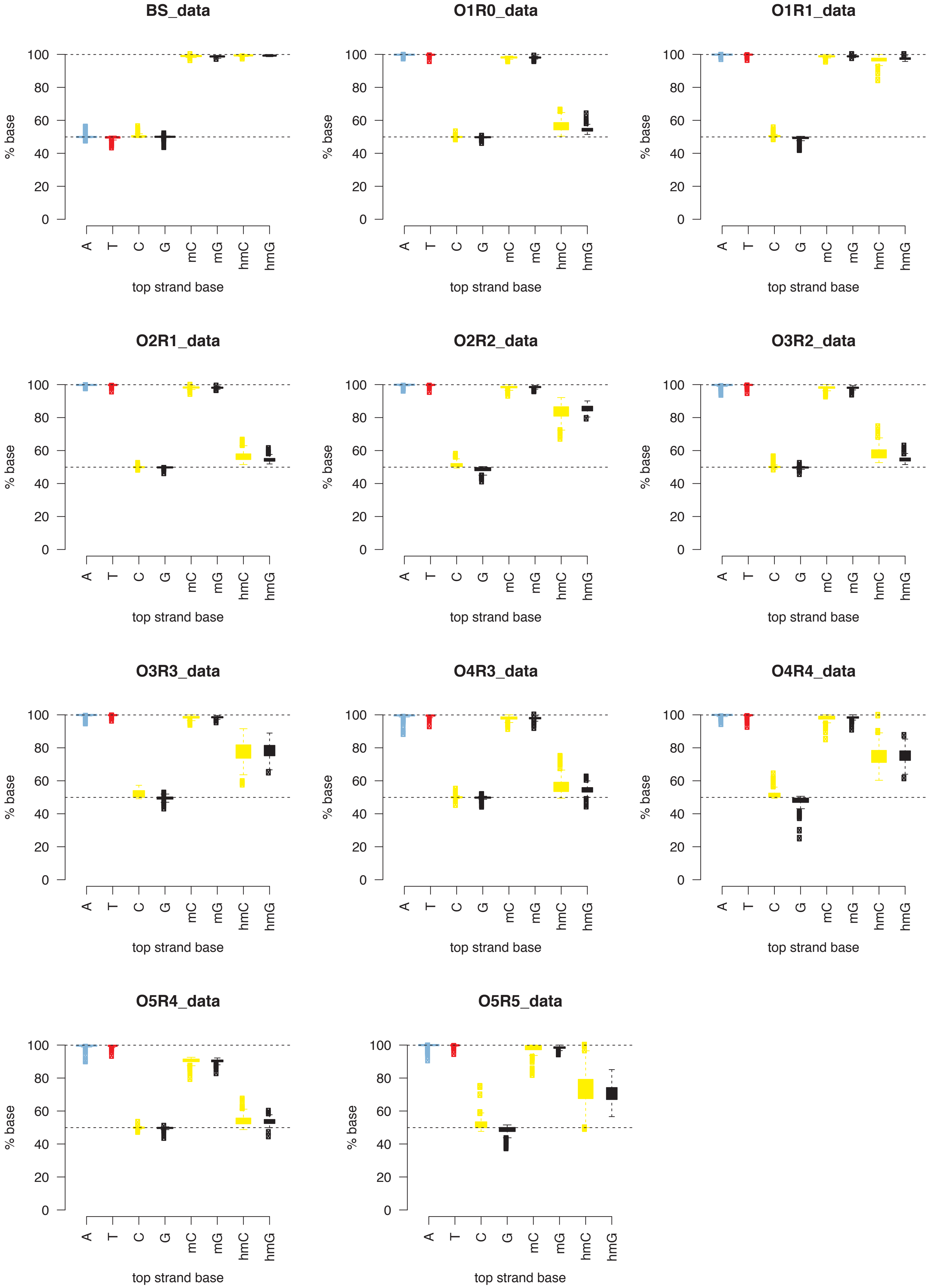
BS_data
O1R0_data
O1R1_data
O2R1_data
O2R2_data
O3R2_data
O3R3_data
O4R3_data
O4R4_data
O5R4_data
O5R5_data
% base
0
20
40
60
80
100
A
T
C
G
mC
mG
hmC
hmG
top strand base
